# Prolactin-Releasing Peptide Differentially Regulates Gene Transcriptomic Profiles in Mouse Bone Marrow-Derived Macrophages

**DOI:** 10.3390/ijms22094456

**Published:** 2021-04-24

**Authors:** Yulong Sun, Zhuo Zuo, Yuanyuan Kuang

**Affiliations:** 1 School of Life Sciences, Northwestern Polytechnical University, Xi’an 710072, China; zuozhuo@mail.nwpu.edu.cn (Z.Z.); kuangyuanyuan@mail.nwpu.edu.cn (Y.K.); 2 Key Laboratory for Space Biosciences & Biotechnology, Institute of Special Environmental Biophysics, School of Life Sciences, Northwestern Polytechnical University, Xi’an 710072, China

**Keywords:** prolactin-releasing peptide, GPR10, bone marrow-derived macrophage, RNA sequencing, bioinformatics, transcriptomic profiles

## Abstract

Prolactin-releasing Peptide (PrRP) is a neuropeptide whose receptor is GPR10. Recently, the regulatory role of PrRP in the neuroendocrine field has attracted increasing attention. However, the influence of PrRP on macrophages, the critical housekeeper in the neuroendocrine field, has not yet been fully elucidated. Here, we investigated the effect of PrRP on the transcriptome of mouse bone marrow-derived macrophages (BMDMs) with RNA sequencing, bioinformatics, and molecular simulation. BMDMs were exposed to PrRP (18 h) and were subjected to RNA sequencing. Differentially expressed genes (DEGs) were acquired, followed by GO, KEGG, and PPI analysis. Eight qPCR-validated DEGs were chosen as hub genes. Next, the three-dimensional structures of the proteins encoded by these hub genes were modeled by Rosetta and Modeller, followed by molecular dynamics simulation by the Gromacs program. Finally, the binding modes between PrRP and hub proteins were investigated with the Rosetta program. PrRP showed no noticeable effect on the morphology of macrophages. A total of 410 DEGs were acquired, and PrRP regulated multiple BMDM-mediated functional pathways. Besides, the possible docking modes between PrRP and hub proteins were investigated. Moreover, GPR10 was expressed on the cell membrane of BMDMs, which increased after PrRP exposure. Collectively, PrRP significantly changed the transcriptome profile of BMDMs, implying that PrRP may be involved in various physiological activities mastered by macrophages.

## 1. Introduction

Prolactin-releasing peptide (PrRP) is a neuropeptide originally extracted from the bovine hypothalamus [1,2]. PrRP has two biologically active isoforms, PrRP20 and PrRP31, which share identical C-terminal Arg-Phe-amide sequences [3,4]. GPR10 (G-protein coupled receptor 10, GPCR 10) is considered as the endogenous receptor for PrRP [2,5]. Both isoforms PrRP20 and PrRP31 bind to GPR10 with high affinity [6]. PrRP belongs to a family named RF-amide, which includes neuropeptide FF (NPFF), gonadotropin-inhibitory hormone (GnIH), kisspeptin, and pyroglutamylated RFamide (QRFP) [7].

The original biological function of PrRP is to promote the release of prolactin from cultured pituitary cells [2]. Then, follow-up research results show that PrRP has an important role in the neuroendocrine system, including reducing food intake in fasting and free-eating rat models [8], regulating energy homeostasis and increasing energy expenditure [9], mediating the anorexia effect of nerves [10], protecting neuron on several rodent neurodegenerative disorder models [11], and participating in stress response [12]. However, the effect of PrRP on macrophages has not been reported yet.

The regulatory effect of PrRP on leukocytes has been investigated. Romeroa and colleagues find that PrRP mRNA is expressed in leukocytes from the head kidney and blood of *Salmon salar*. Moreover, synthetic PrRP promotes the expression of pro-inflammatory cytokines (IL-12, IL-6, IL-8, and IL-1β), suggesting that PrRP may be a local neurotransmitter of innate immune processes in leukocytes from *Salmon salar* [13]. However, the target genes by which PrRP affects the leukocytes have not been identified yet.

RNA-seq (RNA-sequencing) is a useful method to explore the effect of the peptide on the gene expression profile of macrophages from the transcriptome level [14,15,16]. Hence, it would be useful to investigate the PrRP-induced gene expression profile of macrophages by using RNA-seq. In the present study, the effect of PrRP on the transcriptome of BMDM was studied by the following procedures:

(a) PrRP31-triggered differentially expressed genes (DEGs) were acquired from murine bone marrow-derived macrophages (BMDMs). Cells treated with 1 nM PrRP31 (18 h) were detected by RNA-seq. A total of 410 DEGs were obtained. In addition, the influence of PrRP on the morphology of cells was observed with optical microscopy.
(b) DEGs were analyzed by a series of bioinformatics approaches, including GO analysis, functional enrichment, and protein–protein interaction (PPI) studies. Next, eight hub genes were finally acquired for subsequent study.
(c) The three-dimensional structures of hub proteins were studied. By using homology-modeling, the structure of proteins encoded by hub genes (hub proteins) was built. Subsequently, molecular dynamics simulations (at least 300 ns) were performed to capture the trajectory of hub proteins. Finally, molecular dynamics-optimized protein structures of hub proteins were obtained.
(d) The docking models of PrRP and hub proteins were predicted with the peptide-protein docking module of the Rosetta program.

**Figure 1:**
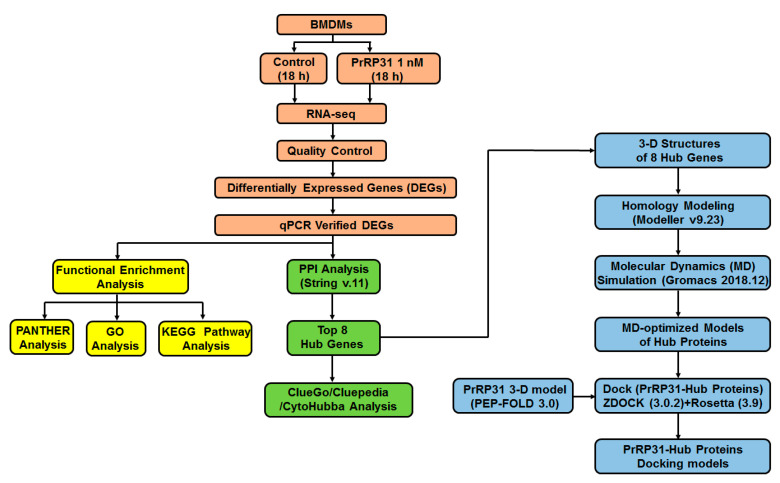
Schematic diagram of this study.

By studying the influence of PrRP on the gene expression of BMDMs at the transcriptome level, we provide clues for exploring the gene expression network of PrRP on macrophages, which will be helpful to investigate the immune-regulating function of PrRP in the future (Figure 1).

## 2. Results

### 2.1. PrRP Demonstrated No Significant Effect on the Shape of BMDMs

**Figure 2:**
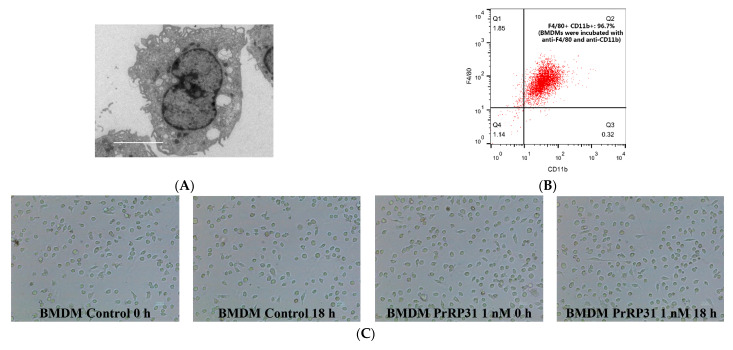
The effect of PrRP31 exposure on BMDMs. (**A**) The cell morphology of BMDMs was examined with electron microscopy. Scale bar, 50 μm. (**B**) BMDMs were analyzed by the flow cytometer with FITC-conjugated anti-CD11b and PE-conjugated anti-F4/80. For the experimental group, the cells were incubated with FITC-conjugated anti-CD11b and PE-conjugated anti-F4/80, and the double-positive rate was 96.7%. (**C**) BMDMs were incubated with vehicle (phosphate buffer saline) or PrRP (1 nM) for 0 h or 18 h, and the morphology of cells was examined under a microscope. Scale bar, 100 μm.

As displayed in Figure 2A, the cell morphology of BMDMs was detected by electron microscopy. In addition, the purity of BMDMs was evaluated using flow cytometry with anti-CD11b and anti-F4/80 (Figure 2B). The results of flow cytometry showed that the double-positive rate of the BMDMs control group (no antibody was added) was 0.14% (the left side of Figure 2B), whereas the double-positive rate of BMDMs (incubated with anti-F4/80 and anti-CD11b) 96.7% (right side of Figure 2B). Hence, these data hinted that the purity of BMDMs was acceptable.

Next, the morphological characters of BMDMs before and after PrRP exposure were examined with an optical microscope. As shown in Figure 2C, BMDMs were oval before PrRP (1 nM) exposure, and the shape of BMDMs did not change remarkably after PrRP exposure for 18 h. Overall, PrRP demonstrated no significant effect on the shape of BMDMs.

### 2.2. Identification of DEGs

**Figure 3:**
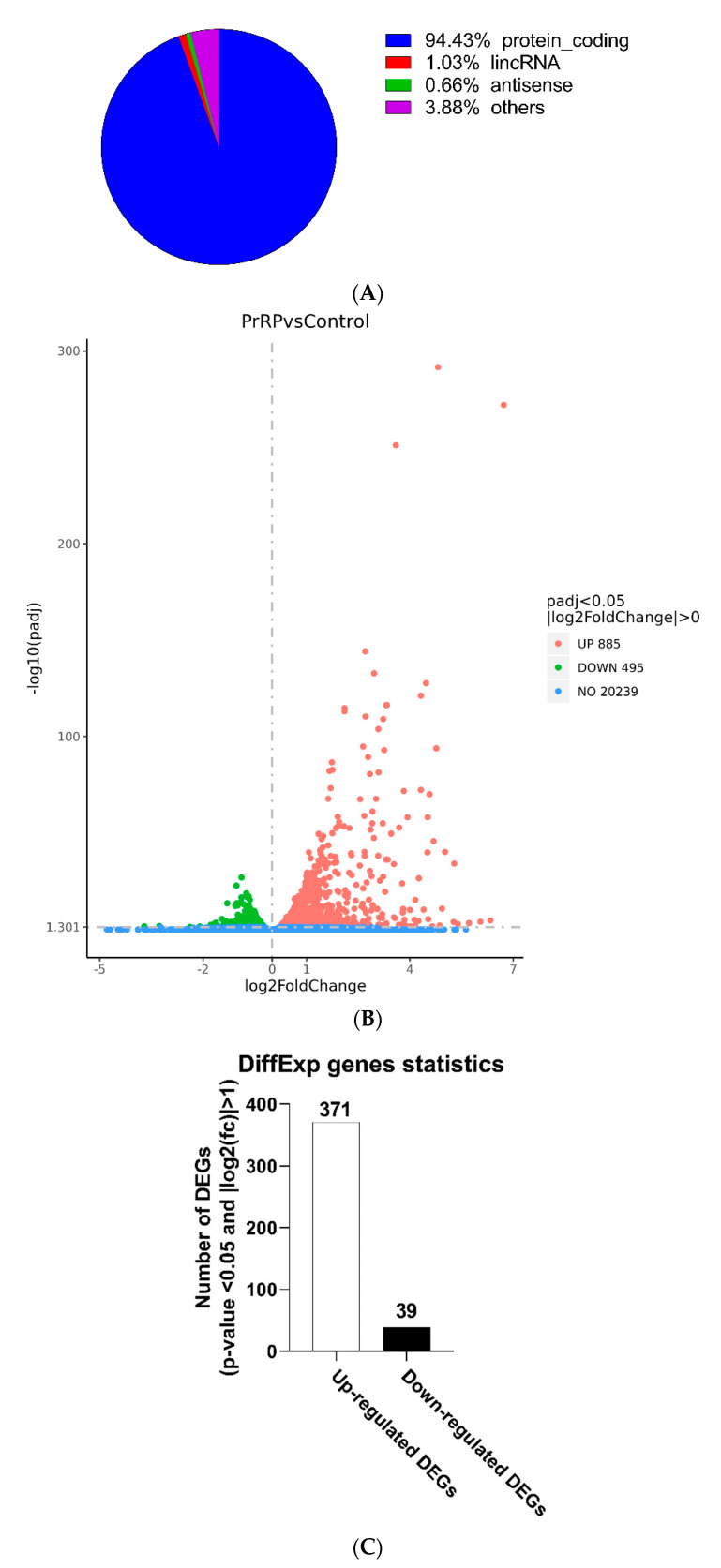

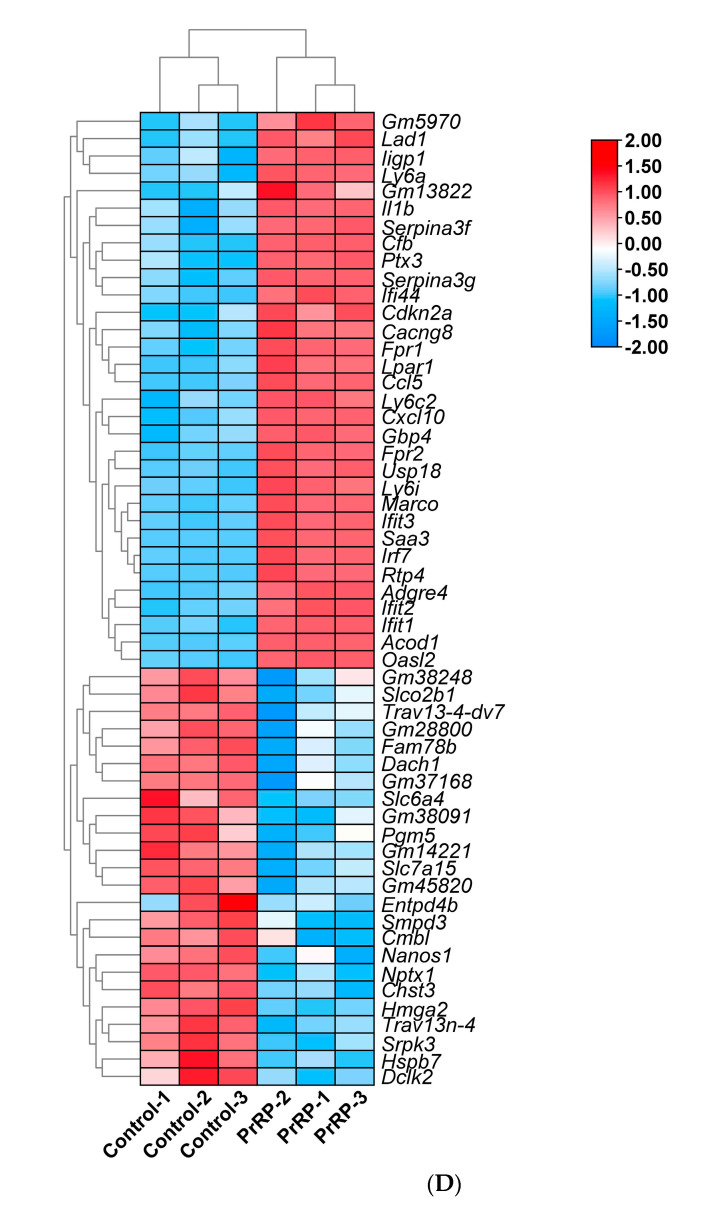
Basic information of the RNA-seq results. (**A**) Proportion plot of the differentiated expressed genes (*p*-value < 0.05 and |log2(fc)| > 1) (DEGs). (**B**) Volcano plot (red dots: up-regulated genes; green dots: down-regulated genes). (**C**) Number of DEGs (*p*-value < 0.05 and |log2(fc)| > 1). (**D**) Heat map of the 24 up-regulated DEGs, 24 down-regulated DEGs, and 8 hub genes, as the ranking was based on the absolute value of the fold of change in descending order. The red indicated the up-regulated genes and blue represented down-regulated genes.

To explore the effect of PrRP on the transcriptome of BMDMs, cell samples were detected by RNA-seq sequencing (Figure 3A–D, Table S1, and Supplementary File 2). The quality control test results showed that our RNA-seq was qualified (Supplementary File S2 Figure S1A,B). A total of 410 DEGs were obtained, of which 371 genes were up-regulated, and 39 genes were down-regulated (*p*-value < 0.05 and |log2(fc)| > 1) (Figure 3C, Supplementary Files 1 and 3). A heatmap and volcano map displayed the distribution of genes in each group (Figure 3B,D). As demonstrated in Table 1, Table 2 and Table 3, PrRP stimulated (371 up-regulated genes) more genes than suppressing genes (39 down-regulated genes) on the transcriptional level of BMDMs. In addition, DEGs stimulated by PrRP were composed of the following types of genes: protein_coding genes (94.43%), lincRNA genes (1.03%), antisense genes (0.66%), and other genes (3.88%) (Figure 3A).

### 2.3. Functional and Pathway Enrichment Analysis of DEGs

To explore the biological meanings of DEGs, several tools were used to analyze the functions of DEGs, such as Metascape, KEGG, and PANTHER.

The KEGG online tool was used to explore the enrichment pathways of DEGs. As shown in Table S2, DEGs were involved in several biological pathways: autoimmune thyroid disease (mmu05320; gene count: 110), Epstein–Barr virus infection (mmu05169; gene count: 24), proteasome (mmu03050; gene count: 127), and allograft rejection (mmu05330; gene count: 46) (Table S2).

**Figure 4:**
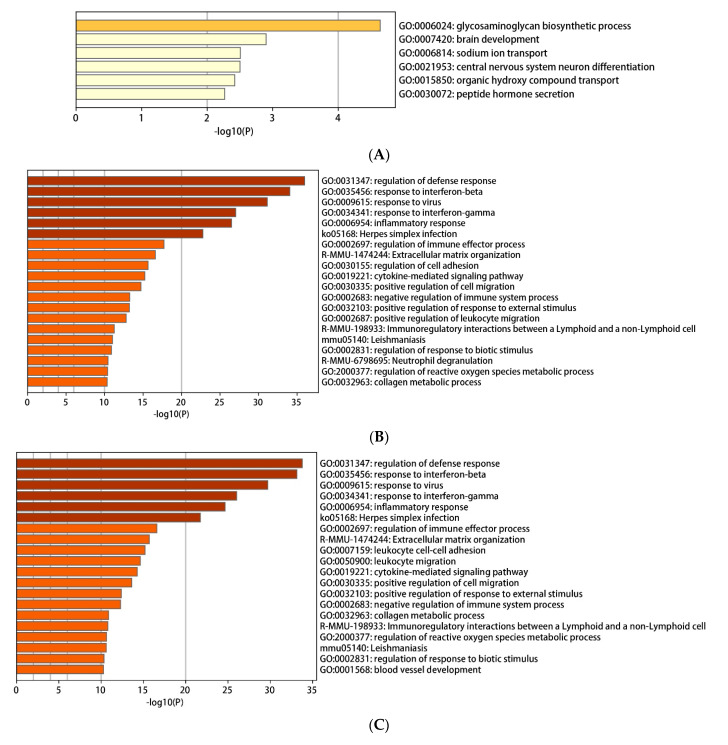

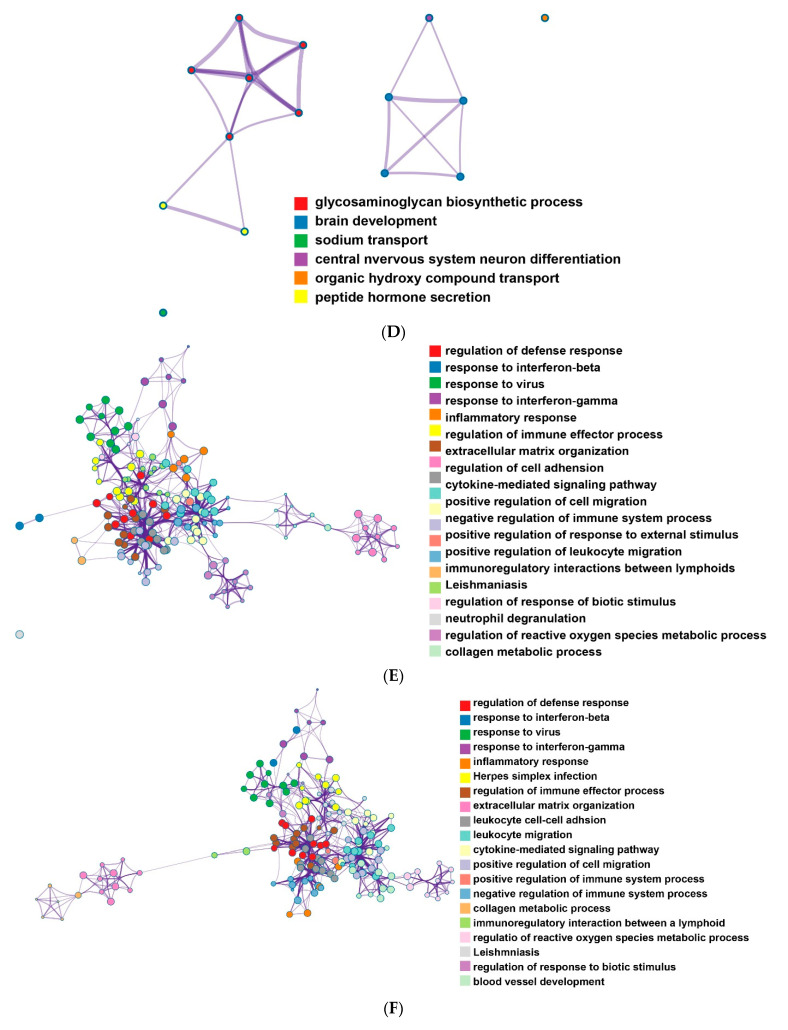

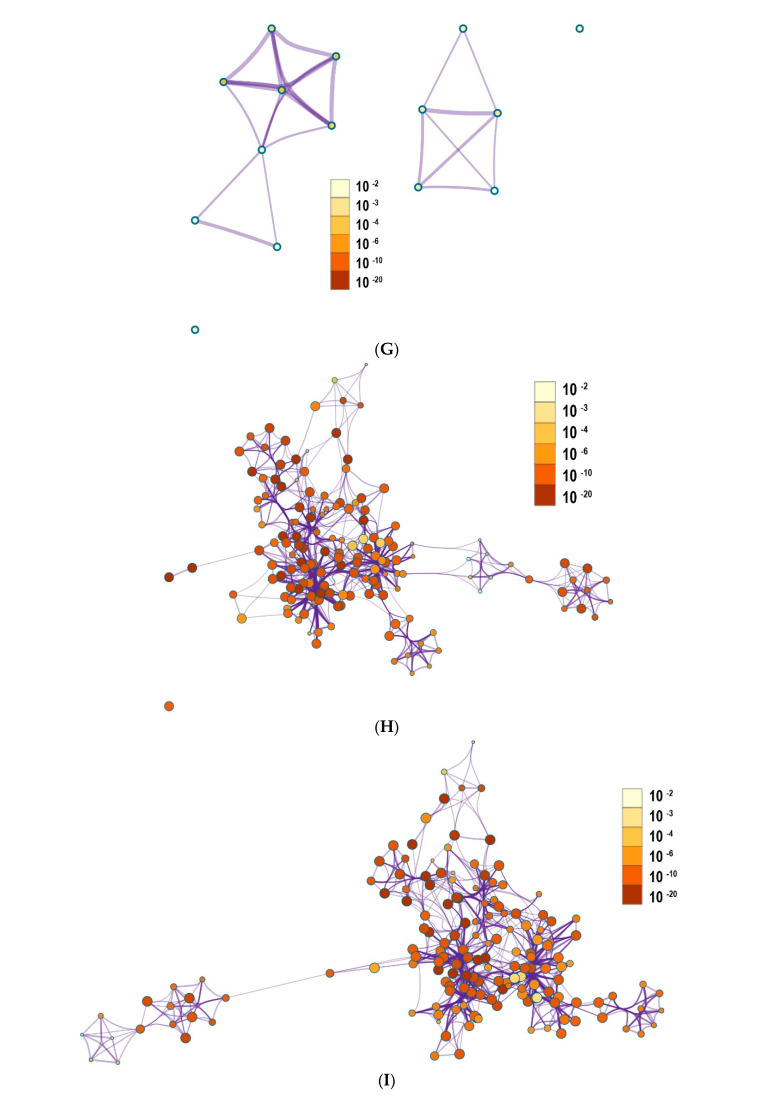
The enrichment analysis results by using Metascape. (**A**–**C**) Bar plots of enriched terms for down-regulated DEGs (**A**), up-regulated DEGs (**B**), and all DEGs (**C**) (colored by p-values) were presented. (**D**–**F**) Network of enriched terms (colored by cluster ID), where nodes that shared the same cluster ID were typically close to each other. The networks for down-regulated DEGs (**D**), up-regulated DEGs (**E**), and all DEGs (**F**) were shown. (**G**–**I**) Network of enriched terms (colored by *p*-value), where terms containing more genes tended to have a more significant p-value. Network plots for down-regulated DEGs (**G**), up-regulated DEGs (**H**), and all DEGs (**I**) were demonstrated.

The Metascape online website was employed to study the functional enrichment of DEGs. All DEGs were subjected to Metascape to acquire the enrichment pathways. The down-regulated DEGs activated several pathways such as the glycosaminoglycan biosynthetic process, brain development, sodium ion transport, and central nervous system neuron differentiation (Figure 4A,D,G). Meanwhile, enriched pathways stimulated by up-regulated DEGs mainly included the regulation of defense response, response to interferon-beta, response to virus, response to interferon-gamma, inflammatory response, Herpes simplex infection (Figure 4B,E,H). Besides, all DEGs activated a series of pathways, including regulation of defense response, response to interferon-beta, response to virus, response to interferon-gamma, inflammatory response, and Herpes simplex infection (Figure 4C,F,I). Finally, these pathways were clustered, followed by connecting with various network diagrams (Figure 4G–I).

The online tool PANTHER was also employed to investigate the enriched processes of DEGs. All DEGs were classified into the following categories: molecular function (MF), biological process (BP), and cellular compartment (CC).

**Figure 5:**
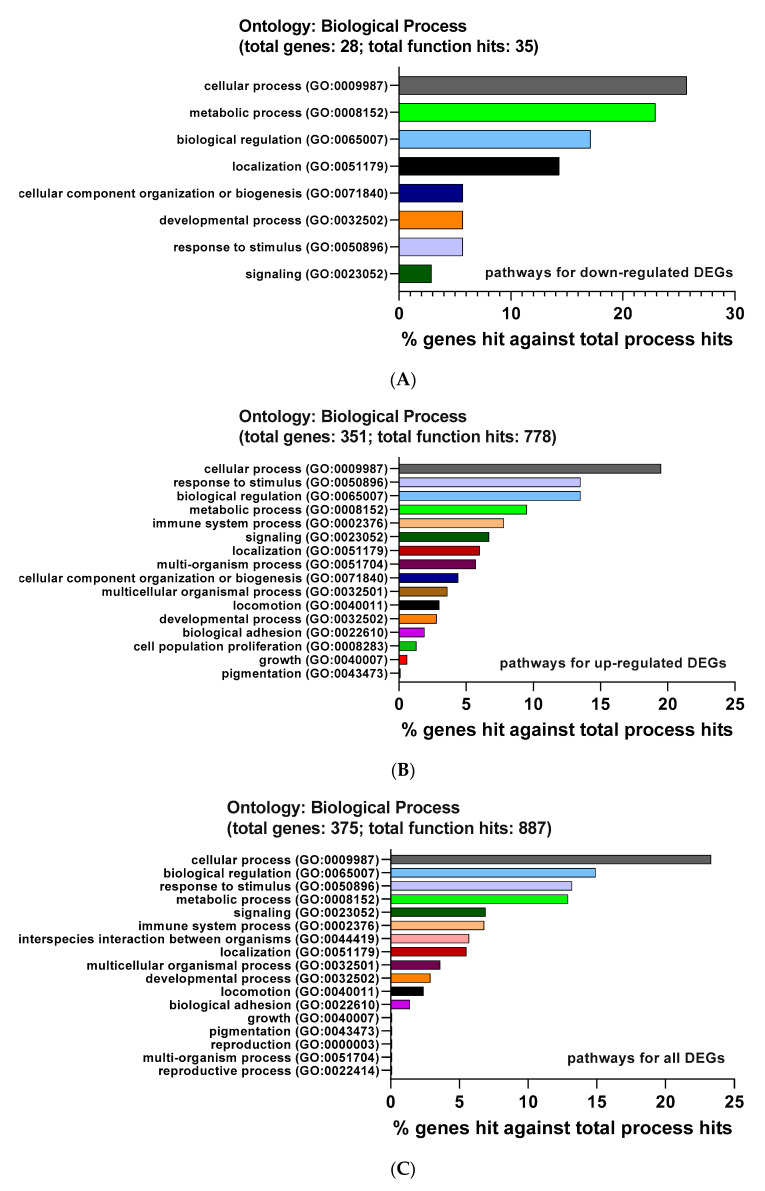

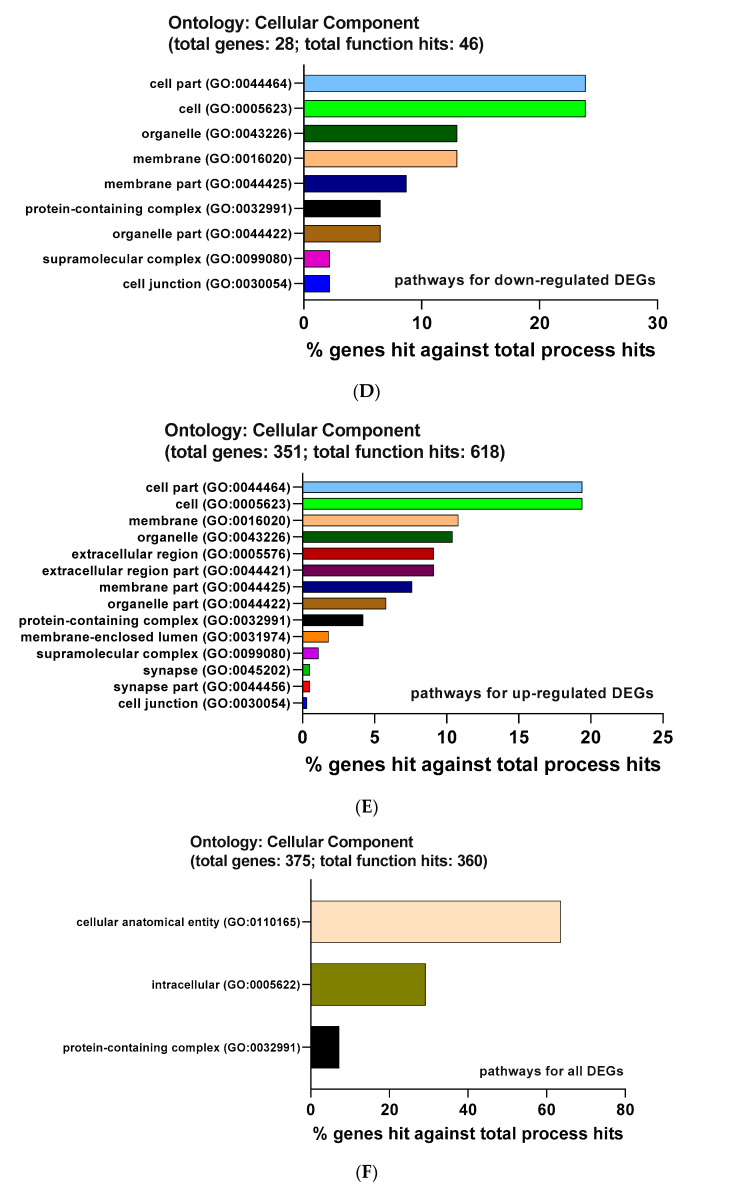

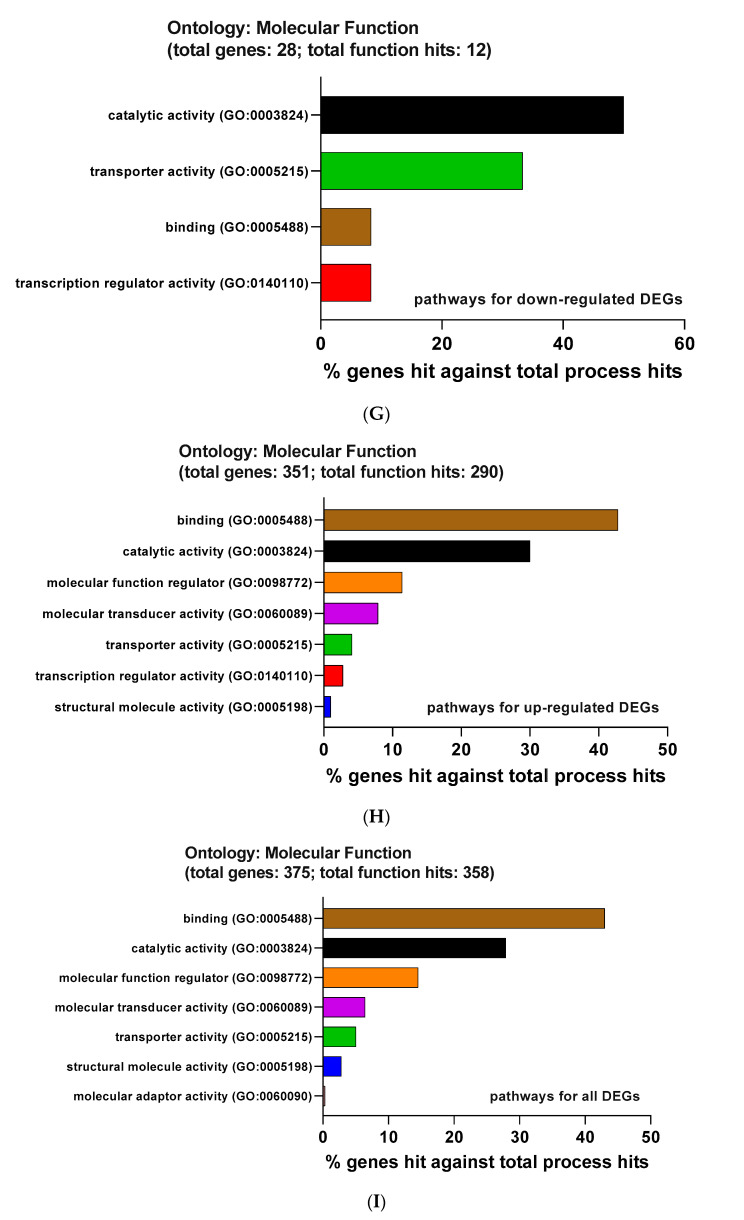
Functional enrichment analysis data of DEGs from PANTHER. The DEGs were analyzed with the PANTHER GO classification. (**A**–**C**) Biological process (BP) of down-regulated DEGs (**A**), up-regulated DEGs (**B**), and all DEGs (**C**). (**D**–**F**) Cellular component (CC) of down-regulated DEGs (**D**), up-regulated DEGs (**E**), and all DEGs (**F**). (**G**–**I**) Molecular function (MF) of down-regulated DEGs (**G**), up-regulated DEGs (**H**), and all DEGs (**I**).

For the biological processes, the biological processes caused by down-regulated DEGs were: cellular process (GO:0009987, 25.7%), metabolic process (GO:0008152, 22.9%), and biological regulation (GO:0065007, 17.1%) (Figure 5A). Meanwhile, up-DEG-caused biological processes were mainly composed of the following processes: cellular process (GO:0009987, 19.5%), response to a stimulus (GO:0050896, 13.5%), and biological regulation (GO:0065007, 13.5%) (Figure 5B). Moreover, all DEGs affected diverse pathways such as cellular process (GO:0009987), biological regulation (GO:0065007), response to stimulus (GO:0050896), and metabolic process (GO:0008152) (Figure 5C).

As for the cellular compartment pathway, down-regulated DEGs activated the following processes: cell part (GO:0044464, 23.9%), cell (GO:0005623, 23.9%), and organelle (GO:0043226, 13%) (Figure 5D). Meanwhile, the main pathways activated by up-regulated DEGs were cell part (GO:0044464, 19.4%), cell (GO:0005623, 19.4%), and membrane (GO:0016020, 10.8%) (Figure 5E). In addition, all DEGs were involved in the following pathways: cellular anatomical entity (GO:0110165), intracellular (GO:0005622), and protein-containing complex (GO:0032991) (Figure 5F).

As displayed in Figure 5E, down-regulated activated the following processes: catalytic activity (GO:0003824, 50%), transporter activity (GO:0005215, 33.3%), and binding (GO:0005488, 8.3%) (Figure 5G). Besides, up-regulated DEGs were involved in the following activities: binding (GO:0005488, 42.8%), catalytic activity (GO:0003824, 30%), and molecular function regulator (GO:0098772, 11.4%) (Figure 5H). Besides, all DEGs provoked a number of pathways, including binding (GO:0005488), catalytic activity (GO:0003824), and molecular function regulator (GO:0098772) (Figure 5I).

### 2.4. Protein–Protein Interaction (PPI) Network Analysis

To explore the DEGs-induced protein–protein interactions, DEGs were analyzed with the online tool STRING database, followed by visualizing with Cytoscape. By using the Cytoscape’s plug-in cyto-Hubba, a total of eight hub genes (*IFIT1, OASL2, IRF7, IFIT3, IFIT2, USP18, IFI44*, and *RTP4*) were acquired with the highest scores. Besides, the Cytoscape plug-in ClueGO was used to study DEGs-induced functional processes.

**Figure 6:**
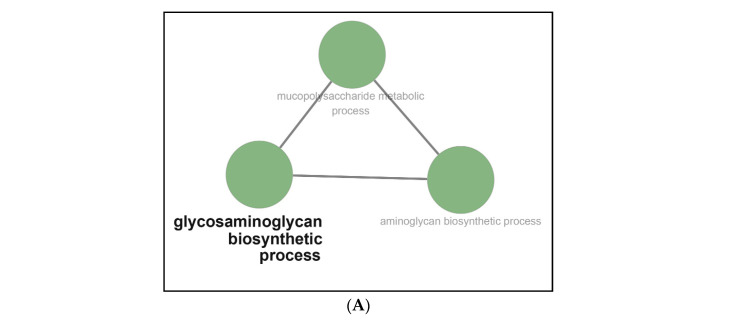

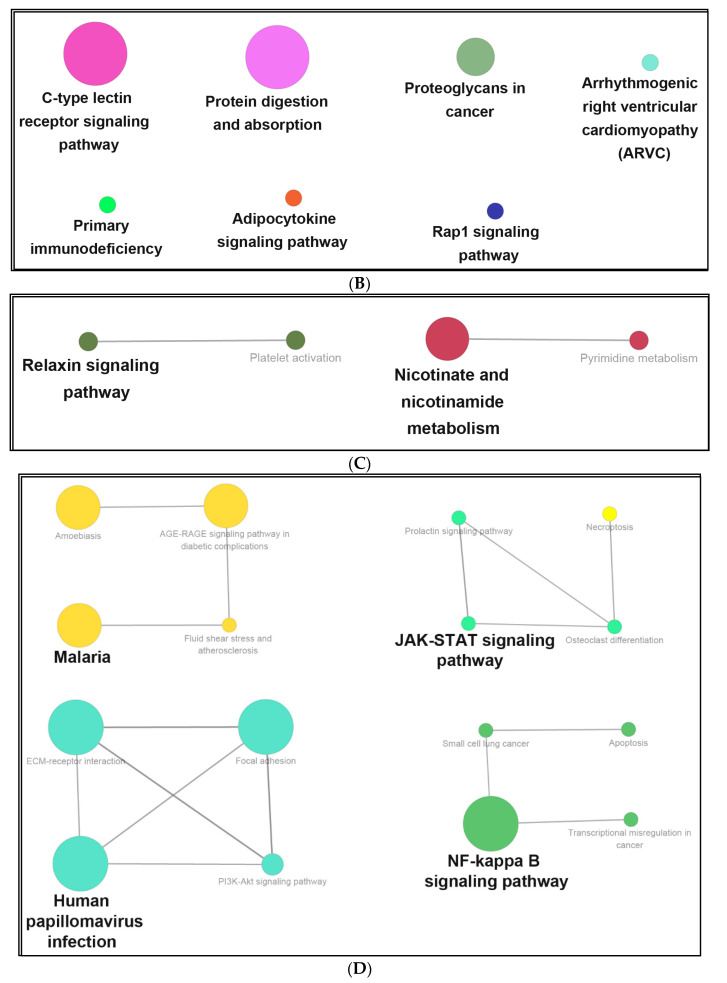

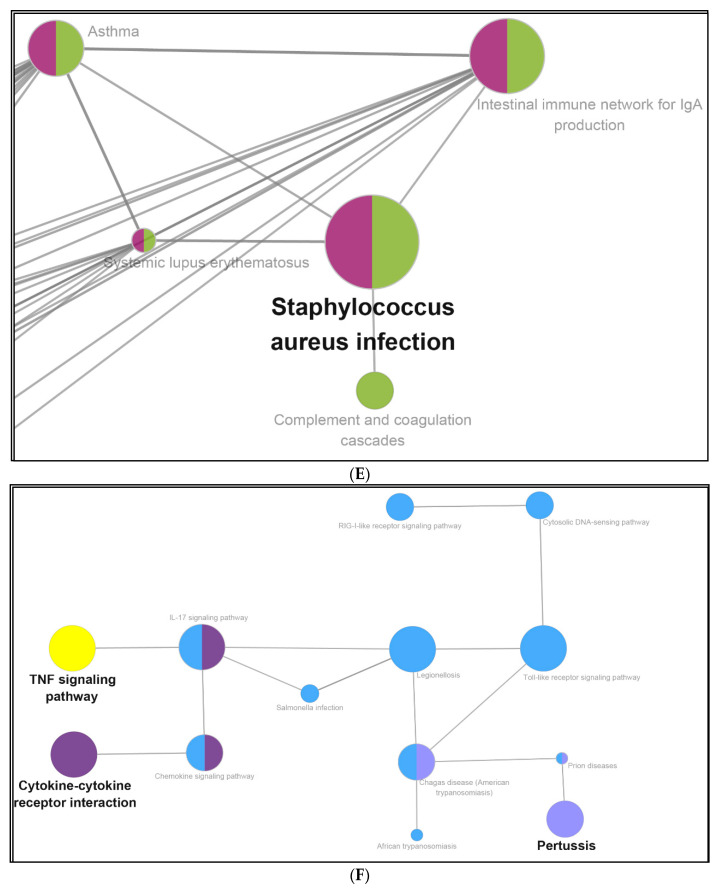

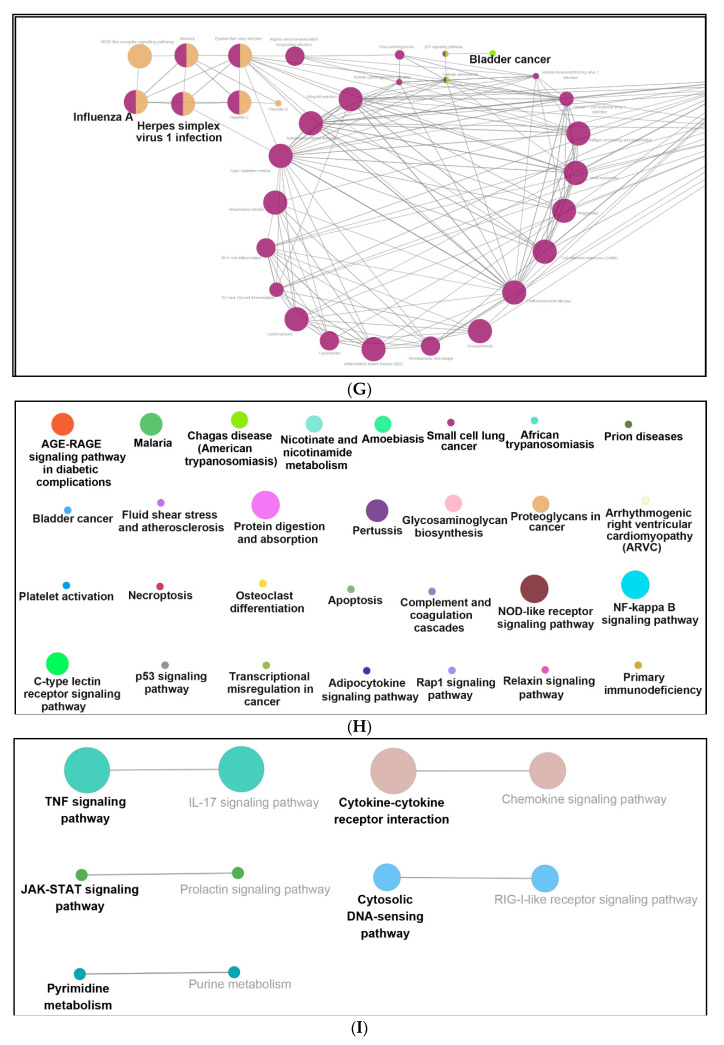

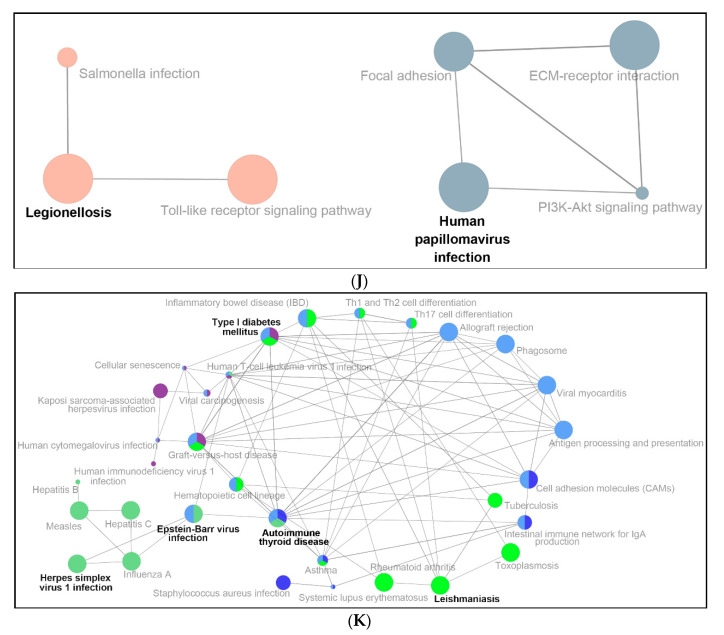
Functional enrichment analysis for DEGs using ClueGO. Functional enrichment for down-regulated DEGs showed that glycosaminoglycan biosynthetic process (**A**) was affected. Besides, the up-regulated DEGs activated various pathways, including (**B**) C-type lectin receptor signaling pathways, protein digestion and absorption, proteoglycans in cancer, arrhythmogenic right venticular cardiomyopathy (ARVC), primary immunodeficiency, adipocytokine signaling pathway, Rap1 signaling pathway; (**C**) relaxin signaling pathway and nicotinate and nicotinamide metabolism; (**D**) Malaria, JAK-STAT signaling pathway, human papillomavirus infection, NF-kappa B signaling pathway; (**E**) Staphylococcus aureus infection; (**F**) TNF signaling pathway, cytokine-cytokine receptor interaction, and pertussis; (**G**) Influenza A, Herpes simplex virus 1 infection, and bladder cancer. Moreover, a series of pathways were affected by all DEGs (include Up-regulated and down-regulated DEGs), including (**H**) AGE-RAGE signaling pathway in diabetic complications, malaria, Chagas disease (American trypanosomiasis), nicotinate and nicotinamide metabolism, amoebiasis, small cell lung cancer, African trypanosomiasis, prison diseases, bladder cancer, fluid shear stress and atherosclerosis, protein digestion and absorption, pertussis, glycosaminoglycan biosynthesis, proteoglycans in cancer, arrhythmogenic right ventricular cardiomyopathy (ARVC), platelet activation, necroptosis, osteoclast differentiation, apoptosis, complement and coagulation cascade, NOD-like receptor signaling pathway, NF-kappa B signaling pathway, C-type lection receptor signaling pathway, p53 signaling pathway, transcriptional misregulation in cancer, adipocytokine signaling pathway, Rap1 signaling pathway, relaxin signaling pathway, and primary immunodeficiency. (**I**) TNF signaling pathway, cytokine-cytokine receptor interaction, JAK-STAT signaling pathway, cytosolic DNA-sensing pathway, and pyrimidine metabolism. (**J**) Legionellosis and human papillomavirus infection. (**K**) Type I diabetes mellitus, Herpes simplex virus 1 infection, Epstein–Barr virus infection, autoimmune thyroid disease, and Leishmaniasis.

Functional enrichment pathways analysis showed that the down-regulated DEGs stimulated the glycosaminoglycan biosynthetic process (Figure 6A). Meanwhile, up-regulated DEGs were involved in the activation of the following pathways: C-type lectin receptor signaling pathways, protein digestion and absorption, proteoglycans in cancer, arrhythmogenic right ventricular cardiomyopathy (ARVC), primary immunodeficiency, adipocytokine signaling pathway, Rap1 signaling pathway (Figure 6B); relaxin signaling pathway and nicotinate and nicotinamide metabolism (Figure 6C); Malaria, JAK-STAT signaling pathway, human papillomavirus infection, NF-kappa B signaling pathway (Figure 6D); Staphylococcus aureus infection (Figure 6E); TNF signaling pathway, cytokine-cytokine receptor interaction, and pertussis (Figure 6F); Influenza A, Herpes simplex virus 1 infection, and bladder cancer (Figure 6G). Moreover, a series of pathways were affected by all DEGs (Up- and down-regulated DEGs), including AGE-RAGE signaling pathway in diabetic compli-cations, malaria, Chagas disease (American trpanosomiasis), nicotinate and nicotinamide metabolism, amoebiasis, small cell lung cancer, African trypnosomiasis, prison diseases, bladder cancer, fluid shear stress and atherosclerosis, protein digestion and absorption, pertussis, glycosaminoglycan biosynthesis, proteoglycans in cancer, arrhythmogenic right ventricular cardiomyopathy (ARVC), platelet activation, necroptosis, osteoclast differenti-ation, apoptosis, complement and coagulation cascade, NOD-like receptor signaling pathway, NF-kappa B signaling pathway, C-type lection receptor signaling pathway, p53 signaling pathway, transcriptional misregulation in cancer, adipocytokine signaling pathway, Rap1 signaling pathway, relaxin signaling pathway, and primary immunode-ficiency (Figure 6H); TNF signaling pathway, cytokine-cytokine receptor interaction, JAK-STAT signaling pathway, cytosolic DNA-sensing pathway, and pyrimidine metabolism (Figure 6I); Le-gionellosis and human papillomavirus infection (Figure 6J); Type I diabetes mellitus, Herpes simplex virus 1 infection, Epstein–Barr virus infection, autoimmune thyroid disease, and Leishmaniasis (Figure 6K).

**Figure 7:**
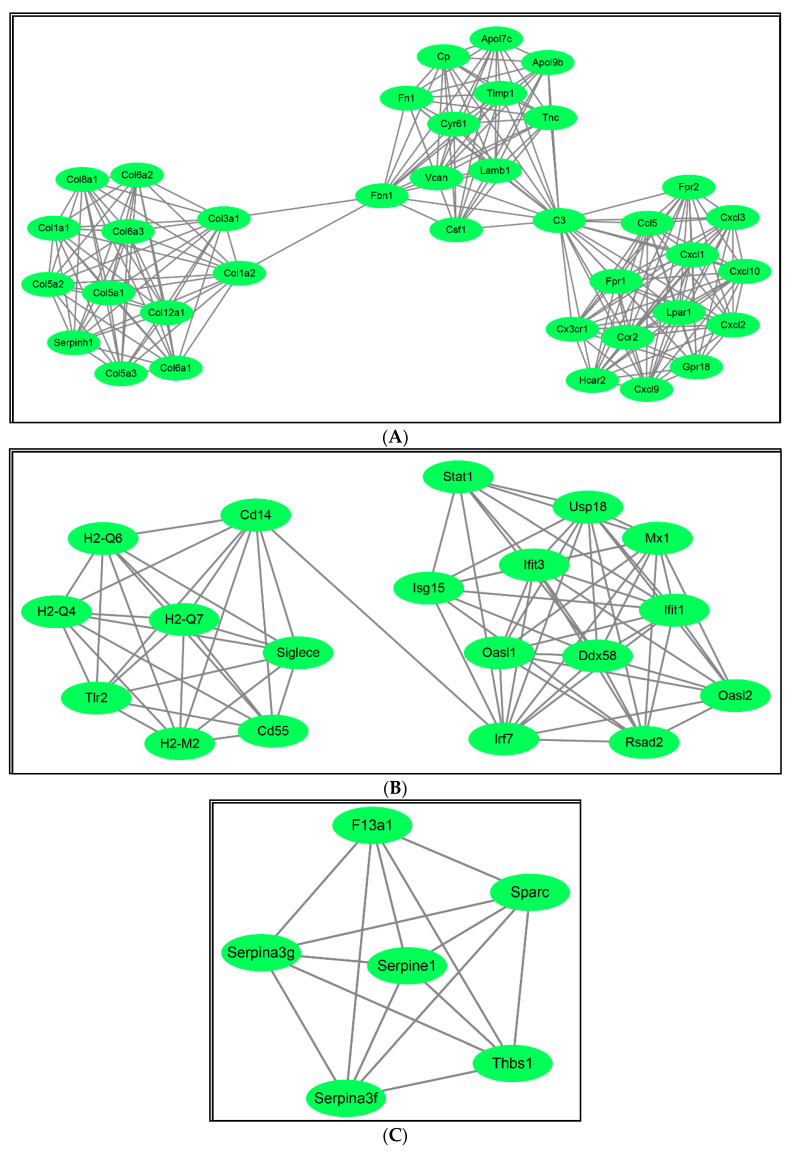

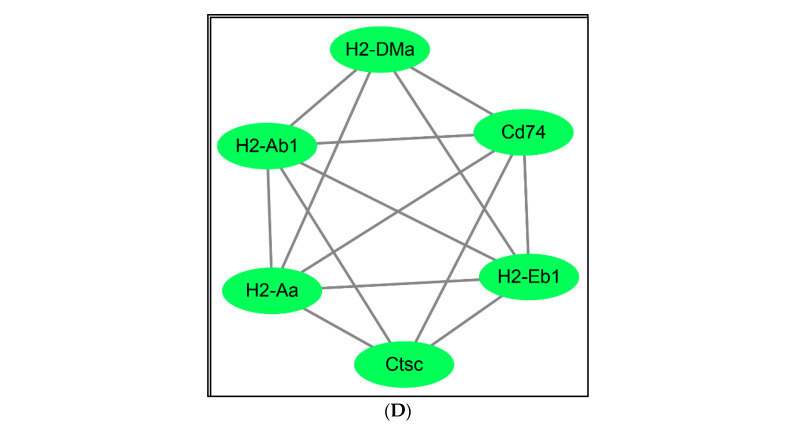
Protein-protein interaction (PPI) analysis of DEGs using the Cytoscape Plug-in Mcode. The PPI interaction between DEGs was analyzed with the Cytoscape Plug-in Mcode. (**A**–**D**) Four networks were activated by the up-regulated DEGs, and no network was stimulated by the down-regulated DEGs.

In addition, the GO analysis and detailed information of eight hub genes were listed in Table 4 and Table 5. Besides, the PPI interaction between DEGs was analyzed with the Cytoscape Plug-in Mcode. As demonstrated in Figure 7, four networks were activated by the up-regulated DEGs while no network was stimulated by the down-regulated DEGs.

### 2.5. Common Transcription Factors Tied to Genes Down-Regulated by PrRP

To investigate the transcription factors of genes up-regulated by PrRP, TRRUST (version 2) was utilized. As demonstrated in Table 6, sixteen transcription factors were collected (screening criterion: *p* < 0.05), which included Irf1, Stat1, Nfkb1, Jun, Irf8, Rel, Rela, Foxo3, Irf4, Ikbkb, Foxm1, Hdac1, Spi1, Cebpb, Fos, and Stat3. In addition, no transcription factors were obtained for the down-regulated DEGs.

### 2.6. Verification of Hub Genes with qPCR and Western Blot

**Figure 8:**
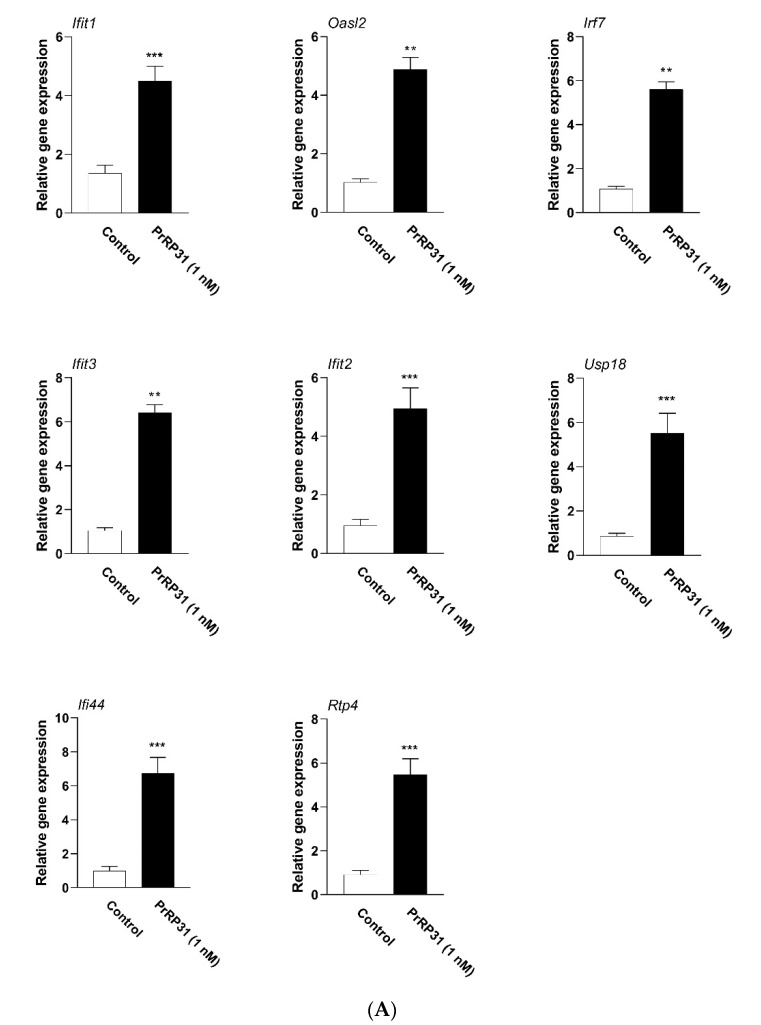

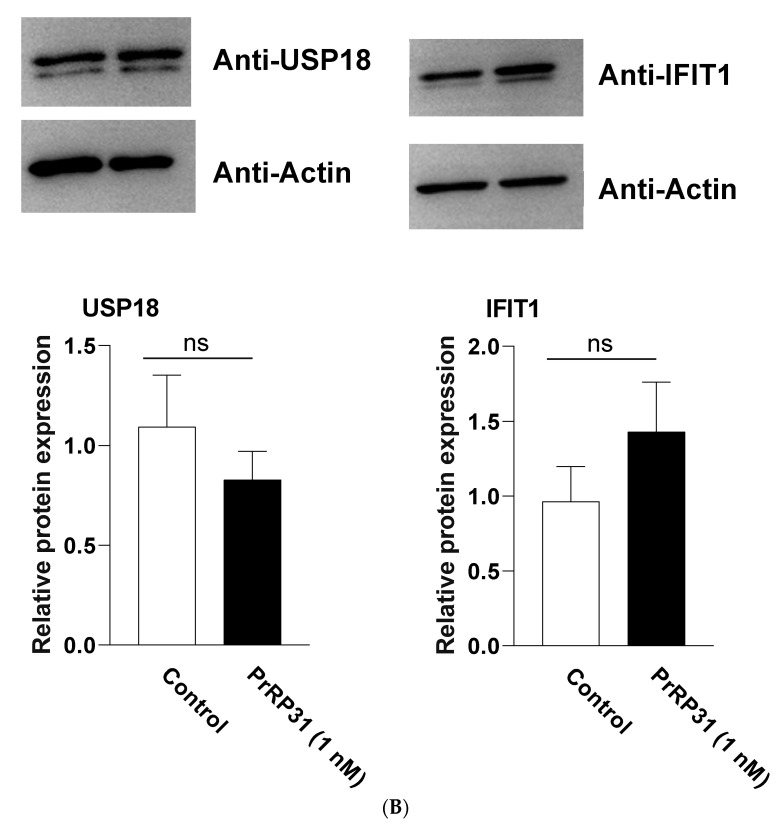
Verification of hub genes. BMDMs were incubated with PrRP (1nM) for 18 h, followed by a qPCR detection and a Western blot. (**A**) Total RNA was extracted, and a qPCR detection was carried out to identify eight hub genes. The mRNA level was normalized by the expression of GAPDH. *, significantly different from the control group, ** *p* < 0.01; *** *p* < 0.001. Each detection was conducted three times in duplicate. The data were presented as the means ± S.E.M. Statistical significance analysis was performed with the t-test method. (**B**) BMDMs were lysed and subsequently subjected to Western blot examination (n = 3). The data are shown as the means ± S.E.M. *, significantly different from the control group; ns, no significance. Statistical significance analysis was conducted with the t-test approach.

In order to verify the accuracy of the RNA-seq data, qPCR was carried out. These hub genes were all protein-coding genes (*IFIT1, OASL2, IRF7, IFIT3, IFIT2, USP18, IFI44,* and *RTP4*). As displayed in Figure 8A, PrRP (1 nM) up-regulated the mRNAs of eight hub genes and no hub genes were down-regulated, which were consistent with the RNA-seq results. Moreover, the expression of the hub protein was also detected by the Western blot. Since only a few commercial antibodies were available for us, the protein expression data of two hub proteins (IFIT1 and USP18) were acquired. As demonstrated in Figure 8B, PrRP showed no noticeable effect on the protein expression of IFIT1 and USP18.

### 2.7. Protein Modeling of Hub Proteins

**Figure 9:**
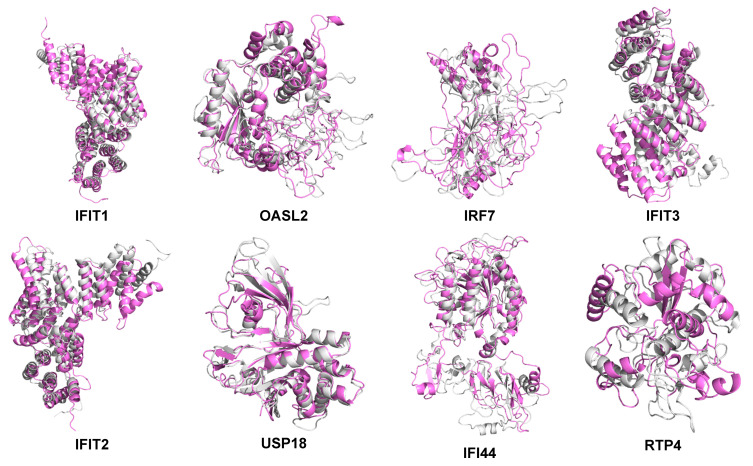
Superposition of the 3-D models of primarily modeled structure (gray) and MD-optimized protein structure (violet) (IFIT1, OASL2, IRF7, IFIT3, IFIT2, USP18, IFI44, and RTP4). The figure was produced with the Pymol software (Delano, W.L. The Pymol Molecular Graphics System (2002) DeLano Scientific, SanCarlos, CA, USA. http://www.pymol.org, date of retrieving data: 11 November 2020).

To investigate the protein structure of hub proteins, the three-dimensional structures of hub proteins were built using the Modeller (9v23). A total of 1000 models for hub proteins were acquired (except for IFI44 and RTP4), and the model with the lowest DOPE value was collected (IFIT1: −54845.33203; OASL2: −49878.21484; IRF7: −32177.46484; IFIT3: −43405.51953; IFIT2: −53080.90625; USP18: −41661.42188) (Figure 9, Table 7).

**Figure 10:**
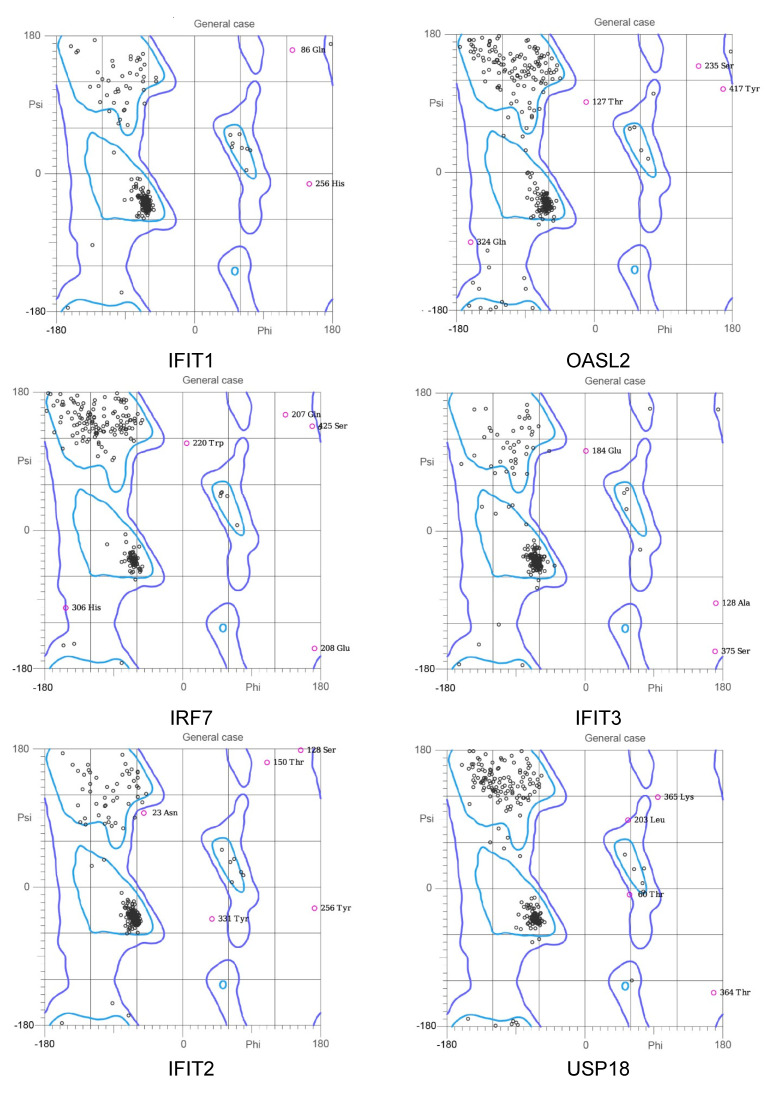

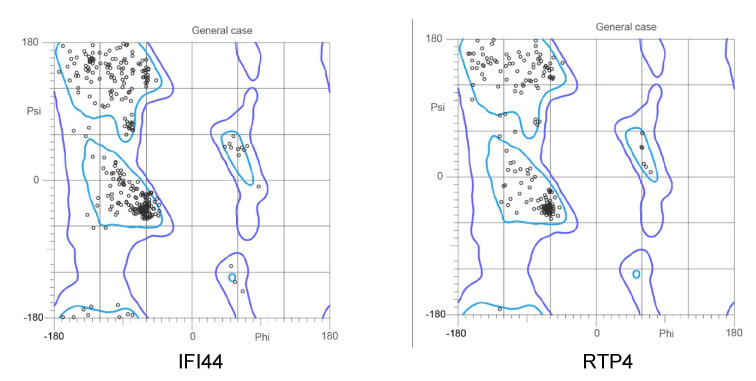
Ramachandran analysis of the homology modeled-models of hub proteins (IFIT1, OASL2, IRF7, IFIT3, IFIT2, USP18, IFI44, and RTP4). The different colored areas: ‘disallowed’ (violet), ‘most favored’ (light blue), and ‘generously allowed’ (blue).

Subsequently, these protein models were sent to the online MolProbity website to evaluate the quality of these models. As displayed in Figure 10 and Table 8, the ratio of residues located in the outlier region of proteins was ranged from 0.01% to 0.04% (IFIT1: 0.004%; OASL2: 0.014%; IRF7: 0.042%; IFIT3: 0.013%; IFIT2: 0.019%; USP18: 0.013%; IFI44: 0.000%; and RTP4: 0.000%), implying that the quality of these models was acceptable.

### 2.8. Molecular Dynamics Simulation of Hub Proteins

**Figure 11:**
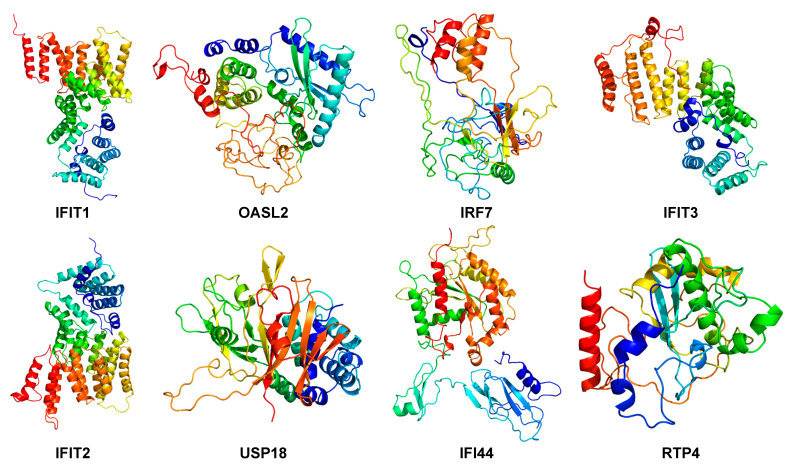
The three-dimensional structure of hub proteins. Three-dimensional models of MD-optimized hub proteins were shown, including IFIT1, OASL2, IRF7, IFIT3, IFIT2, USP18, IFI44, and RTP4. The picture was generated with the Pymol software (Delano, W.L. The Pymol Molecular Graphics System (2002) DeLano Scientific, SanCarlos, CA, USA. http://www.pymol.org, date of retrieving data: 11 November 2020).

**Figure 12:**
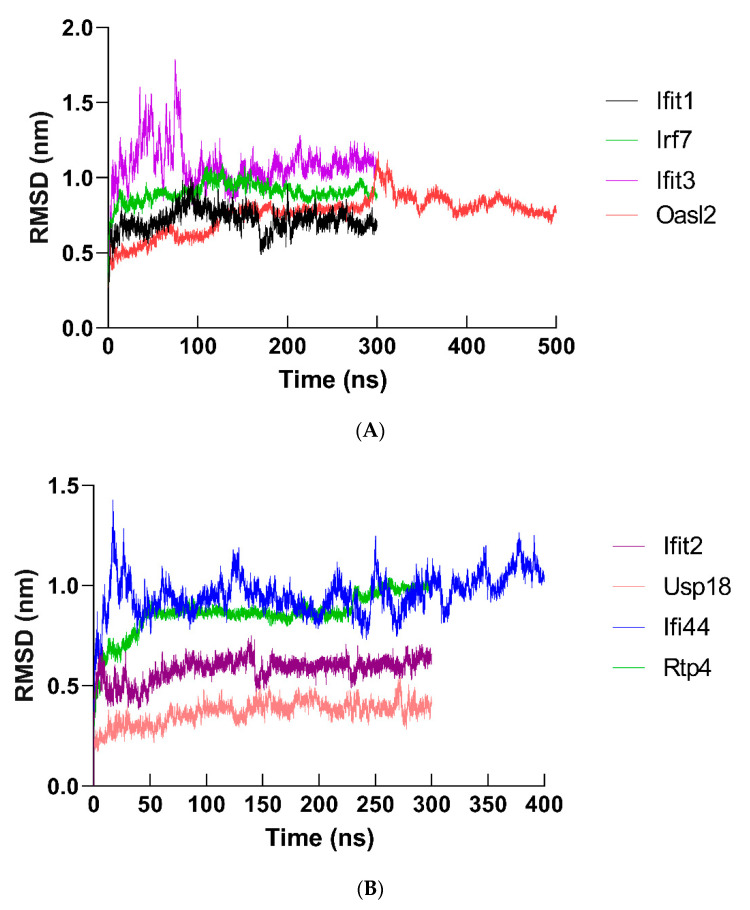
RMSD plots of hub proteins in molecular dynamics simulation (at least 300 ns) (backbone Cα atoms). To show the deviations of DEGs protein clearly, RMSD plots were presented. The RMSD of (**A**) IFIT1, OASL2, IRF7, IFIT3 and (**B**) IFIT2, USP18, IFI44, and RTP4 were shown.

**Figure 13:**
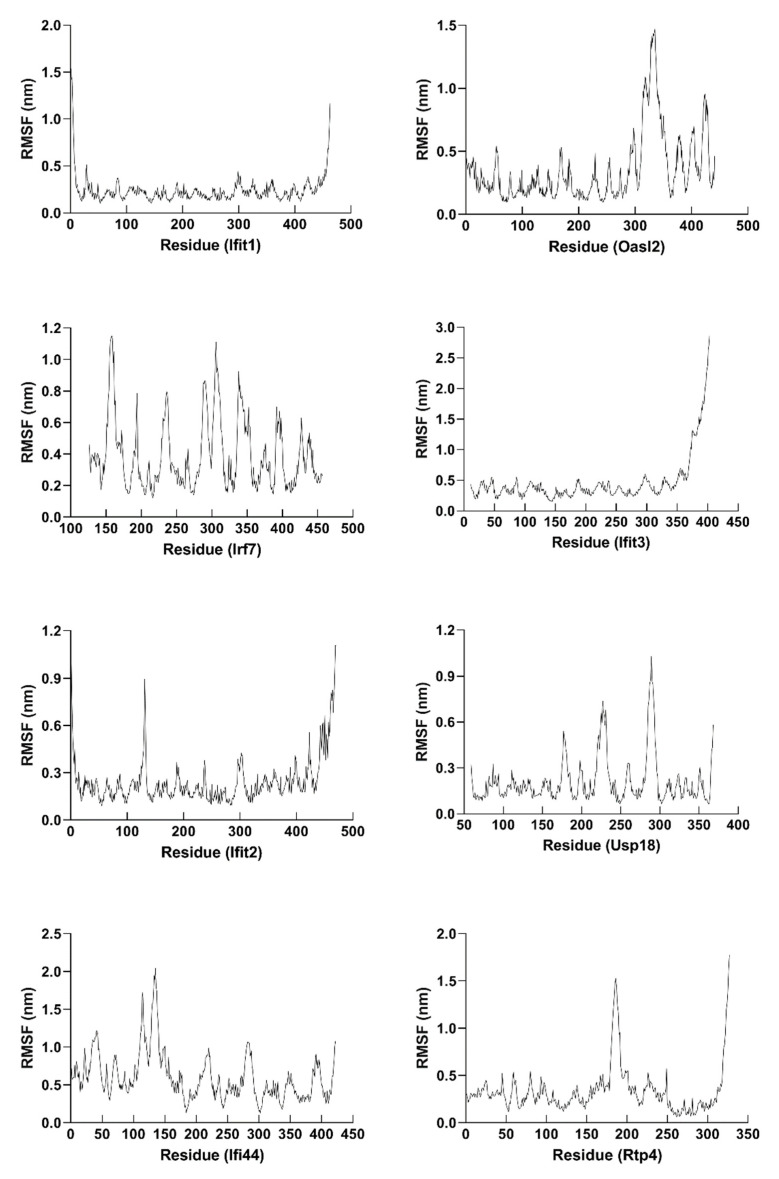
RMSF plots of hub proteins in molecular dynamics simulation (at least 300 ns). The RMSF of IFIT1, OASL2, IRF7, IFIT3, IFIT2, USP18, IFI44, and RTP4 were shown.

**Figure 14:**
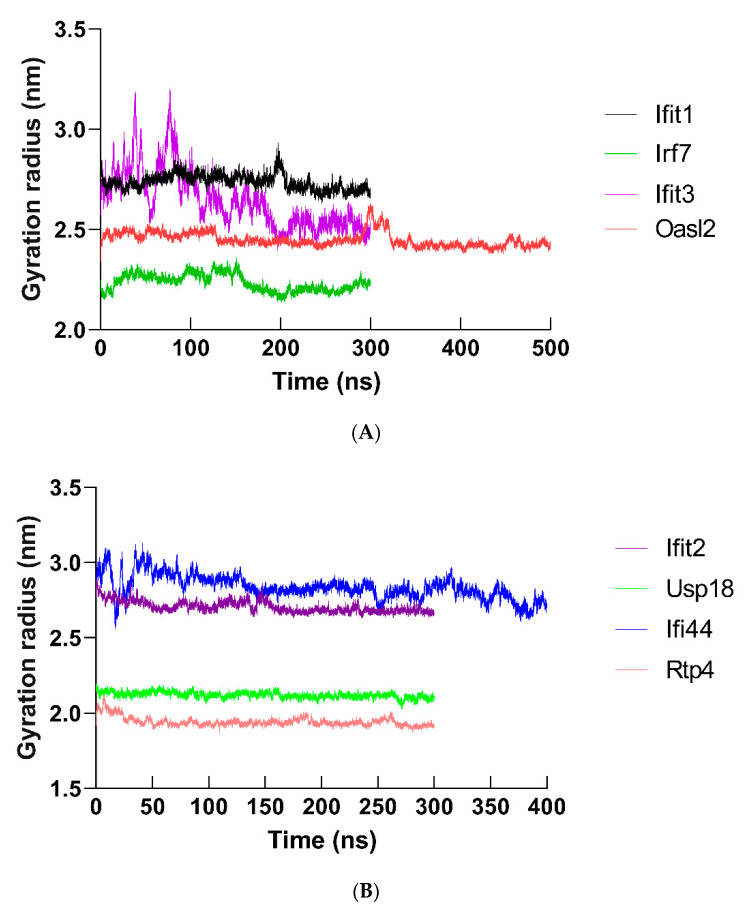
Gyration radius plots of hub proteins. The gyration radius of (**A**) IFIT1, OASL2, IRF7, IFIT3 and (**B**) IFIT2, USP18, IFI44, and RTP4 were shown.

Molecular dynamics (MD) simulation (at least 300 ns) was performed to study the behavior of the hub proteins (Figure 9 and Figure 11). The structural convergence data of hub proteins were displayed as RMSD (Figure 12), RMSF (Figure 13), and gyrate (Figure 14, Supplementary File 4: Table S3).

To investigate the flexibility of hub proteins, the RMSF of the atoms of hub proteins was analyzed (Figure 13). Residues with high RMSF suggested high flexibility, whereas low RMSF values indicated few fluctuations between average positions and residues. As displayed in Table S3 and Figure 13, the average RMSF score varied from 0.2118 and 0.4669 (IFIT1: 0.2412; OASL2: 0.3507; IRF7: 0.3929; IFIT3: 0.4669; IFIT2: 0.2438; USP18: 0.2118; IFI44: 0.6008; and RTP4: 0.3364).

To examine the dynamics and structures of hub proteins, the RMSD of hub protein atoms was interpreted. The backbone atom reached equilibrium within 2.21–4.68 ns from the initial stage (IFIT1: 2.61 ns; OASL2: 2.55 ns; IRF7: 2.22 ns; IFIT3: 2.44 ns; IFIT2: 4.68 ns; USP18: 4.02 ns; IFI44: 2.21 ns; and RTP4: 2.93 ns) (Figure 12). Subsequently, the structure started to converge at different time points (IFIT1: 3.2 ns; OASL2: 3.62 ns; IRF7: 2.85 ns; IFIT3: 5.12 ns; IFIT2: 4.95 ns; USP18: 4.67 ns; IFI44: 2.62 ns; and RTP4: 3.58 ns). The structure of the hub proteins maintained a stable conformation till the end of simulation (IFIT1: 0.66–0.81; OASL2: 0.60–0.82; IRF7: 0.97–1.09; IFIT3: 0.93–1.78; IFIT2: 0.54–0.75; USP18: 0.31–0.22; IFI44: 0.92–1.42; and RTP4: 0.89–0.1.05).

The radius of gyration (Rg) suggested the compression of the protein atoms, and a decrease in Rg indicated an unstable protein structure. As displayed in Figure 14, the average Rg score of hub protein varies from 1.943 to 2.836 (IFIT1: 2.738; OASL2: 2.449; IRF7: 2.235; IFIT3: 2.643; IFIT2: 2.705; USP18: 2.119; IFI44: 2.836; and RTP4: 1.943).

### 2.9. Peptide-Hub Protein Docking

**Figure 15:**
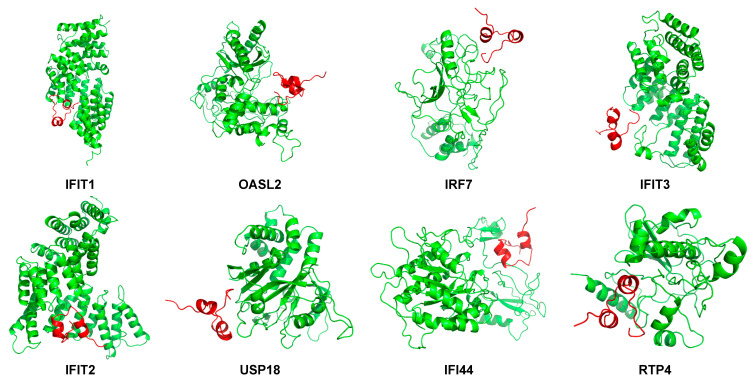
The docking analysis of PrRP31 and hub proteins. There 3-D structures of PrRP31-hub proteins were demonstrated, including IFIT1, OASL2, IRF7, IFIT3, IFIT2, USP18, IFI44, and RTP4. The picture was drawn with the Pymol software (Delano, W.L. The Pymol Molecular Graphics System (2002) DeLano Scientific, SanCarlos, CA, USA. http://www.pymol.org, date of retrieving data: 9 January 2021).

To explore the possible interaction between PrRP and hub proteins, the Rosetta program was employed to analyze the possible dock binding sites for PrRP-hub proteins. As displayed in Figure 15, PrRP was not wholly embedded in the protein structure. As expected, PrRP bound to the N- or C-terminal region of the hub protein, where located at the outside of the protein structure.

### 2.10. Expression of GPR10 on BMDMs

**Figure 16:**
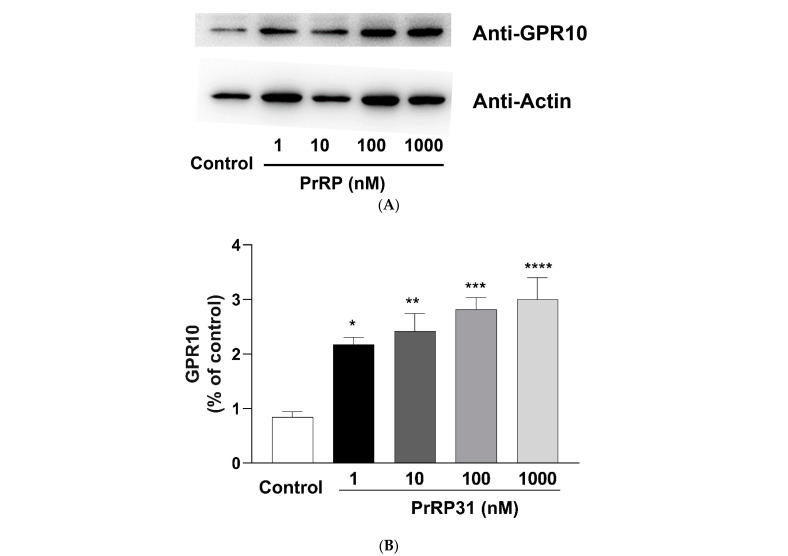

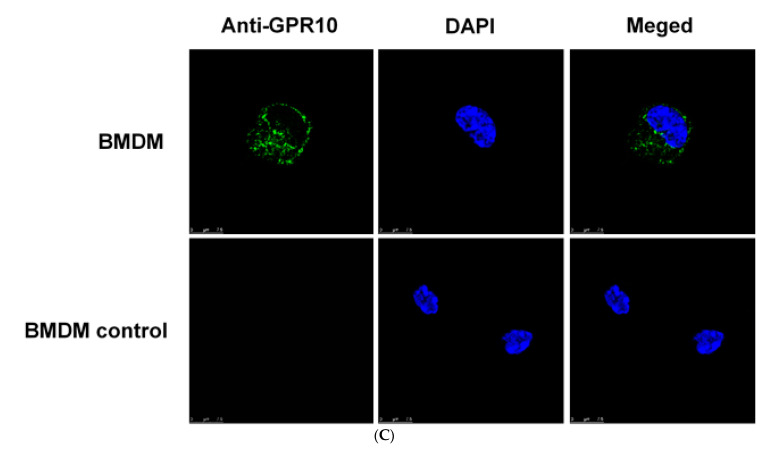
The expression of GPR10 protein in BMDMs was investigated by Western blot and immunofluorescence staining. (**A**) BMDMs were incubated with PrRP31 (1 nM) for 18 h, followed by immunoblot detection for GPR10 protein level (n = 5). (**B**) quantification of the bands in (A). The data were displayed as the means ± S.E.M. *, significantly different from the control group; * *p* < 0.05; ** *p* < 0.01; *** *p* < 0.001; **** *p* < 0.0001. Statistical significance analysis was conducted using the one-way ANOVA followed by the Tukey post hoc tests approach. (**C**) The expression of GPR10 in BMDMs was examined by immunofluorescence staining. The nucleus stained with DAPI was blue, and GPR10 stained with anti-GPR10 antibody was green. Scale bar, 75 μm.

The expression of GPR10 was examined in BMDMs by immunofluorescence stain and Western blot (Figure 16). As shown in Figure 16C, GPR10 protein was distributed on the cell membrane of BMDMs, and no signal was acquired with the IgG control (the negative control). PrRP 1 nM exposure (18 h) provoked a remarkable increase in the GPR10 protein (Figure 16A,B) than the control group.

## 3. Discussion

### 3.1. PrRP Modulated Different Functional Enrichment Pathways of BMDMs

In 2012, the Romeroa team discoveries that PrRP promotes the production of pro-inflammatory cytokines (IL-8, IL-12, IL-1β, and IL-6) in *Salmon salar*, indicating that PrRP is a local transmitter of the innate immune pathway in leukocytes [13]. In the same line, in this study, PrRP significantly promoted inflammatory immune response pathways, including regulation of defense response, response to interferon-beta, response to interferon-gamma, inflammatory response (Figure 4B,E,H). Overall, these data suggested that PrRP may be involved in inflammation and immune regulation processes.

A series of reports show that PrRP plays a vital role in regulating food intake and energy metabolism [8,26,27,28,29,30]. In the present study, PrRP regulated several functional pathways responsible for macrophages, including neuron differentiation of the central nervous system, sodium ion transport, brain development, and peptide hormone secretion (Figure 4), suggesting that the expression profile of related genes was affected by PrRP. Given that macrophages are deeply involved in regulating multiple physiological processes of neuroendocrine [31], PrRP may affect the physiological activities of the neuroendocrine field by regulating macrophages.

### 3.2. Common Transcription Factors Tied to PrRP-Regulated DEGs in BMDMs

In order to capture the effects of DEGs on the transcription profile of macrophages at the transcriptome level, DEGs were subjected to TRRUST (version 2) for transcription factor analysis. As demonstrated in Table 6, PrRP stimulated a series of general transcription factors, including *Irf1* (interferon regulatory factor 1), *Irf8* (interferon regulatory factor 8), and *Irf4* (interferon regulatory factor 4). These data implied that PrRP might stimulate the related genes governed by these transcription factors. Besides, PrRP activated several differentiation-related transcription factors, including *Stat1* (signal transducer and activator of transcription 1), *Nfkb1* (nuclear factor of kappa light polypeptide gene enhancer in B cells 1, p105), *Jun* (jun proto-oncogene), *Ikbkb* (inhibitor of kappaB kinase beta), and *Stat3* (signal transducer and activator of transcription 3), indicating that PrRP might be involved in the biological processes mastered by above transcription factors. Additionally, the number of down-regulated DEGs is too small to obtain transcription factors.

### 3.3. The Concentration of PrRP in the Experimental System for High-Throughput Sequencing

In this study, the treatment concentration of PrRP on cells is a question worth of concern. To the best of our knowledge, the use of high-throughput sequencing (such as RNA-seq) to investigate the effects of PrRP on the transcriptome of target tissues or cells has not been reported yet. In our experimental system, PrRP 1 nM was selected as the concentration to treat cells based on the following considerations.

On the one hand, the concentration of PrRP was limited to a range as close as possible to the physiological concentration in the body (around nM range), which would be helpful to simulate the real effect of neuropeptides in physiological conditions as much as possible. On the other hand, NPFF, another neuropeptide belonging to the RFamide peptide family as PrRP, provides us with a reference. Recently, Waqas and colleagues investigate the effects of NPFF on the gene expression of J774A.1 macrophages and 3T3-L1 preadipocytes by high throughput sequencing where cells were exposed to NPFF 1 nM for 18 h [15,32]. Moreover, our lab also examine the influence of NPFF on the mouse macrophages RAW 264.7 transcriptome where the same exposure (1 nM, 18 h) was applied to the cells [14]. Therefore, in this study, BMDMs were exposed to 1 nM PrRP for 18 h for subsequent RNA-seq detection.

### 3.4. Expression of PrRP-GPR10 on BMDMs

PrRP-GPR10 is widely expressed in the neuroendocrine system. In the peripheral tissues, as a ligand in the PrRP-GPR10 system, PrRP mRNA is found in the lung, adrenal gland, liver, kidney, pancreas, gut, and reproductive organs [33,34]. In addition, PrRP is also distributed in the NTS, ventrolateral reticular, and DMN nucleus [6]. Meanwhile, as the receptor in the PrRP-GPR10 system, GPR10 mRNA is expressed in the rat adrenal medulla, epididymis, and testis [35]. Given that the pons and hypothalamus are both key parts of controlling food intake and energy metabolism [36], the above data shows that PrRP-GPR10 should play a key role in neuroendocrine metabolism and may even be a potential target for anti-obesity therapy [29,37,38].

In the central nervous system, GPR10 is expressed in the paraventricular hypothalamic (PVN), thalamic reticular (TRN), dorsomedial hypothalamic (DMN) nuclei, periventricular hypothalamic (PEVN), and the nucleus of the solitary tract (NTS) of the brainstem [39]. These data suggest that GPR10 is involved in energy metabolism and food intake [8,40]. Very recently, the Lenka Maletínská team find that GPR10 gene deletion in C57BL/6J mice causes significant metabolic disturbances, as GPR10 KO mice demonstrate enhanced basal neuronal activity, disturbed lipid homeostasis, and altered insulin sensitivity [37].

Moreover, the expression of PrRP-GPR10 in the immune system has already been reported. The Romeroa group demonstrates that PrRP mRNA is expressed in leukocytes from the head kidney and blood of *Salmon salar*. Moreover, synthetic PrRP provokes the production of pro-inflammatory cytokines (IL-8, IL-12, IL-1β, and IL-6), implying that PrRP could be a local transmitter of the innate immune pathway in leukocytes [13]. In the present study, GPR10 was found to be expressed on the cell membrane of BMDMs (Figure 16C), which increased after PrRP (1–1000 nM, 18 h) exposure (Figure 16A,B). These data indicated that PrRP might be involved in diverse physiological processes controlled by macrophages.

### 3.5. Prolactin and Inflammatory Processes

To the best of our knowledge, there are few studies on the involvement of PrRP in the inflammatory process. However, prolactin, secreted by neurons stimulated by PrRP, is widely involved in inflammation and immune processes.

Prolactin is a peptide hormone that is generated in the anterior pituitary gland and in various sites outside of the pituitary. It has been reported that prolactin is involved in a variety of biological processes, including lactation, reproduction, and immune functions. Elevated serum prolactin concentration is often associated with inflammation and immune response [41].

The Jörg-Matthias Brand team finds that prolactin stimulates a pro-inflammatory immune response on peripheral inflammatory cells [42]. Prolactin, at concentrations achievable during medication, pregnancy, and anesthesia, significantly enhances the synthesis of tumor necrosis factor-alpha (TNF-alpha) and interleukin (IL)-12 in lipopolysaccharide-activated human whole blood cultures. These data suggest that prolactin may affect pathophysiological processes in physiological hyperprolactinemic disorders [42].

Actually, prolactin has been considered an inflammatory cytokine, which inhibits the negative selection of autoreactive B lymphocytes [41]. Up to now, hyperprolactinemia has been found in patients with a variety of autoimmune diseases, including systemic lupus erythematosus, rheumatoid arthritis, multiple sclerosis, systemic sclerosis, and autoimmune thyroid disease [41,43,44,45]. These data indicate that prolactin is involved in the pathological process of the above diseases.

### 3.6. Prolactin and Macrophages

Prolactin and its receptors are widely involved in the functional regulation of macrophages. On the one hand, prolactin promotes the release of cytokines, chemokines, and reactive oxygen species in macrophages [41,46,47,48,49,50]. On the other hand, the prolactin receptor is widely distributed through the immune system, such as macrophages, lymphocytes, monocytes, granulocytes, natural killer cells, and thymic epithelial cells [51]. Hence, prolactin and its receptor are widely involved in the immune response process.

In the present study, PrRP differentially regulated the gene expression of mouse BMDMs. Besides, GPR10, the receptor of PrRP, was expressed on mouse BMDMs. Given that BMDMs are deeply involved in regulating the neuroendocrine system, our data suggested that PrRP may be involved in various physiological processes controlled by macrophages.

### 3.7. PrRP and Microglia

Recently, the potential neuroprotective effect of PrRP has also been reported. By using a model of AD-like β-amyloid (Aβ) pathology (double transgenic APP/PS1 mice), Lenka Maletínskáa’s team find that a PrRP analog (palm^11^-PrRP31) reduces the total amount of senile Aβ plaques in APP/PS1 mice. Moreover, palm^11^-PrRP31 reduces the marker (ionized calcium-binding adaptor molecule 1 (Iba1)) of microglia near the Aβ plaque, suggesting that PrRP may have a potential neuroprotective effect [52].

Microglia are a type of cells derived from bone marrow hematopoietic stem cells. In the early stage of embryonic development, bone marrow hematopoietic stem cells enter the central nervous system and finally differentiate into microglia through the monocyte lineage [53]. As the resident macrophages in the central nervous system (CNS), microglia play an essential role in the innate and acquired immune response in local regions [54]. Given that microglia are mainly distributed in large non-overlapping areas throughout the CNS [55,56], and these areas may overlap with areas where PrRP is distributed (such as pons and hypothalamus) [33,34,35,40]. Therefore, a question arises: does PrRP regulate glial cells? Unfortunately, to the best of our knowledge, the research on the effect of PrRP on microglia has not been reported yet, which will be an exciting direction worth exploring.

### 3.8. Considerations of Double Positive Cells in the Control Group of Flow Cytometry Results

In the present study, mouse BMDMs were differentiated from primary mouse bone marrow cells induced by cytokines. It is worth noting that the results of the control group (no antibody was added) showed that 0.14% of the cells were still positive for anti-F4/80 and anti-CD11b (Figure S1A). Regarding this phenomenon, we believe that the following reasons may be responsible for this.

#### 3.8.1. Non-Specific Fluorescent Signal Caused by Dead Cells

Primary cells are of great significance in biomedical research because they have certain advantages over cell lines in maintaining the physiological environment of biological samples. However, a significant shortcoming in primary cell research is the difficulty in obtaining a single type of cell with 100% purity. In our experimental system, the monocytes in the bone marrow of mice were isolated according to a widely used method [57,58,59], followed by cytokine induction and eventually formed BMDMs. It should be pointed out that this method has a certain probability of introducing non-specific cells that initially existed in the bone marrow of mice. These cells may die in an unsuitable environment and eventually introduce a non-specific fluorescent signal in the flow cytometry test results. Moreover, the fluorescent signal of dead cells is often displayed as a diagonal signal on the scatter plot of a flow cytometer (it should be noted that the fluorescent signal on the diagonal of the scatter plot does not mean that these signals are certainly come from dead cells, as these signals may be a superposition of signals from live and dead cells) [60,61,62]. Furthermore, the intensity of non-specific fluorescence produced by dead cells can be similar to the intensity of fluorescence produced by fluorescein, which may introduce non-specific fluorescence signals into the final flow cytometer results [60,61,62]. In this study, the fluorescence signal of the control group (no antibody was added) was on the diagonal line (Figure 2B). Therefore, there is a possibility that the double-positive signal (only 0.14%) in the control cells may be part of the fluorescent signal introduced by dead cells.

#### 3.8.2. Non-Specific Fluorescent Signal from Cell Debris or Tiny Tissue Pieces

Cells or cell-like particulate matter is the object of flow cytometry analysis, and cell debris is inevitably present in the process of flow cytometry detection [62]. In this study, a series of measures were adopted to reduce non-specific signals detected by flow cytometry. Firstly, a widely used sterile filter (100 mesh) was used to obtain a single-cell suspension as much as possible during the experiment of obtaining mouse primary bone marrow cells. Secondly, in the flow cytometry detection step, a routinely used threshold was set to eliminate obvious cell debris and other non-target cells as much as possible.

Even so, our experimental system was still unable to absolutely distinguish the tiny amounts of other components that may be introduced during the isolation and culture of primary cells from BMDMs (especially those with tiny tissue masses similar in size to BMDMs). These non-specific substances might cause minor non-specific signals in the final results. In our experimental system, the double-positive rate of BMDMs in the experimental group was 96.7%, which was significantly higher than the control group (no antibody was added, double-positive rate: 0.14%), indicating that the purity of BMDMs was acceptable.

#### 3.8.3. The Excitation Wavelength of Fluorescein Is the Same as the Emission Wavelength, Which Is a Wavelength Range, Rather Than a Specific Value

In this study, PE-conjugated anti-F4/80 and FITC-conjugated anti-CD11b were used. In addition, the detection results of the flow cytometer have also undergone channel compensation processing as usual [63,64,65]. However, the excitation wavelength of fluorescein (the same as the emission wavelength) belongs to a wavelength range rather than a specific value [64]. Hence, it is difficult to obtain a completely 100% pure signal. In our opinion, the above factors may also contribute to the small amount of double-positive fluorescence signal in the control group of the flow cytometry test result (Figure 2B).

#### 3.8.4. Non-Specific Fluorescence Signals Caused by BMDMs

The intensity of the non-specific fluorescence of the cell is determined by the cell itself and is related to the size of the cell. Generally speaking, non-specific fluorescence produced by large cells is strong, while non-specific fluorescence produced by small cells is weak. Larger macrophages have stronger non-specific fluorescence than smaller lymphocytes [62,63,66]. In our study, flow cytometry was used to detect the purity of BMDMs, and the large volume of BMDMs might also introduce non-specific fluorescent signals for the control group (Figure 2B).

### 3.9. The Limitations of Our Study

Firstly, the changes in the protein levels of hub proteins are worth studying. In this study, through a series of bioinformatics methods, eight hub genes were obtained, which may be the critical nodes of PrRP regulating the gene network of BMDMs. Although the RNA-seq results of the hub genes were verified by qPCR, the changes in the protein levels of the eight hub genes would provide a solid biological basis for the significance of this study. However, limited by the fact that only a few commercial antibodies were currently available to us, the two hub proteins’ Western blot data (IFIT1 and USP18) were obtained. As shown in Figure 8B, PrRP did not significantly change the protein levels of IFIT1 and USP18 (Figure 8B). In our opinion, the following concerns would be helpful to understand this phenomenon.

(1) Hub genes responded to PrRP stimulation in protein levels might be late than in mRNA level. After PrRP (1 nM) treatment for 18 h, the mRNA levels of IFIT1 and USP18 were significantly increased. However, the influence of PrRP on the protein expression of hub genes might be longer than 18 h. In the process of gene expression (from mRNA to protein), it takes minutes to hours to translate mRNA into proteins [67].
(2) PrRP may regulate the expression of the hub proteins of BMDMs in a variety of ways. Although PrRP (1 nM) treatment for 18 h significantly affected the expression of hub genes, this does not mean that PrRP will cause changes in hub proteins’ expression levels. In the step from mRNA to protein, a variety of post-transcriptional modifications can cause changes in protein levels, including mRNA degradation control, mRNA translocation control, protein degradation control, translation control, mRNA translocation control, and protein degradation control [67]. Therefore, these above links may be employed by PrRP to regulate the expression of the hub proteins of BMDMs.
(3) PrRP may modulate the functions of the hub proteins of BMDMs in diverse manners. It is the function of proteins, rather than the protein expression, that plays a pivotal role in cellular activities [68]. Various biologically active molecules (including neuropeptides) may regulate each hub protein’s activity in a variety of ways, including phosphorylation, heterogeneous regulation, covalent modification, etc. [67]. However, this study’s focus is to investigate the effect of PrRP on the transcriptomic gene expression of BMDMs, which may provide clues for the subsequent exploration of the functional regulation of PrRP on BMDMs. Therefore, follow-up studies on the complex biological functions of PrRP on BMDMs are worth looking in to.
(4) The regulation of PrRP on the transcriptome gene expression of BMDMs may be more complicated than we previously assumed. Since proteins and various protein–protein interaction (PPI) networks are prominent members that play an essential role in regulating various biological processes in cells, the influence of neuropeptide PrRP on BMDMs may be a complicated process. In the same vein, emerging data have indicated that PrRP may have a wide range of effects in regulating the neuroendocrine system [40,69].

Secondly, the binding mode of PrRP and hub proteins needs to be verified by experiments. In this study, with the help of molecular simulation tools, the possible binding modes of PrRP and hub proteins were predicted, which may provide clues for exploring the mode of action of PrRP. However, reliable experimental verification is necessary to understand the mechanism of PrRP fully.

Finally, the key “driver” of the network formed by PrRP-activated DEGs needs to be further studied. In the present study, our data described the gene-expression signature of murine bone marrow-derived macrophages from the transcriptome level, which may provide preliminary clues for the understanding of the possible effects of PrRP on the immune system. However, considering the complexity of the gene expression regulatory network [70], it is of great significance to identify the essential molecules of PrRP to regulate the gene expression of BMDMs from the experimental level-such as these “driver” genes.

## 4. Materials and Methods

### 4.1. Ethical Statement

The study was conducted according to the guidelines of the Declaration of Helsinki, and approved by the Ethics Committee of Northwestern Polytechnical University (protocol code 201900048, 2 January 2020).

### 4.2. Animals

Male mice (C57BL/6 strain, 18–22 g) were kept in approved plastic cages with humidity of 65–74% and temperature of 20–22 °C and a photocycle of 12 h dark/light. The mice had free access to water and food. All measures were conducted to minimize animals suffering during the whole experiment process.

### 4.3. Reagents

TRIzol, Dulbecco’s modified Eagle’s medium (DMEM), β-mercaptoethanol, and Fetal bovine serum (FBS) were purchased from Invitrogen™ and Gibco™ (Thermo Fisher Scientific, Inc., Waltham, MA, USA). Streptomycin (10,000 μg/mL)/penicillin (10,000 units/mL) antibiotics and Trypsin-EDTA solution (0.05% Trypsin—EDTA) were obtained from Merck-Millipore (Merck-Millipore, Ontario, Canada).

Cell culture dishes were purchased from Corning, Inc. (Corning, NY, USA). PrimeScript ^TM^ 1st Strand cDNA Synthesis Kit and SYBR^®^ Premix Ex Taq TM II kit were acquired from TaKaRa (Dalian, China). QiaQuick PCR extraction kit was obtained from Qiagen (Venlo, The Netherlands). The red blood cell lysing solution was from Beyotime (Shanghai, China). L-929 cells were purchased from the Stem Cell Bank of the Chinese Academy of Sciences (Shanghai, China).

BCA Protein Assay Kit was from Thermo Scientific Pierce (Thermo Fisher scientific, MA, USA). PVDF membrane, ECL detection kit, and protease inhibitor cocktail III (EDTA-free) were provided by Millipore Corporation (Bedford, MA, USA). The Rabbit anti-GPR10 polyclonal antibody and horseradish peroxidase conjugated-goat anti-rabbit IgG was acquired from Thermo Fisher Scientific (Beverly, MA, USA). Rabbit anti-IFIT1 monoclonal antibody, rabbit anti-USP18 monoclonal antibody, rabbit anti-Actin monoclonal antibody, and anti-rabbit IgG (Alexa Fluor 488 Conjugate) were obtained from Cell Signaling Technology (Beverly, MA, USA). FITC Rat anti-mouse CD11b antibody and PE Rat-anti mouse F4/80 antibody were from BD Pharmingen (San Diego, CA, USA).

PrRP was synthesized by the GL Biochem Ltd. (Shanghai, China) using the solid-phase peptide synthesis method. The mass of the peptide was confirmed using a mass spectrometer (LCMS-2010EV, Shimadzu, Japan). PrRP31 was purified by HPLC, and peptide (purity > 98%) was used in this study.

### 4.4. BMDM Preparation

BMDMs were isolated by flushing mouse tibias and femurs with cold PBS. Cells were centrifuged at 1200 rpm/min for 7 min (4 °C), and the pellet was resuspended with 3 mL red blood cell lysing solution for 5 min. Then, the cell suspensions were centrifuged at 800 rpm/min for 5 min (at 4 °C), and the pellet was resuspended with 10 mL PBS. Next, the cell suspensions were filtered through a sterile filter (100 mesh) to collect a single cell suspension.

The single-cell suspensions were maintained in DMEM complete culture medium (streptomycin (100 μg/mL)/penicillin (100 units/mL), 10% FBS) at 37 °C with 5% CO_2_ in a humidified incubator. Fourteen h later, the non-adherent cell supernatant was obtained, centrifuged at 800 rpm/min for 5 min (4 °C), and the pellet was resuspended in DMEM complete cell culture medium. Next, cells were maintained in a 100-mm culture dish supplemented with 20% L-929 conditioned media for 8 d (the fresh medium was refreshed every 3 d). At 8 days, the cells were differentiated into BMDMs, where more than 96% of the cells were double positive for F4/80 and CD11b.

### 4.5. RNA-seq Sample Preparation and Microscope Detection

BMDMs were exposed to PrRP (1 nM) for 18 h, followed by RNA sequencing examination. Cells were washed with PBS (3 times) and lysed with TRIzol (1 mL). Next, cell lysates were quickly stored in liquid nitrogen. Subsequently, the RNA-seq detection was performed by the Novogene Co Ltd. (Beijing, China).

Cell images were acquired by a confocal microscope (Leica TCS SP5, Leica Microsystems, Mannheim, Germany). In addition, the detailed structure of BMDMs was detected by a transmission electron microscope HT7700 (Hitachi High-Technologies, Tokyo, Japan).

### 4.6. Flow Cytometry Detection

Cells were rinsed twice with pre-cooled buffer (BSA-PBS-1%) and washed with trypsin (0.25%). The cells were resuspended in a fresh medium to form a uniform cell suspension, followed by centrifugation (1000/rpm, 5 min). Next, the pelleted cells acquired with centrifugation were resuspended in a buffer solution (BSA-PBS-1%) to form a uniform cell suspension. Five min later, the cells were centrifuged (1000/rpm, 5 min), and the pellet was resuspended in a buffer solution (BSA-PBS-1%). Then, the number of cells were counted (1 × 10^6^/100 μL). The antibody was added into the cell suspension, followed by incubating in the dark for 25 min. During the whole incubation process, the cell mixture was mixed every 2 min to make sure the antibody and the cells to be thoroughly combined. Subsequently, the cell mixture was subjected to centrifugation (1000 rpm/min, 5 min), and the supernatant was discarded. Then, 400 uL of buffer solution (BSA-PBS-1%) was added into the tube to resuspend the cell pellet. Next, the whole-cell suspension was filtered with a 300-mesh sterile filter to ensure that only single cells were subjected to flow cytometry detection. Finally, the purity of macrophages was detected by flow cytometry (BD Calibur, Biosciences, San Jose, CA, USA), followed by analysis with the Cellquest (BD) and Modfit software.

### 4.7. RNA-seq Sample Collection and Preparation

#### 4.7.1. RNA Quantification and Qualification

BMDMs RNA was detected on 1% agarose gels. RNA purity was examined with a NanoPhotometer^®^ spectrophotometer (IMPLEN, CA, USA). RNA integrity was investigated with a RNA Nano 6000 Assay Kit of the Bioanalyzer 2100 system (Agilent Technologies, CA, USA). RNA concentration was assessed with a Qubit^®^ RNA Assay Kit of Qubit^®^2.0 Fluorometer (Life Technologies, Carlsbad, CA, USA).

#### 4.7.2. Library Construction for RNA Sequencing

A total of 1 µg RNA (per sample) was acquired as input RNA for the RNA sample examination. RNA libraries were constructed with a NEBNext^®^ UltraTM RNA Library Prep Kit for Illumina^®^ (Lincoln, NE, USA).

#### 4.7.3. Sequencing and Clustering

The clustering of samples was conducted with the cBot Cluster Generation System and the TruSeq PE Cluster Kit v3-cBot-HS (Illumia). After cluster generation was carried out, the samples were sequenced with the Illumina Hiseq platform. Finally, 125 bp/150 bp paired-end reads were acquired.

### 4.8. RNA-seq Data Interpretation

#### 4.8.1. Quality Control, Mapping Reads to the Reference Genome and Quantification of Gene Expression

Low-quality reads were removed, and the clean reads with high quality were used. The paired-end clean reads were aligned to the musculus reference genome with the Hisat2 v2.0.5. Finally, the FPKM (fragments per kilobase of exon model per million reads mapped) of each gene was calculated, and the read was mapped to each gene by using the FeatureCounts v1.5.0-p3.

#### 4.8.2. Differential Expression Interpretation

Differential expression gene (DEGs) detection was conducted with the DESeq2 R package (1.16.1). The *p*-values were adjusted with the Benjamini and Hochberg’s method to analyze the false discovery rate (FDR). Genes with an adjusted *p*-value < 0.05 were considered as DEGs. The heat map of DEGs was generated by the toolkit TBtools [71].

#### 4.8.3. GO and KEGG Enrichment Interpretation

In order to analyze the ontology (GO) enrichment of differentially expressed genes (DEGs), the R package “ClusterProfiler” [72] was utilized. GO terms (adjusted *p* < 0.05) were considered as significantly enriched.

To explore the pathways associated with DEGs, the enrichment study was performed with Metascape online tool (http://metascape.org/, date of retrieving data: 8 November 2020) [73,74], and the significant biological processes were obtained.

To further investigate the functions of DEGs, DEGs were subjected to further analysis using PANTHER (http://www.pantherdb.org/, date of retrieving data: 11 November 2020) [75]. The main GO categories were acquired, including molecular function (MF), biological process (BP), and cellular component (CC).

Besides, KEGG (http://www.genome.jp/kegg/, date of retrieving data: 11 November 2020) was used to explore the functional enrichment of DEGs. To show the KEGG pathway maps clearly, a Cytoscape plug-in KEGGParser was employed to show biological networks.

#### 4.8.4. Protein-Protein Interaction (PPI) Network Analysis

In order to explore the interaction among DEGs, all DEGs were analyzed with the online tool STRING (http://string-db.org, date of retrieving data: 15 November 2020) [76]. Only experimentally proved interactions (a combined score > 0.4) were considered significant. Next, the PPI network was built with the Cytoscape software (Ver3.8.0), and the Cytoscape plug-in (Molecular Complex Detection, MCODE) was utilized to analyze the modules of the PPI network. Subsequently, function enrichment analysis for DEGs of the modules was conducted (*p* < 0.05 was considered as statistically significant).

To further explore the pathways DEGs, pathway enrichment analysis was carried out by Cytoscape software and two plug-ins (clueGO (http://apps.cytoscape.org/apps/cluego) [77] and Cluepedia (http://apps.cytoscape.org/apps/cluepedia, date of retrieving data: 15 November 2020)) [78]. Subsequently, KEGG pathway enrichment analysis was conducted with ClueGO and CluePedia tool kits, and pathways with *p* < 0.05 and kappa coefficient > 0.4 were selected.

In order to identify the top-ranked hub genes from the PPI network, CytoHubba (a Cytoscape plug-in) was used in the following analysis. A total of eight hub genes were obtained according to the scores of the following methods, including MCC, MNC, Radiality, DMNC, Degree, Betweenness, Closeness, BottleNeck, EcCentricity, EPC, Stress and Clustering Coefficient [79,80].

### 4.9. qPCR Analysis

This approach has been described elsewhere [81]. Briefly, total RNA was extracted from cells with the TRIzol following the manufacturer’s instruction. Then, cDNA was transcribed from 1 μg of RNA with a PrimeScript ^TM^ 1st Strand cDNA Synthesis Kit. Next, the gene expression level was detected with the SYBR^®^ Premix Ex Taq TM II system and the MX3000P Real-Time PCR System (Stratagene). Real-time PCR conditions were set as follows with minor modifications: 94 °C for 30 s, 95 °C for 5 s, 56 °C. Data were normalized by GAPDH data using the comparative 2^−ΔΔCT^ approach. Primers were shown in Table 9. Data were acquired in duplicates three times.

### 4.10. Western Blot Detection

This approach has been previously described [82]. In brief, cells were washed with phosphate-buffered saline3 times and lysed by lysis solution (150 mM NaCl, 5 mM EGTA, 1% Nonidet P-40, 50 mM Tris/HCl, 0.5% sodium deoxycholate, 1 unit protease inhibitor cocktail III (EDTA-free), 0.1% SDS, PH 7.4). Then, cell lysates were centrifuged (12,000× *g* for 12 min, 4 °C), and the protein concentration of the supernatants was evaluated by the BCA Protein Assay Kit. Samples for immunoblot (18–24 µg/lane) were analyzed with the Bio-Rad mini-gel system by 10% SDS-polyacrylamide gel electrophoresis. Next, the proteins were blotted onto PVDF membranes using the Bio-Rad wet blotter system. The membranes were blocked with the solution (5% non-fat milk in the tris-buffered saline containing 0.05% tween-20) for 1 h. Then, the membranes were rinsed three times with TBST and were treated at 4 °C with appropriate antibodies (anti-GPR10 was 1:1000, and anti-Rabbit antibody was 1:10,000, and anti-Actin was 1:5000) for 12 h. The membranes were rinsed with TBST 3 times, followed by treated with horseradish peroxidase-conjugated secondary antibodies at room temperature for 2 h. Subsequently, the PVDF membranes were detected using an ECL detection kit. The band intensity was analyzed by the ChemDocTM XRS (Bio-Rad, Hercules, CA, USA) and Image J [83].

### 4.11. Immunofluorescence Stain

Immunofluorescence stain was conducted as previously described [84]. In brief, BMDMs were cultured on the glass slides, followed by fixing with 4% paraformaldehyde for 15 min. Then, cells were rinsed with PBS 3 times, exposed to 0.1% Triton X-100 (12 min), and treated with 5% normal rabbit serum (3 h). Next, BMDMs were incubated with rabbit anti-GPR10 polyclonal antibody (1:250) at 4 °C for 14 h, followed by incubation with anti-rabbit IgG (Alexa Fluor 488 Conjugate) for 1.5 h. Subsequently, cells were treated with DAPI for 5 min to stain the nucleus. All figures were captured with a confocal microscope (Leica TCS SP5, Leica Microsystems, Mannheim, Germany).

### 4.12. Homology Modeling of Proteins

The three-dimensional (3-D) structures of proteins were built using homology modeling (the modeling of Ifi44 and Rtp4 was conducted with Robetta de novo structure prediction program [85,86] due to the lack of sufficient templates). Briefly, protein sequences were acquired from the NCBI nucleotide database (https://www.ncbi.nlm.nih.gov/protein/, date of retrieving data: 27 September 2020), and the blast module was employed to align the hub protein sequence in the PDB database [87]. Protein templates were retrieved from the online RCSB PDB Protein Data Bank. Firstly, three templates (query cover > 45%) for each protein were selected for homology modeling. Next, 3-D models of the hub protein were constructed using Modeller (9v23) [88]. Modules for multiple template modeling were used, including Align2d, Salign, and Model. One thousand candidate three-dimensional models for each protein were built, and the model with the lowest discrete optimized protein energy (DOPE) value was finally selected. Finally, all models were subjected to the online tool MolProbity (http://molprobity.biochem.duke.edu/, date of retrieving data: 13 December 2020) for quality evaluation [89].

### 4.13. Molecular Dynamics (MD) Simulation

The molecular dynamics simulation of the hub protein was performed by using the Gromacs 2018.12 [90]. All simulations were carried out in the CHARMM36 force field [91].

#### 4.13.1. Molecular Dynamic Simulation: Protein in Water

Molecular dynamics simulation was performed in a condition with minor modifications. The protein model was solvated in an octahedron box with a TIP3P water model (1.0 nm). The simulated system was neutralized by adding Cl^−^ or Na^+^ ions, and periodic boundary conditions (PBC) were used in all directions. Next, energy minimization for the protein model was conducted with the steepest descent (50,000 steps) with the max force (<100 KJ/mol). Subsequently, the whole simulation was performed under equilibration phases with NVT (100 ps, 298.15 K) and NPT (300 ps, 298.15 K, 1.0 bar), respectively. The whole simulation was conducted at least 300 ns for each protein.

#### 4.13.2. Molecular Dynamic Simulation: Analysis

The MD trajectory was analyzed using GROMACS utilities to obtain the RMSF (root mean square fluctuation), RMSD (root mean square deviation), and radius of gyration (Rg). The 3-D structures of proteins were drawn with the Pymol software (Delano, W.L. The Pymol Molecular Graphics System (2002) DeLano Scientific, SanCarlos, CA, USA. http://www.pymol.org, date of retrieving data: 11 November 2020).

### 4.14. Dock

The 3-D model of PrRP was constructed by PEP-FOLD3 [92], and the 3-D structure of hub proteins was obtained from the MD-optimized protein structures.

(1) The primary docking complex, consisting of PrRP (ligand) and hub protein (receptor), was constructed by ZDOCK (3.02) [93,94,95].
(2) The primary docking complexes were sent to the Flexible peptide docking module of the Rosetta program (3.9) for subsequent docking. (A) Pre-pack mode: 1 model was constructed. (B) Low-resolution ab-initio analysis: 100 models were obtained, and 1 model with the best docking score was chosen for subsequent analysis. (C) Refinement analysis: 100 docking models were acquired, and a docking model was finally selected based on the total_score.

### 4.15. Statistical Analysis

Data were demonstrated as means ± standard error of the mean (S.E.M). Data were interpreted by one-ANOVA followed by the post hoc tests (Tukey). The statistical analysis was performed by using the GraphPad Prism software version 8.0 (San Diego, CA, USA).

## 5. Conclusions

Our work showed that PrRP significantly changed the transcriptome profile of BMDMs, implying that PrRP may be involved in various physiological activities mastered by macrophages. Our data provided clues for in-depth exploration of the role of PrRP in regulating diverse physiological processes governed by macrophages.

## Figures and Tables

**Table 1:** Details of DEGs-protein_coding (up-regulated and down-regulated 24 proteincodingotein_coding genes).

Gene_Symbol	log2FoldChange	Padj	Gene_Chrosome	Change
Entpd4b	−3.707639059	2.19 × 10^−2^	14	Down
Hspb7	−3.272825898	1.68 × 10^−2^	4	Down
Dclk2	−2.383273176	4.25 × 10^−2^	3	Down
Slc7a15	−2.101674471	2.79 × 10^−2^	12	Down
Nanos1	−1.666406569	6.68 × 10^−2^	19	Down
Dach1	−1.613454215	1.72 × 10^−3^	14	Down
Srpk3	−1.591562463	6.24 × 10^−3^	X	Down
Slc6a4	−1.584727023	3.21 × 10^−2^	11	Down
Nptx1	−1.450738194	3.30 × 10^−6^	11	Down
Smpd3	−1.391289481	7.65 × 10^−4^	8	Down
Pgm5	−1.31912871	4.92 × 10^−2^	19	Down
Hmga2	−1.300711982	2.19 × 10^−14^	10	Down
Fam78b	−1.285355042	1.93 × 10^−3^	1	Down
Cmbl	−1.256341752	2.78 × 10^−2^	15	Down
Chst3	−1.22498333	1.07 × 10^−3^	10	Down
Slco2b1	−1.20549131	1.27 × 10^−4^	7	Down
Klhl3	−1.173243947	1.22 × 10^−2^	13	Down
Slc24a3	−1.163460987	1.40 × 10^−4^	2	Down
Prokr1	−1.100130971	9.67 × 10^−4^	6	Down
Efr3b	−1.063158734	5.39 × 10^−13^	12	Down
Sox4	−1.052753293	2.51 × 10^−2^	13	Down
Fabp7	−1.049413652	1.71 × 10^−2^	10	Down
Etl4	−1.039530137	1.92 × 10^−3^	2	Down
Angptl2	−1.033436675	1.59 × 10^−23^	2	Down
Saa3	6.725187234	1.04 × 10^−272^	7	Up
Cfb	6.339511013	1.73 × 10^−5^	17	Up
Lad1	6.049625834	8.45 × 10^−5^	1	Up
Iigp1	5.288407326	5.88 × 10^−35^	18	Up
Cacng8	5.269289134	1.18 × 10^−4^	7	Up
Ly6i	5.021584581	5.25 × 10^−41^	15	Up
Serpina3g	4.932160399	1.43 × 10^−11^	12	Up
Cdkn2a	4.865779496	9.13 × 10^−3^	4	Up
Ptx3	4.863346866	8.39 × 10^−3^	3	Up
Acod1	4.818905659	2.31 × 10^−292^	14	Up
Adgre4	4.767373764	1.20 × 10^−94^	17	Up
Il1b	4.68955843	1.37 × 10^−46^	2	Up
Ly6a	4.571707887	8.85 × 10^−71^	15	Up
Lpar1	4.533771177	4.00 × 10^−6^	4	Up
Fpr1	4.518260903	7.01 × 10^−59^	17	Up
Cxcl10	4.51118115	8.99 × 10^−41^	5	Up
Ccl5	4.470370636	2.30 × 10^−128^	11	Up
Gbp4	4.398911115	4.55 × 10^−11^	5	Up
Marco	4.320725477	6.79 × 10^−122^	1	Up
Fpr2	4.318699393	5.20 × 10^−73^	17	Up
Ly6c2	4.262622986	2.41 × 10^−27^	15	Up
Serpina3f	4.249141119	3.35 × 10^−5^	12	Up
Ppm1n	4.157670609	4.40 × 10^−16^	7	Up
Apol9b	4.156227055	1.44 × 10^−2^	15	Up

**Table 2:** Details of DEGs-antisense RNA.

Gene_Symbol	log2FoldChange	Padj	Gene_Chrosome	Change
Gm45820	−1.686832907	2.71 × 10^−2^	8	Down
Gm28800	−1.563748269	2.10 × 10^−2^	1	Down
Dnmt3aos	−1.056605119	2.92 × 10^−5^	12	Down
Gm13822	4.654103949	2.29 × 10^−2^	5	Up
AC113595.1	1.919370036	7.44 × 10^−4^	15	Up
Gm11772	1.832830691	1.76 × 10^−4^	11	Up

**Table 3:** Details of DEGs-lincRNA.

Gene_Symbol	log2FoldChange	Padj	Gene_Chrosome	Change
AW112010	2.84032013	3.87 × 10^−31^	19	Up
Gm34643	2.150264718	2.24 × 10^−5^	14	Up
Gm36161	1.365143899	6.92 × 10^−26^	13	Up
Gm17705	1.333355523	7.90 × 10^−3^	17	Up
Gm14221	−1.62639326	2.98 × 10^−4^	2	Down
Gm37168	−1.332031628	4.17 × 10^−5^	1	Down
Gm16907	−1.027914414	7.90 × 10^−3^	13	Down

**Table 4:** Hub genes of PrRP-treated BMDMs.

Gene Name Ensembl ID	Species Gene Type	Location Length	Expression Changes (PrRP vs. Control)	Function	Refs.
*Ifit1* (Interferon Induced Protein with Tetratricopeptide Repeats 1) (ENSEMBL: ENSG00000185745)	Mus musculus Protein coding	Chr 19 (2638 bp)	Up-regulated	Activities: Inhibit the translational initiation and replication of virus. Diseases: Hepatitis and Hepatitis C. Pathways: innate immune system and interferon gamma signaling.	[17]
*Oasl2* (2’-5’ oligoadenylate synthetase-like 2) (ENSMUSG00000029561)	Mus musculus Protein coding	Chr 5 (3136 bp)	Up-regulated	Activities: is involved in the innate immune response to viral infection. Diseases: microphthalmia with limb anomalies and tick-Borne encephalitis. Pathways: innate immune system and interferon gamma signaling.	[18]
*Irf7* (Interferon Regulatory Factor 7) (ENSG00000185507)	Mus musculus Protein coding	Chr 7 (1876 bp)	Up-regulated	Activities: regulates the transcriptional activation of virus-inducible cellular genes such as interferon beta chain genes. Diseases: influenza and immunodeficiency 39. Pathways: activated TLR4 signaling and apoptosis modulation and signaling.	[19]
*Ifit3* (Interferon Induced Protein with Tetratricopeptide Repeats 3) (ENSG00000119917)	Mus musculus Protein coding	Chr 19 (1998 bp)	Up-regulated	Activities: inhibits cellular events, including viral processes, signaling, proliferation, cell migration, and viral replication. Diseases: systemic lupus erythematosus and lupus erythematosus. Pathways: innate immune system and interferon gamma signaling.	[17]
*Ifit2* (Interferon Induced Protein with Tetratricopeptide Repeats 2) (ENSG00000119922)	Mus musculus Protein coding	Chr 19 (3949 bp)	Up-regulated	Activities: inhibits the expression of viral mRNAs. Diseases: microphthalmia with limb anomalies. Pathways: innate immune system and interferon gamma signaling.	[20]
*Usp18* (Ubiquitin Specific Peptidase 18) (ENSG00000184979)	Mus musculus Protein coding	Chr 6 (1778 bp)	Up-regulated	Activities: inhibits interferon responses. Diseases: torch syndrome and pseudo-torch syndrome 2. Pathways: interferon gamma signaling and immune response IFN alpha/beta signaling pathway.	[21]
*Ifi44* (Interferon Induced Protein 44) (ENSG00000137965)	Mus musculus Protein coding	Chr 3 (2860 bp)	Up-regulated	Activities: aggregates to form microtubular structures. Diseases: Potocki-Shaffer syndrome and hepatitis D. Pathways: interferon gamma signaling.	[22]
*Rtp4* (Receptor Transporter Protein 4) (ENSG00000136514)	Mus musculus Protein coding	Chr 16 (1573 bp)	Up-regulated	Activities: enhances functional expression of the opioid receptor heterodimer OPRM1-OPRD1. Diseases: pain-related diseases. Pathways: olfactory transduction and signaling by GPCR.	[23]

**Table 5:** GO analysis of hub genes.

Gene	GO Analysis [24,25]
*Ifit1*	MF: RNA binding
	BP: immune system process; response to stimulus
	CC: cytosol
*Oasl2*	MF: carbohydrate derivative binding; RNA binding; transferase
	BP: immune system process; response to stimulus
	CC: cytosol; nucleus; organelle lumen
*Irf7*	MF: DNA binding; transcription
	BP: immune system process; nucleic acid-templated transcription; response to stimulus; signaling; system development
	CC: cytosol; nucleus; organelle lumen
*Ifit3*	MF: RNA binding
	BP: immune system process; response to stimulus
	CC: cytosol; mitochondrion
*Ifit2*	MF: RNA binding
	BP: cell death; immune system process; response to stimulus
	CC: cytosol; endoplasmic reticulum
*Usp18*	MF: hydrolase
	BP: protein metabolic process; response to stimulus
	CC: cytosol; nucleus
*Ifi44*	MF: none
	BP: immune system process; response to stimulus
	CC: none
*Rtp4*	MF: signaling receptor binding
	BP: cellular component organization; establishment of localization; immune system process; response to stimulus
	CC: none

Note: MF: Molecular Function; CC: Cellular Component; BP: Biological Process. This information was obtained from the Mouse Genome Database (MGD) at the Mouse Genome Informatics website, the Jackson Laboratory, Bar Harbor, Maine (URL: http://www.informatics.jax.org) [24,25] (date of retrieving data: 11 January 2021).

**Table 6:** Transcriptional factors tied to the up-regulated DEGs.

#	Key TF	Description	# of Overlapped Genes	*p* Value	FDR
1	Irf1	interferon regulatory factor 1	5	2.16 × 10^−9^	3.46 × 10^−8^
2	Stat1	signal transducer and activator of transcription 1	5	1.12 × 10^−8^	8.92 × 10^−8^
3	Nfkb1	nuclear factor of kappa light polypeptide gene enhancer in B cells 1, p105	6	4.95 × 10^−6^	2.64 × 10^−5^
4	Jun	jun proto-oncogene	5	1.02 × 10^−5^	4.07 × 10^−5^
5	Irf8	interferon regulatory factor 8	3	1.74 × 10^−5^	5.56 × 10^−5^
6	Rel	reticuloendotheliosis oncogene	3	3.19 × 10^−5^	8.50 × 10^−5^
7	Rela	v-rel reticuloendotheliosis viral oncogene homolog A (avian)	4	1.47 × 10^−4^	0.000336
8	Foxo3	forkhead box O3	2	1.71 × 10^−4^	0.000341
9	Irf4	interferon regulatory factor 4	2	2.60 × 10^−4^	0.000462
10	Ikbkb	inhibitor of kappaB kinase beta	2	3.11 × 10^−4^	0.000498
11	Foxm1	forkhead box M1	2	1.18 × 10^−3^	0.00171
12	Hdac1	histone deacetylase 1	2	1.74 × 10^−3^	0.00232
13	Spi1	spleen focus forming virus (SFFV) proviral integration oncogene	2	3.54 × 10^−3^	0.00435
14	Cebpb	CCAAT/enhancer binding protein (C/EBP), beta	2	3.89 × 10^−3^	0.00445
15	Fos	FBJ osteosarcoma oncogene	2	6.83 × 10^−3^	0.00729
16	Stat3	signal transducer and activator of transcription 3	2	1.32 × 10^−2^	0.0132

Note: data acquired from TRRUST (version 2) (https://www.grnpedia.org/trrust/, date of retrieving data: 9 January 2021).

**Table 7:** Protein modeling templates.

Proteins	Species	Protein Length (aa)	Model Templates (Query Cover, Identify)
Ifit1	Mus musculus	461	5W5H_A (2.79 Å) (99%, 52.98%) 6C6K_A (2.54 Å) (97%, 53.25%) 5UDI_A (1.58 Å) (95%, 53.22%)
Oasl2	Mus musculus	439	4XQ7_A (1.60 Å) (68%, 49%) 4IG8_A (2.70 Å) (65%, 45.40%) 1PX5_A (1.74 Å) (66%, 43.02%)
Irf7	Mus musculus	330	1QWT_A (2.10 Å) (49%, 28.57%) 1J2F_A (2.30 Å) (49%, 28.57%) 5JEJ_A (2.00 Å) (46%, 28.97%)
Ifit3	Mus musculus	391	4G1T_A (2.80 Å) (99%, 51.33%) 6C6K_A (2.54 Å) (92%, 41.58%) 5W5H_A (2.79 Å) (92%, 41.58%)
Ifit2	Mus musculus	468	4G1T_A (2.80 Å) (98%, 63.03%) 5UDI_A (1.58 Å) (92%, 41.03%) 5W5H_A (2.79 Å) (92%, 41.03%)
Usp18	Mus musculus	308	5CHV_A (3.00 Å) (87%, 100%) 5CHT_A (2.80 Å) (87%, 100%) 2F1Z_A (3.20 Å) (87%, 26.89%)
Ifi44	Mus musculus	420	De novo by Rosetta
Rtp4	Mus musculus	247	De novo by Rosetta

**Table 8:** Ramachandran plot analysis.

Proteins	Number of Residues in Favoured Regions	Number of Residues in Allowed Region	Number of Outliers
Ifit1	452/461 (98%)	459/461 (99.6%)	2 (0.004%)
Oasl2	408/439 (92.9%)	433/439 (98.6%)	6 (0.014%)
Irf7	295/330 (89.4%)	316/330 (95.8%)	14 (0.042%)
Ifit3	372/391 (95.1%)	386/391 (98.7%)	5 (0.013%)
Ifit2	450/468 (96.2%)	459/468 (98.1%)	9 (0.019%)
Usp18	292/308 (94.8%)	304/308 (98.7%)	4 (0.013%)
Ifi44	399/420 (95.0%)	420/420 (100.0%)	0 (0.000%)
Rtp4	237/247 (96.0%)	247/247 (100.0%)	0 (0.000%)

Note: part of the N-terminal sequence of the hub proteins were removed for subsequent molecular dynamics simulation.

**Table 9:** Primers for qPCR.

Genes	Primers	Sequences (5′ to 3′)	Products (bp)
*Gapdh*	Forward	TGTGTCCGTCGTGGATCTGA	150
	Reverse	TTGCTGTTGAAGTCGCAGGAG	
*Ifit1*	Forward	TTGTTGTTGTTGTTGTTC	127
	Reverse	GTGAGTATGTATCCTTGG	
*Oasl2*	Forward	TGTTGGATGATGAGGAGTTG	75
	Reverse	GTATGATGGTGTCGCAGTC	
*Irf7*	Forward	AATCTACACTGAGTTCTG	154
	Reverse	GACCAAGTTTCACAAATG	
*Ifit3*	Forward	GTCCTTTGAACTCCTACTC	80
	Reverse	GCTCTCCTTACTGATGAC	
*Ifit2*	Forward	TATATGACACAGACAGAG	163
	Reverse	TCTAACTTCTTCCTATCC	
*Usp18*	Forward	CTTAGGTGACAGAACTTG	94
	Reverse	AACAGGAAGAAGAACTATTAG	
*Ifi44*	Forward	TAGTTCTGCTTGCTTCTC	180
	Reverse	TCTGTGCCTTCTTCATTC	
*Rtp4*	Forward	TCAGAAGTGCCAGAAGTG	123
	Reverse	TTCCTGTGTCCATAGTATCTC	

## Data Availability

The data presented in this study are available in article and Supplementary Material.

## Supplementary Materials

The following are available online at https://www.mdpi.com/article/10.3390/ijms22094456/s1.
