# Supplementary material for: Prolactin-Releasing Peptide Differentially Regulates Gene Transcriptomic Profiles in Mouse Bone Marrow-Derived Macrophages
Source: Int J Mol Sci. 2021 Apr 24;22(9):4456. doi: 10.3390/ijms22094456 (PMC8123224; doi:10.3390/ijms22094456)
Supplement: Supplementary file 1 [file ijms-22-04456-s001.zip › Supplementary File 1_Table S1 PrRPvsControl_deg_all.pdf]

gene_id	PrRP_1	PrRP_2	PrRP_3	Ctrl_1	Ctrl_2	Ctrl_3	PrRP	Control
ENSMUSG(	7.871623	5.243306	3.690681	5.07311	69.33034	144.9514	5.60187	73.11829
ENSMUSG(	1.967906	1.048661	0.92267	7.102355	19.80867	11.07268	1.313079	12.66123
ENSMUSG(	1.967906	3.145984	2.76801	7.102355	18.81823	15.09911	2.6273	13.67323
ENSMUSG(	3.935811	2.097323	5.536021	18.2632	16.83737	15.09911	3.856385	16.73322
ENSMUSG(	5.903717	6.291968	11.99471	34.49715	30.70344	19.12554	8.063465	28.10871
ENSMUSG(	9.839528	7.340629	11.07204	26.38017	38.6269	32.21143	9.4174	32.40617
ENSMUSG(	13.77534	4.194645	25.83476	40.58488	63.38774	43.28411	14.60158	49.08558
ENSMUSG(	8.855576	4.194645	9.226701	25.36555	27.73214	19.12554	7.425641	24.07441
ENSMUSG(	31.48649	15.72992	11.07204	53.77497	60.41644	70.4625	19.42948	61.5513
ENSMUSG(	23.61487	12.58394	23.06675	77.11128	56.4547	50.33036	19.75519	61.29878
ENSMUSG(	20.66301	8.38929	15.68539	44.64337	43.57907	49.32375	14.91256	45.84873
ENSMUSG(	9.839528	10.48661	13.84005	30.43866	40.60777	32.21143	11.38873	34.41929
ENSMUSG(	8.855576	7.340629	9.226701	33.48253	17.8278	25.16518	8.474302	25.49184
ENSMUSG(	15.74325	5.243306	11.07204	25.36555	36.64604	33.21804	10.6862	31.74321
ENSMUSG(	43.29392	31.45984	31.37078	99.43296	99.04334	91.60125	35.37485	96.69252
ENSMUSG(	21.64696	36.70315	21.22141	57.83346	71.3112	78.51536	26.52384	69.22001
ENSMUSG(	166.288	58.72503	129.1738	289.1673	295.1492	309.0284	118.0623	297.7816
ENSMUSG(	11.80743	9.437952	20.29874	39.57026	40.60777	24.15857	13.84804	34.77887
ENSMUSG(	141.6892	153.1045	159.6219	328.7376	384.2882	406.6693	151.4719	373.2317
ENSMUSG(	35.4223	18.8759	28.60277	57.83346	70.32077	74.48893	27.63366	67.54772
ENSMUSG(	12.79139	30.41118	14.76272	44.64337	40.60777	52.34357	19.32176	45.8649
ENSMUSG(	55.10136	26.21653	61.8189	107.5499	109.9381	118.7796	47.71226	112.0892
ENSMUSG(	32.47044	30.41118	24.91209	72.03817	63.38774	69.45589	29.26457	68.29393
ENSMUSG(	57.06926	40.89779	76.58162	122.7693	156.4885	124.8193	58.18289	134.6923
ENSMUSG(	18.6951	25.16787	44.28817	64.93581	67.34947	67.44268	29.38371	66.57599
ENSMUSG(	38.37416	67.11432	52.5922	115.6669	115.8807	121.7995	52.69356	117.7824
ENSMUSG(	81.66809	37.75181	70.12293	140.0178	136.6798	130.8589	63.18094	135.8522
ENSMUSG(	598.2433	443.5837	615.421	1141.45	1168.711	1155.585	552.416	1155.249
ENSMUSG(	82.65204	62.91968	83.04031	146.1056	179.2684	150.9911	76.20401	158.7884
ENSMUSG(	50.18159	24.11921	25.83476	64.93581	65.3686	77.50875	33.37852	69.27106
ENSMUSG(	24.59882	26.21653	25.83476	53.77497	51.50254	53.35018	25.55004	52.87589
ENSMUSG(	27.55068	20.97323	21.22141	53.77497	44.5695	45.29732	23.24844	47.8806
ENSMUSG(	86.58785	41.94645	70.12293	136.974	150.5459	121.7995	66.21908	136.4398
ENSMUSG(	834.392	804.3232	916.2114	1740.077	1726.325	1765.589	851.6422	1743.997
ENSMUSG(	130.8657	57.67637	140.2459	217.1291	214.924	241.5857	109.596	224.5463
ENSMUSG(	163.3362	67.11432	209.4461	276.9918	319.91	301.9821	146.6322	299.628
ENSMUSG(	318.8007	272.6519	292.4864	584.4223	603.1739	615.037	294.6464	600.8777
ENSMUSG(	996.7442	524.3306	989.1024	1605.132	1708.498	1776.662	836.7257	1696.764
ENSMUSG(	39.35811	58.72503	44.28817	91.31599	87.15814	108.7136	47.4571	95.72923
ENSMUSG(	782.2425	579.9097	687.3892	1361.623	1417.31	1313.622	683.1805	1364.185
ENSMUSG(	523.4629	357.5935	549.9114	900.9844	957.7491	984.4618	476.9893	947.7318
ENSMUSG(	122.0102	90.18487	93.18968	173.5004	210.9623	221.4536	101.7949	201.9721
ENSMUSG(	53.13345	66.06566	50.74686	112.6231	92.11031	130.8589	56.64866	111.8641
ENSMUSG(	56.08531	58.72503	41.52016	94.35985	94.09117	115.7598	52.11017	101.4036
ENSMUSG(	46.24578	36.70315	45.21084	88.27212	76.26337	82.54178	42.71992	82.35909
ENSMUSG(	30.50254	33.55716	39.67482	70.00892	60.41644	68.44928	34.57817	66.29155
ENSMUSG(	73.79646	77.60094	84.88565	151.1787	153.5172	143.9448	78.76102	149.5469
ENSMUSG(	44.27788	30.41118	59.05089	87.2575	86.16771	79.52196	44.57998	84.31572
ENSMUSG(	321.7526	225.4622	310.9398	523.545	544.7384	545.5811	286.0515	537.9548

ENSMUSG(	527.3987	498.1141	548.0661	1007.52	935.9596	1002.581	524.5263	982.02
ENSMUSG(	44.27788	31.45984	26.75743	64.93581	68.3399	57.37661	34.16505	63.55077
ENSMUSG(	622.8421	660.6566	651.4051	1188.122	1182.577	1208.935	644.968	1193.212
ENSMUSG(	72.81251	92.28219	87.65366	170.4565	127.7659	169.11	84.24946	155.7775
ENSMUSG(	93.47552	62.91968	83.96298	167.4126	147.5746	129.8523	80.11939	148.2798
ENSMUSG(	5832.872	5426.822	5656.891	10375.53	10512.46	10342.89	5638.862	10410.29
ENSMUSG(	233.1968	264.2626	198.3741	439.3314	425.8864	415.7287	231.9445	426.9822
ENSMUSG(	57.06926	80.74692	83.04031	116.6815	148.565	139.9184	73.61883	135.055
ENSMUSG(	50.18159	34.60582	42.44283	72.03817	93.10074	68.44928	42.41008	77.86273
ENSMUSG(	55.10136	29.36252	54.43754	79.14052	90.12944	85.56161	46.30047	84.94386
ENSMUSG(	130.8657	89.13621	142.0912	225.2461	215.9145	220.447	120.6977	220.5358
ENSMUSG(	224.3412	213.9269	263.8837	442.3752	391.2212	439.8873	234.0506	424.4946
ENSMUSG(	126.9299	75.50361	155.9313	215.0999	232.7518	202.328	119.4549	216.7266
ENSMUSG(	38.37416	48.23842	41.52016	65.95044	85.17727	77.50875	42.71091	76.21215
ENSMUSG(	45.26183	52.43306	64.58691	97.40372	89.13901	103.6805	54.09393	96.74109
ENSMUSG(	865.8785	789.642	914.3661	1478.304	1565.875	1504.878	856.6288	1516.352
ENSMUSG(	60.02112	50.33574	50.74686	86.24288	104.9859	93.61446	53.70124	94.94776
ENSMUSG(	301.0896	252.7274	314.6305	486.004	532.8532	518.4027	289.4825	512.4199
ENSMUSG(	57.06926	40.89779	44.28817	81.16977	72.30164	96.63428	47.41841	83.36856
ENSMUSG(	120.0422	174.0778	70.12293	206.9829	200.0675	229.5064	121.4143	212.1856
ENSMUSG(	149.5608	135.2773	153.1632	270.9041	249.5892	246.6187	146.0005	255.704
ENSMUSG(	35.4223	62.91968	48.90152	79.14052	92.11031	85.56161	49.08117	85.60414
ENSMUSG(	185.9671	196.0997	181.766	311.489	316.9387	351.3059	187.9443	326.5779
ENSMUSG(	188.9189	109.0608	193.7607	270.9041	300.1013	284.8698	163.9135	285.2917
ENSMUSG(	47991.32	29987.52	56799.57	73987.26	78414.59	80936.25	44926.14	77779.37
ENSMUSG(	61.98903	49.28708	53.51487	85.22825	102.0146	97.64089	54.93033	94.96126
ENSMUSG(	111.1867	71.30897	98.7257	180.6027	136.6798	168.1034	93.74045	161.7953
ENSMUSG(	437.859	445.6811	459.4897	813.7269	766.5954	731.8034	447.6766	770.7086
ENSMUSG(	774.3709	633.3914	796.2643	1174.932	1232.099	1358.92	734.6755	1255.317
ENSMUSG(	567.7408	262.1653	601.5809	834.0193	819.0884	789.18	477.1623	814.0959
ENSMUSG(	336.5119	371.2261	298.9451	558.0421	579.4035	574.7727	335.561	570.7394
ENSMUSG(	113.1546	78.6496	103.3391	149.1494	162.4311	190.2487	98.38108	167.2764
ENSMUSG(	1066.605	605.0776	1309.269	1614.264	1740.191	1692.107	993.6505	1682.187
ENSMUSG(	392.5972	167.7858	399.5162	531.662	531.8627	561.6868	319.9664	541.7372
ENSMUSG(	293.2179	355.4962	310.9398	530.6473	554.6427	534.5084	319.8847	539.9328
ENSMUSG(	463.4418	327.1823	533.3033	709.2208	791.3563	734.8232	441.3091	745.1334
ENSMUSG(	3413.332	3502.529	3473.853	5686.957	5777.198	5953.075	3463.238	5805.743
ENSMUSG(	74.78042	45.09244	69.20026	96.3891	106.9668	113.7466	63.02437	105.7008
ENSMUSG(	4024.367	2402.483	4258.123	5810.741	5922.792	6109.099	3561.658	5947.544
ENSMUSG(	305.0254	236.9975	303.5585	455.5653	469.4654	485.1846	281.8604	470.0718
ENSMUSG(	1151.225	1263.637	1101.668	2048.522	1908.565	1892.421	1172.177	1949.836
ENSMUSG(	610.0508	418.4159	623.725	835.034	891.3901	1024.726	550.7305	917.05
ENSMUSG(	267.6352	311.4524	250.9663	471.7993	477.3889	429.8212	276.6846	459.6698
ENSMUSG(	86.58785	124.7907	119.0244	199.8805	173.3258	176.1562	110.1343	183.1209
ENSMUSG(	55.10136	52.43306	52.5922	91.31599	91.11987	83.54839	53.37554	88.66142
ENSMUSG(	130.8657	121.6447	121.7925	217.1291	206.0101	198.3016	124.7676	207.147
ENSMUSG(	66.90879	57.67637	53.51487	117.6962	87.15814	90.59464	59.36668	98.48298
ENSMUSG(	151.5287	128.9853	168.8486	258.7286	249.5892	236.5527	149.7876	248.2902
ENSMUSG(	110.2027	99.62282	133.7872	176.5442	192.1441	200.3148	114.5376	189.6677
ENSMUSG(	382.7577	320.8904	426.2736	579.3492	662.5999	627.1162	376.6405	623.0218

ENSMUSG(	6212.678	6479.678	5970.598	10500.32	10290.6	10031.85	6220.985	10274.26
ENSMUSG(	173.1757	120.596	168.8486	249.597	253.5509	261.7179	154.2068	254.9553
ENSMUSG(	113.1546	63.96834	99.64837	148.1348	162.4311	146.9646	92.2571	152.5102
ENSMUSG(	4722.974	3314.818	5148.499	7171.349	7292.561	7239.518	4395.43	7234.476
ENSMUSG(	226.3092	228.6082	224.2088	348.0154	409.049	356.3389	226.3754	371.1344
ENSMUSG(	572.6606	479.2382	647.7144	934.4669	923.0839	931.1116	566.5377	929.5541
ENSMUSG(	201.7103	206.5863	230.6675	352.0739	345.6613	349.2927	212.988	349.0093
ENSMUSG(	1230.925	702.6031	1102.591	1471.202	1701.565	1795.787	1012.04	1656.185
ENSMUSG(	368.9823	367.0315	380.1401	642.2558	600.2026	580.8123	372.0513	607.7569
ENSMUSG(	740.9165	612.4182	843.3205	1152.611	1258.841	1176.724	732.2184	1196.058
ENSMUSG(	1987.585	1706.172	2106.456	3126.051	3205.042	3136.588	1933.404	3155.894
ENSMUSG(	431.9553	453.0217	418.8922	689.943	696.2747	733.8166	434.6231	706.6781
ENSMUSG(	778.3067	372.2748	793.4963	1001.432	1134.046	1026.739	648.0259	1054.072
ENSMUSG(	5261.196	4416.961	5111.592	7807.517	8104.716	8078.022	4929.917	7996.752
ENSMUSG(	373.9021	455.119	383.8308	619.9341	643.7817	696.5721	404.284	653.4293
novel.46	1028.231	1017.201	1114.586	478.9016	474.4176	459.0128	1053.339	470.7774
ENSMUSG(	12236.44	7316.51	11418.04	15453.71	16846.28	17721.32	10323.66	16673.77
ENSMUSG(	449.6664	330.3283	467.7938	699.0746	691.3225	626.1096	415.9295	672.1689
ENSMUSG(	5346.8	5167.803	5145.731	8544.133	8314.688	8415.236	5220.111	8424.685
ENSMUSG(	261.7315	173.0291	240.8169	329.7522	397.1638	363.3852	225.1925	363.4337
ENSMUSG(	4314.633	3378.787	4390.987	6367.768	6354.621	6654.68	4028.136	6459.023
ENSMUSG(	20208.42	19070.95	19450.81	30911.48	31459.14	31519.89	19576.73	31296.83
ENSMUSG(	1959.05	1048.661	2121.219	2545.687	2816.793	2818.5	1709.643	2726.993
ENSMUSG(	71286.4	63808.94	72328.11	107618.9	109983.7	113145.7	69141.15	110249.4
ENSMUSG(	813.729	553.6932	834.0938	1206.386	1153.855	1145.519	733.8387	1168.586
ENSMUSG(	633.6656	433.0971	660.6318	899.9698	889.4092	952.2503	575.7982	913.8764
ENSMUSG(	1984.633	1397.866	1909.927	2754.699	2911.874	2713.813	1764.142	2793.462
ENSMUSG(	181.0473	216.0242	183.6114	293.2258	320.9004	302.9887	193.561	305.705
ENSMUSG(	487.0567	438.3404	569.2875	834.0193	762.6337	760.995	498.2282	785.8827
ENSMUSG(	105.283	75.50361	103.3391	171.4711	145.5937	129.8523	94.70854	148.9724
ENSMUSG(	2640.929	1574.041	2733.872	3506.534	3761.666	3641.905	2316.281	3636.702
ENSMUSG(	5354.671	4773.506	5560.933	8248.877	8123.535	8257.198	5229.703	8209.87
ENSMUSG(	131.8497	104.8661	106.1071	172.4858	175.3067	189.2421	114.2743	179.0115
ENSMUSG(	180.0634	169.8831	185.4567	266.8456	279.3022	292.9227	178.4677	279.6902
ENSMUSG(	179.0794	176.1751	167.926	263.8017	258.5031	295.9425	174.3935	272.7491
ENSMUSG(	519.5271	384.8587	506.5459	754.8788	712.1216	739.8562	470.3106	735.6189
ENSMUSG(	13590.36	13666.15	13914.79	21983.82	21472.6	20867.97	13723.77	21441.46
ENSMUSG(	105.283	118.4987	107.9524	178.5735	155.498	183.2025	110.578	172.4247
ENSMUSG(	162.3522	104.8661	158.6993	216.1145	218.8858	228.4998	141.9725	221.1667
ENSMUSG(	2432.331	1732.388	2550.26	3374.633	3443.737	3605.667	2238.327	3474.679
ENSMUSG(	1629.426	1733.437	1634.049	2600.476	2547.395	2603.086	1665.637	2583.652
ENSMUSG(	176.1276	225.4622	192.8381	289.1673	317.9291	312.0482	198.1426	306.3815
ENSMUSG(	155.4645	159.3965	174.3847	249.597	247.6083	259.7046	163.0819	252.3033
ENSMUSG(	2511.048	2850.261	2557.642	3999.64	4080.586	4157.287	2639.65	4079.171
ENSMUSG(	106.2669	87.03889	146.7045	178.5735	160.4502	187.2289	113.3368	175.4175
ENSMUSG(	177.1115	133.18	144.8592	218.1437	219.8762	264.7377	151.7169	234.2525
ENSMUSG(	587.4198	356.5448	560.9834	727.484	756.6911	839.5103	501.6494	774.5618
ENSMUSG(	147.5929	144.7153	171.6166	218.1437	225.8188	271.7839	154.6416	238.5822
ENSMUSG(	376.8539	375.4207	393.0575	549.9252	616.0496	594.9048	381.7774	586.9598
ENSMUSG(	173.1757	114.3041	184.534	258.7286	244.637	221.4536	157.3379	241.6064

ENSMUSG(	550.0296	428.9025	583.1275	784.3029	845.8301	765.0214	520.6865	798.3848
ENSMUSG(	374.886	412.1239	429.0416	635.1534	596.2409	630.1361	405.3505	620.5101
ENSMUSG(	1207.31	879.8268	1238.223	1636.585	1753.067	1692.107	1108.453	1693.92
ENSMUSG(	198.7585	121.6447	149.4726	238.4362	216.9049	262.7245	156.6252	239.3552
ENSMUSG(	872.7662	615.5642	865.4646	1183.049	1244.975	1164.644	784.5983	1197.556
ENSMUSG(	353.2391	239.0948	360.764	457.5946	564.547	432.8411	317.6993	484.9942
ENSMUSG(	919.9959	656.462	975.2623	1221.605	1330.152	1338.787	850.5734	1296.848
ENSMUSG(	855.055	687.9218	763.9709	1073.47	1184.558	1244.166	768.9826	1167.398
ENSMUSG(	833.4081	795.9339	918.0568	1373.798	1232.099	1257.252	849.1329	1287.717
ENSMUSG(	607.0989	521.1847	634.797	851.2679	905.2561	917.0191	587.6935	891.181
ENSMUSG(	597.2594	395.3453	664.3225	814.7415	819.0884	874.7416	552.3091	836.1905
ENSMUSG(	5046.694	6027.705	5165.107	8277.287	8010.625	8252.165	5413.169	8180.026
ENSMUSG(	301.0896	184.5644	268.497	379.4687	384.2882	376.4711	251.3837	380.076
ENSMUSG(	1116.786	989.9363	1111.818	1549.328	1628.273	1682.041	1072.847	1619.88
ENSMUSG(	169.2399	169.8831	134.7098	255.6848	224.8284	233.5329	157.9443	238.0153
ENSMUSG(	1552.678	1497.488	1448.592	2254.49	2255.217	2276.945	1499.586	2262.217
ENSMUSG(	5014.224	3815.03	4784.045	6616.351	6968.689	6952.635	4537.766	6845.892
ENSMUSG(	5337.944	3849.636	5789.755	7422.975	7585.729	7584.785	4992.445	7531.163
ENSMUSG(	688.767	526.428	773.1976	986.2127	1017.175	996.5411	662.7975	999.9763
ENSMUSG(	3708.518	2235.746	3604.872	4604.355	4902.645	4890.097	3183.045	4799.033
ENSMUSG(	893.4292	870.3889	936.5102	1333.213	1410.377	1327.715	900.1094	1357.102
ENSMUSG(	329.6242	274.7493	391.2121	498.1794	498.188	505.3168	331.8619	500.5614
ENSMUSG(	830.4562	828.4424	858.0832	1348.433	1227.147	1211.955	838.9939	1262.512
ENSMUSG(	123.9781	118.4987	119.0244	182.632	181.2493	178.1695	120.5004	180.6836
ENSMUSG(	120.0422	122.6934	109.7977	168.4273	196.1058	163.0704	117.5111	175.8678
ENSMUSG(	4561.605	4160.039	4578.289	6637.658	6560.631	6682.865	4433.311	6627.051
ENSMUSG(	2116.483	2213.724	2309.443	3408.116	3216.928	3292.612	2213.217	3305.885
ENSMUSG(	692.7028	728.8196	704.92	1073.47	1023.118	1077.07	708.8141	1057.886
ENSMUSG(	981.001	985.7416	919.9021	1453.953	1442.071	1411.263	962.2149	1435.763
ENSMUSG(	1445.427	1079.072	1276.975	1989.674	1874.89	1806.86	1267.158	1890.475
ENSMUSG(	2316.225	1509.024	2551.183	3047.925	3189.196	3263.42	2125.477	3166.847
ENSMUSG(	111.1867	128.9853	125.4831	166.398	165.4024	212.3941	121.885	181.3982
ENSMUSG(	1867.542	1062.294	1963.442	2386.391	2447.361	2451.088	1631.093	2428.28
ENSMUSG(	203.6782	338.7176	232.5129	399.7611	375.3743	376.4711	258.3029	383.8688
ENSMUSG(	447.6985	391.1507	369.0681	593.5539	558.6044	642.2153	402.6391	598.1246
ENSMUSG(	790.1141	521.1847	706.7653	945.6278	1040.946	1010.634	672.688	999.0689
ENSMUSG(	1240.765	1058.099	1300.042	1813.13	1743.163	1787.734	1199.635	1781.342
ENSMUSG(	229.261	233.8515	244.5076	340.913	360.5178	348.2861	235.8734	349.9056
ENSMUSG(	528.3827	490.7735	559.1381	789.376	743.8155	807.2989	526.0981	780.1635
ENSMUSG(	236.1487	204.489	195.6061	303.372	308.0248	330.1671	212.0812	313.8546
ENSMUSG(	128.8978	103.8175	131.9418	171.4711	186.2015	182.1959	121.5524	179.9562
ENSMUSG(	135.7855	158.3479	165.158	220.173	241.6657	216.4205	153.0971	226.0864
ENSMUSG(	1166.968	996.2282	1240.991	1635.571	1651.052	1736.397	1134.729	1674.34
ENSMUSG(	396.533	432.0485	417.0469	638.1973	584.3557	614.0303	415.2094	612.1944
ENSMUSG(	4935.507	5769.734	4814.493	7641.119	7568.892	7668.333	5173.245	7626.115
ENSMUSG(	2658.641	1652.69	2535.498	3139.241	3317.952	3633.852	2282.276	3363.681
ENSMUSG(	2427.412	1495.391	2158.125	2692.807	3106.99	3122.495	2026.976	2974.097
ENSMUSG(	2001.36	1382.136	1893.319	2469.59	2632.572	2631.271	1758.938	2577.811
ENSMUSG(	1381.47	1383.184	1383.083	2016.054	1982.848	2059.518	1382.579	2019.473
ENSMUSG(	853.0871	662.7539	945.7369	1196.239	1153.855	1246.18	820.526	1198.758

ENSMUSG(	269.6031	239.0948	223.2862	391.6441	363.4891	312.0482	243.9947	355.7271
ENSMUSG(	1286.026	941.6978	1154.26	1594.986	1731.278	1601.512	1127.328	1642.592
ENSMUSG(	293.2179	255.8734	341.3879	420.0535	461.542	416.7353	296.8264	432.777
ENSMUSG(	264.6833	179.3211	291.5638	372.3663	355.5656	342.2464	245.1894	356.7261
ENSMUSG(	1610.731	1525.802	1701.404	2454.371	2316.624	2254.8	1612.646	2341.931
ENSMUSG(	1382.454	1517.413	1411.685	2095.195	2037.321	2117.901	1437.184	2083.472
ENSMUSG(	562.821	472.9462	634.797	762.9958	789.3754	870.7152	556.8548	807.6955
ENSMUSG(	185.9671	234.9001	203.9101	299.3135	310.9961	293.9293	208.2591	301.413
ENSMUSG(	4428.772	3868.512	4289.493	6137.449	5991.132	6091.986	4195.592	6073.522
ENSMUSG(	1482.817	1544.678	1428.293	2187.525	2134.384	2125.954	1485.263	2149.288
ENSMUSG(	1351.951	889.2648	1300.965	1699.492	1728.306	1695.126	1180.727	1707.642
ENSMUSG(	5040.79	3033.777	4595.82	5754.936	6298.166	6249.017	4223.462	6100.706
ENSMUSG(	1045.942	1053.905	1111.818	1566.576	1508.43	1556.215	1070.555	1543.74
ENSMUSG(	8722.742	6215.416	7898.056	11064.45	11083.94	10752.58	7612.071	10966.99
ENSMUSG(	340.4477	313.5497	344.156	474.8431	447.6759	515.3828	332.7178	479.3006
ENSMUSG(	240.0845	187.7104	244.5076	323.6644	302.0822	343.253	224.1008	322.9999
ENSMUSG(	4092.26	3954.502	4106.805	5854.369	5928.734	5701.423	4051.189	5828.175
ENSMUSG(	188.9189	173.0291	176.23	245.5385	286.2353	240.5791	179.3927	257.451
ENSMUSG(	2883.966	1935.829	3114.012	3782.511	3784.446	3821.081	2644.602	3796.013
ENSMUSG(	7524.287	8428.091	7884.216	11540.31	11317.68	11309.23	7945.531	11389.07
ENSMUSG(	116.1064	128.9853	133.7872	175.5296	167.3832	199.3082	126.293	180.7404
ENSMUSG(	292.234	224.4135	311.8625	363.2347	407.0681	415.7287	276.17	395.3439
ENSMUSG(	182.0313	139.472	163.3126	238.4362	229.7805	225.48	161.6053	231.2322
ENSMUSG(	2905.613	2218.967	2983.915	3731.78	3959.753	3897.583	2702.832	3863.039
ENSMUSG(	2275.883	1851.936	2354.654	3072.276	3098.076	3092.297	2160.824	3087.549
ENSMUSG(	5615.419	3409.198	5871.873	6880.152	7158.853	7239.518	4965.496	7092.841
ENSMUSG(	11736.59	11282.55	11804.64	16436.88	16487.74	16811.35	11607.93	16578.66
ENSMUSG(	559.8692	375.4207	531.458	630.0803	717.0738	747.9091	488.916	698.3544
ENSMUSG(	510.6715	350.2529	433.655	616.8902	638.8295	588.8652	431.5265	614.8616
ENSMUSG(	377.8379	376.4694	372.7587	499.1941	544.7384	559.6736	375.6887	534.5353
ENSMUSG(	1005.6	781.2527	1076.756	1331.184	1329.162	1416.296	954.5362	1358.881
novel.36	599.2273	268.4573	592.3542	135.9594	126.7755	112.74	486.6796	125.1583
ENSMUSG(	3486.145	3067.334	3543.976	4808.294	4703.568	4855.873	3365.818	4789.245
ENSMUSG(	19962.44	16515.37	18817.86	25774.44	26824.9	26052	18431.89	26217.11
ENSMUSG(	490.0085	328.231	558.2154	652.402	647.7434	658.3211	458.8183	652.8222
ENSMUSG(	1669.768	1424.082	1777.985	2219.993	2426.562	2279.965	1623.945	2308.84
ENSMUSG(	1426.732	972.109	1402.459	1724.858	1853.101	1825.985	1267.1	1801.315
ENSMUSG(	4992.577	4255.468	4701.004	6497.64	6642.837	6677.832	4649.683	6606.103
ENSMUSG(	9403.637	6544.695	10170.59	12229.24	12283.35	12540.31	8706.308	12350.97
ENSMUSG(	368.9823	355.4962	393.0575	567.1737	473.4272	543.5678	372.512	528.0563
ENSMUSG(	2822.961	2902.694	2921.174	4260.398	4034.035	3947.913	2882.276	4080.782
ENSMUSG(	5625.258	4586.845	5843.27	7503.13	7507.485	7677.393	5351.791	7562.669
ENSMUSG(	2976.457	1949.461	3220.119	3692.21	3860.709	3939.86	2715.346	3830.926
ENSMUSG(	480.169	392.1993	418.8922	646.3143	583.3653	590.8784	430.4202	606.8526
ENSMUSG(	1463.138	1050.759	1420.912	1750.223	1852.11	1940.739	1311.603	1847.691
ENSMUSG(	286.3303	232.8028	310.0172	381.4979	414.9916	370.4314	276.3834	388.9736
ENSMUSG(	415.2281	284.1872	370.9134	513.3988	454.6089	535.515	356.7762	501.1742
ENSMUSG(	4371.702	4271.197	4359.616	6090.776	6104.041	6024.544	4334.172	6073.12
ENSMUSG(	262.7154	269.506	271.265	385.5564	370.4221	369.4248	267.8288	375.1344
ENSMUSG(	8362.615	7141.383	8715.542	11353.62	11365.22	11159.25	8073.18	11292.7

ENSMUSG(	9930.052	9013.244	9998.976	13430.55	13487.72	13448.27	9647.424	13455.52
ENSMUSG(	1641.233	1509.024	1718.934	2242.315	2297.805	2245.74	1623.064	2261.954
ENSMUSG(	210.5659	211.8296	224.2088	328.7376	265.4362	307.0152	215.5348	300.3963
ENSMUSG(	6476.378	4483.027	6224.333	7789.254	7916.534	8231.026	5727.912	7978.938
ENSMUSG(	267.6352	217.0729	295.2544	370.3371	368.4412	348.2861	259.9875	362.3548
ENSMUSG(	1282.091	1277.269	1329.568	1799.94	1813.484	1797.8	1296.309	1803.741
ENSMUSG(	525.4308	428.9025	451.1857	614.861	674.4851	663.3541	468.5063	650.9001
ENSMUSG(	306.9933	260.068	247.2756	379.4687	404.0968	347.2795	271.4456	376.9483
ENSMUSG(	418.18	433.0971	472.4071	593.5539	661.6095	583.8321	441.2281	612.9985
ENSMUSG(	196.7906	214.9756	175.3073	283.0796	272.3692	258.698	195.6912	271.3823
ENSMUSG(	1185.663	1154.576	1157.951	1651.805	1615.397	1589.433	1166.063	1618.878
ENSMUSG(	192.8548	151.0072	190.07	255.6848	243.6466	242.5923	177.9773	247.3079
ENSMUSG(	1709.126	1811.038	1612.827	2441.181	2331.48	2342.375	1710.997	2371.679
ENSMUSG(	3438.915	2200.091	3295.778	4119.366	4089.499	4174.4	2978.261	4127.755
ENSMUSG(	481.1529	558.9365	529.6127	737.6303	729.9494	704.625	523.234	724.0682
ENSMUSG(	1219.118	809.5665	1345.253	1546.284	1582.713	1539.102	1124.646	1556.033
ENSMUSG(	6037.535	5594.608	5820.203	7751.713	7977.941	8391.077	5817.449	8040.244
ENSMUSG(	973.1294	1205.96	966.9583	1435.69	1483.669	1425.356	1048.683	1448.238
ENSMUSG(	856.039	690.0191	826.7124	1056.222	1082.544	1139.479	790.9235	1092.748
ENSMUSG(	2418.556	2231.551	2511.508	3245.776	3365.493	3275.5	2387.205	3295.589
ENSMUSG(	1196.487	845.221	1212.389	1444.822	1525.267	1517.964	1084.699	1496.018
ENSMUSG(	9459.723	6072.798	9555.172	10971.11	11722.77	11878.97	8362.564	11524.28
ENSMUSG(	442.7788	290.4792	400.4388	515.428	528.8914	515.3828	377.8989	519.9008
ENSMUSG(	276.4907	244.3381	280.4917	370.3371	359.5273	372.4446	267.1068	367.4363
ENSMUSG(	2455.946	2051.181	2541.034	3132.138	3302.105	3253.354	2349.387	3229.199
ENSMUSG(	9542.375	6714.578	9898.405	11534.22	12101.12	12285.64	8718.453	11973.66
ENSMUSG(	500.832	432.0485	520.386	655.4459	664.5808	674.4268	484.4221	664.8178
ENSMUSG(	21271.09	15793.89	22347.07	26510.05	27446.89	27483.39	19804.02	27146.78
ENSMUSG(	9055.318	10339.8	8969.276	12965.86	12782.53	13129.18	9454.798	12959.19
ENSMUSG(	1328.336	1280.415	1207.775	1749.208	1790.704	1690.093	1272.176	1743.335
ENSMUSG(	2815.089	2484.279	2667.439	3711.488	3710.164	3497.96	2655.602	3639.87
ENSMUSG(	810.7771	855.7076	715.992	1117.099	1127.113	1017.68	794.1589	1087.297
ENSMUSG(	276.4907	297.8198	290.6411	419.0389	362.4986	402.6428	288.3172	394.7268
ENSMUSG(	252.8759	292.5765	250.9663	380.4833	338.7282	369.4248	265.4729	362.8788
ENSMUSG(	5942.091	6412.564	5941.996	8542.103	8323.602	8141.438	6098.884	8335.715
ENSMUSG(	5043.742	5510.715	5056.232	7245.416	6929.072	7137.851	5203.563	7104.113
ENSMUSG(	1309.641	1432.471	1324.032	1906.475	1894.699	1744.45	1355.381	1848.541
ENSMUSG(	461.4739	397.4426	407.8202	571.2322	572.4705	583.8321	422.2456	575.845
ENSMUSG(	724.1893	575.7151	702.152	934.4669	887.4283	908.9662	667.3521	910.2872
ENSMUSG(	848.1673	704.7004	853.4699	1137.391	1054.812	1089.149	802.1125	1093.784
ENSMUSG(	4942.395	5922.839	4914.141	7282.957	7212.336	6997.933	5259.792	7164.409
ENSMUSG(	558.8852	481.3355	611.7303	713.2793	774.5189	764.0148	550.6503	750.6043
ENSMUSG(	2332.952	2520.982	2544.724	3478.124	3299.134	3277.513	2466.219	3351.59
ENSMUSG(	1034.134	931.2112	1100.745	1370.754	1437.119	1355.9	1022.03	1387.924
ENSMUSG(	2244.396	1589.771	2362.036	2754.699	2862.353	2782.262	2065.401	2799.771
ENSMUSG(	5616.403	3736.38	5804.518	6796.953	6923.129	6812.717	5052.434	6844.267
ENSMUSG(	789.1302	591.445	762.1255	967.9495	995.3856	940.1711	714.2336	967.8354
ENSMUSG(	2988.265	2178.07	3237.649	3676.99	3885.47	3816.048	2801.328	3792.836
ENSMUSG(	298.1377	309.3551	315.5532	447.4483	371.4125	430.8278	307.682	416.5629
ENSMUSG(	1774.067	1647.447	1706.017	2295.075	2340.394	2302.11	1709.177	2312.527

ENSMUSG(	1449.363	1158.771	1432.907	1777.618	1843.197	1846.117	1347.013	1822.311
ENSMUSG(	448.6825	423.6592	396.7482	604.7148	524.9297	585.8453	423.0299	571.8299
ENSMUSG(	2234.557	2061.668	2112.915	2922.112	2803.917	2939.293	2136.38	2888.44
ENSMUSG(	5603.611	5540.078	5802.672	7621.841	7807.586	7475.064	5648.787	7634.831
ENSMUSG(	1033.15	1091.656	1098.9	1453.953	1468.813	1420.323	1074.569	1447.696
ENSMUSG(	1050.862	1168.209	1055.535	1505.699	1398.492	1503.871	1091.535	1469.354
ENSMUSG(	2598.619	2140.318	2470.911	3105.758	3252.583	3345.962	2403.283	3234.768
ENSMUSG(	381.7737	423.6592	354.3053	513.3988	506.1115	539.5414	386.5794	519.6839
ENSMUSG(	2003.328	1460.785	1973.591	2337.689	2479.055	2500.412	1812.568	2439.052
ENSMUSG(	18714.78	15747.75	21264.78	24437.17	25009.43	25521.52	18575.77	24989.37
ENSMUSG(	241.0684	223.3649	242.6622	294.2404	337.7378	317.0812	235.6985	316.3531
ENSMUSG(	6488.185	5617.679	6579.561	8281.345	8413.732	8369.938	6228.475	8355.005
ENSMUSG(	450.6504	410.0266	429.9643	574.2761	583.3653	572.7595	430.2137	576.8003
ENSMUSG(	17180.8	12811.5	16663.42	20133.15	20851.59	21555.48	15551.91	20846.74
ENSMUSG(	719.2695	591.445	812.8724	953.7448	936.95	955.2702	707.8623	948.655
ENSMUSG(	7471.154	6721.919	7653.549	9832.703	9625.032	9772.142	7282.207	9743.292
ENSMUSG(	24438.44	24744.21	21981.69	33274.53	31402.68	30499.19	23721.45	31725.47
ENSMUSG(	309.9451	352.3502	312.7852	466.7262	434.8003	401.6362	325.0268	434.3876
ENSMUSG(	1634.346	1636.96	1654.348	2191.584	2219.561	2174.271	1641.884	2195.139
ENSMUSG(	761.5795	861.9996	797.187	1069.412	1121.171	1044.858	806.922	1078.48
ENSMUSG(	615.9545	611.3695	594.1996	734.5864	805.2224	886.8209	607.1745	808.8765
ENSMUSG(	13267.62	12378.4	13186.8	17275.97	17395.97	17067.02	12944.27	17246.32
ENSMUSG(	1106.947	835.7831	1207.775	1321.038	1416.32	1458.574	1050.168	1398.644
ENSMUSG(	2213.894	2084.739	2150.744	2852.103	2931.683	2797.361	2149.792	2860.382
ENSMUSG(	233.1968	235.9488	226.0542	297.2843	335.7569	290.9095	231.7333	307.9835
ENSMUSG(	516.5752	516.99	470.5618	679.7968	660.6191	658.3211	501.3757	666.2456
ENSMUSG(	1806.537	1610.744	1844.418	2291.017	2330.49	2374.586	1753.9	2332.031
ENSMUSG(	1305.705	906.0434	1271.439	1529.035	1511.401	1590.439	1161.063	1543.625
ENSMUSG(	705.4942	854.659	795.3416	1081.587	947.8448	1100.222	785.1649	1043.218
ENSMUSG(	429.0034	529.574	430.8869	588.4808	636.8487	619.0634	463.1548	614.7976
ENSMUSG(	7963.13	6112.647	8257.898	9886.478	9813.214	9958.364	7444.558	9886.019
ENSMUSG(	3894.485	4488.27	3989.626	5474.901	5361.216	5590.696	4124.127	5475.604
ENSMUSG(	2693.079	2447.575	2649.909	3458.847	3414.024	3459.709	2596.854	3444.193
ENSMUSG(	895.3971	721.479	882.0726	1111.011	1107.305	1097.202	832.9829	1105.172
ENSMUSG(	6892.59	5700.523	6670.905	8490.358	8496.928	8547.101	6421.339	8511.462
ENSMUSG(	2287.69	1630.668	2273.459	2738.465	2761.328	2707.773	2063.939	2735.855
ENSMUSG(	1085.3	898.7027	1049.999	1335.243	1358.875	1325.702	1011.334	1339.94
ENSMUSG(	5947.995	4438.983	6363.656	7236.285	7321.284	7616.996	5583.545	7391.521
ENSMUSG(	2713.742	2298.666	2729.258	3493.344	3341.722	3405.352	2580.555	3413.473
ENSMUSG(	8901.821	6291.968	8867.783	10525.69	10744.22	10537.16	8020.524	10602.36
ENSMUSG(	850.1353	699.4571	863.6192	1057.236	1098.391	1035.799	804.4039	1063.809
ENSMUSG(	425.0676	405.8319	451.1857	531.662	573.4609	586.852	427.3617	563.9916
ENSMUSG(	2805.25	2273.498	2924.864	3479.139	3386.292	3693.242	2667.871	3519.557
ENSMUSG(	893.4292	682.6785	846.0885	999.4027	1075.611	1120.354	807.3987	1065.122
ENSMUSG(	6477.362	5270.572	6365.501	7869.409	7923.467	8075.002	6037.811	7955.959
ENSMUSG(	2727.517	3298.04	2666.517	3887.017	3715.116	3843.226	2897.358	3815.12
ENSMUSG(	1227.973	906.0434	1274.207	1478.304	1519.325	1487.765	1136.075	1495.132
ENSMUSG(	695.6547	566.2771	664.3225	849.2387	800.2702	883.8011	642.0847	844.4366
ENSMUSG(	26160.35	18451.2	25943.64	30621.29	31296.7	30770.97	23518.4	30896.32
ENSMUSG(	6605.275	5548.467	6196.653	7886.657	8082.927	8071.983	6116.798	8013.856

ENSMUSG(	1146.305	839.9777	1092.441	1353.506	1340.056	1339.794	1026.241	1344.452
ENSMUSG(	527.3987	420.5132	512.0819	602.6855	641.8008	668.3871	486.6646	637.6245
ENSMUSG(	1874.43	1688.345	1840.727	2329.572	2281.959	2431.963	1801.167	2347.831
ENSMUSG(	1485.769	1095.851	1497.494	1703.55	1820.417	1786.728	1359.704	1770.232
ENSMUSG(	1368.678	968.963	1228.997	1496.568	1575.78	1570.307	1188.879	1547.551
ENSMUSG(	1069.557	1019.299	1176.404	1419.456	1442.071	1387.105	1088.42	1416.211
ENSMUSG(	5168.704	5088.105	5746.39	6798.983	6839.933	7144.897	5334.399	6927.938
ENSMUSG(	6675.136	6647.464	6274.157	8352.369	8410.76	8663.868	6532.252	8475.666
ENSMUSG(	2462.834	2123.539	2768.01	3115.904	3147.597	3281.539	2451.461	3181.68
ENSMUSG(	847.1834	761.3281	871.0006	1097.821	1068.678	1048.885	826.504	1071.794
ENSMUSG(	2483.497	2590.193	2400.788	3305.639	3154.53	3219.13	2491.493	3226.433
ENSMUSG(	1875.414	1503.78	1788.135	2145.926	2265.121	2279.965	1722.443	2230.337
ENSMUSG(	1011.504	849.4157	955.8862	1208.415	1234.08	1204.909	938.9351	1215.801
ENSMUSG(	1882.302	1834.109	1914.541	2536.555	2436.466	2309.157	1876.984	2427.393
ENSMUSG(	2411.668	1872.909	2435.849	2839.927	2827.687	3022.841	2240.142	2896.819
ENSMUSG(	1532.015	1240.566	1511.334	1792.837	1838.244	1907.52	1427.972	1846.201
ENSMUSG(	1163.032	1010.909	1079.524	1261.175	1409.387	1530.043	1084.489	1400.202
ENSMUSG(	1343.096	1119.97	1443.979	1712.682	1627.282	1702.173	1302.348	1680.712
ENSMUSG(	3028.607	2604.875	2989.451	3734.824	3642.814	3745.585	2874.311	3707.741
ENSMUSG(	5721.686	5275.815	5885.713	7225.124	7245.02	7281.796	5627.738	7250.647
ENSMUSG(	678.9275	693.1651	712.3013	871.5604	900.304	910.9794	694.798	894.2813
ENSMUSG(	8901.821	7647.887	8465.498	10497.28	10815.53	10874.38	8338.402	10729.06
ENSMUSG(	1854.751	2086.836	1885.938	2617.725	2489.95	2386.665	1942.508	2498.113
ENSMUSG(	5794.498	5594.608	5827.585	7295.133	7451.03	7390.509	5738.897	7378.891
ENSMUSG(	2665.528	3453.242	2621.306	3743.955	3707.192	3760.684	2913.359	3737.277
ENSMUSG(	890.4773	802.2259	881.15	1112.026	1114.238	1073.043	857.9511	1099.769
ENSMUSG(	4266.42	3916.75	4307.947	5338.941	5196.804	5466.883	4163.705	5334.21
ENSMUSG(	969.1935	1121.019	1151.492	1340.316	1418.301	1392.138	1080.568	1383.585
ENSMUSG(	2409.7	2173.875	2330.665	2981.974	2973.281	2898.022	2304.747	2951.092
ENSMUSG(	519.5271	435.1944	497.3192	581.3785	659.6286	618.0568	484.0136	619.688
ENSMUSG(	6646.601	5318.81	6304.605	7642.133	7828.386	7905.892	6090.005	7792.137
ENSMUSG(	2907.581	2303.909	2876.885	3328.975	3501.182	3511.046	2696.125	3447.068
ENSMUSG(	2779.667	2764.271	2822.448	3570.455	3517.029	3607.68	2788.795	3565.055
ENSMUSG(	658.2644	698.4084	631.1064	792.4198	878.5144	867.6953	662.5931	846.2099
ENSMUSG(	1611.715	1765.946	1684.796	2136.794	2230.456	2099.782	1687.485	2155.678
ENSMUSG(	8524.967	8451.161	8348.319	10774.27	10660.03	10906.59	8441.483	10780.3
ENSMUSG(	19435.04	22148.78	19150.94	26213.78	25737.4	25589.97	20244.92	25847.05
ENSMUSG(	803.8895	640.7321	780.5789	935.4816	950.8161	955.2702	741.7335	947.1893
ENSMUSG(	2943.987	2553.49	3154.609	3680.034	3701.25	3658.01	2884.029	3679.765
ENSMUSG(	1025.279	847.3183	1098.9	1284.512	1253.889	1252.219	990.4991	1263.54
ENSMUSG(	1319.481	1235.323	1285.279	1597.015	1659.966	1638.756	1280.028	1631.913
ENSMUSG(	775.3548	735.1116	684.6212	909.1014	933.9787	953.2569	731.6959	932.1123
ENSMUSG(	13253.84	11485.99	12737.46	15623.15	16144.06	15938.62	12492.43	15901.94
ENSMUSG(	3036.478	3121.865	3342.834	4064.576	3957.772	4070.719	3167.059	4031.022
ENSMUSG(	2016.119	1812.087	2004.962	2438.137	2494.902	2489.339	1944.389	2474.126
ENSMUSG(	459.506	479.2382	468.7164	593.5539	634.8678	561.6868	469.1535	596.7028
ENSMUSG(	1458.218	1760.702	1535.323	2128.677	2023.455	1890.408	1584.748	2014.18
ENSMUSG(	23701.46	21457.71	21987.23	28930.93	28468.03	27952.47	22382.13	28450.48
ENSMUSG(	3312.969	3032.728	3406.498	4115.307	3999.37	4283.113	3250.732	4132.597
ENSMUSG(	998.7121	789.642	962.3449	1133.333	1161.778	1201.889	916.8997	1165.667

ENSMUSG(	1931.499	1538.386	1810.279	2160.13	2245.313	2296.071	1760.055	2233.838
ENSMUSG(	1594.004	1792.162	1658.038	2171.291	2106.652	2120.921	1681.401	2132.955
novel.101	582.5001	519.0873	540.6847	358.1616	333.7761	321.1077	547.424	337.6818
ENSMUSG(	5939.139	6557.279	5798.059	7864.336	7757.074	7584.785	6098.159	7735.398
ENSMUSG(	27858.66	27642.71	27025.93	35163.76	34376.95	35071.2	27509.1	34870.64
ENSMUSG(	4103.083	3462.68	4223.984	4834.674	4968.014	5108.531	3929.916	4970.406
ENSMUSG(	981.9849	983.6443	1022.318	1299.731	1213.281	1264.299	995.9826	1259.103
ENSMUSG(	4103.083	3735.332	4125.258	4974.692	5074.981	5069.273	3987.891	5039.649
ENSMUSG(	1010.52	839.9777	1027.855	1218.561	1195.453	1222.021	959.4506	1212.012
ENSMUSG(	351939.3	359071.1	350847.2	454664.3	441331.2	444632.5	353952.5	446876
ENSMUSG(	832.4241	811.6638	888.5313	1079.558	1093.438	1020.7	844.2064	1064.565
ENSMUSG(	8063.494	9536.526	7994.014	11253.17	10503.55	10477.77	8531.344	10744.83
ENSMUSG(	7451.475	7953.047	7670.157	9979.823	9504.199	9566.794	7691.56	9683.605
ENSMUSG(	5991.289	7435.009	6090.545	8417.305	8020.53	8120.3	6505.614	8186.045
ENSMUSG(	764.5314	730.9169	791.651	926.35	965.6726	984.4618	762.3664	958.8281
ENSMUSG(	2123.37	1743.924	2236.552	2433.064	2657.333	2573.894	2034.615	2554.764
ENSMUSG(	4730.845	5543.224	4610.583	6496.625	6164.457	6017.497	4961.55	6226.193
ENSMUSG(	5447.163	5078.667	5452.058	6806.085	6564.593	6668.772	5325.962	6679.817
ENSMUSG(	875.718	812.7125	836.8618	1054.192	1025.099	1086.129	841.7641	1055.14
ENSMUSG(	28696	23332.71	26948.43	33599.21	33189.42	32134.93	26325.71	32974.52
ENSMUSG(	2193.231	1816.281	2350.963	2626.857	2645.448	2694.687	2120.159	2655.664
ENSMUSG(	49271.44	45364.04	48137.55	59689.2	59914.29	59150.25	47591.01	59584.58
ENSMUSG(	2514.983	2454.916	2379.566	2981.974	3102.037	3112.429	2449.822	3065.48
ENSMUSG(	2258.172	1952.607	2313.134	2734.406	2656.342	2764.143	2174.638	2718.297
ENSMUSG(	1374.582	1364.308	1288.047	1718.77	1662.938	1646.809	1342.313	1676.172
ENSMUSG(	2615.347	2169.68	2778.16	3144.314	3215.937	3085.251	2521.062	3148.501
ENSMUSG(	1389.341	1155.625	1305.578	1621.366	1582.713	1599.499	1283.515	1601.192
ENSMUSG(	2310.321	2011.332	2484.751	2875.439	2848.486	2757.097	2268.801	2827.007
ENSMUSG(	982.9689	955.3304	914.3661	1184.064	1168.711	1198.869	950.8885	1183.881
ENSMUSG(	1133.514	957.4278	1144.111	1343.36	1278.65	1406.23	1078.351	1342.746
ENSMUSG(	1453.298	1420.936	1525.174	1846.612	1805.56	1823.972	1466.469	1825.381
ENSMUSG(	2743.261	2597.534	2636.069	3206.206	3311.019	3396.292	2658.954	3304.506
ENSMUSG(	1978.729	1936.877	2120.296	2438.137	2572.156	2491.353	2011.968	2500.548
ENSMUSG(	1675.672	1384.233	1572.23	1881.109	1940.259	1929.666	1544.045	1917.011
ENSMUSG(	175.1436	188.759	184.534	65.95044	97.06247	91.60125	182.8122	84.87139
ENSMUSG(	918.028	799.0799	939.2782	1112.026	1093.438	1088.142	885.462	1097.869
ENSMUSG(	647.441	625.0021	639.4104	789.376	763.6241	815.3518	637.2845	789.4506
ENSMUSG(	2803.282	2704.497	3025.435	3657.713	3461.565	3446.623	2844.405	3521.967
ENSMUSG(	12202.98	11463.97	12398.84	14810.44	15166.51	14631.03	12021.93	14869.33
ENSMUSG(	2175.52	1903.32	2350.963	2704.982	2683.084	2550.742	2143.268	2646.27
ENSMUSG(	3073.869	3529.794	3201.665	4239.091	3898.346	3959.992	3268.443	4032.476
ENSMUSG(	2567.133	2694.011	2655.445	3338.107	3100.057	3312.744	2638.863	3250.302
ENSMUSG(	970.1775	876.6808	921.7475	1125.216	1153.855	1131.426	922.8686	1136.832
ENSMUSG(	987.8886	956.3791	982.6437	1231.751	1181.587	1191.823	975.6371	1201.72
ENSMUSG(	1416.892	1260.491	1326.8	1651.805	1553	1727.338	1334.728	1644.047
ENSMUSG(	2953.826	2690.865	2941.472	3612.055	3443.737	3506.013	2862.055	3520.601
ENSMUSG(	3168.328	2818.802	3423.106	3797.73	3917.164	3859.332	3136.745	3858.075
ENSMUSG(	6519.671	6886.559	6529.736	8420.349	8175.037	7917.972	6645.322	8171.119
ENSMUSG(	1332.272	1205.96	1196.703	1509.758	1490.602	1584.4	1244.979	1528.253
ENSMUSG(	1397.213	1246.858	1443.979	1670.068	1657.986	1690.093	1362.683	1672.716

ENSMUSG(	2398.877	2417.164	2350.041	2947.477	2957.434	2882.923	2388.694	2929.278
ENSMUSG(	1028.231	977.3523	1031.545	1284.512	1246.956	1191.823	1012.376	1241.097
ENSMUSG(	1680.591	1520.559	1573.153	1885.168	1973.934	1993.082	1591.434	1950.728
ENSMUSG(	12613.29	10515.98	11294.41	13775.52	14363.27	13998.89	11474.56	14045.89
ENSMUSG(	1034.134	939.6005	1080.447	1455.983	1438.109	1478.706	1018.061	1457.599
ENSMUSG(	1302.754	1160.868	1302.81	1524.977	1521.306	1564.267	1255.477	1536.85
ENSMUSG(	11648.03	13958.73	11782.5	15718.53	15045.67	14979.32	12463.09	15247.84
ENSMUSG(	1411.972	1225.885	1261.29	1549.328	1564.885	1655.869	1299.716	1590.027
ENSMUSG(	1995.456	2023.916	1873.943	2389.435	2366.145	2447.062	1964.439	2400.881
ENSMUSG(	11257.4	11564.64	11327.62	14133.69	13684.82	13911.31	11383.22	13909.94
ENSMUSG(	2028.911	1882.347	1935.762	2367.113	2357.231	2418.877	1949.007	2381.074
ENSMUSG(	6293.362	6742.892	6434.701	7933.33	7827.395	8022.659	6490.319	7927.795
ENSMUSG(	16004.98	13982.85	16725.24	18882.12	19144.09	18992.66	15571.02	19006.29
ENSMUSG(	8002.488	6973.598	7852.845	9094.058	9591.357	9112.814	7609.644	9266.076
ENSMUSG(	2762.94	2571.318	2716.341	3200.118	3388.273	3200.004	2683.533	3262.798
ENSMUSG(	1247.652	1346.481	1298.197	1554.401	1639.167	1536.082	1297.443	1576.55
ENSMUSG(	1953.146	2208.481	2052.018	2504.087	2597.907	2443.035	2071.215	2515.01
ENSMUSG(	3641.609	3435.414	3693.449	4385.197	4326.213	4356.596	3590.157	4356.002
ENSMUSG(	3354.295	2862.845	3245.031	3812.95	3944.896	3713.374	3154.057	3823.74
ENSMUSG(	6697.767	5728.837	6512.206	7622.856	7696.658	7636.122	6312.936	7651.878
ENSMUSG(	11217.06	12733.89	11480.78	14686.65	14220.64	14029.08	11810.58	14312.13
ENSMUSG(	3621.93	3589.568	3719.283	4554.639	4304.424	4374.715	3643.594	4411.259
ENSMUSG(	16524.5	19576.41	16556.39	21643.92	21190.32	20910.25	17552.44	21248.16
ENSMUSG(	3354.295	3474.215	3139.846	4108.205	4047.901	3901.609	3322.786	4019.238
ENSMUSG(	943.6108	961.6224	961.4223	1171.888	1118.199	1177.73	955.5518	1155.939
ENSMUSG(	1156.145	1214.35	1161.642	1421.486	1427.215	1416.296	1177.379	1421.665
ENSMUSG(	1227.973	1246.858	1128.426	1434.676	1489.612	1424.349	1201.086	1449.546
ENSMUSG(	1101.043	1210.155	1149.647	1412.354	1370.76	1394.151	1153.615	1392.422
ENSMUSG(	4534.055	4093.974	4441.734	5124.856	5263.163	5390.381	4356.587	5259.467
ENSMUSG(	13086.57	12349.04	12719.93	15322.82	15465.62	15268.22	12718.51	15352.22
ENSMUSG(	7001.808	6402.077	7595.42	8415.276	8370.153	8508.85	6999.769	8431.426
ENSMUSG(	2777.699	3100.891	2898.107	3696.268	3488.306	3383.207	2925.566	3522.594
ENSMUSG(	1129.578	1211.204	1218.847	1416.412	1394.53	1472.666	1186.543	1427.87
ENSMUSG(	7506.576	6972.549	7720.904	8939.835	8653.417	9077.583	7400.01	8890.278
ENSMUSG(	2857.399	2936.252	2844.592	3430.437	3497.22	3395.286	2879.414	3440.981
ENSMUSG(	7684.672	8285.473	7545.596	9330.465	9484.39	9273.871	7838.58	9362.909
ENSMUSG(	3909.245	3510.918	3939.801	4663.203	4432.189	4448.197	3786.655	4514.53
ENSMUSG(	14361.78	15459.36	14060.57	17469.76	17520.77	17272.37	14627.24	17420.97
ENSMUSG(	3394.637	3442.755	3528.291	4101.102	4033.045	4164.334	3455.228	4099.494
ENSMUSG(	15464.79	15339.82	14958.33	17399.75	17947.64	18732.96	15254.31	18026.79
ENSMUSG(	11212.14	10483.47	11341.46	12606.68	13205.45	13191.59	11012.36	13001.24
ENSMUSG(	10140.62	10223.4	9561.631	11791.94	11879.26	11611.21	9975.216	11760.8
ENSMUSG(	20165.13	19693.86	20911.4	23715.78	23767.43	23921.01	20256.79	23801.41
ENSMUSG(	5704.959	5825.314	5899.553	6844.641	6946.9	6668.772	5809.942	6820.104
ENSMUSG(	18675.42	16974.68	17960.7	20424.34	21016.01	21438.72	17870.27	20959.69
ENSMUSG(	11931.41	12309.19	11542.6	14027.15	13819.52	13913.32	11927.73	13920
ENSMUSG(	8021.184	7675.152	7737.512	6677.228	6749.804	6648.64	7811.282	6691.891
ENSMUSG(	8179.6	8765.76	8319.717	7106.413	7363.872	7034.171	8421.692	7168.152
ENSMUSG(	4997.496	5120.613	5016.557	4325.334	4243.017	4311.298	5044.889	4293.216
ENSMUSG(	5682.328	6259.459	5693.797	5063.979	4959.1	4854.866	5878.528	4959.315

ENSMUSG(	7098.236	6970.452	6832.372	5815.814	5869.308	5933.949	6967.02	5873.024
ENSMUSG(	4374.654	4712.684	4175.082	3695.254	3697.288	3781.823	4420.807	3724.788
ENSMUSG(	2322.129	2380.461	2372.185	1924.738	1950.163	2085.69	2358.258	1986.864
ENSMUSG(	13144.63	12652.1	12788.21	11025.9	10563.96	10811.97	12861.64	10800.61
ENSMUSG(	5046.694	5307.275	4830.178	4208.652	4274.711	4207.618	5061.382	4230.327
ENSMUSG(	10027.46	10722.56	10126.3	8688.209	8404.818	8712.185	10292.11	8601.737
ENSMUSG(	1419.844	1483.856	1386.773	1166.815	1221.204	1188.803	1430.158	1192.274
ENSMUSG(	2252.268	2308.104	2282.686	1932.855	1886.776	1882.355	2281.019	1900.662
ENSMUSG(	7440.651	8025.405	7645.245	6464.157	6612.133	6165.469	7703.767	6413.92
ENSMUSG(	2082.044	2049.084	2019.725	1791.823	1658.976	1661.908	2050.284	1704.236
ENSMUSG(	11832.03	11532.13	12021.47	9607.456	9819.157	9972.457	11795.21	9799.69
ENSMUSG(	7976.906	7904.809	7805.789	6337.329	6479.415	6814.73	7895.835	6543.825
ENSMUSG(	9019.896	10101.75	9080.919	8125.094	7669.916	7533.448	9400.856	7776.153
ENSMUSG(	3354.295	3576.984	3420.338	2876.454	2802.927	2874.87	3450.539	2851.417
ENSMUSG(	1549.726	1750.216	1627.59	1347.418	1381.655	1337.781	1642.511	1355.618
ENSMUSG(	7392.438	8642.018	7468.092	6772.602	6366.506	6233.918	7834.182	6457.675
ENSMUSG(	3291.322	3215.196	3518.141	2773.977	2736.567	2741.998	3341.553	2750.847
ENSMUSG(	94393.55	110648.4	91741.09	83837.21	81446.31	78950.21	98927.7	81411.24
ENSMUSG(	2137.146	2083.69	2275.305	1810.086	1823.388	1712.239	2165.38	1781.904
ENSMUSG(	5234.629	6076.992	5148.499	4694.656	4435.161	4398.873	5486.707	4509.563
ENSMUSG(	5438.307	6448.218	5472.357	4716.978	4725.358	4807.556	5786.294	4749.964
ENSMUSG(	3464.498	3884.241	3508.914	2990.091	3051.525	2870.844	3619.218	2970.82
ENSMUSG(	4853.839	5141.586	4654.871	3967.172	3895.375	4155.274	4883.432	4005.94
ENSMUSG(	1352.935	1230.08	1311.114	1047.09	1114.238	1030.766	1298.043	1064.031
ENSMUSG(	1865.575	2071.106	1870.252	1581.796	1591.626	1580.373	1935.644	1584.598
ENSMUSG(	1422.796	1640.106	1449.515	1246.971	1255.87	1189.81	1504.139	1230.883
ENSMUSG(	1932.483	1780.627	2020.648	1532.079	1592.617	1563.261	1911.253	1562.652
ENSMUSG(	3892.517	4464.151	3918.58	3461.891	3325.875	3230.202	4091.75	3339.323
ENSMUSG(	18215.92	21122.14	18904.59	16192.35	15630.03	15592.34	19414.21	15804.91
ENSMUSG(	17793.8	15651.27	18261.49	14015.99	14020.58	14059.28	17235.52	14031.95
ENSMUSG(	55137.77	56195.66	57070.84	46469.69	45536.17	45075.87	56134.75	45693.91
ENSMUSG(	1262.411	1412.547	1212.389	1055.207	1061.745	1043.852	1295.782	1053.601
ENSMUSG(	1422.796	1421.985	1442.133	1186.093	1205.357	1091.162	1428.971	1160.871
ENSMUSG(	25524.72	30360.84	26416.05	22571.28	22500.67	21587.7	27433.87	22219.88
ENSMUSG(	2739.325	2774.758	2484.751	2132.736	2212.628	2115.888	2666.278	2153.751
ENSMUSG(	6666.28	6021.413	6336.898	4984.838	4995.746	5388.368	6341.531	5122.984
ENSMUSG(	20904.08	24874.25	21149.44	18603.1	17653.48	17775.68	22309.26	18010.75
ENSMUSG(	2599.603	2735.957	2303.907	2069.829	2033.36	2039.386	2546.489	2047.525
ENSMUSG(	4304.794	4998.968	4366.998	3634.376	3594.283	3732.499	4556.92	3653.719
ENSMUSG(	6428.164	6052.873	6168.05	4935.122	5037.344	4974.652	6216.362	4982.373
ENSMUSG(	1147.289	1189.182	1132.116	939.54	920.1126	919.0323	1156.196	926.2283
ENSMUSG(	1427.716	1433.52	1597.142	1246.971	1204.367	1119.347	1486.126	1190.228
ENSMUSG(	5479.633	6530.014	5551.706	4776.841	4752.099	4513.626	5853.784	4680.856
ENSMUSG(	903.2687	986.7903	958.6543	802.5661	738.8633	732.81	949.5711	758.0798
ENSMUSG(	9258.012	11567.78	9490.585	8318.886	8068.07	7815.298	10105.46	8067.418
ENSMUSG(	98750.49	119939.6	99154.75	87520.29	82133.67	84068.81	105948.3	84574.25
ENSMUSG(	777.3227	903.946	795.3416	662.5482	645.7626	667.3805	825.5368	658.5638
ENSMUSG(	1172.872	1394.72	1166.255	1044.046	994.3951	940.1711	1244.615	992.8708
ENSMUSG(	8763.084	10156.28	8801.35	7676.631	7301.475	7136.844	9240.24	7371.65
ENSMUSG(	1053.813	1146.187	1024.164	853.2972	809.1841	905.9464	1074.721	856.1426

ENSMUSG(	39634.6	37646.94	40678.68	31290.94	31337.31	31240.05	39320.08	31289.44
ENSMUSG(	4710.182	5757.151	4697.314	4149.804	4036.016	3876.444	5054.882	4020.755
ENSMUSG(	1040.038	1025.591	1049.999	854.3118	792.3467	827.4311	1038.543	824.6965
ENSMUSG(	2661.592	2953.03	2628.687	2245.359	2145.279	2149.106	2747.77	2179.915
ENSMUSG(	1063.653	1096.9	1014.937	844.1656	832.9545	839.5103	1058.497	838.8768
ENSMUSG(	2140.097	1789.016	2218.099	1610.205	1612.426	1641.776	2049.071	1621.469
ENSMUSG(	2582.876	2907.938	2573.327	2172.306	2127.451	2074.617	2688.047	2124.791
ENSMUSG(	3256.884	2939.398	3091.868	2381.318	2471.131	2490.346	3096.05	2447.598
ENSMUSG(	1111.867	1063.343	1101.668	905.0429	840.878	844.5434	1092.292	863.4881
ENSMUSG(	3227.365	2924.716	3293.932	2520.321	2504.806	2435.989	3148.671	2487.039
ENSMUSG(	1494.624	1793.211	1393.232	1253.058	1208.329	1233.094	1560.356	1231.494
ENSMUSG(	2281.787	2602.777	2446.921	1987.645	1858.053	1941.745	2443.828	1929.148
ENSMUSG(	14015.42	18071.58	13773.62	12753.8	11952.55	11485.39	15286.87	12063.91
ENSMUSG(	8882.142	11345.47	8836.412	7746.64	7650.108	7531.434	9688.007	7642.727
ENSMUSG(	928.8515	865.1456	993.7157	687.9138	743.8155	768.0412	929.2376	733.2568
ENSMUSG(	8935.276	10152.09	9333.731	7556.905	7438.155	7388.496	9473.699	7461.185
ENSMUSG(	1410.988	1802.649	1610.059	1280.453	1306.382	1210.948	1607.899	1265.928
ENSMUSG(	1262.411	1487.002	1277.898	1045.061	1098.391	1026.739	1342.437	1056.73
ENSMUSG(	1079.396	1201.766	1154.26	952.7301	866.6292	882.7944	1145.141	900.7179
ENSMUSG(	2544.502	3125.011	2574.25	2244.344	2167.068	2061.531	2747.921	2157.648
ENSMUSG(	1131.546	1253.15	1118.276	921.2768	901.2944	924.0653	1167.657	915.5455
ENSMUSG(	797.9858	997.2769	861.7739	680.8114	672.5043	729.7902	885.6788	694.3686
ENSMUSG(	594.3075	593.5423	576.6688	477.887	432.8194	473.1053	588.1729	461.2706
ENSMUSG(	3054.19	2796.78	3208.124	2395.523	2367.136	2332.309	3019.698	2364.989
ENSMUSG(	1803.586	2148.707	1718.934	1436.705	1498.526	1501.858	1890.409	1479.029
ENSMUSG(	8165.825	7352.164	8815.19	6444.879	6399.19	6200.7	8111.06	6348.256
ENSMUSG(	753.7079	819.0045	765.8162	650.3728	582.3748	593.8982	779.5095	608.8819
ENSMUSG(	29918.07	38741.74	30924.21	27267.97	25870.12	24625.64	33194.67	25921.24
ENSMUSG(	2045.638	2146.61	1912.695	1516.86	1677.794	1568.294	2034.981	1587.649
ENSMUSG(	6875.862	6484.921	7012.293	5314.59	5355.273	5228.317	6791.026	5299.394
ENSMUSG(	5955.867	7364.748	5697.488	5053.833	4987.823	4773.331	6339.368	4938.329
ENSMUSG(	1630.41	2093.128	1626.667	1389.018	1425.234	1346.84	1783.402	1387.031
ENSMUSG(	1584.164	1630.668	1494.726	1188.122	1244.975	1228.061	1569.853	1220.386
ENSMUSG(	1566.453	1374.795	1620.209	1211.459	1213.281	1122.367	1520.486	1182.369
ENSMUSG(	3678.016	4074.049	3827.236	3003.281	3124.817	2856.751	3859.767	2994.95
ENSMUSG(	652.3607	620.8075	619.1117	486.004	506.1115	476.1252	630.76	489.4135
ENSMUSG(	731.077	876.6808	775.0429	654.4312	570.4896	623.0898	794.2669	616.0036
ENSMUSG(	4192.623	5442.552	4474.95	3788.599	3655.69	3499.973	4703.375	3648.087
ENSMUSG(	748.7881	913.384	727.9867	615.8756	613.0783	622.0832	796.7196	617.0124
ENSMUSG(	2273.915	2954.079	2296.526	2055.624	1894.699	1878.329	2508.173	1942.884
ENSMUSG(	1976.761	2763.223	2161.816	1817.188	1780.799	1740.424	2300.6	1779.47
ENSMUSG(	9985.153	12527.31	9987.904	8640.522	8438.493	8034.738	10833.46	8371.251
ENSMUSG(	1434.603	1905.418	1477.195	1309.877	1209.319	1201.889	1605.739	1240.362
ENSMUSG(	479.185	571.5204	484.4018	383.5271	408.0586	393.5834	511.7024	395.0564
ENSMUSG(	932.7873	1150.381	946.6595	814.7415	772.5381	751.9355	1009.943	779.7384
ENSMUSG(	7559.71	8493.108	7695.069	6206.443	6102.06	6026.557	7915.962	6111.687
ENSMUSG(	1973.809	2325.931	2145.208	1638.615	1675.813	1659.895	2148.316	1658.108
ENSMUSG(	1962.986	1893.882	1904.391	1493.524	1403.444	1550.175	1920.42	1482.381
ENSMUSG(	1142.369	1337.043	1111.818	984.1834	887.4283	897.8936	1197.077	923.1684
ENSMUSG(	1106.947	885.0701	1007.556	773.142	799.2798	741.8694	999.8576	771.4304

ENSMUSG(	6670.216	7902.712	6622.926	5672.752	5325.56	5342.064	7065.285	5446.792
ENSMUSG(	654.3286	777.058	673.5492	553.9837	541.7671	524.4423	701.6453	540.0643
ENSMUSG(	2227.669	2944.641	2272.537	1941.987	1867.957	1917.587	2481.616	1909.177
ENSMUSG(	10518.46	12656.29	11018.53	8965.201	8848.532	8481.672	11397.76	8765.135
ENSMUSG(	3101.419	3572.789	3269.943	2612.652	2490.94	2534.637	3314.717	2546.076
ENSMUSG(	404.4046	477.1409	483.4791	359.1762	341.6995	347.2795	455.0082	349.3851
ENSMUSG(	1631.394	1914.856	1576.843	1346.403	1309.353	1276.378	1707.698	1310.711
ENSMUSG(	3868.903	4046.784	4084.661	3110.831	3061.43	3040.96	4000.116	3071.074
ENSMUSG(	4238.869	4321.533	4359.616	3282.302	3276.354	3349.989	4306.673	3302.882
ENSMUSG(	829.4722	1048.661	898.6807	680.8114	711.1312	736.8364	925.6047	709.593
ENSMUSG(	2037.766	2352.147	2231.016	1740.077	1649.072	1687.074	2206.977	1692.074
ENSMUSG(	2876.094	2350.05	2724.645	1967.352	1973.934	2155.146	2650.263	2032.144
ENSMUSG(	964.2738	1215.398	925.4381	786.3321	823.0502	768.0412	1035.037	792.4745
ENSMUSG(	1250.604	1662.128	1315.728	1101.88	1072.639	1060.964	1409.487	1078.494
ENSMUSG(	2402.813	3098.794	2598.239	2183.467	2045.245	1969.93	2699.949	2066.214
ENSMUSG(	1415.908	1713.513	1483.654	1200.298	1164.75	1163.638	1537.691	1176.228
ENSMUSG(	651.3768	828.4424	686.4666	595.5832	514.0349	544.5745	722.0953	551.3975
ENSMUSG(	1324.401	1621.23	1372.01	1118.114	1052.831	1125.387	1439.214	1098.777
ENSMUSG(	2810.169	2159.194	2786.464	1835.451	1967.991	2119.915	2585.276	1974.452
ENSMUSG(	734.0288	726.7223	688.3119	531.662	543.7479	564.7066	716.3543	546.7055
ENSMUSG(	1429.683	1545.727	1576.843	1182.035	1142.96	1148.539	1517.418	1157.845
ENSMUSG(	628.7459	602.9802	664.3225	461.653	501.1593	483.1714	632.0162	481.9946
ENSMUSG(	1948.227	2669.892	2017.88	1675.141	1739.201	1639.763	2211.999	1684.702
ENSMUSG(	5526.863	6924.311	5378.244	4588.121	4472.797	4517.653	5943.139	4526.19
ENSMUSG(	1279.139	1705.123	1259.445	1149.567	1085.515	993.5212	1414.569	1076.201
ENSMUSG(	671.0558	706.7977	617.2663	518.4719	445.695	553.6339	665.04	505.9336
ENSMUSG(	2701.934	2743.298	2684.97	2060.697	2060.101	2057.505	2710.068	2059.435
ENSMUSG(	1149.257	925.9679	1174.559	807.6392	862.6675	800.2527	1083.261	823.5198
ENSMUSG(	585.4519	606.1262	545.298	410.9219	416.9725	384.5239	578.9587	404.1394
ENSMUSG(	1244.7	982.5956	1302.81	856.341	902.2848	924.0653	1176.702	894.2304
ENSMUSG(	386.6935	410.0266	394.9028	311.489	284.2544	309.0284	397.2076	301.5906
ENSMUSG(	2365.423	2393.045	2406.324	1806.027	1829.33	1800.82	2388.264	1812.059
ENSMUSG(	521.495	477.1409	536.994	394.688	387.2595	383.5173	511.8766	388.4883
ENSMUSG(	583.484	772.8634	617.2663	516.4426	480.3602	499.2771	657.8712	498.6933
ENSMUSG(	4354.975	6279.384	4833.869	4086.898	3880.518	3752.631	5156.076	3906.682
ENSMUSG(	3401.525	4723.17	3639.934	3165.621	2924.75	2812.46	3921.543	2967.61
ENSMUSG(	704.5102	710.9924	733.5227	556.0129	516.0158	554.6405	716.3418	542.2231
ENSMUSG(	798.9697	954.2818	858.0832	647.3289	660.6191	665.3673	870.4449	657.7718
ENSMUSG(	1600.891	2055.376	1603.601	1370.754	1345.999	1256.246	1753.289	1324.333
ENSMUSG(	2288.674	2933.106	2289.145	1963.294	1884.795	1821.959	2503.642	1890.016
ENSMUSG(	663.1842	909.1893	760.2802	630.0803	572.4705	557.6603	777.5512	586.7371
ENSMUSG(	1555.629	1733.437	1510.411	1218.561	1151.874	1251.213	1599.826	1207.216
ENSMUSG(	702.5423	811.6638	624.6477	541.8082	534.834	536.5216	712.9513	537.7213
ENSMUSG(	614.9705	565.2284	660.6318	476.8724	455.5994	456.9996	613.6103	463.1571
ENSMUSG(	1873.446	1963.094	1805.665	1444.822	1417.31	1387.105	1880.735	1416.412
ENSMUSG(	914.0922	1189.182	990.9477	810.683	737.8729	781.1271	1031.407	776.561
ENSMUSG(	364.0625	364.9341	330.3159	273.948	250.5796	272.7905	353.1042	265.7727
ENSMUSG(	976.0812	1325.508	1085.06	864.458	895.3518	787.1668	1128.883	848.9922
ENSMUSG(	723.2053	848.367	869.1553	618.9195	649.7243	566.7198	813.5759	611.7879
ENSMUSG(	791.0981	1020.347	766.7389	650.3728	648.7339	638.1889	859.3948	645.7652

ENSMUSG(	18456	24492.53	18751.42	16045.23	15362.61	14943.08	20566.65	15450.31
ENSMUSG(	676.9596	626.0508	703.0746	511.3695	479.3698	516.3895	668.695	502.3762
ENSMUSG(	38187.21	51493.46	39312.21	33964.47	31671.09	30984.37	42997.63	32206.65
ENSMUSG(	3309.033	2568.172	3583.651	2306.236	2413.686	2363.514	3153.619	2361.145
ENSMUSG(	3197.847	4188.353	3304.082	2780.064	2576.117	2644.357	3563.427	2666.846
ENSMUSG(	1385.406	1619.133	1366.474	1106.953	1096.41	1065.997	1457.004	1089.786
ENSMUSG(	1638.281	1534.191	1502.107	1190.152	1204.367	1100.222	1558.193	1164.913
ENSMUSG(	415.2281	475.0436	371.8361	286.1234	314.9578	340.2332	420.7026	313.7715
ENSMUSG(	15418.54	15610.37	15974.19	11674.24	11768.33	11634.37	15667.7	11692.31
ENSMUSG(	731.077	984.693	790.7283	663.5628	605.1548	599.9378	835.4994	622.8852
ENSMUSG(	746.8202	673.2406	711.3787	524.5596	512.0541	552.6273	710.4798	529.747
ENSMUSG(	4343.168	4086.633	4008.079	3032.705	3122.837	3104.376	4145.96	3086.639
ENSMUSG(	1713.062	1856.13	1868.407	1377.857	1307.372	1361.939	1812.533	1349.056
ENSMUSG(	1954.13	2343.758	1826.887	1534.109	1548.047	1473.673	2041.592	1518.61
ENSMUSG(	795.0339	1087.462	954.9636	708.2062	707.1694	694.5589	945.8197	703.3115
ENSMUSG(	1164.016	1579.284	1229.919	998.3881	963.6917	986.475	1324.406	982.8516
ENSMUSG(	1497.576	1664.225	1440.288	1174.932	1131.075	1104.248	1534.03	1136.752
ENSMUSG(	901.3008	1218.544	909.7527	690.9576	761.6433	787.1668	1009.866	746.5892
ENSMUSG(	990.8405	1267.832	1000.174	915.1891	746.7868	742.8761	1086.282	801.6173
ENSMUSG(	495.9122	381.7127	481.6338	374.3955	307.0344	322.1143	453.0862	334.5147
ENSMUSG(	1286.026	1852.985	1433.829	1121.157	1134.046	1117.334	1524.28	1124.179
ENSMUSG(	1558.581	1958.899	1439.365	1360.608	1204.367	1088.142	1652.282	1217.706
ENSMUSG(	1741.597	2237.843	1718.934	1434.676	1373.731	1391.131	1899.458	1399.846
ENSMUSG(	872.7662	993.0822	860.8512	650.3728	691.3225	665.3673	908.8999	669.0209
ENSMUSG(	2131.242	2880.673	2285.454	1861.832	1790.704	1716.265	2432.456	1789.6
ENSMUSG(	520.5111	510.6981	563.7514	401.7903	360.5178	411.7023	531.6535	391.3368
ENSMUSG(	297.1538	294.6738	306.3265	243.5093	213.9336	203.3346	299.3847	220.2592
ENSMUSG(	2760.972	4464.151	3601.181	2714.114	2706.854	2534.637	3608.768	2651.868
ENSMUSG(	798.9697	950.0871	857.1605	708.2062	578.4131	627.1162	868.7391	637.9119
ENSMUSG(	5269.067	6824.688	5429.914	4424.767	4154.868	4287.14	5841.223	4288.925
ENSMUSG(	1021.343	747.6955	1032.468	674.7237	676.466	703.6184	933.8355	684.936
ENSMUSG(	429.0034	618.7102	466.8711	383.5271	370.4221	354.3257	504.8616	369.425
ENSMUSG(	790.1141	684.7758	771.3522	545.8667	541.7671	557.6603	748.7474	548.4314
ENSMUSG(	36568.61	46529.1	36581.1	30466.06	28971.17	28181.98	39892.94	29206.4
ENSMUSG(	1146.305	1353.822	1057.38	919.2476	821.0693	862.6623	1185.836	867.6597
ENSMUSG(	2852.479	3314.818	2562.255	2166.218	2138.346	2073.611	2909.851	2126.058
ENSMUSG(	807.8253	718.333	814.7177	543.8374	683.399	481.1582	780.292	569.4649
ENSMUSG(	511.6555	649.1213	526.8446	420.0535	411.0299	399.623	562.5405	410.2355
ENSMUSG(	1052.83	1176.598	1169.023	823.8731	817.1076	838.5037	1132.817	826.4948
novel.24	57.06926	40.89779	40.59749	12.17546	19.80867	9.059464	46.18818	13.6812
ENSMUSG(	1384.422	2022.868	1545.472	1196.239	1261.812	1148.539	1650.921	1202.197
ENSMUSG(	605.131	652.2673	618.189	487.0186	441.7333	436.8675	625.1958	455.2065
ENSMUSG(	251.8919	328.231	285.1051	211.0414	199.0771	219.4404	288.4093	209.853
ENSMUSG(	669.0879	521.1847	596.0449	413.9658	435.7907	448.9468	595.4392	432.9011
ENSMUSG(	269.6031	293.6252	267.5743	221.1876	191.1536	191.2554	276.9342	201.1989
ENSMUSG(	1540.87	1570.895	1692.177	1145.508	1174.654	1170.684	1601.314	1163.615
ENSMUSG(	3724.261	3509.869	3817.086	2717.158	2652.381	2657.443	3683.739	2675.66
ENSMUSG(	4093.244	5260.085	4058.826	3267.083	3288.239	3180.879	4470.718	3245.4
ENSMUSG(	763.5474	1044.467	762.1255	654.4312	604.1644	605.9775	856.7132	621.5244
ENSMUSG(	1692.399	2328.028	2324.206	1517.875	1563.894	1507.897	2114.878	1529.889

ENSMUSG(	657.2805	725.6736	729.8321	514.4134	505.121	508.3366	704.2621	509.2903
ENSMUSG(	1202.39	1726.096	1279.743	1048.105	1037.974	954.2636	1402.743	1013.447
ENSMUSG(	915.0761	1335.994	1036.159	844.1656	753.7198	775.0875	1095.743	790.991
ENSMUSG(	7658.105	6652.707	7399.814	5080.213	5247.316	5348.104	7236.876	5225.211
ENSMUSG(	1295.866	1100.046	1252.986	844.1656	870.591	920.0389	1216.299	878.2651
ENSMUSG(	1376.55	1098.997	1441.211	988.2419	931.0074	907.9596	1305.586	942.403
ENSMUSG(	1926.58	2500.009	2381.412	1731.96	1734.249	1430.389	2269.333	1632.199
ENSMUSG(	1554.645	1451.347	1428.293	1074.485	1053.821	1060.964	1478.095	1063.09
ENSMUSG(	784.2104	1053.905	824.8671	715.3086	600.2026	598.9312	887.6607	638.1475
ENSMUSG(	1550.71	2082.641	1524.251	1287.555	1215.262	1203.902	1719.201	1235.573
ENSMUSG(	1475.929	2053.279	1421.835	1246.971	1179.606	1130.42	1650.348	1185.666
ENSMUSG(	9498.097	12258.85	9843.045	7807.517	7421.317	7449.899	10533.33	7559.578
ENSMUSG(	320.7686	389.0533	346.924	243.5093	271.3788	242.5923	352.2486	252.4935
ENSMUSG(	246.9722	333.4743	273.1104	189.7343	208.9814	212.3941	284.5189	203.7033
ENSMUSG(	1468.058	1392.622	1289.893	973.0226	990.4334	1006.607	1383.524	990.021
ENSMUSG(	1029.215	1008.812	978.0303	686.8991	711.1312	758.9818	1005.352	719.004
ENSMUSG(	1479.865	1509.024	1497.494	1067.382	1109.285	1031.772	1495.461	1069.48
ENSMUSG(	447.6985	615.5642	562.8288	425.1267	374.3838	362.3786	542.0305	387.2963
novel.90	11.80743	18.8759	14.76272	0	0	1.006607	15.14869	0.335536
ENSMUSG(	235.1647	290.4792	258.3476	183.6466	198.0867	177.1629	261.3305	186.2987
ENSMUSG(	505.7518	638.6347	464.1031	354.1031	389.2403	402.6428	536.1632	381.9954
ENSMUSG(	682.8633	556.8391	686.4666	413.9658	487.2932	472.0987	642.0563	457.7859
ENSMUSG(	755.6758	953.2331	732.6001	595.5832	585.3461	557.6603	813.8363	579.5299
ENSMUSG(	7953.291	7298.683	7961.721	5455.623	5601.891	5478.963	7737.898	5512.159
ENSMUSG(	965.2577	1268.88	984.489	754.8788	786.4041	749.9223	1072.876	763.7351
ENSMUSG(	1962.002	1753.362	2160.893	1391.047	1398.492	1392.138	1958.752	1393.892
ENSMUSG(	2412.652	2065.863	2591.78	1630.498	1689.679	1708.212	2356.765	1676.13
ENSMUSG(	2439.219	1865.568	2377.721	1533.094	1634.215	1582.386	2227.503	1583.232
ENSMUSG(	398.5009	521.1847	464.1031	289.1673	360.5178	332.1804	461.2629	327.2885
ENSMUSG(	443.7627	498.1141	429.9643	332.796	299.1109	341.2398	457.2804	324.3822
ENSMUSG(	323.7205	338.7176	311.8625	235.3923	240.6753	214.4073	324.7669	230.1583
ENSMUSG(	250.908	286.2845	241.7396	182.632	168.3737	200.3148	259.644	183.7735
ENSMUSG(	1602.859	1952.607	1675.569	1230.737	1241.013	1232.087	1743.678	1234.612
ENSMUSG(	2670.448	2959.322	2529.039	1994.747	1918.469	1863.23	2719.603	1925.482
ENSMUSG(	2803.282	3701.774	2863.968	2352.909	2126.461	2150.113	3123.008	2209.827
ENSMUSG(	645.4731	637.5861	650.4824	469.77	452.6281	445.927	644.5139	456.1083
ENSMUSG(	540.1901	575.7151	516.6953	404.8342	376.3647	373.4512	544.2001	384.8834
ENSMUSG(	341.4316	345.0096	280.4917	231.3338	235.7231	215.4139	322.311	227.4903
ENSMUSG(	1821.297	2401.434	1872.098	1507.728	1382.645	1409.25	2031.61	1433.208
ENSMUSG(	714.3498	1011.958	848.8565	613.8464	649.7243	551.6207	858.3881	605.0638
ENSMUSG(	771.419	994.1309	817.4857	594.5685	624.9635	599.9378	861.0119	606.49
ENSMUSG(	994.7763	1102.143	1030.623	797.493	709.1503	696.5721	1042.514	734.4051
ENSMUSG(	1163.032	860.9509	1106.281	747.7765	741.8346	715.6977	1043.422	735.1029
ENSMUSG(	362.0946	399.54	363.532	282.0649	247.6083	261.7179	375.0555	263.797
ENSMUSG(	251.8919	290.4792	253.7343	182.632	173.3258	203.3346	265.3685	186.4308
ENSMUSG(	482.1369	686.8731	509.3139	421.0682	384.2882	373.4512	559.4413	392.9359
ENSMUSG(	409.3244	595.6396	501.0099	379.4687	321.8909	355.3323	501.9913	352.2306
ENSMUSG(	993.7924	1177.647	1028.777	747.7765	735.892	759.9884	1066.739	747.8856
ENSMUSG(	397.5169	540.0606	502.8552	352.0739	375.3743	281.85	480.1442	336.4327
ENSMUSG(	849.1513	893.4594	851.6245	664.5775	559.5949	593.8982	864.7451	606.0235

ENSMUSG(	5005.368	4733.657	5164.185	3571.47	3363.512	3500.98	4967.737	3478.654
ENSMUSG(	1689.447	2479.035	1884.092	1483.377	1403.444	1347.847	2017.525	1411.556
ENSMUSG(	612.0187	572.5691	543.4527	372.3663	431.829	403.6495	576.0135	402.6149
ENSMUSG(	19293.35	26770.23	19770.98	15851.44	14991.2	15184.67	21944.85	15342.44
ENSMUSG(	784.2104	921.7733	845.1658	641.2412	553.6523	585.8453	850.3832	593.5796
ENSMUSG(	2500.224	3692.336	2481.06	1971.411	1867.957	2212.522	2891.207	2017.297
ENSMUSG(	534.2864	454.0703	561.9061	341.9276	364.4795	375.4645	516.7543	360.6239
ENSMUSG(	2461.85	3432.268	2547.492	2018.083	1927.383	1935.705	2813.87	1960.391
ENSMUSG(	795.0339	1032.931	753.8215	619.9341	640.8104	533.5018	860.5956	598.0821
ENSMUSG(	2736.373	4031.054	3680.531	2531.482	2623.658	2097.769	3482.653	2417.636
ENSMUSG(	5600.66	6900.191	5476.047	4233.003	4208.352	4035.488	5992.299	4158.948
novel.52	147.5929	117.4501	154.0859	67.97968	72.30164	88.58143	139.7096	76.28758
ENSMUSG(	1210.262	1500.634	1299.12	939.54	895.3518	947.2173	1336.672	927.3697
ENSMUSG(	563.805	377.5181	555.4474	350.0446	323.8717	365.3984	498.9235	346.4382
ENSMUSG(	349.3033	351.3015	314.6305	212.056	263.4553	227.4932	338.4118	234.3348
ENSMUSG(	685.8151	766.5714	719.6827	535.7205	439.7524	527.4621	724.0231	500.9783
ENSMUSG(	529.3666	502.3088	429.9643	352.0739	344.6708	311.0416	487.2132	335.9288
ENSMUSG(	4063.725	5336.637	4080.97	3249.835	3129.77	2896.009	4493.778	3091.871
ENSMUSG(	766.4993	935.4059	656.0185	573.2615	528.8914	515.3828	785.9745	539.1786
ENSMUSG(	655.3126	757.1335	620.0343	447.4483	460.5515	485.1846	677.4935	464.3948
ENSMUSG(	613.9866	723.5763	532.3807	425.1267	457.5802	398.6164	623.3145	427.1078
ENSMUSG(	3842.336	4006.935	3726.665	2631.93	2610.782	2694.687	3858.645	2645.8
ENSMUSG(	217.4536	192.9537	190.9927	134.9447	152.5267	124.8193	200.4667	137.4303
ENSMUSG(	1040.038	1319.216	1132.116	857.3557	785.4137	748.9157	1163.79	797.2283
ENSMUSG(	239.1005	302.0145	249.1209	206.9829	166.3928	167.0968	263.412	180.1575
ENSMUSG(	9701.775	12449.71	9881.797	7624.885	7205.403	7081.481	10677.76	7303.923
ENSMUSG(	2432.331	2613.264	2356.5	1738.048	1735.239	1581.38	2467.365	1684.889
ENSMUSG(	1561.533	1859.276	1617.441	1162.757	1104.333	1167.664	1679.417	1144.918
ENSMUSG(	2218.814	2644.724	2393.406	1637.6	1616.387	1693.113	2418.981	1649.034
ENSMUSG(	3438.915	4490.368	3615.944	2671.5	2644.457	2526.584	3848.409	2614.18
ENSMUSG(	11194.43	12648.95	11024.99	8216.41	7712.505	7745.842	11622.79	7891.585
ENSMUSG(	702.5423	796.9826	721.528	565.1445	466.4941	475.1186	740.351	502.2524
ENSMUSG(	1648.121	2132.977	1568.539	1239.868	1198.424	1186.79	1783.212	1208.361
ENSMUSG(	3836.432	4652.91	3843.844	2847.03	2720.721	2789.308	4111.062	2785.686
ENSMUSG(	6679.072	7285.05	6841.599	4852.937	4689.702	4527.719	6935.24	4690.119
ENSMUSG(	2877.078	3874.803	3185.98	2336.675	2245.313	2125.954	3312.621	2235.98
ENSMUSG(	2916.436	4228.202	3818.932	2418.859	2676.151	2303.117	3654.523	2466.042
ENSMUSG(	549.0457	626.0508	562.8288	392.6587	407.0681	370.4314	579.3084	390.0528
ENSMUSG(	1204.358	1604.452	1320.341	923.3061	932.9883	924.0653	1376.384	926.7866
ENSMUSG(	2054.494	1741.826	1950.525	1264.219	1314.305	1292.484	1915.615	1290.336
ENSMUSG(	5223.806	6016.17	4970.424	3698.297	3511.086	3700.288	5403.466	3636.557
ENSMUSG(	1284.058	1317.119	1337.872	870.5457	917.1413	859.6425	1313.016	882.4432
ENSMUSG(	1103.995	1169.257	1094.287	743.718	765.605	744.8893	1122.513	751.4041
ENSMUSG(	1132.53	1559.359	1198.548	874.6042	882.4762	844.5434	1296.813	867.2079
ENSMUSG(	1151.225	1310.827	1071.22	834.0193	737.8729	789.18	1177.757	787.0241
ENSMUSG(	1133.514	1439.812	1129.348	885.7651	842.8588	739.8562	1234.225	822.8267
ENSMUSG(	470.3295	559.9851	448.4177	333.8107	336.7474	314.0614	492.9108	328.2065
ENSMUSG(	1899.029	2541.955	1979.127	1508.743	1423.253	1343.821	2140.037	1425.272
ENSMUSG(	4186.719	4728.414	4082.815	2936.316	2921.779	2799.374	4332.649	2885.823
ENSMUSG(	3944.667	3326.354	4135.408	2529.453	2571.165	2486.32	3802.143	2528.979

ENSMUSG(	590.3717	448.827	638.4877	359.1762	402.116	353.3191	559.2288	371.5371
ENSMUSG(	806.8413	1051.807	836.8618	634.1388	569.4992	583.8321	898.5035	595.8234
ENSMUSG(	3430.06	4632.986	3448.941	2532.497	2555.318	2547.723	3837.329	2545.179
ENSMUSG(	182.0313	191.905	176.23	133.9301	118.852	111.7334	183.3888	121.5052
ENSMUSG(	589.3877	720.4303	563.7514	454.5507	381.3169	404.6561	624.5232	413.5079
ENSMUSG(	3450.723	3674.509	3435.101	2400.596	2215.6	2378.613	3520.111	2331.603
ENSMUSG(	904.2527	954.2818	874.6913	627.0364	571.4801	611.0105	911.0752	603.1757
ENSMUSG(	2172.568	1836.206	1980.973	1337.272	1259.831	1361.939	1996.582	1319.681
ENSMUSG(	2661.592	2642.626	2792	1765.442	1817.445	1767.602	2698.74	1783.497
ENSMUSG(	363.0786	440.4377	417.0469	290.1819	245.6275	269.7707	406.8544	268.5267
ENSMUSG(	1283.074	1121.019	1314.805	861.4141	795.318	795.2196	1239.633	817.3173
ENSMUSG(	656.2965	822.1505	647.7144	480.9309	441.7333	476.1252	708.7205	466.2631
ENSMUSG(	15406.73	11290.94	16011.09	9163.052	9405.156	9494.318	14236.25	9354.175
ENSMUSG(	7433.764	9944.455	8210.841	5931.481	5507.8	5337.031	8529.687	5592.104
ENSMUSG(	359.1428	361.7881	336.7746	244.5239	221.8571	226.4866	352.5685	230.9559
ENSMUSG(	54848.48	62203.44	52684.46	37668.86	36750.03	36740.15	56578.8	37053.01
ENSMUSG(	11572.27	13399.79	11212.29	8103.787	7842.252	7684.439	12061.45	7876.826
ENSMUSG(	193.8387	195.051	178.998	117.6962	134.6989	117.773	189.2959	123.3894
ENSMUSG(	329.6242	357.5935	321.0892	241.4801	215.9145	199.3082	336.1023	218.9009
ENSMUSG(	180.0634	178.2724	214.9821	128.857	107.9572	136.8986	191.106	124.5709
ENSMUSG(	4296.922	4678.078	4131.717	2922.112	2801.936	2786.289	4368.906	2836.779
ENSMUSG(	613.9866	454.0703	513.0046	335.8399	351.6039	337.2134	527.0205	341.5524
ENSMUSG(	593.3236	526.428	628.3384	338.8838	395.1829	397.6098	582.6966	377.2255
ENSMUSG(	2175.52	2706.595	2250.392	1592.957	1516.354	1502.864	2377.502	1537.392
ENSMUSG(	1009.536	1150.381	1004.788	736.6156	672.5043	634.1625	1054.902	681.0941
ENSMUSG(	1302.754	1330.751	1220.693	836.0486	817.1076	834.4773	1284.732	829.2111
ENSMUSG(	152.5127	239.0948	225.1315	140.0178	117.8616	139.9184	205.5797	132.5993
ENSMUSG(	273.5389	225.4622	251.8889	175.5296	153.5172	155.0175	250.2967	161.3548
ENSMUSG(	140.7053	191.905	165.158	109.5792	106.9668	103.6805	165.9227	106.7422
ENSMUSG(	1175.824	1205.96	1175.482	774.1566	753.7198	762.0016	1185.755	763.2927
ENSMUSG(	849.1513	1047.613	886.686	603.7001	579.4035	602.9577	927.8166	595.3538
ENSMUSG(	13071.81	12307.09	13077.93	8294.535	7723.4	8645.749	12818.94	8221.228
ENSMUSG(	470.3295	534.8173	521.3086	319.606	320.9004	338.22	508.8184	326.2421
ENSMUSG(	592.3396	543.2066	578.5142	356.1323	358.5369	383.5173	571.3534	366.0622
ENSMUSG(	223.3573	267.4086	287.8731	168.4273	176.2971	154.0109	259.5463	166.2451
ENSMUSG(	7156.289	9357.205	7194.059	5281.108	4959.1	4940.428	7902.518	5060.212
ENSMUSG(	2747.196	1845.644	2556.719	1439.749	1520.315	1616.611	2383.186	1525.558
ENSMUSG(	306.0093	438.3404	331.2386	231.3338	216.9049	237.5593	358.5294	228.5993
ENSMUSG(	7696.479	8733.251	7951.571	5223.274	5117.569	5199.126	8127.101	5179.99
ENSMUSG(	4359.895	6091.673	6388.568	3822.081	3773.551	3118.469	5613.379	3571.367
ENSMUSG(	1630.41	1974.629	1660.806	2329.572	2266.112	2298.084	1755.282	2297.923
ENSMUSG(	8946.099	9122.305	8939.751	5795.521	5637.547	5719.542	9002.718	5717.537
ENSMUSG(	1202.39	684.7758	1461.509	671.6798	745.7963	708.6514	1116.225	708.7092
ENSMUSG(	115.1225	120.596	138.4005	72.03817	92.11031	72.47571	124.7063	78.87473
ENSMUSG(	617.9224	611.3695	536.0713	401.7903	347.6421	365.3984	588.4544	371.6103
ENSMUSG(	492.9604	664.8513	513.0046	340.913	345.6613	367.4116	556.9387	351.3286
ENSMUSG(	7604.971	7444.447	7456.097	4737.27	4740.214	4723.001	7501.838	4733.495
ENSMUSG(	1778.003	2110.955	1794.593	1182.035	1201.396	1188.803	1894.517	1190.744
ENSMUSG(	783.2265	724.625	811.9497	491.0771	472.4367	495.2507	773.267	486.2548
ENSMUSG(	1445.427	1352.773	1377.546	881.7066	928.0361	814.3452	1391.915	874.6959

ENSMUSG(	2674.384	2809.364	2589.935	1690.36	1704.536	1677.007	2691.227	1690.635
ENSMUSG(	408.3404	509.6494	597.8902	341.9276	341.6995	266.7509	505.2934	316.7927
ENSMUSG(	107.2509	113.2554	95.95769	75.08203	61.40687	61.40303	105.488	65.96398
ENSMUSG(	364123.6	455655.9	366202.2	254925.8	243812	243107.7	395327.2	247281.8
ENSMUSG(	703.5263	935.4059	745.5175	537.7497	465.5037	487.1978	794.8165	496.8171
ENSMUSG(	928.8515	1328.654	991.8704	708.2062	628.9252	693.5523	1083.125	676.8946
ENSMUSG(	1468.058	1202.815	1407.072	862.4288	808.1937	878.768	1359.315	849.7968
ENSMUSG(	2241.445	1786.919	2235.63	1285.526	1305.391	1323.688	2087.998	1304.869
ENSMUSG(	3604.219	4692.759	3783.87	2538.584	2561.261	2447.062	4026.95	2515.636
ENSMUSG(	227.2931	203.4403	232.5129	124.7985	142.6224	146.9646	221.0821	138.1285
ENSMUSG(	2001.36	2337.466	2103.688	1361.623	1309.353	1350.867	2147.505	1340.614
ENSMUSG(	758.6276	1047.613	781.5016	598.627	538.7958	475.1186	862.5806	537.5138
ENSMUSG(	378.8218	450.9244	442.8817	302.3574	244.637	245.6121	424.2093	264.2022
ENSMUSG(	1770.131	2364.731	1912.695	1329.155	1244.975	1185.783	2015.853	1253.304
ENSMUSG(	797.0018	701.5544	757.5122	483.9747	499.1784	417.742	752.0228	466.965
ENSMUSG(	4909.925	2874.381	5200.169	2662.368	2698.931	2697.707	4328.158	2686.335
ENSMUSG(	74.78042	79.69826	59.05089	31.45328	36.64604	33.21804	71.17652	33.77245
ENSMUSG(	383.7416	459.3136	372.7587	279.0211	236.7136	236.5527	405.2713	250.7624
ENSMUSG(	5365.495	5246.452	5463.13	3270.127	3383.32	3297.645	5358.359	3317.031
ENSMUSG(	389.6453	450.9244	396.7482	240.4654	237.704	286.883	412.4393	255.0175
ENSMUSG(	3038.446	3263.434	3100.172	1998.805	1879.843	1929.666	3134.017	1936.105
ENSMUSG(	943.6108	968.963	952.1956	606.744	559.5949	602.9577	954.9231	589.7655
ENSMUSG(	725.1732	792.7879	694.7706	431.2144	469.4654	465.0525	737.5773	455.2441
ENSMUSG(	659.2484	702.6031	717.8374	448.463	416.9725	416.7353	693.2296	427.3903
ENSMUSG(	4150.313	3199.466	4180.618	2360.011	2391.897	2355.461	3843.466	2369.123
ENSMUSG(	684.8312	445.6811	606.1943	332.796	388.2499	349.2927	578.9022	356.7795
ENSMUSG(	1122.69	1231.128	1122.89	677.7675	706.179	752.9421	1158.903	712.2962
ENSMUSG(	365.0465	442.5351	375.5267	256.6994	248.5988	221.4536	394.3694	242.2506
ENSMUSG(	1404.101	1622.279	1316.65	901.999	856.7249	899.9068	1447.677	886.2102
ENSMUSG(	173.1757	116.4014	117.1791	78.1259	89.13901	81.53518	135.5854	82.93336
ENSMUSG(	13293.2	18120.87	13679.51	9830.673	8839.618	8910.486	15031.19	9193.593
ENSMUSG(	463.4418	392.1993	437.3456	258.7286	278.3118	252.6584	430.9956	263.2329
ENSMUSG(	535.2703	552.6445	575.7462	335.8399	339.7187	338.22	554.5537	337.9262
ENSMUSG(	911.1403	822.1505	1052.767	556.0129	537.8053	601.9511	928.6858	565.2564
ENSMUSG(	740.9165	892.4108	739.9814	464.6969	493.2358	482.1648	791.1029	480.0325
ENSMUSG(	4368.751	5131.1	4568.14	2877.468	2853.439	2791.322	4689.33	2840.743
ENSMUSG(	120.0422	174.0778	136.5552	90.30136	78.24424	91.60125	143.5584	86.71562
ENSMUSG(	7392.438	6268.897	8058.601	4379.109	4399.505	4346.53	7239.979	4375.048
ENSMUSG(	242.0524	189.8077	239.8942	134.9447	118.852	151.9977	223.9181	135.2648
ENSMUSG(	113.1546	130.034	143.0139	93.34523	83.19641	56.37	128.7341	77.63721
ENSMUSG(	482.1369	552.6445	492.7058	329.7522	285.2448	301.9821	509.1624	305.6597
ENSMUSG(	122.9941	205.5376	152.2406	95.37448	76.26337	116.7664	160.2574	96.13476
ENSMUSG(	264.6833	360.7395	280.4917	205.9683	168.3737	169.11	301.9715	181.1507
ENSMUSG(	123.9781	176.1751	116.2564	95.37448	65.3686	88.58143	138.8032	83.10817
ENSMUSG(	629.7298	714.1383	651.4051	395.7026	394.1925	401.6362	665.0911	397.1771
ENSMUSG(	420.1479	493.9195	513.9273	315.5475	276.3309	258.698	475.9982	283.5255
ENSMUSG(	93.47552	95.42818	107.9524	55.80421	58.43557	62.40964	98.95203	58.88314
ENSMUSG(	2244.396	2670.94	2241.166	1466.129	1363.827	1410.257	2385.501	1413.404
ENSMUSG(	135.7855	151.0072	137.4778	85.22825	86.16771	79.52196	141.4235	83.63931
ENSMUSG(	1191.567	719.3817	1174.559	568.1884	640.8104	614.0303	1028.503	607.6764

ENSMUSG(	80.68413	117.4501	83.04031	50.7311	51.50254	63.41625	93.72484	55.21663
ENSMUSG(	1124.658	1058.099	1198.548	670.6652	674.4851	650.2682	1127.102	665.1395
ENSMUSG(	898.3489	1322.362	978.0303	635.1534	645.7626	604.9709	1066.247	628.629
ENSMUSG(	1883.286	2512.592	2029.874	1282.482	1244.975	1242.153	2141.917	1256.537
ENSMUSG(	1629.426	1754.41	1695.868	975.0518	1020.146	984.4618	1693.235	993.22
ENSMUSG(	1403.117	1248.956	1205.93	797.493	684.3895	776.0941	1286.001	752.6588
ENSMUSG(	3119.13	3205.758	2984.838	1851.685	1810.512	1772.635	3103.242	1811.611
ENSMUSG(	1199.439	1287.756	1044.463	707.1916	665.5712	685.4995	1177.219	686.0874
ENSMUSG(	43418.89	32984.59	43070.24	22799.57	23351.45	23474.08	39824.57	23208.37
ENSMUSG(	4543.894	4200.937	4414.977	2626.857	2467.17	2565.842	4386.603	2553.289
ENSMUSG(	320.7686	358.6422	428.1189	226.2607	204.0293	212.3941	369.1766	214.228
ENSMUSG(	2081.06	1861.374	2113.837	1185.079	1120.18	1205.915	2018.757	1170.391
ENSMUSG(	505.7518	358.6422	476.0978	220.173	300.1013	256.6848	446.8306	258.9864
ENSMUSG(	765.5153	765.5227	729.8321	429.1851	428.8577	449.9534	753.6234	435.9987
ENSMUSG(	2545.486	2770.563	2373.108	1491.494	1465.841	1484.746	2563.052	1480.694
ENSMUSG(	200.7264	206.5863	203.9101	115.6669	116.8711	119.7862	203.7409	117.4414
ENSMUSG(	890.4773	1076.975	853.4699	532.6766	540.7766	549.6075	940.3074	541.0202
ENSMUSG(	1568.421	2214.773	1557.467	1066.368	1009.252	991.508	1780.22	1022.376
ENSMUSG(	325.6884	458.265	395.8255	260.7579	208.9814	207.3611	393.2596	225.7001
ENSMUSG(	1359.823	1906.466	1479.963	930.4084	922.0935	860.6491	1582.084	904.3837
ENSMUSG(	482.1369	353.3989	455.799	214.0853	261.4744	262.7245	430.4449	246.0947
ENSMUSG(	2597.635	3689.19	2583.476	1715.726	1618.368	1728.344	2956.767	1687.48
ENSMUSG(	228.2771	209.7323	212.2141	114.6523	141.632	113.7466	216.7411	123.3436
ENSMUSG(	2822.961	3779.375	3024.513	1934.884	1807.541	1736.397	3208.95	1826.274
ENSMUSG(	472.2974	401.6373	435.5003	241.4801	226.8092	275.8104	436.4783	248.0332
ENSMUSG(	4560.621	3955.55	4989.8	2575.111	2521.643	2575.908	4501.991	2557.554
ENSMUSG(	537.2382	552.6445	549.9114	306.4159	323.8717	300.9755	546.598	310.421
ENSMUSG(	48672.21	59922.6	49368.39	31096.14	29089.03	29419.1	52654.4	29868.09
ENSMUSG(	6426.196	7171.795	6384.877	3967.172	3735.915	3627.812	6660.956	3776.966
ENSMUSG(	443.7627	335.5716	438.2683	209.0121	224.8284	254.6716	405.8676	229.504
ENSMUSG(	1770.131	1123.116	1914.541	867.5019	939.9213	905.9464	1602.596	904.4565
ENSMUSG(	493.9443	336.6203	504.7006	242.4947	249.5892	260.7112	445.0884	250.9317
ENSMUSG(	282.3945	236.9975	249.1209	154.2226	149.5554	198.3016	256.171	167.3599
ENSMUSG(	10790.03	12494.8	10245.33	6488.508	6170.4	6191.64	11176.72	6283.516
ENSMUSG(	587.4198	699.4571	590.5089	352.0739	359.5273	342.2464	625.7953	351.2825
ENSMUSG(	627.7619	639.6834	625.5703	353.0885	325.8526	379.4909	631.0052	352.8107
ENSMUSG(	64.94089	67.11432	63.66424	35.51177	43.57907	30.19821	65.23982	36.42969
ENSMUSG(	209.582	314.5984	220.5182	153.2079	144.6033	117.773	248.2328	138.5281
ENSMUSG(	195.8066	261.1167	205.7554	132.9155	118.852	117.773	220.8929	123.1802
ENSMUSG(	422.1158	605.0776	388.4441	260.7579	233.7423	292.9227	471.8792	262.4743
ENSMUSG(	569.7087	657.5106	596.9676	327.7229	346.6517	337.2134	608.0623	337.196
ENSMUSG(	3818.721	4457.859	3795.865	2284.929	2302.758	2104.815	4024.148	2230.834
ENSMUSG(	348.3193	377.5181	376.4494	221.1876	182.2397	206.3545	367.4289	203.2606
ENSMUSG(	631.6977	430.9998	553.6021	312.5036	287.2257	293.9293	538.7665	297.8862
ENSMUSG(	164.3201	155.2019	126.4058	93.34523	67.34947	85.56161	148.6426	82.08544
ENSMUSG(	541.1741	467.7029	535.1487	270.9041	281.2831	299.9689	514.6752	284.052
ENSMUSG(	74.78042	78.6496	88.57633	48.70186	44.5695	40.26428	80.66878	44.51188
ENSMUSG(	1462.154	920.7246	1525.174	683.8553	733.9111	736.8364	1302.684	718.2009
ENSMUSG(	1870.494	1491.196	1949.602	973.0226	945.8639	1001.574	1770.431	973.4869
ENSMUSG(	1150.241	881.9242	1128.426	590.51	587.327	559.6736	1053.53	579.1702

ENSMUSG(	165.3041	190.8564	188.2247	101.4622	103.9955	93.61446	181.4617	99.69073
ENSMUSG(	2078.108	2032.306	2033.565	1132.318	1138.998	1097.202	2047.993	1122.839
ENSMUSG(	309.9451	381.7127	268.497	191.7636	178.278	155.0175	320.0516	175.0197
ENSMUSG(	319.7847	368.0801	303.5585	189.7343	176.2971	176.1562	330.4744	180.7292
ENSMUSG(	257.7956	254.8247	290.6411	134.9447	164.4119	138.9118	267.7538	146.0895
ENSMUSG(	120.0422	134.2286	112.5658	74.06741	67.34947	57.37661	122.2789	66.2645
ENSMUSG(	368.9823	368.0801	362.6094	187.7051	190.1632	216.4205	366.5573	198.0963
ENSMUSG(	219.4215	146.8126	181.766	93.34523	103.0051	99.65411	182.6667	98.66814
ENSMUSG(	1048.894	1292.999	1165.332	597.6124	647.7434	645.2352	1169.075	630.197
ENSMUSG(	219.4215	231.7541	197.4514	136.974	112.9094	99.65411	216.209	116.5125
ENSMUSG(	100.3632	133.18	137.4778	84.21363	62.3973	53.35018	123.6737	66.6537
ENSMUSG(	4936.491	5798.048	4945.512	2981.974	2752.414	2700.727	5226.684	2811.705
ENSMUSG(	114.1385	100.6715	119.0244	64.93581	54.47384	60.39643	111.2782	59.93536
ENSMUSG(	658.2644	678.4839	644.9464	378.454	368.4412	317.0812	660.5649	354.6588
ENSMUSG(	1910.836	1795.308	1784.444	943.5985	1034.012	968.3561	1830.196	981.989
ENSMUSG(	179.0794	271.6033	179.9207	128.857	119.8424	88.58143	210.2011	112.427
ENSMUSG(	2636.994	3926.188	3440.637	1146.523	2270.073	1930.672	3334.606	1782.423
ENSMUSG(	144.6411	109.0608	163.3126	67.97968	70.32077	84.555	139.0048	74.28515
ENSMUSG(	134.8015	102.7688	118.1018	71.02355	61.40687	57.37661	118.5574	63.26901
ENSMUSG(	3489.097	4083.487	3356.674	1934.884	1944.221	1942.752	3643.086	1940.619
ENSMUSG(	102.3311	139.472	113.4884	73.05279	66.35904	49.32375	118.4305	62.91186
ENSMUSG(	505.7518	242.2408	200.2194	172.4858	191.1536	139.9184	316.0706	167.8526
ENSMUSG(	91.50761	73.40629	75.65895	36.52639	39.61734	51.33696	80.19095	42.49356
ENSMUSG(	952.4663	1006.715	1032.468	521.5157	517.9967	544.5745	997.2164	528.029
ENSMUSG(	2515.967	2750.639	2262.387	1337.272	1344.018	1300.536	2509.664	1327.275
ENSMUSG(	466.3936	393.248	509.3139	219.1584	249.5892	252.6584	456.3185	240.4687
ENSMUSG(	2496.288	3227.779	2593.626	1518.889	1473.765	1384.085	2772.565	1458.913
ENSMUSG(	131.8497	109.0608	151.3179	78.1259	60.41644	67.44268	130.7428	68.66167
ENSMUSG(	1564.485	1345.432	1605.446	871.5604	743.8155	744.8893	1505.121	786.755
ENSMUSG(	1395.245	1429.325	1433.829	1729.931	1713.45	1780.688	1419.467	1741.356
ENSMUSG(	2709.806	2371.023	2541.956	1316.979	1342.037	1312.616	2540.929	1323.877
ENSMUSG(	1403.117	1471.272	1553.776	762.9958	730.9398	809.3121	1476.055	767.7493
ENSMUSG(	1502.496	1879.201	1669.11	903.0136	873.5623	849.5764	1683.602	875.3841
ENSMUSG(	634.6496	791.7393	599.7356	343.9569	376.3647	332.1804	675.3748	350.834
ENSMUSG(	1226.989	1722.951	1265.903	804.5953	701.2268	683.4862	1405.281	729.7695
ENSMUSG(	343.3995	389.0533	347.8466	205.9683	167.3832	187.2289	360.0998	186.8602
ENSMUSG(	8005.44	9863.708	8389.839	4713.934	4426.247	4478.395	8752.996	4539.525
ENSMUSG(	117.0904	87.03889	111.6431	46.67262	51.50254	65.42946	105.2575	54.53487
ENSMUSG(	109.2188	62.91968	117.1791	50.7311	48.53124	50.33036	96.43918	49.86423
ENSMUSG(	6949.659	9539.672	7428.417	4288.808	4085.538	3963.012	7972.583	4112.453
ENSMUSG(	2127.306	2185.41	2277.15	1137.391	1169.702	1078.076	2196.622	1128.39
ENSMUSG(	238.1166	229.6568	199.2967	121.7546	106.9668	113.7466	222.3567	114.156
ENSMUSG(	3301.162	3572.789	3326.226	1799.94	1670.861	1757.536	3400.059	1742.779
ENSMUSG(	66.90879	60.82236	70.12293	32.46791	38.6269	30.19821	65.95136	33.76434
ENSMUSG(	4659.017	5965.834	4860.626	2832.825	2616.725	2467.194	5161.826	2638.915
ENSMUSG(	1014.455	757.1335	1123.812	505.2818	490.2645	485.1846	965.1337	493.577
ENSMUSG(	199.7424	265.3113	196.5287	122.7693	114.8903	98.6475	220.5275	112.1023
ENSMUSG(	99.37924	82.84424	102.4164	55.80421	45.55994	43.28411	94.87995	48.21609
ENSMUSG(	1725.853	2065.863	1674.646	939.54	935.9596	895.8803	1822.121	923.7933
ENSMUSG(	397.5169	314.5984	348.7693	168.4273	190.1632	179.1761	353.6282	179.2555

ENSMUSG(	2213.894	3329.5	2252.238	1367.711	1253.889	1326.708	2598.544	1316.102
ENSMUSG(	256.8117	177.2238	213.1368	136.974	98.05291	91.60125	215.7241	108.876
ENSMUSG(	236.1487	125.8394	218.6728	99.43296	102.0146	91.60125	193.5536	97.68295
ENSMUSG(	198.7585	92.28219	197.4514	95.37448	66.35904	84.555	162.8307	82.09617
ENSMUSG(	108.2348	75.50361	97.80303	52.76035	42.58864	46.30393	93.84715	47.21764
ENSMUSG(	3835.448	3264.483	4215.68	1909.519	1934.316	1835.045	3771.87	1892.96
ENSMUSG(	3222.446	3304.332	3139.846	1545.269	1640.158	1657.882	3222.208	1614.436
ENSMUSG(	54.11741	57.67637	60.89623	33.48253	28.72257	24.15857	57.56334	28.78789
ENSMUSG(	866.8624	568.3744	936.5102	381.4979	414.9916	389.557	790.5824	395.3488
ENSMUSG(	363.0786	393.248	411.5109	195.8221	175.3067	211.3875	389.2792	194.1721
ENSMUSG(	2416.588	2747.493	2299.294	1256.102	1246.956	1210.948	2487.792	1238.002
ENSMUSG(	13807.81	6384.25	15928.98	5709.278	6274.396	5883.619	12040.35	5955.764
ENSMUSG(	2365.423	2405.629	2430.313	1246.971	1127.113	1185.783	2400.455	1186.622
ENSMUSG(	2624.202	2738.055	2651.754	1327.126	1336.095	1292.484	2671.337	1318.568
ENSMUSG(	201.7103	184.5644	183.6114	98.41834	104.9859	77.50875	189.962	93.63768
ENSMUSG(	404.4046	484.4815	379.2174	189.7343	206.0101	226.4866	422.7012	207.4104
ENSMUSG(	2500.224	2855.505	2644.373	1403.222	1267.755	1257.252	2666.7	1309.41
ENSMUSG(	237.1326	250.6301	200.2194	107.5499	119.8424	109.7202	229.3274	112.3709
ENSMUSG(	54.11741	81.79558	47.05618	27.3948	27.73214	34.22464	60.98972	29.78386
ENSMUSG(	162.3522	189.8077	145.7819	83.19901	81.21554	78.51536	165.9806	80.97663
ENSMUSG(	408.3404	329.2796	404.1295	192.7782	170.3545	194.2752	380.5832	185.8026
ENSMUSG(	72.81251	53.48173	68.27759	42.61413	26.7417	25.16518	64.85728	31.507
ENSMUSG(	1386.39	1190.231	1217.002	587.4662	614.0687	635.1691	1264.541	612.2347
ENSMUSG(	42.30997	58.72503	44.28817	22.32169	28.72257	19.12554	48.44106	23.38993
ENSMUSG(	1260.444	1069.635	1280.666	544.8521	627.9348	571.7528	1203.581	581.5132
ENSMUSG(	362.0946	411.0752	377.3721	203.939	169.3641	181.1893	383.514	184.8308
ENSMUSG(	813.729	586.2017	860.8512	388.6003	340.7091	357.3455	753.594	362.2183
ENSMUSG(	1117.77	1007.764	1134.884	533.6912	527.901	501.2903	1086.806	520.9609
ENSMUSG(	51.16555	131.0827	63.66424	44.64337	34.66517	38.25107	81.97082	39.18654
novel.112	122.9941	44.04377	124.5605	180.6027	197.0962	207.3611	97.19945	195.02
ENSMUSG(	1887.222	1703.026	1825.964	851.2679	884.457	857.6293	1805.404	864.4514
ENSMUSG(	6076.893	5935.423	5900.475	2824.708	2835.611	2880.91	5970.93	2847.076
ENSMUSG(	110.2027	79.69826	92.26701	42.61413	39.61734	52.34357	94.056	44.85834
ENSMUSG(	64.94089	75.50361	64.58691	20.29244	34.66517	42.2775	68.3438	32.4117
ENSMUSG(	1207.31	1480.71	1320.341	671.6798	606.1452	624.0964	1336.12	633.9738
ENSMUSG(	206.6301	196.0997	183.6114	90.30136	86.16771	101.6673	195.447	92.71213
ENSMUSG(	1504.464	1817.33	1541.782	801.5514	790.3659	699.592	1621.192	763.8364
ENSMUSG(	1753.404	1660.031	1591.606	764.0104	776.4998	817.365	1668.347	785.9584
ENSMUSG(	5806.306	4298.463	5834.043	2539.599	2579.089	2377.606	5312.937	2498.765
ENSMUSG(	702.5423	778.1067	674.4719	341.9276	329.8143	340.2332	718.3736	337.3251
ENSMUSG(	250.908	161.4938	263.8837	111.6084	90.12944	115.7598	225.4285	105.8326
ENSMUSG(	178.0955	171.9805	182.6887	85.22825	91.11987	70.4625	177.5882	82.27021
ENSMUSG(	38.37416	46.1411	57.20555	26.38017	19.80867	19.12554	47.24027	21.77146
ENSMUSG(	11843.84	16298.29	11912.59	6465.172	6072.347	5870.533	13351.58	6136.017
ENSMUSG(	389.6453	232.8028	429.9643	143.0617	167.3832	173.1364	350.8041	161.1938
ENSMUSG(	1727.821	1184.987	1694.022	667.6213	697.2651	749.9223	1535.61	704.9362
ENSMUSG(	19456.68	21187.15	19551.38	9689.641	8928.757	8998.061	20065.07	9205.486
ENSMUSG(	1031.183	854.659	936.5102	425.1267	451.6376	410.6957	940.7839	429.1533
ENSMUSG(	127.9139	173.0291	164.2353	83.19901	59.426	69.45589	155.0594	70.69364
ENSMUSG(	44.27788	93.33086	63.66424	37.54102	23.7704	30.19821	67.09099	30.50321

ENSMUSG(	954.4343	988.8876	1001.097	437.3021	428.8577	473.1053	981.473	446.4217
ENSMUSG(	61.00508	80.74692	75.65895	42.61413	35.6556	20.13214	72.47032	32.80062
ENSMUSG(	711.3979	880.8755	726.1414	344.9715	337.7378	364.3918	772.8049	349.0337
ENSMUSG(	1467.074	1283.561	1377.546	626.0218	624.9635	610.0039	1376.061	620.3297
ENSMUSG(	197.7745	243.2894	184.534	90.30136	90.12944	100.6607	208.5327	93.69717
ENSMUSG(	984.9368	1462.883	892.222	421.0682	485.3124	591.885	1113.347	499.4218
ENSMUSG(	1718.966	1625.425	1781.676	737.6303	778.4807	776.0941	1708.689	764.0683
ENSMUSG(	60.02112	58.72503	49.82419	27.3948	24.76083	23.15196	56.19011	25.10253
ENSMUSG(	2616.331	2367.877	2727.413	1153.625	1178.616	1109.281	2570.54	1147.174
ENSMUSG(	67.89275	58.72503	60.89623	32.46791	24.76083	26.17179	62.50467	27.80018
ENSMUSG(	1263.395	1268.88	1250.218	583.4077	571.4801	525.4489	1260.831	560.1122
ENSMUSG(	19691.85	26803.78	19269.97	10143.18	9605.223	9432.915	21921.87	9727.105
ENSMUSG(	50.18159	72.35763	81.19497	33.48253	27.73214	29.19161	67.9114	30.13542
ENSMUSG(	1157.129	1004.618	1134.884	496.1502	504.1306	461.0261	1098.877	487.1023
ENSMUSG(	311.913	361.7881	318.3212	128.857	173.3258	136.8986	330.6741	146.3605
ENSMUSG(	722.2214	845.221	727.0641	357.147	319.91	335.2002	764.8355	337.419
ENSMUSG(	1181.727	1413.595	1279.743	561.086	543.7479	602.9577	1291.689	569.2639
ENSMUSG(	341.4316	401.6373	339.5426	168.4273	172.3354	133.8787	360.8705	158.2138
ENSMUSG(	81.66809	49.28708	73.81361	31.45328	29.713	28.185	68.25626	29.78376
ENSMUSG(	868.8304	743.5009	813.7951	343.9569	355.5656	354.3257	808.7088	351.2827
ENSMUSG(	381.7737	378.5667	430.8869	197.8513	166.3928	151.9977	397.0758	172.0806
ENSMUSG(	2115.499	2526.225	2211.64	994.3296	959.73	991.508	2284.455	981.8559
ENSMUSG(	91.50761	98.57416	88.57633	40.58488	33.67474	45.29732	92.88604	39.85231
ENSMUSG(	1238.797	1041.321	1176.404	538.7643	497.1976	442.9071	1152.174	492.9563
ENSMUSG(	621.8582	744.5495	548.9887	271.9187	273.3596	272.7905	638.4655	272.6896
ENSMUSG(	561.8371	848.367	691.0799	315.5475	284.2544	296.9491	700.428	298.917
ENSMUSG(	2806.233	3457.436	2935.014	1346.403	1314.305	1252.219	3066.228	1304.309
ENSMUSG(	1458.218	1441.909	1384.928	598.627	629.9156	583.8321	1428.352	604.1249
ENSMUSG(	75.76437	71.30897	69.20026	37.54102	33.67474	20.13214	72.0912	30.4493
ENSMUSG(	98.39528	112.2068	110.7204	63.92119	47.5408	73.48232	107.1075	61.6481
ENSMUSG(	830.4562	903.946	836.8618	358.1616	364.4795	362.3786	857.088	361.6732
ENSMUSG(	58.05322	63.96834	57.20555	15.21933	30.70344	29.19161	59.74237	25.03812
ENSMUSG(	6787.307	9463.12	7080.571	3327.96	3250.602	3163.766	7776.999	3247.443
ENSMUSG(	1621.554	2581.804	1978.205	760.9666	997.3664	822.398	2060.521	860.2437
ENSMUSG(	46.24578	65.017	45.21084	20.29244	29.713	15.09911	52.15787	21.70152
ENSMUSG(	37.39021	37.75181	28.60277	16.23395	13.86607	13.08589	34.5816	14.3953
ENSMUSG(	1778.987	2493.717	1801.052	877.6481	819.0884	833.4707	2024.585	843.4024
ENSMUSG(	1718.966	1996.651	1879.479	822.8585	734.9016	773.0743	1865.032	776.9448
ENSMUSG(	191.8708	180.3697	206.6781	63.92119	83.19641	93.61446	192.9729	80.24402
ENSMUSG(	8187.472	10697.39	8536.544	3985.436	3645.785	3758.671	9140.47	3796.631
ENSMUSG(	684.8312	566.2771	755.6668	283.0796	288.2161	262.7245	668.925	278.0067
ENSMUSG(	117.0904	140.5206	107.9524	55.80421	46.55037	49.32375	121.8545	50.55944
ENSMUSG(	120.0422	130.034	122.7151	56.81884	38.6269	59.38982	124.2638	51.61185
ENSMUSG(	160.3843	166.7371	181.766	65.95044	70.32077	74.48893	169.6292	70.25338
ENSMUSG(	5419.612	6996.668	5447.444	2504.087	2499.854	2384.652	5954.575	2462.864
ENSMUSG(	183.0152	176.1751	226.0542	79.14052	82.20597	79.52196	195.0815	80.28949
ENSMUSG(	1300.786	1808.941	1303.733	641.2412	553.6523	608.9973	1471.153	601.2969
ENSMUSG(	52.1495	81.79558	70.12293	27.3948	28.72257	27.17839	68.02267	27.76525
ENSMUSG(	1793.746	2077.398	1864.716	788.3614	757.6815	794.213	1911.953	780.0853
ENSMUSG(	586.4359	438.3404	573.9008	219.1584	211.9527	220.447	532.8924	217.186

ENSMUSG(	59.03717	31.45984	42.44283	14.20471	21.78953	18.11893	44.31328	18.03772
ENSMUSG(	34.43835	38.80047	50.74686	15.21933	16.83737	18.11893	41.32856	16.72521
ENSMUSG(	215.4857	228.6082	231.5902	106.5353	69.33034	95.62768	225.228	90.49778
ENSMUSG(	256.8117	259.0193	308.1718	101.4622	109.9381	117.773	274.6676	109.7244
ENSMUSG(	317.8168	147.8612	278.6464	112.6231	90.12944	93.61446	248.1081	98.78898
ENSMUSG(	44.27788	42.99511	51.66953	13.19009	17.8278	24.15857	46.31417	18.39215
ENSMUSG(	429.0034	388.0047	420.7376	151.1787	170.3545	169.11	412.5819	163.5477
ENSMUSG(	135.7855	100.6715	132.8645	48.70186	52.49297	45.29732	123.1072	48.83072
ENSMUSG(	505.7518	525.3793	520.386	219.1584	198.0867	196.2884	517.1723	204.5111
ENSMUSG(	349.3033	332.4256	346.924	131.9009	138.6607	134.8854	342.8843	135.149
ENSMUSG(	54.11741	102.7688	52.5922	22.32169	26.7417	33.21804	69.82614	27.42714
ENSMUSG(	2121.402	2361.585	2199.646	944.6132	821.0693	859.6425	2227.544	875.1083
ENSMUSG(	153.4966	194.0023	143.9365	64.93581	66.35904	61.40303	163.8118	64.23263
ENSMUSG(	6287.459	6983.036	6506.67	2692.807	2492.921	2571.881	6592.388	2585.87
ENSMUSG(	78.71623	61.87102	74.73628	30.43866	31.69387	22.14536	71.77451	28.09263
ENSMUSG(	482.1369	328.231	453.9537	160.3103	170.3545	163.0704	421.4405	164.5784
ENSMUSG(	191.8708	212.8782	190.9927	83.19901	74.2825	74.48893	198.5806	77.32348
ENSMUSG(	226.3092	173.0291	234.3582	66.96506	91.11987	88.58143	211.2322	82.22212
ENSMUSG(	1240.765	1283.561	1031.545	453.5361	481.3506	444.9203	1185.29	459.9357
ENSMUSG(	6023.759	8600.071	6215.106	2742.523	2629.601	2707.773	6946.312	2693.299
ENSMUSG(	1355.887	1494.342	1308.346	494.121	546.7192	570.7462	1386.192	537.1955
ENSMUSG(	142.6732	91.23353	147.6272	46.67262	50.5121	50.33036	127.178	49.17169
ENSMUSG(	38.37416	37.75181	31.37078	17.24858	13.86607	10.06607	35.83225	13.7269
ENSMUSG(	226.3092	204.489	185.4567	65.95044	97.06247	72.47571	205.4183	78.49621
ENSMUSG(	1002.648	893.4594	1093.364	371.3517	352.5943	419.7552	996.4905	381.2337
ENSMUSG(	117.0904	58.72503	120.8698	35.51177	32.6843	45.29732	98.89507	37.83113
ENSMUSG(	913.1082	959.5251	926.3608	366.2786	348.6326	349.2927	932.998	354.7346
ENSMUSG(	1667.8	1828.865	1683.873	635.1534	659.6286	674.4268	1726.846	656.4029
ENSMUSG(	42.30997	44.04377	53.51487	18.2632	14.8565	20.13214	46.62287	17.75061
ENSMUSG(	198.7585	222.3162	218.6728	102.4768	82.20597	57.37661	213.2492	80.68647
ENSMUSG(	2012.184	2331.174	2057.554	802.5661	813.1458	802.2659	2133.637	805.9926
ENSMUSG(	163.3362	219.1702	182.6887	75.08203	63.38774	74.48893	188.3984	70.98623
ENSMUSG(	1030.199	764.4741	1020.473	316.5621	365.4699	372.4446	938.382	351.4922
ENSMUSG(	314.8649	266.36	368.1454	130.8862	107.9572	114.7532	316.4568	117.8656
ENSMUSG(	1108.915	1081.17	940.2009	403.8196	332.7856	418.7486	1043.429	385.1179
ENSMUSG(	1298.818	1346.481	1329.568	491.0771	521.9584	452.9732	1324.956	488.6696
ENSMUSG(	1773.083	1744.972	1734.62	632.1096	679.4373	625.103	1750.892	645.55
ENSMUSG(	1561.533	1871.86	1618.363	643.2704	601.1931	582.8255	1683.919	609.0963
ENSMUSG(	2465.786	2095.225	2207.95	824.8877	805.2224	818.3716	2256.32	816.1606
ENSMUSG(	370.9502	253.776	388.4441	123.7839	120.8329	121.7995	337.7235	122.1387
ENSMUSG(	21.64696	23.07055	27.6801	8.116977	7.923467	10.06607	24.13254	8.702172
ENSMUSG(	59.03717	55.57905	58.12822	20.29244	21.78953	20.13214	57.58148	20.73804
ENSMUSG(	65.92484	46.1411	58.12822	22.32169	14.8565	24.15857	56.73139	20.44559
ENSMUSG(	2077.124	1882.347	2067.704	731.5425	699.246	720.7307	2009.058	717.1731
ENSMUSG(	28.53463	39.84913	19.37607	10.14622	5.9426	15.09911	29.25328	10.39598
ENSMUSG(	48.21369	28.31386	59.05089	18.2632	17.8278	12.07929	45.19281	16.05676
ENSMUSG(	25.58277	24.11921	21.22141	11.16084	7.923467	6.039643	23.64113	8.374651
ENSMUSG(	360.1267	429.9511	407.8202	152.1933	139.6511	128.8457	399.2994	140.23
ENSMUSG(	2257.188	2854.456	2291.913	869.5311	870.591	858.6359	2467.852	866.2527
ENSMUSG(	80.68413	87.03889	112.5658	43.62875	26.7417	28.185	93.42959	32.85182

ENSMUSG(	891.4613	1134.652	924.5155	337.8692	330.8048	366.405	983.5428	345.0263
ENSMUSG(	1362.775	1565.651	1356.325	539.7789	495.2167	456.9996	1428.25	497.3318
ENSMUSG(	1220.102	1338.092	1121.044	430.1998	432.8194	407.6759	1226.413	423.565
ENSMUSG(	97.41133	155.2019	134.7098	42.61413	48.53124	42.2775	129.1077	44.47429
ENSMUSG(	83.63599	90.18487	98.7257	31.45328	34.66517	27.17839	90.84886	31.09895
ENSMUSG(	23.61487	28.31386	38.75215	7.102355	14.8565	9.059464	30.22696	10.33944
ENSMUSG(	626.778	513.844	627.4157	202.9244	215.9145	182.1959	589.3459	200.3449
ENSMUSG(	3855.127	5028.331	3890.9	1542.226	1348.97	1438.442	4258.119	1443.212
ENSMUSG(	71.82856	69.21165	87.65366	33.48253	23.7704	20.13214	76.23129	25.79502
ENSMUSG(	33.4544	32.5085	35.06146	13.19009	11.8852	9.059464	33.67479	11.37825
ENSMUSG(	24.59882	50.33574	32.29345	8.116977	11.8852	16.10571	35.74267	12.03596
novel.63	425.0676	376.4694	443.8043	530.6473	566.5279	533.5018	415.1138	543.559
ENSMUSG(	26.56673	42.99511	37.82948	16.23395	9.904334	10.06607	35.7971	12.06812
ENSMUSG(	43.29392	32.5085	58.12822	22.32169	13.86607	9.059464	44.64355	15.08241
ENSMUSG(	316.8328	383.81	331.2386	120.74	114.8903	111.7334	343.9605	115.7879
ENSMUSG(	402.4367	326.1337	397.6708	133.9301	124.7946	120.7929	375.4137	126.5059
ENSMUSG(	306.0093	379.6154	317.3985	108.5646	122.8137	105.6937	334.3411	112.3574
ENSMUSG(	211.5499	226.5108	205.7554	76.09666	57.44514	82.54178	214.6054	72.02786
ENSMUSG(	17.71115	36.70315	38.75215	14.20471	9.904334	7.04625	31.05548	10.3851
ENSMUSG(	20.66301	26.21653	31.37078	10.14622	8.913901	7.04625	26.08344	8.702124
ENSMUSG(	205.6461	219.1702	188.2247	81.16977	57.44514	65.42946	204.347	68.01479
ENSMUSG(	44.27788	28.31386	26.75743	6.087732	15.84693	11.07268	33.11639	11.00245
ENSMUSG(	105.283	122.6934	94.11235	33.48253	38.6269	34.22464	107.3629	35.44469
ENSMUSG(	22.63092	25.16787	22.14408	5.07311	8.913901	9.059464	23.31429	7.682158
ENSMUSG(	146.609	134.2286	134.7098	39.57026	40.60777	56.37	138.5158	45.51601
ENSMUSG(	3994.849	4511.341	4131.717	1370.754	1350.951	1356.906	4212.635	1359.537
ENSMUSG(	4264.452	3679.752	4538.614	1272.336	1292.516	1463.607	4160.939	1342.819
ENSMUSG(	719.2695	780.204	751.0535	260.7579	217.8953	244.6055	750.1757	241.0863
ENSMUSG(	222.3733	341.8636	254.657	74.06741	96.07204	92.60786	272.9646	87.58244
ENSMUSG(	208.598	181.4184	216.8275	73.05279	57.44514	64.42286	202.2813	64.97359
ENSMUSG(	197.7745	217.0729	159.6219	76.09666	56.4547	49.32375	191.4898	60.62504
ENSMUSG(	164.3201	134.2286	132.8645	41.59951	36.64604	58.38321	143.8044	45.54292
ENSMUSG(	311.913	350.2529	319.2439	113.6377	107.9572	88.58143	327.1366	103.3921
ENSMUSG(	80.68413	62.91968	75.65895	21.30706	28.72257	19.12554	73.08759	23.05172
ENSMUSG(	5979.481	6005.683	6427.32	1987.645	1870.929	1934.699	6137.495	1931.091
ENSMUSG(	439.8269	506.5034	476.0978	158.281	143.6128	144.9514	474.1427	148.9484
ENSMUSG(	43.29392	35.65448	48.90152	9.131599	10.89477	20.13214	42.61664	13.38617
ENSMUSG(	46.24578	23.07055	30.44811	8.116977	12.87563	10.06607	33.25482	10.35289
ENSMUSG(	5061.453	5530.64	5011.021	1672.097	1527.248	1601.512	5201.038	1600.286
ENSMUSG(	5618.371	7556.653	5756.539	1918.65	1881.823	2016.234	6310.521	1938.903
ENSMUSG(	85.6039	117.4501	110.7204	30.43866	31.69387	34.22464	104.5915	32.11906
ENSMUSG(	26.56673	18.8759	29.52544	5.07311	11.8852	6.039643	24.98936	7.665985
ENSMUSG(	12.79139	31.45984	24.91209	9.131599	3.961734	8.052857	23.05444	7.04873
ENSMUSG(	82.65204	40.89779	71.0456	27.3948	13.86607	18.11893	64.86514	19.79326
ENSMUSG(	30.50254	58.72503	42.44283	1.014622	21.78953	17.11232	43.89013	13.30549
ENSMUSG(	465.4097	230.7055	408.7429	95.37448	124.7946	114.7532	368.286	111.6408
ENSMUSG(	3197.847	3159.616	3108.476	945.6278	962.7013	933.1248	3155.313	947.1513
ENSMUSG(	191.8708	196.0997	197.4514	54.78959	75.27294	45.29732	195.1406	58.45328
ENSMUSG(	4592.108	5804.34	4774.818	1579.767	1463.861	1460.587	5057.089	1501.405
ENSMUSG(	2970.554	2979.247	2885.189	901.999	870.591	843.5368	2944.997	872.0423

ENSMUSG(	91.50761	105.9148	82.11764	25.36555	21.78953	35.23125	93.18002	27.46211
ENSMUSG(	17.71115	33.55716	22.14408	4.058488	10.89477	6.039643	24.4708	6.997633
ENSMUSG(	37.39021	45.09244	29.52544	9.131599	14.8565	8.052857	37.33603	10.68032
ENSMUSG(	869.8143	989.9363	825.7898	294.2404	240.6753	232.5262	895.1801	255.814
ENSMUSG(	15.74325	15.72992	21.22141	6.087732	4.952167	4.026428	17.56486	5.022109
ENSMUSG(	52.1495	44.04377	39.67482	11.16084	14.8565	12.07929	45.28936	12.69888
ENSMUSG(	555.9334	537.9632	579.4368	149.1494	161.4406	159.0439	557.7778	156.5447
ENSMUSG(	455.5702	559.9851	530.5353	155.2372	137.6702	140.925	515.3635	144.6108
ENSMUSG(	50.18159	25.16787	35.98413	7.102355	16.83737	7.04625	37.1112	10.32866
ENSMUSG(	103.315	99.62282	121.7925	26.38017	24.76083	39.25768	108.2434	30.1329
ENSMUSG(	7334.384	9079.31	7217.126	2314.353	2205.695	1989.056	7876.94	2169.701
ENSMUSG(	15.74325	26.21653	16.60806	8.116977	4.952167	3.019821	19.52261	5.362988
ENSMUSG(	60.02112	102.7688	64.58691	21.30706	21.78953	19.12554	75.79228	20.74071
ENSMUSG(	1920.676	1417.79	2046.482	483.9747	488.2837	501.2903	1794.983	491.1829
ENSMUSG(	262.7154	339.7663	304.4811	82.18439	91.11987	69.45589	302.3209	80.92005
ENSMUSG(	28.53463	37.75181	27.6801	7.102355	5.9426	12.07929	31.32218	8.374747
ENSMUSG(	1350.967	1165.063	1252.063	342.9423	330.8048	331.1737	1256.031	334.9736
ENSMUSG(	136.7694	169.8831	140.2459	55.80421	30.70344	32.21143	148.9661	39.57303
ENSMUSG(	417.196	484.4815	420.7376	126.8278	122.8137	100.6607	440.805	116.7674
ENSMUSG(	39.35811	34.60582	35.98413	6.087732	12.87563	10.06607	36.64936	9.676479
ENSMUSG(	1833.104	2255.67	1894.242	556.0129	531.8627	488.2045	1994.339	525.36
ENSMUSG(	169.2399	173.0291	141.1685	46.67262	34.66517	45.29732	161.1458	42.2117
ENSMUSG(	89.53971	70.26031	85.80832	30.43866	15.84693	18.11893	81.86945	21.46817
ENSMUSG(	215.4857	247.4841	214.9821	58.84808	62.3973	55.36339	225.984	58.86959
ENSMUSG(	942.6268	864.0969	926.3608	230.3192	246.6179	231.5196	911.0282	236.1523
ENSMUSG(	21.64696	28.31386	23.98942	7.102355	3.961734	8.052857	24.65008	6.372315
ENSMUSG(	81.66809	99.62282	80.2723	17.24858	20.7991	29.19161	87.18774	22.41309
ENSMUSG(	27.55068	54.53039	43.3655	9.131599	7.923467	15.09911	41.81552	10.71806
ENSMUSG(	20.66301	26.21653	20.29874	7.102355	2.9713	7.04625	22.39276	5.706635
ENSMUSG(	37.39021	42.99511	54.43754	11.16084	17.8278	5.033036	44.94095	11.34056
ENSMUSG(	34.43835	38.80047	46.13351	12.17546	7.923467	10.06607	39.79077	10.055
ENSMUSG(	64.94089	34.60582	84.88565	13.19009	22.77997	9.059464	61.47745	15.00984
ENSMUSG(	15.74325	22.02189	11.99471	6.087732	3.961734	2.013214	16.58661	4.020893
ENSMUSG(	19.67906	22.02189	12.91738	6.087732	5.9426	1.006607	18.20611	4.345647
ENSMUSG(	65.92484	71.30897	72.89094	29.42404	12.87563	8.052857	70.04158	16.78418
ENSMUSG(	129.8818	99.62282	100.571	30.43866	22.77997	25.16518	110.0252	26.12794
ENSMUSG(	54.11741	45.09244	66.43225	22.32169	9.904334	7.04625	55.21403	13.09076
ENSMUSG(	1025.279	876.6808	1056.457	222.2022	227.7997	246.6187	986.139	232.2069
ENSMUSG(	4221.158	4026.859	4141.866	1016.651	952.7969	911.9861	4129.961	960.4781
ENSMUSG(	6008.016	6854.05	6456.846	1488.451	1536.162	1467.633	6439.637	1497.415
ENSMUSG(	22.63092	26.21653	11.99471	6.087732	5.9426	2.013214	20.28072	4.681182
ENSMUSG(	21.64696	13.6326	29.52544	6.087732	6.933034	2.013214	21.60167	5.011327
ENSMUSG(	44.27788	39.84913	63.66424	18.2632	10.89477	5.033036	49.26375	11.397
ENSMUSG(	259.7635	187.7104	205.7554	49.71648	43.57907	57.37661	217.7431	50.22405
ENSMUSG(	338.4798	362.8368	248.1983	74.06741	83.19641	60.39643	316.5049	72.55341
ENSMUSG(	73.79646	40.89779	61.8189	18.2632	7.923467	14.0925	58.83772	13.42639
ENSMUSG(	79.70018	124.7907	107.0297	25.36555	26.7417	18.11893	103.8402	23.40873
ENSMUSG(	47.22974	59.77369	35.98413	12.17546	8.913901	11.07268	47.66252	10.72068
ENSMUSG(	21.64696	32.5085	26.75743	13.19009	0.990433	4.026428	26.97097	6.068983
ENSMUSG(	23.61487	29.36252	23.06675	1.014622	9.904334	6.039643	25.34805	5.652866

ENSMUSG(	142.6732	142.6179	130.0965	22.32169	33.67474	36.23786	138.4625	30.74476
ENSMUSG(	19.67906	15.72992	14.76272	6.087732	0.990433	4.026428	16.7239	3.701531
ENSMUSG(	49.19764	12.58394	65.50958	11.16084	6.933034	10.06607	42.43039	9.386649
ENSMUSG(	151.5287	167.7858	191.9154	42.61413	31.69387	38.25107	170.41	37.51969
ENSMUSG(	52.1495	55.57905	57.20555	16.23395	9.904334	10.06607	54.97803	12.06812
ENSMUSG(	17.71115	18.8759	18.4534	5.07311	4.952167	2.013214	18.34682	4.012831
ENSMUSG(	58.05322	49.28708	48.90152	7.102355	15.84693	11.07268	52.08061	11.34066
ENSMUSG(	350.2872	214.9756	315.5532	70.00892	53.4834	68.44928	293.6053	63.98054
ENSMUSG(	18.6951	41.94645	13.84005	7.102355	1.980867	7.04625	24.8272	5.37649
ENSMUSG(	15.74325	14.68126	11.99471	3.043866	1.980867	4.026428	14.13974	3.017054
ENSMUSG(	443.7627	677.4352	534.226	103.4915	122.8137	124.8193	551.808	117.0415
ENSMUSG(	20.66301	17.82724	23.06675	2.029244	6.933034	4.026428	20.519	4.329569
ENSMUSG(	1165	1416.741	1193.012	281.0503	292.1779	222.4602	1258.251	265.2294
ENSMUSG(	418.18	386.956	413.3562	76.09666	79.23467	93.61446	406.1641	82.98193
ENSMUSG(	381.7737	375.4207	392.1348	78.1259	86.16771	68.44928	383.1097	77.58096
ENSMUSG(	26.56673	17.82724	24.91209	4.058488	6.933034	3.019821	23.10202	4.670448
ENSMUSG(	294.2019	254.8247	266.6517	57.83346	46.55037	59.38982	271.8928	54.59122
ENSMUSG(	53.13345	23.07055	71.96827	12.17546	4.952167	12.07929	49.39076	9.735639
ENSMUSG(	51.16555	73.40629	52.5922	15.21933	8.913901	10.06607	59.05468	11.39977
ENSMUSG(	43.29392	35.65448	40.59749	9.131599	7.923467	6.039643	39.84863	7.698236
ENSMUSG(	91.50761	87.03889	110.7204	18.2632	23.7704	13.08589	96.42231	18.37316
ENSMUSG(	31.48649	31.45984	34.13879	4.058488	2.9713	11.07268	32.36171	6.034156
ENSMUSG(	225.3252	236.9975	197.4514	64.93581	30.70344	27.17839	219.9247	40.93921
ENSMUSG(	16.7272	13.6326	23.98942	6.087732	1.980867	2.013214	18.11641	3.360605
ENSMUSG(	191.8708	233.8515	240.8169	33.48253	49.52167	39.25768	222.1797	40.75396
ENSMUSG(	358.1588	408.9779	364.4547	44.64337	73.29207	75.49553	377.1971	64.47699
ENSMUSG(	1054.797	1397.866	1143.188	206.9829	195.1154	208.3677	1198.617	203.4887
ENSMUSG(	12.79139	31.45984	22.14408	6.087732	1.980867	3.019821	22.13177	3.69614
ENSMUSG(	104.299	134.2286	79.34963	19.27782	13.86607	19.12554	105.9591	17.42314
ENSMUSG(	23.61487	6.291968	19.37607	2.029244	1.980867	4.026428	16.42764	2.678846
ENSMUSG(	99.37924	96.47684	90.42167	16.23395	13.86607	16.10571	95.42592	15.40191
ENSMUSG(	2057.445	2589.145	2091.693	363.2347	363.4891	352.3125	2246.094	359.6788
ENSMUSG(	164.3201	178.2724	184.534	37.54102	25.75127	21.13875	175.7089	28.14368
ENSMUSG(	43.29392	36.70315	45.21084	3.043866	13.86607	3.019821	41.73597	6.643252
ENSMUSG(	31.48649	20.97323	35.98413	4.058488	4.952167	5.033036	29.48128	4.68123
ENSMUSG(	27.55068	4.194645	12.91738	3.043866	2.9713	1.006607	14.88757	2.340591
ENSMUSG(	73.79646	69.21165	87.65366	12.17546	10.89477	13.08589	76.88726	12.05204
ENSMUSG(	518.5431	614.5155	562.8288	87.2575	96.07204	81.53518	565.2958	88.28824
ENSMUSG(	353.2391	317.7444	381.0628	62.90657	47.5408	53.35018	350.6821	54.59918
ENSMUSG(	578.5643	869.3402	670.7812	136.974	102.0146	89.58803	706.2286	109.5256
ENSMUSG(	20.66301	7.340629	11.07204	2.029244	0.990433	3.019821	13.02523	2.013166
ENSMUSG(	12897.65	11324.49	13599.23	1798.925	2047.226	1963.89	12607.13	1936.68
ENSMUSG(	12690.04	16296.2	12656.27	2199.701	2155.183	2002.142	13880.83	2119.008
ENSMUSG(	72.81251	57.67637	82.11764	15.21933	11.8852	5.033036	70.86884	10.71252
ENSMUSG(	15.74325	24.11921	13.84005	3.043866	2.9713	2.013214	17.90084	2.676127
ENSMUSG(	300.1056	276.8466	299.8678	66.96506	32.6843	31.20482	292.2733	43.61806
ENSMUSG(	19.67906	15.72992	11.99471	3.043866	1.980867	2.013214	15.80123	2.345982
ENSMUSG(	23.61487	40.89779	37.82948	4.058488	4.952167	6.039643	34.11404	5.016766
ENSMUSG(	1282.091	1557.262	1334.181	221.1876	179.2684	203.3346	1391.178	201.2636
ENSMUSG(	39.35811	57.67637	57.20555	2.029244	13.86607	6.039643	51.41334	7.311651

ENSMUSG(	105.283	111.1581	94.11235	14.20471	14.8565	15.09911	103.5178	14.72011
ENSMUSG(	11.80743	24.11921	21.22141	2.029244	4.952167	1.006607	19.04935	2.662673
ENSMUSG(	234.1808	249.5814	214.0595	28.40942	27.73214	41.27089	232.6072	32.47082
ENSMUSG(	846.1994	942.7465	835.9391	132.9155	129.7468	103.6805	874.9617	122.1143
ENSMUSG(	558.8852	782.3013	670.7812	104.5061	76.26337	96.63428	670.6559	92.46791
ENSMUSG(	60.02112	98.57416	85.80832	7.102355	8.913901	17.11232	81.46787	11.04286
ENSMUSG(	235.1647	283.1386	211.2915	37.54102	38.6269	22.14536	243.1982	32.77109
ENSMUSG(	434.9072	479.2382	489.0152	63.92119	50.5121	72.47571	467.7202	62.303
ENSMUSG(	583.484	502.3088	521.3086	82.18439	68.3399	62.40964	535.7005	70.97798
ENSMUSG(	70.8446	106.9635	88.57633	12.17546	12.87563	10.06607	88.7948	11.70572
ENSMUSG(	314.8649	338.7176	343.2333	43.62875	39.61734	45.29732	332.2719	42.8478
ENSMUSG(	3755.748	4786.09	3962.868	546.8813	519.9775	536.5216	4168.235	534.4601
ENSMUSG(	468.3616	497.0655	493.6285	60.87732	60.41644	58.38321	486.3518	59.89232
ENSMUSG(	62.97298	74.45495	62.74157	7.102355	6.933034	10.06607	66.72317	8.03382
ENSMUSG(	261.7315	282.0899	254.657	39.57026	28.72257	26.17179	266.1594	31.4882
ENSMUSG(	9.839528	9.437952	23.06675	1.014622	0.990433	3.019821	14.11474	1.674959
ENSMUSG(	1991.521	1609.695	1938.53	185.6758	226.8092	240.5791	1846.582	217.6881
ENSMUSG(	18.6951	17.82724	14.76272	2.029244	2.9713	1.006607	17.09502	2.002384
ENSMUSG(	610.0508	617.6615	679.0852	71.02355	74.2825	78.51536	635.5992	74.60714
ENSMUSG(	41.32602	47.18976	32.29345	4.058488	5.9426	4.026428	40.26974	4.675839
ENSMUSG(	197.7745	157.2992	190.07	28.40942	14.8565	19.12554	181.7146	20.79715
ENSMUSG(	30.50254	49.28708	35.06146	3.043866	4.952167	5.033036	38.28369	4.343023
ENSMUSG(	52.1495	57.67637	47.05618	6.087732	1.980867	9.059464	52.29402	5.709354
ENSMUSG(	29.51859	60.82236	38.75215	5.07311	6.933034	2.013214	43.03103	4.673119
ENSMUSG(	569.7087	430.9998	489.9378	65.95044	48.53124	46.30393	496.8821	53.5952
ENSMUSG(	18.6951	7.340629	29.52544	0	3.961734	2.013214	18.52039	1.991649
ENSMUSG(	2682.255	3570.692	2664.671	299.3135	347.6421	306.0086	2972.539	317.6547
ENSMUSG(	185.9671	220.2189	167.926	24.35093	13.86607	23.15196	191.3706	20.45632
ENSMUSG(	20.66301	14.68126	11.99471	0	2.9713	2.013214	15.77966	1.661505
ENSMUSG(	784.2104	743.5009	781.5016	81.16977	93.10074	67.44268	769.7376	80.57106
ENSMUSG(	114.1385	116.4014	120.8698	10.14622	9.904334	16.10571	117.1366	12.05209
ENSMUSG(	334.544	332.4256	317.3985	17.24858	47.5408	34.22464	328.1227	33.00467
ENSMUSG(	1335.224	1674.712	1376.624	153.2079	136.6798	149.9845	1462.187	146.6241
ENSMUSG(	18.6951	24.11921	17.53073	2.029244	0.990433	3.019821	20.11502	2.013166
ENSMUSG(	1056.765	1176.598	1004.788	113.6377	108.9477	98.6475	1079.384	107.0776
ENSMUSG(	266.6512	241.1921	256.5023	35.51177	16.83737	22.14536	254.7819	24.8315
ENSMUSG(	433.9232	351.3015	381.9854	24.35093	46.55037	35.23125	389.0701	35.37752
ENSMUSG(	237.1326	236.9975	214.0595	15.21933	13.86607	30.19821	229.3965	19.7612
ENSMUSG(	27.55068	41.94645	47.05618	2.029244	4.952167	3.019821	38.8511	3.333744
ENSMUSG(	32.47044	34.60582	39.67482	1.014622	5.9426	2.013214	35.58369	2.990146
ENSMUSG(	4535.039	4527.071	4327.323	411.9366	351.6039	345.2662	4463.144	369.6022
ENSMUSG(	23.61487	12.58394	14.76272	0	3.961734	0	16.98718	1.320578
ENSMUSG(	298.1377	299.9171	308.1718	25.36555	22.77997	22.14536	302.0756	23.43029
ENSMUSG(	25.58277	7.340629	33.21612	0	4.952167	0	22.04651	1.650722
ENSMUSG(	139.7213	120.596	166.0806	8.116977	8.913901	14.0925	142.1327	10.37446
ENSMUSG(	49.19764	72.35763	46.13351	8.116977	1.980867	2.013214	55.89626	4.037019
ENSMUSG(	64.94089	55.57905	75.65895	4.058488	8.913901	1.006607	65.39296	4.659665
ENSMUSG(	627.7619	479.2382	613.5756	32.46791	39.61734	49.32375	573.5253	40.46966
ENSMUSG(	6.88767	11.53527	10.14937	0	0.990433	1.006607	9.524105	0.66568
ENSMUSG(	35.4223	24.11921	26.75743	2.029244	1.980867	2.013214	28.76632	2.007775

ENSMUSG(	18.6951	7.340629	17.53073	1.014622	0.990433	1.006607	14.52216	1.003888
ENSMUSG(	377.8379	552.6445	464.1031	38.55564	28.72257	24.15857	464.8618	30.47893
ENSMUSG(	49.19764	62.91968	64.58691	3.043866	6.933034	1.006607	58.90141	3.661169
ENSMUSG(	11.80743	11.53527	9.226701	0	0	2.013214	10.85647	0.671071
ENSMUSG(	29.51859	27.26519	31.37078	4.058488	0.990433	0	29.38485	1.682974
ENSMUSG(	10.82348	15.72992	9.226701	0	1.980867	0	11.9267	0.660289
ENSMUSG(	71.82856	145.7639	87.65366	8.116977	3.961734	5.033036	101.7487	5.703915
ENSMUSG(	24.59882	24.11921	27.6801	2.029244	0	2.013214	25.46604	1.347486
ENSMUSG(	175.1436	178.2724	128.2511	4.058488	11.8852	9.059464	160.5557	8.334384
ENSMUSG(	479.185	626.0508	457.6444	22.32169	28.72257	27.17839	520.9601	26.07422
ENSMUSG(	1168.936	1609.695	1195.78	68.9943	58.43557	71.46911	1324.804	66.29966
ENSMUSG(	61.00508	57.67637	50.74686	1.014622	2.9713	4.026428	56.4761	2.670784
ENSMUSG(	1345.064	1828.865	1389.541	60.87732	60.41644	84.555	1521.157	68.61625
ENSMUSG(	213.5178	245.3867	227.8995	6.087732	8.913901	15.09911	228.9347	10.03358
ENSMUSG(	360.1267	472.9462	341.3879	18.2632	12.87563	20.13214	391.487	17.09032
ENSMUSG(	26.56673	39.84913	26.75743	1.014622	0.990433	2.013214	31.05776	1.339423
ENSMUSG(	522.479	618.7102	481.6338	25.36555	31.69387	11.07268	540.941	22.7107
ENSMUSG(	7.871623	15.72992	3.690681	0	0	1.006607	9.097408	0.335536
ENSMUSG(	278.4587	350.2529	305.4038	18.2632	2.9713	15.09911	311.3718	12.1112
ENSMUSG(	748.7881	570.4717	703.0746	21.30706	22.77997	30.19821	674.1115	24.76175
ENSMUSG(	2521.871	2556.636	2402.633	89.28674	83.19641	92.60786	2493.713	88.36367
ENSMUSG(	9.839528	10.48661	11.07204	1.014622	0	0	10.46606	0.338207
ENSMUSG(	6.88767	12.58394	11.99471	0	0	1.006607	10.48877	0.335536
ENSMUSG(	59.03717	65.017	59.97356	3.043866	0.990433	2.013214	61.34258	2.015838
ENSMUSG(	266.6512	367.0315	214.9821	10.14622	8.913901	7.04625	282.8883	8.702124
ENSMUSG(	20.66301	36.70315	20.29874	1.014622	0	1.006607	25.8883	0.673743
ENSMUSG(	225.3252	195.051	245.4303	4.058488	11.8852	1.006607	221.9355	5.650099
ENSMUSG(	27.55068	11.53527	17.53073	0	0.990433	0	18.87223	0.330144
ENSMUSG(	16.7272	25.16787	29.52544	0	0.990433	0	23.80684	0.330144
ENSMUSG(	29.51859	28.31386	29.52544	1.014622	0	0	29.11929	0.338207
ENSMUSG(	4116.859	5859.919	4361.462	45.65799	45.55994	44.29071	4779.413	45.16955

log2FoldCh	pvalue	padj	gene_name	gene_chr	gene_start	gene_end	gene_stran
-3.70764	0.001721732	0.021867964	Entpd4b	14	69555424	69585520	+
-3.27283	0.001233866	0.016789848	Hspb7	4	1.41E+08	1.41E+08	+
-2.38327	0.003864856	0.042463606	Dclk2	3	86786151	86920852	-
-2.10167	0.002340885	0.027921069	Slc7a15	12	8528483	8599066	-
-1.79113	0.000918777	0.013183092	Gm38091	9	89259525	89263395	-
-1.77712	0.000201204	0.003534357	Trav13n-4	14	53362267	53364106	+
-1.73473	0.000536916	0.008321907	Gm38248	1	1.28E+08	1.28E+08	-
-1.68683	0.002250524	0.027111054	Gm45820	8	75037434	75045246	-
-1.66641	3.09E-05	0.000667585	Nanos1	19	60755987	60759913	+
-1.62639	1.27E-05	0.00029831	Gm14221	2	1.61E+08	1.61E+08	-
-1.61345	8.87E-05	0.001719993	Dach1	14	97786853	98169765	-
-1.59156	0.000380847	0.006236426	Srpk3	X	73774405	73778925	+
-1.58473	0.002775397	0.032099802	Slc6a4	11	76998603	77032340	+
-1.56375	0.001642805	0.020994291	Gm28800	1	1.28E+08	1.28E+08	+
-1.45074	9.85E-08	3.30E-06	Nptx1	11	1.2E+08	1.2E+08	-
-1.39129	3.60E-05	0.000764873	Smpd3	8	1.06E+08	1.06E+08	-
-1.33203	1.50E-06	4.17E-05	Gm37168	1	1.91E+08	1.91E+08	+
-1.31913	0.004651733	0.049189709	Pgm5	19	24683016	24861855	-
-1.30071	3.03E-16	2.19E-14	Hmga2	10	1.2E+08	1.2E+08	-
-1.28536	0.000101266	0.001930253	Fam78b	1	1.67E+08	1.67E+08	+
-1.25634	0.00233221	0.02784052	Cmbl	15	31565389	31590119	+
-1.22547	3.95E-05	0.00083321	Trav13-4-d	14	53757356	53757921	+
-1.22498	5.23E-05	0.001069671	Chst3	10	60181532	60219260	-
-1.20549	5.01E-06	0.00012712	Slco2b1	7	99657804	99711340	-
-1.17324	0.000832223	0.012157837	Klhl3	13	58000228	58113592	-
-1.16346	5.53E-06	0.000139605	Slc24a3	2	1.45E+08	1.46E+08	+
-1.10013	4.68E-05	0.000966687	Prokr1	6	87578591	87590743	-
-1.06316	7.95E-15	5.39E-13	Efr3b	12	3962554	4038915	-
-1.05661	1.03E-06	2.92E-05	Dnmt3aos	12	3859293	3862244	-
-1.05275	0.002048581	0.025095549	Sox4	13	28948919	28953713	-
-1.04941	0.001261767	0.017057398	Fabp7	10	57784881	57788450	+
-1.04189	0.002905005	0.033235015	Gm26569	16	29907519	29946754	-
-1.03953	0.0001004	0.001919529	Etl4	2	19909780	20810713	+
-1.03344	1.34E-25	1.59E-23	Angptl2	2	33216069	33247717	+
-1.03101	0.000138624	0.002542952	Gm9821	2	91945703	91947655	+
-1.02791	0.000504539	0.007904621	Gm16907	13	63289601	63297147	-
-1.02754	3.09E-16	2.23E-14	Tox2	2	1.63E+08	1.63E+08	+
-1.01925	2.56E-07	8.14E-06	Xylt1	7	1.17E+08	1.18E+08	+
-1.01566	0.000144922	0.002648445	Lrrc39	3	1.17E+08	1.17E+08	+
-0.99713	4.22E-15	2.91E-13	Rasgrp3	17	75435905	75529043	+
-0.98916	4.59E-10	2.05E-08	Fam214a	9	74952884	75032468	+
-0.98836	1.23E-06	3.45E-05	Shisa9	16	11984113	12270902	+
-0.98423	9.63E-05	0.001856284	Npas4	19	4984355	4989971	-
-0.96409	0.000179553	0.003192744	Gm17828	9	72292542	72293340	-
-0.94432	0.000415925	0.006675072	Slc9b2	3	1.35E+08	1.35E+08	+
-0.9365	0.001509469	0.019689676	Gabbr2	4	46662305	46991873	-
-0.92399	4.71E-06	0.000120057	Fgfr1	8	25513654	25575718	+
-0.91293	0.001826882	0.022882607	Cmah	13	24327404	24477377	+
-0.90948	4.34E-09	1.74E-07	Arap2	5	62602445	62766159	-

-0.90406	5.66E-17	4.29E-15	Fzd7		1	59482424	59486955	+
-0.89707	0.00434223	0.046613039	Ticrr		7	79660196	79698148	+
-0.88764	2.30E-19	2.01E-17	Mamdc2		19	23302609	23448322	-
-0.88693	4.83E-05	0.000994448	Cdca7		2	72476159	72486893	+
-0.88549	9.39E-05	0.001816415	Kit		5	75574916	75656722	+
-0.88449	6.12E-30	8.79E-28	Emp1		6	1.35E+08	1.35E+08	+
-0.88256	3.44E-09	1.40E-07	Rhoj		12	75308322	75401456	+
-0.87538	0.000180215	0.00320058	Alox12		11	70241457	70255353	-
-0.87492	0.002385193	0.028332959	Slc18a1		8	69037711	69089235	-
-0.87035	0.003023232	0.034272964	Jcad		18	4634929	4682868	+
-0.86602	1.64E-05	0.000377408	F730311O2		1	1.32E+08	1.32E+08	+
-0.85713	5.37E-09	2.11E-07	Arhgap6	X		1.69E+08	1.69E+08	+
-0.85554	0.000357067	0.005873566	Gm30329		8	79225930	79233224	+
-0.83765	0.002497999	0.029361569	Gp5		16	30307685	30310779	-
-0.83601	0.000959962	0.013652381	Npas2		1	39193731	39363236	+
-0.82324	1.10E-15	7.76E-14	Ccnd1		7	1.45E+08	1.45E+08	-
-0.82239	0.000919896	0.013186081	C030034L1		3	9403064	9437233	+
-0.82237	2.27E-09	9.41E-08	Gprc5b		7	1.19E+08	1.19E+08	-
-0.81305	0.00308753	0.034929339	Fam171a1		2	3114224	3227806	+
-0.80932	0.002699152	0.031418568	Gm5805		15	81972390	81972845	+
-0.80716	6.13E-07	1.80E-05	Gm13391		2	6472000	6478619	+
-0.80562	0.004149089	0.044972773	Sez6l2		7	1.27E+08	1.27E+08	+
-0.79784	7.14E-08	2.43E-06	P2ry1		3	61002795	61008982	+
-0.79672	0.000167625	0.003013919	Ptchd1	X		1.56E+08	1.56E+08	-
-0.79182	1.12E-05	0.000265476	Lrp1		10	1.28E+08	1.28E+08	-
-0.7891	0.001438062	0.019014956	Sncaip		18	52767709	52915935	+
-0.7847	0.000525298	0.008176825	Hdac9		12	34047580	34917095	-
-0.78348	3.60E-12	1.99E-10	Egr2		10	67535475	67542188	+
-0.77208	6.00E-10	2.63E-08	Apbb2		5	66298703	66618784	-
-0.76957	0.000985368	0.013958894	Adcy9		16	4287529	4420498	-
-0.7678	3.92E-09	1.59E-07	Serpinb6b		13	32965209	32979067	+
-0.76359	0.000341044	0.005642019	Dennd2c		3	1.03E+08	1.03E+08	+
-0.75885	0.000285263	0.004801424	Syne1		10	5020917	5551482	-
-0.75815	0.002323965	0.027787877	Syne3		12	1.05E+08	1.05E+08	-
-0.75631	6.33E-09	2.46E-07	Tspan13		12	36014557	36042500	-
-0.75422	7.76E-06	0.00018953	Abca9		11	1.1E+08	1.1E+08	-
-0.74538	1.46E-21	1.47E-19	Plk2		13	1.1E+08	1.1E+08	+
-0.74245	0.00376247	0.041527587	Samd4		14	46882854	47105815	+
-0.73954	2.35E-05	0.000518819	Piezo1		8	1.22E+08	1.23E+08	-
-0.7363	3.40E-07	1.05E-05	Abcb4		5	8893717	8959231	+
-0.73462	2.42E-13	1.47E-11	Ndrg1		15	66929318	66969640	-
-0.7346	6.27E-06	0.000156712	Reps2	X		1.62E+08	1.63E+08	-
-0.73398	1.96E-07	6.35E-06	Gm10419		5	1.08E+08	1.08E+08	+
-0.73386	0.000294359	0.004948783	Tmem158		9	1.23E+08	1.23E+08	-
-0.73212	0.00280002	0.03225587	Ankrd55		13	1.12E+08	1.12E+08	+
-0.73134	1.98E-05	0.000447111	Mmp27		9	7571396	7581885	+
-0.73055	0.003748	0.041430978	Dtl		1	1.92E+08	1.92E+08	-
-0.72667	2.09E-05	0.000467516	Frmd6		12	70825514	70902234	+
-0.72493	0.000149815	0.002734414	Kitl		10	1E+08	1E+08	+
-0.72466	2.68E-07	8.50E-06	P2ry10b	X		1.07E+08	1.07E+08	+

-0.7239	1.67E-19	1.47E-17	Mmp12		9	7344381	7369499	+
-0.72291	6.53E-05	0.001313212	Dhrs9		2	69380445	69404533	+
-0.72221	0.002003217	0.024747636	Ankdd1a		9	65488470	65520193	-
-0.7187	8.48E-07	2.45E-05	Hip1		5	1.35E+08	1.36E+08	-
-0.71359	6.23E-07	1.82E-05	Gpr176		2	1.18E+08	1.18E+08	-
-0.7131	4.50E-08	1.56E-06	Cgnl1		9	71626509	71771602	-
-0.71143	5.62E-07	1.67E-05	Anapc15		7	1.02E+08	1.02E+08	+
-0.7101	7.90E-05	0.001551242	Cpeb2		5	43233170	43289724	+
-0.70767	2.32E-09	9.60E-08	Havcr2		11	46454935	46481255	+
-0.70697	9.29E-08	3.13E-06	Itgb3		11	1.05E+08	1.05E+08	+
-0.7065	1.30E-11	6.91E-10	Dot1l		10	80755206	80795461	+
-0.70185	5.13E-10	2.28E-08	Ankrd13b		11	77470485	77489678	-
-0.70102	0.001614266	0.020757716	Lonrf3	X		36328353	36362341	+
-0.69772	4.00E-13	2.37E-11	Il7r		15	9505788	9530176	-
-0.69373	9.20E-08	3.11E-06	Gm42517		5	1.08E+08	1.08E+08	-
1.162196	3.46E-27	4.36E-25	-		17	34995779	35000396	-
-0.69157	2.52E-05	0.000552274	Osbpl8		10	1.11E+08	1.11E+08	+
-0.69106	4.55E-06	0.000116642	Cacna1a		8	84388440	84640246	+
-0.69054	1.02E-19	9.11E-18	Atp6v0d2		4	19876841	19922605	-
-0.68895	0.000109647	0.00206633	Mfap3l		8	60632827	60676729	+
-0.68101	3.69E-09	1.50E-07	Mtss1		15	58941234	59082005	-
-0.67687	8.36E-20	7.58E-18	Cd36		5	17781690	17888801	-
-0.67324	0.000848913	0.012327095	Peak1		9	56201126	56418067	-
-0.67314	4.53E-15	3.11E-13	Lpl		8	68880491	68907448	+
-0.67029	1.20E-05	0.000282527	Mctp1		13	76384535	77031810	+
-0.66528	2.34E-05	0.000518595	Chst11		10	82985498	83195900	+
-0.66272	9.17E-07	2.64E-05	Cd28		1	60716800	60773359	+
-0.66094	1.62E-05	0.000373771	Ppbp		5	90768518	90770063	+
-0.6562	5.41E-07	1.61E-05	Rps6ka2		17	7170115	7303315	+
-0.65077	0.003170155	0.035805268	Plxna3	X		74329066	74344689	+
-0.65053	0.000188481	0.003335116	Jmjd1c		10	67096125	67256326	+
-0.65048	7.23E-13	4.24E-11	Myo1e		9	70207350	70399766	+
-0.64743	0.000606697	0.00917771	Ets1		9	32636221	32757820	+
-0.64736	1.99E-05	0.00044728	C5ar2		7	16234585	16244154	-
-0.64561	3.29E-05	0.000705942	Abcg3		5	1.05E+08	1.05E+08	-
-0.64413	3.49E-06	9.20E-05	Sulf2		2	1.66E+08	1.66E+08	-
-0.64372	4.45E-18	3.53E-16	Adam8		7	1.4E+08	1.4E+08	-
-0.64166	0.000615239	0.009277883	Angptl4		17	33773750	33781575	-
-0.63677	0.001283651	0.017288724	Lrp8		4	1.08E+08	1.08E+08	+
-0.63411	6.39E-06	0.000159092	Asph		4	9448069	9669344	-
-0.63353	1.59E-13	9.75E-12	Plau		14	20836660	20843385	+
-0.63024	6.41E-05	0.001296327	Myc		15	61985391	61990374	+
-0.62864	6.30E-05	0.001276922	Kif23		9	61915905	61946774	-
-0.62815	4.47E-12	2.45E-10	Arhgap25		6	87458545	87533259	-
-0.62626	0.004733988	0.049748724	Tmod1		4	46038935	46116032	+
-0.62602	0.000622048	0.009361083	Wee1		7	1.1E+08	1.1E+08	+
-0.62562	0.000395857	0.006424136	Kctd12b	X		1.54E+08	1.54E+08	-
-0.62408	0.000372735	0.00611049	Gm15513		5	34211810	34213802	-
-0.6203	2.30E-07	7.39E-06	Zranb3		1	1.28E+08	1.28E+08	-
-0.61593	0.002215515	0.026755558	Hyal1		9	1.08E+08	1.08E+08	+

-0.6155	8.89E-06	0.000214041	Ercc6		14	32513521	32580990	+
-0.61394	3.10E-07	9.64E-06	Mrgpre		7	1.44E+08	1.44E+08	-
-0.61119	8.11E-06	0.000197206	Dusp4		8	34807297	34819894	+
-0.61059	0.002634489	0.030789526	Bcl2		1	1.07E+08	1.07E+08	-
-0.60926	2.00E-05	0.000449876	Adra1a		14	66635251	66771168	+
-0.60893	0.000811407	0.011901725	Epas1		17	86753907	86833410	+
-0.60769	4.23E-05	0.000885454	Mkl2		16	13256481	13417529	+
-0.60194	6.96E-07	2.02E-05	Fam198b		3	79884533	79946280	+
-0.60011	3.02E-08	1.09E-06	Tbc1d2		4	46604390	46650209	-
-0.59973	4.22E-07	1.30E-05	Zfyve28		5	34194893	34288449	-
-0.59712	0.000545209	0.008423424	Irs2		8	10984681	11008458	-
-0.59576	9.70E-10	4.20E-08	Atf3		1	1.91E+08	1.91E+08	-
-0.59473	0.000990702	0.013997417	Tm4sf19		16	32400506	32408227	+
-0.59403	4.05E-09	1.63E-07	Irf2bpl		12	86880701	86884798	-
-0.59339	0.000576655	0.008833909	Gm21399		8	1.29E+08	1.29E+08	+
-0.59329	8.55E-12	4.59E-10	Rgs1		1	1.44E+08	1.44E+08	-
-0.59311	2.83E-07	8.90E-06	Alcam		16	52248996	52454074	-
-0.59296	2.22E-05	0.000494295	Tns1		1	73910231	74124449	-
-0.59222	3.90E-05	0.000823441	Eif2ak4		2	1.18E+08	1.18E+08	+
-0.59213	0.000333647	0.005532288	Cpeb4		11	31872211	31935634	+
-0.59204	1.04E-09	4.44E-08	S1pr1		3	1.16E+08	1.16E+08	-
-0.59106	8.77E-05	0.001702921	Otud7b		3	96104527	96161129	+
-0.58934	5.18E-09	2.04E-07	Padi2		4	1.41E+08	1.41E+08	+
-0.58437	0.000852572	0.012367836	Chn2		6	54039554	54301810	+
-0.58303	0.00172332	0.021868957	Nkain1		4	1.31E+08	1.31E+08	-
-0.57985	3.89E-12	2.14E-10	Slc6a8	X		73673150	73682502	+
-0.57877	2.66E-11	1.35E-09	Daglb		5	1.43E+08	1.44E+08	+
-0.57786	7.07E-09	2.73E-07	Bhlhe40		6	1.09E+08	1.09E+08	+
-0.57771	8.89E-10	3.87E-08	Pparg		6	1.15E+08	1.15E+08	+
-0.5768	2.96E-06	7.86E-05	Cdk19		10	40339564	40483818	+
-0.57491	0.000488582	0.007687878	Nav1		1	1.35E+08	1.36E+08	-
-0.57382	0.002980609	0.033905015	Anks6		4	47015669	47057427	-
-0.57368	0.002014936	0.024830314	Heatr5a		12	51875871	51971321	-
-0.57338	0.002296889	0.027561707	Rhov		2	1.19E+08	1.19E+08	-
-0.5712	1.35E-05	0.000316516	Lrrc20		10	61475801	61582791	+
-0.56993	0.00017289	0.003093244	Slc39a10		1	46807544	46892852	-
-0.56976	1.47E-07	4.81E-06	Bsn		9	1.08E+08	1.08E+08	-
-0.56862	3.29E-05	0.000705607	Thbd		2	1.48E+08	1.48E+08	-
-0.56761	5.85E-07	1.73E-05	Etv5		16	22381309	22439719	-
-0.56582	0.000176343	0.003151123	Cnksr3		10	7119063	7212237	-
-0.56397	0.002409343	0.028526311	Nipa1		7	55977567	56019954	-
-0.56217	0.000902148	0.013008885	F7		8	13026034	13035809	+
-0.56061	5.19E-07	1.55E-05	Ank		15	27466677	27594909	+
-0.56032	1.80E-06	4.94E-05	Arhgap22		14	33214026	33369934	+
-0.56004	9.68E-09	3.67E-07	Pcna		2	1.32E+08	1.32E+08	-
-0.55929	0.000529477	0.008215392	Fgd4		16	16416917	16600549	-
-0.55287	0.000562081	0.008638047	Itgav		2	83724397	83806916	+
-0.55108	6.45E-05	0.001300006	Kcnj2		11	1.11E+08	1.11E+08	+
-0.5466	2.22E-10	1.03E-08	Pdgfb		15	79995874	80014977	-
-0.54599	7.81E-05	0.001535634	Pitpnc1		11	1.07E+08	1.07E+08	-

-0.54432	0.000459321	0.007266892	Galnt12		4	47091909	47123070	+
-0.54266	2.18E-05	0.000486592	Cblb		16	52031225	52208048	+
-0.54232	0.00026838	0.004554286	Dagla		19	10245265	10304877	-
-0.53863	0.003380114	0.037825959	Arsb		13	93771630	93943016	+
-0.53794	1.13E-08	4.26E-07	Elmo1		13	20090596	20608353	+
-0.53599	3.94E-09	1.59E-07	Pcp4l1		1	1.71E+08	1.71E+08	-
-0.53536	8.63E-05	0.001679029	Itpr3		17	27057304	27122223	+
-0.53464	0.000523254	0.008153755	Itga2b		11	1.02E+08	1.02E+08	-
-0.53353	1.87E-09	7.82E-08	Entpd1		19	40612366	40741602	+
-0.53341	1.65E-09	6.92E-08	Steap3		1	1.2E+08	1.2E+08	-
-0.53178	0.000394677	0.00641937	Uhrf1bp1l		10	89744991	89819871	+
-0.53041	0.00098304	0.013939541	Ogt	X		1.02E+08	1.02E+08	+
-0.52781	1.15E-08	4.34E-07	Mical1		10	41476314	41487032	+
-0.52673	2.33E-05	0.000516706	Lilrb4a		10	51490956	51496611	+
-0.52588	5.07E-05	0.001039132	Plscr4		9	92457373	92492456	+
-0.52549	0.000988186	0.013985152	Gm26947		10	60931991	60940942	+
-0.52464	1.78E-11	9.37E-10	Cd300lb		11	1.15E+08	1.15E+08	-
-0.52125	0.001158029	0.015862155	Gab3	X		74966843	75085458	-
-0.52114	0.000768955	0.011371111	Man2a2		7	80349097	80371375	-
-0.51949	3.78E-10	1.70E-08	Fam129b		2	32876114	32925254	+
-0.51668	0.004665055	0.049203768	Rtl6		15	84553398	84557823	-
-0.51579	0.001251428	0.016933424	Adcy3		12	4133103	4213525	+
-0.51555	0.002391829	0.028368837	Dpep2		8	1.06E+08	1.06E+08	-
-0.51498	2.96E-05	0.000642079	Sort1		3	1.08E+08	1.08E+08	+
-0.51452	3.67E-06	9.63E-05	Specc1		11	61956763	62223013	+
-0.51428	0.002482494	0.029272828	Nrp1		8	1.28E+08	1.29E+08	+
-0.51419	4.62E-12	2.51E-10	Atp6v0a1		11	1.01E+08	1.01E+08	+
-0.51337	0.001459248	0.019172638	Zfp715		7	43296197	43313294	-
-0.51001	0.000703578	0.010468435	Map3k20		2	72285637	72442610	+
-0.50889	2.68E-05	0.000585055	Slc25a13		6	6041218	6217173	-
-0.50876	0.000113645	0.002127834	Tmx4		2	1.35E+08	1.35E+08	-
1.960157	6.25E-14	3.92E-12	-		16	93121263	93142320	-
-0.50863	3.85E-08	1.35E-06	Dusp3		11	1.02E+08	1.02E+08	-
-0.50828	1.11E-07	3.69E-06	Lilr4b		10	51480632	51486703	+
-0.5073	0.003964373	0.043327064	Micall1		15	79108898	79136900	+
-0.50724	4.75E-06	0.000120769	Phka2	X		1.61E+08	1.61E+08	+
-0.507	0.000501303	0.007879487	Cdk14		5	4803391	5420312	-
-0.50657	3.52E-08	1.25E-06	Slc40a1		1	45908068	45926523	-
-0.5044	0.000550538	0.008496689	Tns3		11	8431652	8664535	-
-0.50259	0.000123182	0.002274076	Gdpd5		7	99381414	99461877	+
-0.50161	1.57E-09	6.62E-08	Ldlrap1		4	1.35E+08	1.35E+08	-
-0.49872	4.39E-06	0.000112806	Dnmt3a		12	3806007	3914443	+
-0.49628	0.002025479	0.024939033	Il6st		13	1.12E+08	1.13E+08	+
-0.49516	0.000122437	0.002267856	Igf2bp2		16	22059009	22163299	-
-0.49391	0.000263158	0.004481425	Cdk6		5	3341485	3531008	+
-0.49132	0.001250527	0.016933424	Cpeb3		19	37021291	37208601	-
-0.48911	0.002349703	0.027980191	Kdr		5	75932827	75978458	-
-0.48665	1.34E-10	6.32E-09	Ptpra		2	1.3E+08	1.31E+08	+
-0.486	0.000216753	0.003770932	Tmem154		3	84666192	84704575	+
-0.48408	9.72E-07	2.78E-05	Tlr13	X		1.06E+08	1.06E+08	+

-0.47992	5.84E-09	2.29E-07	Slc37a2	9	37227585	37255738	-
-0.47848	3.10E-07	9.64E-06	Itgax	7	1.28E+08	1.28E+08	+
-0.47823	0.001853238	0.023152696	Gm20056	16	32408536	32409054	+
-0.47806	0.000400035	0.006484687	Wwp1	4	19608303	19708993	-
-0.477	0.002040929	0.025042208	Acot11	4	1.07E+08	1.07E+08	-
-0.47642	5.53E-08	1.90E-06	Slc9a9	9	94669909	95230445	+
-0.47417	0.000183475	0.003254497	Abcd2	15	91145871	91191799	-
-0.4741	0.001418998	0.018831609	Yae1d1	13	17981330	17993349	-
-0.47393	0.000114615	0.002137716	Mcm2	6	88883474	88898780	-
-0.47343	0.002521681	0.029590452	Mthfs	9	89210676	89377713	+
-0.47332	1.23E-07	4.06E-06	Slc46a3	5	1.48E+08	1.48E+08	-
-0.47274	0.004376992	0.046942193	Noct	3	51224447	51251644	+
-0.47139	3.05E-07	9.53E-06	Rcbtb2	14	73123037	73207843	+
-0.47066	0.001894321	0.023625212	Slc23a2	2	1.32E+08	1.32E+08	-
-0.46901	4.36E-05	0.000908677	Rcan1	16	92391953	92470867	-
-0.46775	0.004379371	0.046942193	Kif21b	1	1.36E+08	1.36E+08	+
-0.46681	1.88E-08	6.95E-07	Gpr137b	13	13357620	13394014	-
-0.4664	6.67E-05	0.001339262	Amz1	5	1.41E+08	1.41E+08	+
-0.4657	7.61E-05	0.001502461	Dio2	12	90724552	90738438	-
-0.46497	1.92E-07	6.25E-06	Antxr2	5	97882783	98031043	-
-0.4632	0.001133896	0.015575756	Mfsd6	1	52656286	52727462	-
-0.46257	0.002899668	0.033218963	Myo5a	9	75071015	75223688	+
-0.45902	0.004458906	0.047688903	Herc3	6	58831465	58920398	+
-0.45893	0.000791077	0.01162706	Plcg1	2	1.61E+08	1.61E+08	+
-0.45858	1.78E-05	0.000405119	Clip1	5	1.24E+08	1.24E+08	-
-0.45763	0.001009815	0.014207999	Fnip2	3	79455974	79567796	-
-0.45564	0.000165381	0.002977271	Dnmbp	19	43846821	43940191	-
-0.45495	0.000402336	0.006514695	Iqgap1	7	80711583	80825974	-
-0.45494	4.60E-07	1.40E-05	Por	5	1.36E+08	1.36E+08	+
-0.45478	1.21E-06	3.41E-05	Stap1	5	86071746	86106125	+
-0.45472	5.97E-07	1.76E-05	Mcoln3	3	1.46E+08	1.46E+08	+
-0.45397	6.75E-05	0.001353363	Plaur	7	24462484	24475968	+
-0.45322	0.000820687	0.012013535	Wfs1	5	36966104	36989205	-
-0.45197	0.001517674	0.019778895	Gsdme	6	50188888	50263862	-
-0.45084	3.07E-08	1.10E-06	Pmp22	11	63128982	63159547	+
-0.44925	7.15E-08	2.43E-06	Cdk18	1	1.32E+08	1.32E+08	-
-0.44796	3.34E-06	8.82E-05	B430306N(	17	48316141	48327024	+
-0.44743	0.000223903	0.003876699	Enpp1	10	24637914	24712159	-
-0.44705	0.000242926	0.004166255	Eya1	1	14168954	14310235	-
-0.44669	0.000110989	0.002088893	Rnf144b	13	47122656	47247991	+
-0.44599	1.08E-05	0.000257418	Cd9	6	1.25E+08	1.25E+08	-
-0.44586	0.000419579	0.006726263	Fyn	10	39368855	39565381	+
-0.44251	4.59E-07	1.40E-05	Mocos	18	24653691	24701556	+
-0.44091	2.76E-05	0.000601244	Hs6st1	1	36068400	36106446	+
-0.43852	0.002033859	0.025020953	Mgat5	1	1.27E+08	1.27E+08	+
-0.43777	0.003525458	0.039330978	Pdxk	10	78436744	78464975	-
-0.43757	0.000840516	0.012254288	Mbtps2	X	1.58E+08	1.58E+08	-
-0.43688	0.001745906	0.022058999	Kif13b	14	64647265	64809617	+
-0.43675	0.001433322	0.018986935	Gm14461	2	78237547	78302230	+
-0.43606	4.75E-07	1.44E-05	Rassf3	10	1.21E+08	1.21E+08	-

-0.43552	0.000126865	0.002339095	Pik3cb	9	99036654	99140621	-
-0.43508	0.000507475	0.007933477	Cdca7l	12	1.18E+08	1.18E+08	+
-0.43504	5.08E-07	1.53E-05	Capn2	1	1.82E+08	1.83E+08	-
-0.4346	1.99E-08	7.25E-07	Rab7b	1	1.32E+08	1.32E+08	+
-0.42998	3.58E-06	9.41E-05	Inf2	12	1.13E+08	1.13E+08	+
-0.42917	1.79E-05	0.000407687	Pqlc1	18	80253292	80292725	+
-0.42844	3.33E-05	0.000713261	Azin1	15	38487427	38519266	-
-0.42802	0.000773222	0.011410924	Cnrip1	11	17051586	17079371	+
-0.42791	0.001046283	0.014649983	Map4k3	17	80580512	80728093	-
-0.42784	0.000218681	0.00379991	Tln1	4	43531519	43562691	-
-0.42409	0.003292526	0.036931332	Mcu	10	59446984	59616692	-
-0.42364	5.32E-06	0.000134467	Lhfpl2	13	94057796	94195409	+
-0.4227	0.000256169	0.004372682	Tcp11l2	10	84576626	84614359	+
-0.42268	0.000424435	0.006781597	Atp6v1a	16	44085402	44139705	-
-0.42136	0.001431904	0.018985508	Synj2	17	5941280	6044290	+
-0.41993	1.30E-06	3.65E-05	Tecpr1	5	1.44E+08	1.44E+08	-
-0.41947	2.07E-06	5.61E-05	Neat1	19	5824708	5845478	-
-0.41922	0.001937753	0.024104644	Wrb	16	96145407	96157852	+
-0.41893	5.98E-07	1.76E-05	Slc26a11	11	1.19E+08	1.19E+08	+
-0.41892	6.14E-05	0.00124604	Lrrc27	7	1.39E+08	1.39E+08	+
-0.41399	0.000388328	0.006323752	Rgs18	1	1.45E+08	1.45E+08	-
-0.41394	7.23E-08	2.45E-06	Aplp2	9	31149557	31211815	-
-0.41271	0.003338918	0.037422738	Tsc1	2	28641228	28691167	+
-0.41194	1.06E-06	2.99E-05	Adgre5	8	83723251	83741326	-
-0.41093	0.004679968	0.049289316	Cenpa	5	30666777	30674830	+
-0.41073	0.000258342	0.00440458	Nck2	1	43444579	43570515	+
-0.41067	1.57E-05	0.000364202	Rin2	2	1.46E+08	1.46E+08	+
-0.4103	0.003753156	0.041456349	Ints6l	X	56454857	56507843	+
-0.41018	0.000648253	0.009715075	Cdt1	8	1.23E+08	1.23E+08	+
-0.4098	0.001692388	0.021551917	Eno2	6	1.25E+08	1.25E+08	-
-0.40909	0.000676173	0.010102167	Xdh	17	73883895	73950196	-
-0.40907	8.03E-06	0.000195541	Cebpa	7	35119293	35121928	+
-0.40722	2.06E-06	5.59E-05	Cipc	12	86947043	86965362	+
-0.4072	0.000484808	0.007636775	Trerf1	17	47140875	47361958	+
-0.40643	3.26E-05	0.000700289	Clic4	4	1.35E+08	1.35E+08	-
-0.40624	0.002460848	0.029064879	Dag1	9	1.08E+08	1.08E+08	-
-0.40539	0.000186458	0.00330337	Ubash3b	9	41011098	41161697	-
-0.40454	0.002090597	0.025481021	Adcy7	8	88272403	88329962	+
-0.40328	5.79E-05	0.001179141	Dennd4b	3	90265185	90280669	+
-0.40252	0.002475674	0.029216177	Npc1	18	12189693	12236400	-
-0.40251	0.000613833	0.009266321	Stra6l	4	45848664	45887008	+
-0.39958	0.000901914	0.013008885	Ppp4r1l-ps	2	1.74E+08	1.74E+08	-
-0.39941	0.000512168	0.007998207	Iqsec1	6	90656088	90988685	-
-0.39905	0.001820944	0.022847723	Gclc	9	77754535	77794485	+
-0.39789	0.000107773	0.00203631	Vwa5a	9	38718268	38743337	+
-0.39726	0.00021309	0.00371613	Rgs2	1	1.44E+08	1.44E+08	-
-0.39557	0.003533878	0.039394581	R3hdm1	1	1.28E+08	1.28E+08	+
-0.3945	0.001099075	0.01522734	Lpin1	12	16535669	16610966	-
-0.39361	0.00292398	0.033391899	Slc6a6	6	91684053	91759066	+
-0.38964	3.26E-05	0.000700289	Gpr137b-ps	13	12615057	12650388	-

-0.38904	0.00268743	0.031307265	Gcnt1	19	17326141	17356667	-
-0.38887	0.002905656	0.033235015	Ogfod1	8	94037198	94067921	+
-0.38215	3.40E-05	0.00072532	Arhgap18	10	26753421	26918648	+
-0.38014	0.004020616	0.043842564	Dopey2	16	93711904	93810590	+
-0.37996	0.004224109	0.045513594	Snx13	12	35047186	35147479	+
-0.37927	0.000170396	0.003056158	Myo7a	7	98051060	98119524	-
-0.37698	2.03E-05	0.000456403	C77080	4	1.29E+08	1.29E+08	-
-0.3758	1.71E-06	4.70E-05	Nptn	9	58582240	58657955	+
-0.3758	0.000924918	0.013231901	Itpkb	1	1.8E+08	1.8E+08	+
-0.37431	0.000351443	0.005794206	Cnr2	4	1.36E+08	1.36E+08	+
-0.37309	1.05E-05	0.00025	Jun	4	95049034	95052222	-
-0.37248	0.000697114	0.010393592	Flvcr1	1	1.91E+08	1.91E+08	-
-0.37238	0.00047431	0.007487685	Prkar2a	9	1.09E+08	1.09E+08	+
-0.37086	2.98E-05	0.000645891	Dusp7	9	1.06E+08	1.06E+08	+
-0.37055	0.001958604	0.024291163	Cyfip2	11	46193850	46312859	-
-0.37015	0.000913099	0.013127615	Ahcyl2	6	29768011	29912310	+
-0.36846	0.001282728	0.017288724	Cav2	6	17281185	17289115	+
-0.36735	0.001618547	0.020794308	Vwa8	14	78849052	79202310	+
-0.3671	0.000108633	0.002049886	Cadm1	9	47530173	47857637	+
-0.36544	1.22E-05	0.000287332	Plekhm1	11	1.03E+08	1.03E+08	-
-0.364	0.000325947	0.005423214	Mettl7a1	15	1E+08	1E+08	+
-0.36362	5.19E-05	0.001061863	Nceh1	3	27182965	27284608	+
-0.36315	0.000152312	0.002770372	Mindy1	3	95281345	95296166	+
-0.36258	1.81E-06	4.96E-05	Ipo5	14	1.21E+08	1.21E+08	+
-0.35959	0.002893178	0.033170832	Hist1h1c	13	23738808	23740367	+
-0.3578	0.000406954	0.006574779	Slc41a2	10	83230848	83337882	-
-0.35727	2.68E-05	0.000584448	Fblim1	4	1.42E+08	1.42E+08	-
-0.35656	0.000847804	0.012327095	Slc39a6	18	24579881	24603817	-
-0.35648	4.73E-05	0.000975378	Ggta1	2	35400179	35463231	-
-0.3559	0.004588511	0.048679866	Usp6nl	2	6322667	6446390	+
-0.35547	0.000617897	0.009308273	Edem1	6	1.09E+08	1.09E+08	+
-0.35423	0.001593401	0.020544101	Rab3gap2	1	1.85E+08	1.85E+08	+
-0.35423	7.66E-06	0.000187843	Zfand2a	5	1.39E+08	1.39E+08	-
-0.35349	0.001294007	0.017363657	Spryd7	14	61531993	61556886	-
-0.35344	9.67E-05	0.00186001	Tmem206	1	1.91E+08	1.91E+08	+
-0.35284	1.54E-06	4.28E-05	Clec7a	6	1.29E+08	1.29E+08	-
-0.35248	8.36E-05	0.001631119	Spp1	5	1.04E+08	1.04E+08	+
-0.35202	0.003288339	0.036912947	Msl3	X	1.69E+08	1.69E+08	-
-0.35124	0.000627747	0.009437036	Tpcn1	5	1.21E+08	1.21E+08	-
-0.35049	0.003417636	0.038186878	Kpna1	16	35978750	36037131	+
-0.35026	0.000111337	0.002092732	Socs6	18	88665224	88927481	-
-0.34962	0.000941029	0.013449103	Snx24	18	53245662	53390823	+
-0.34811	7.47E-05	0.001478761	Igf1	10	87858265	87937042	+
-0.34787	4.18E-05	0.000876805	Ttyh2	11	1.15E+08	1.15E+08	+
-0.34735	0.000127483	0.002347503	Arap3	18	37972624	37997574	-
-0.34719	0.002929329	0.033426647	Slc25a25	2	32414487	32451445	-
-0.34625	0.00149246	0.019485348	Adssl1	12	1.13E+08	1.13E+08	+
-0.3461	1.74E-05	0.000397149	C3ar1	6	1.23E+08	1.23E+08	-
-0.34609	0.000114451	0.002137405	Dgkd	1	87853287	87945180	+
-0.34571	0.003590628	0.039996453	Slain2	5	72914304	72978829	+

-0.34361	0.001783355	0.022464257	Tmem87b		2	1.29E+08	1.29E+08	+
-0.34341	0.00019797	0.00348177	Tubb2a		13	34074274	34078007	-
0.697281	3.06E-08	1.10E-06	-		7	1.07E+08	1.07E+08	+
-0.34321	7.75E-05	0.001526427	Degs1		1	1.82E+08	1.82E+08	-
-0.34211	2.00E-06	5.45E-05	Selenop		15	3268547	3280508	+
-0.33867	0.000947919	0.013507607	Lrp12		15	39870589	39943994	-
-0.33798	0.000388362	0.006323752	Zc3h12d		10	7832470	7870396	+
-0.33756	7.01E-05	0.00139705	Rap2a		14	1.2E+08	1.21E+08	+
-0.33644	0.002863574	0.032925442	Abcb1b		5	8798147	8866315	+
-0.33632	2.26E-06	6.08E-05	Gpnmb		6	49036546	49070929	+
-0.33419	0.000998091	0.01407037	Kansl3		1	36335730	36369181	-
-0.33288	0.0008968	0.01297048	Hmox1		8	75093621	75100589	+
-0.3323	2.42E-05	0.000532963	Mfsd12		10	81357491	81366225	+
-0.3316	0.001848914	0.023118604	Dhrs3		4	1.45E+08	1.45E+08	+
-0.3304	0.001188088	0.016227867	Ttc7b		12	1E+08	1.01E+08	-
-0.32808	0.004396997	0.047096293	Ptpre		7	1.36E+08	1.36E+08	+
-0.32771	0.00120582	0.016439079	Syngr1		15	80091334	80119501	+
-0.32667	4.89E-05	0.001004678	Man1c1		4	1.35E+08	1.35E+08	-
-0.32577	0.001045928	0.014649983	Pdk1		2	71873224	71903858	+
-0.32486	0.001035737	0.014530397	mt-Cytb	MT		14145	15288	+
-0.32451	0.004204182	0.045349672	Nhlrc3		3	53448583	53463332	-
-0.32424	2.33E-05	0.000517171	Lipa		19	34492318	34527474	-
-0.32352	0.000101705	0.001934259	Galnt6		15	1.01E+08	1.01E+08	-
-0.32159	0.001229317	0.016743667	Eaf1		14	31494399	31509858	+
-0.32065	0.000410038	0.006615508	Fmnl3		15	99317225	99370482	-
-0.32031	0.003979121	0.043455482	Taok3		5	1.17E+08	1.17E+08	+
-0.31868	0.00222162	0.026785118	Acbd5		2	23068167	23115558	+
-0.31699	0.002448451	0.028965673	Epb41		4	1.32E+08	1.32E+08	-
-0.31637	0.000947818	0.013507607	Amd1		10	40287458	40302186	-
-0.31579	0.004534262	0.048217204	Tsc22d1		14	76414961	76507765	+
-0.31558	0.000382886	0.006255671	Tatdn2		6	1.14E+08	1.14E+08	+
-0.31355	0.000170257	0.003056158	Apbb1ip		2	22774094	22875653	+
-0.31342	0.000426444	0.006798704	Rusc2		4	43381979	43427088	+
-0.31185	0.002638738	0.030814341	Camk2d		3	1.27E+08	1.27E+08	+
1.106192	9.87E-08	3.30E-06	2210406H1		7	1.21E+08	1.21E+08	-
-0.30955	0.003908216	0.042874996	Firre	X		50555744	50635321	-
-0.30873	0.003098379	0.035024735	Tmem175		5	1.09E+08	1.09E+08	+
-0.30803	0.000586756	0.008941402	Arhgef7		8	11727721	11835219	+
-0.30663	9.48E-05	0.001831388	Cd84		1	1.72E+08	1.72E+08	+
-0.30378	0.004049591	0.044059061	Epb41l1		2	1.56E+08	1.57E+08	+
-0.30319	0.001328666	0.017762937	Lat2		5	1.35E+08	1.35E+08	-
-0.30066	0.000411878	0.006632154	Speg		1	75375297	75432320	+
-0.30061	0.002295904	0.027561707	Pitrm1		13	6548149	6580515	+
-0.30052	0.001388659	0.018496731	Nt5c2		19	46886831	47015153	-
-0.30051	0.002602666	0.030466754	Bcl6		16	23965052	23988852	-
-0.29859	0.000605314	0.00916637	Trim35		14	66297031	66311424	+
-0.29836	0.002707545	0.031455563	Pacs2		12	1.13E+08	1.13E+08	+
-0.29825	0.000179534	0.003192744	C5ar1		7	16246743	16259540	-
-0.29577	0.00245894	0.029064879	Nek6		2	38511643	38594606	+
-0.29528	0.002791061	0.032178245	Rptor		11	1.2E+08	1.2E+08	+

-0.2944	0.00027992	0.004727969	Tpcn2		7	1.45E+08	1.45E+08	-
-0.29362	0.002319415	0.027756363	Aatk		11	1.2E+08	1.2E+08	-
-0.2936	0.001485346	0.019427471	Fam111a		19	12545740	12589768	+
-0.29168	0.002072778	0.025327745	mt-Nd4	MT		10167	11544	+
-0.5172	2.38E-07	7.58E-06	6430548M		8	1.2E+08	1.2E+08	+
-0.29134	0.002756361	0.031905102	Tnpo2		8	85036915	85057583	+
-0.291	0.003226665	0.036304989	Cd5l		3	87357881	87371073	+
-0.29076	0.004068708	0.044211602	Slc17a5		9	78536488	78588041	-
-0.28965	0.000857326	0.012424369	Prkch		12	73584796	73778185	+
-0.28922	8.33E-05	0.00162764	Vsir		10	60346851	60372684	+
-0.28879	0.000801176	0.011763566	Senp2		16	22009484	22049269	+
-0.28868	0.000219306	0.003801662	Tpd52		3	8925593	9004723	-
-0.28756	0.002193284	0.026553941	Nckap1l		15	1.03E+08	1.03E+08	+
-0.28406	0.00166636	0.02127655	Cd180		13	1.03E+08	1.03E+08	+
-0.28188	0.000990985	0.013997417	Tmem65		15	58782269	58823638	-
-0.28128	0.002568183	0.030111733	Pfkp		13	6579768	6648777	-
-0.28028	0.002487608	0.029309279	Aph1c		9	66814994	66834726	-
-0.27883	0.000586039	0.008939869	Inppl1		7	1.02E+08	1.02E+08	-
-0.27761	0.003824161	0.042080776	Actn1		12	80167547	80260371	-
-0.2774	0.002490066	0.029314409	Arhgap19		19	41766588	41802084	-
-0.27721	0.001410817	0.018740196	Gas6		8	13465374	13494490	-
-0.27575	0.000585454	0.008939869	S1pr2		9	20962361	20976781	-
-0.27571	0.004187448	0.045253165	Plin2		4	86648386	86670060	-
-0.2747	0.001455977	0.019166654	Srxn1		2	1.52E+08	1.52E+08	+
-0.27462	0.003796873	0.041843565	Gnpat		8	1.25E+08	1.25E+08	+
-0.2722	0.002708842	0.031455563	Tec		5	72755716	72868483	-
-0.27166	0.004635387	0.049141201	Mcm6		1	1.28E+08	1.28E+08	-
-0.27163	0.004069713	0.044211602	Sh2d3c		2	32721055	32755512	+
-0.27162	0.001476471	0.019346314	Cab39		1	85793441	85851576	+
-0.2715	0.000282735	0.004764408	Cat		2	1.03E+08	1.03E+08	-
-0.26835	0.003736441	0.041334735	Actn4		7	28893248	28962340	-
-0.26803	0.003255605	0.036602191	Vipas39		12	87238868	87266256	-
-0.26706	0.004678672	0.049289316	Hadhb		5	30155248	30184593	+
-0.26461	0.001480725	0.019384518	Cerk		15	86139128	86186141	-
-0.25713	0.001287374	0.017308801	Scarb1		5	1.25E+08	1.25E+08	-
-0.25644	0.00157421	0.020350981	Ywhah		5	33018816	33027966	+
-0.25348	0.004523505	0.048166524	Abcc3		11	94343295	94392997	-
-0.25221	0.001571855	0.020338683	Atp6v1b2		8	69088646	69113711	+
-0.24661	0.001787071	0.0224811	Hexb		13	97176331	97198357	-
-0.24094	0.001778923	0.022437032	Rtn4		11	29692947	29744331	+
-0.23948	0.002782914	0.03213545	Soat1		1	1.56E+08	1.56E+08	-
-0.23761	0.002079976	0.02537291	Apobec1		6	1.23E+08	1.23E+08	-
-0.23262	0.001738239	0.022013646	Myo1f		17	33555719	33607764	+
-0.23126	0.002498124	0.029361569	Dgkz		2	91932824	91975864	-
-0.23004	0.004273854	0.045981213	BC005537		13	24801657	24816197	+
-0.22288	0.003209777	0.036143042	Timp2		11	1.18E+08	1.18E+08	-
0.223151	0.003172368	0.035805268	Nfe2l2		2	75675513	75704641	-
0.232459	0.003399665	0.038015393	M6pr		6	1.22E+08	1.22E+08	+
0.23274	0.002355314	0.028023978	Glul		1	1.54E+08	1.54E+08	+
0.245221	0.003940765	0.043166717	Nucb1		7	45490458	45510406	-

0.246416	0.001117516	0.015409175	Capza2		6	17636234	17666972	+
0.247	0.00465105	0.049189709	Arpc4		6	1.13E+08	1.13E+08	+
0.247222	0.004534919	0.048217204	Dnajb6		5	29735688	29818134	+
0.251975	0.00078182	0.011502674	Serinc3		2	1.64E+08	1.64E+08	-
0.258642	0.00173052	0.021941118	Hsp90b1		10	86690209	86705509	-
0.258805	0.000951996	0.013552386	Fam20c		5	1.39E+08	1.39E+08	+
0.262186	0.004684228	0.049298351	Tmem38b		4	53826045	53862019	+
0.263159	0.001598862	0.02059618	Otud5	X		7841364	7876626	+
0.26431	0.001280889	0.017283649	P2rx4		5	1.23E+08	1.23E+08	+
0.266704	0.002706576	0.031455563	Ptpro		6	1.37E+08	1.37E+08	+
0.267417	0.000364969	0.005996751	App		16	84949685	85173766	-
0.270945	0.000553172	0.008519203	Marcks		10	37133375	37138920	-
0.27368	0.002042499	0.025042208	Ptpn6		6	1.25E+08	1.25E+08	-
0.275071	0.000812562	0.011906616	Snd1		6	28475139	28935162	+
0.276718	0.003824205	0.042080776	Max		12	76937269	76962201	-
0.278688	0.004128349	0.044781458	Ptma		1	86526726	86530712	+
0.280806	0.000944923	0.013491453	Themis2		4	1.33E+08	1.33E+08	-
0.281138	0.004157485	0.045002732	Ctss		3	95526786	95556403	+
0.281398	0.001959616	0.024291163	Fads1		19	10182888	10196870	+
0.282826	0.004034141	0.043923943	Surf4		2	26920040	26933928	-
0.284594	0.003675456	0.040847169	Pcbp1		6	86524492	86526321	-
0.284657	0.001691583	0.021551917	Zfp36		7	28376784	28380253	-
0.285646	0.000906163	0.013040836	Neurl3		1	36264597	36274679	-
0.286954	0.003704585	0.04110785	Vegfa		17	46016993	46032369	-
0.288427	0.001914739	0.023859325	Dnajb11		16	22857845	22879634	+
0.288886	0.004659038	0.049189709	Tma7		9	1.09E+08	1.09E+08	-
0.29081	0.002162527	0.026225391	Camkk2		5	1.23E+08	1.23E+08	-
0.293016	0.002009862	0.024788864	Fam234a		17	26211822	26244223	-
0.296711	0.000905791	0.013040836	Unc93b1		19	3935186	3949340	+
0.296715	0.000997055	0.014069447	Sdc3		4	1.31E+08	1.31E+08	+
0.296895	3.87E-05	0.000818547	Csf1r		18	61105572	61132149	+
0.298011	0.003932892	0.043113061	Gm6793		8	1.12E+08	1.12E+08	-
0.299787	0.001625199	0.020842818	Ppp1r21		17	88530118	88588367	+
0.304082	0.001324814	0.017727791	Hspa8		9	40800984	40810087	+
0.30775	0.000740228	0.010991169	Itgam		7	1.28E+08	1.28E+08	+
0.307894	0.000423124	0.00676811	Rnf149		1	39551296	39577405	-
0.308753	0.001491045	0.019484419	Arpc1b		5	1.45E+08	1.45E+08	+
0.314347	0.001464425	0.019211483	Vmp1		11	86583865	86683836	-
0.318552	0.000748626	0.011104489	Rnpep		1	1.35E+08	1.35E+08	-
0.319254	4.37E-05	0.000909642	Serp1		3	58519817	58525892	-
0.31976	0.000756393	0.011208234	Trim3		7	1.06E+08	1.06E+08	-
0.320625	0.001789276	0.022489321	Arhgap4	X		73891442	73921870	-
0.322474	0.001245049	0.016911401	Slc25a5	X		36795651	36798807	+
0.324871	0.002063171	0.025231627	Slc35c1		2	92452764	92460538	-
0.324883	0.002505599	0.029425554	Cstb		10	78425669	78427622	+
0.325063	0.001448644	0.019119482	H2-D1		17	35262730	35267499	+
0.325493	0.003775222	0.041636615	Naaa		5	92257659	92278170	-
0.32556	0.004450935	0.047638808	Rexo2		9	48468512	48480623	-
0.325872	0.000591137	0.008998699	Shisa5		9	1.09E+08	1.09E+08	+
0.327699	0.002106635	0.025654918	Plcl2		17	50509403	50688484	+

0.329605	1.42E-05	0.000330035	Adgre1		17	57358691	57483527	+
0.330061	0.002122514	0.025783293	Arrb2		11	70432635	70440828	+
0.332801	0.000727095	0.010807247	Slc12a9		5	1.37E+08	1.37E+08	-
0.333792	0.00030064	0.005036842	Zfp385a		15	1.03E+08	1.03E+08	-
0.335175	0.000651599	0.009755141	Triobp		15	78947724	79005869	+
0.338034	0.001961695	0.024291163	Zfp263		16	3744093	3750790	+
0.339018	0.000303415	0.005071619	Sdc4		2	1.64E+08	1.64E+08	-
0.339138	0.000114201	0.002135491	Csf3r		4	1.26E+08	1.26E+08	+
0.339316	0.000594372	0.009038451	Rnf31		14	55591708	55603693	+
0.340508	0.000163929	0.002962176	Nfkb1		3	1.36E+08	1.36E+08	-
0.34098	0.0038443	0.0422698	Tmem134		19	4125934	4132307	+
0.341085	0.000315972	0.005263297	Aars		8	1.11E+08	1.11E+08	+
0.341544	0.004580292	0.04862827	Fcer1g		1	1.71E+08	1.71E+08	-
0.342026	0.002781343	0.03213545	Spi1		2	91082390	91115756	+
0.34219	0.002116842	0.025735971	Trim12c		7	1.04E+08	1.04E+08	-
0.344472	5.97E-05	0.001214699	Hspa5		2	34771970	34777547	+
0.344725	0.002995517	0.034047866	Cox7c		13	86044816	86046904	-
0.344784	0.001464856	0.019211483	Vdac3		8	22577075	22593813	-
0.346303	0.000837253	0.012218999	Def8		8	1.23E+08	1.23E+08	+
0.348633	0.001623338	0.020837395	AB124611		9	21526176	21545333	+
0.35052	0.000541426	0.008382855	Ube2f		1	91250304	91290337	+
0.350601	0.004033098	0.043923943	Ubac2		14	1.22E+08	1.22E+08	+
0.350606	0.002400036	0.028439345	Zfp467		6	48427697	48445825	-
0.352799	0.000128412	0.00236161	Stat3		11	1.01E+08	1.01E+08	-
0.353625	0.00163233	0.020897281	Psma6		12	55384222	55418454	+
0.353633	0.000210323	0.003676718	Axl		7	25757273	25788705	-
0.356206	0.001363587	0.018196227	Cars		7	1.44E+08	1.44E+08	-
0.356795	0.002004811	0.024747636	C1qc		4	1.37E+08	1.37E+08	-
0.357814	0.000236343	0.004062972	Tifa		3	1.28E+08	1.28E+08	+
0.357875	7.70E-06	0.000188593	Acp2		2	91202885	91214098	+
0.360181	0.001571336	0.020338683	Rhog		7	1.02E+08	1.02E+08	-
0.362203	0.003177391	0.035834048	Psmb5		14	54614119	54618022	-
0.36299	0.000105604	0.001997928	Cept1		3	1.07E+08	1.07E+08	-
0.363242	0.000635269	0.009540222	Abca7		10	79996494	80015572	+
0.365875	4.67E-05	0.00096606	Tbc1d13		2	30133746	30152013	+
0.365961	0.001062967	0.014826293	Parp11		6	1.27E+08	1.27E+08	+
0.366337	0.002674795	0.031185141	Slc25a22		7	1.41E+08	1.41E+08	-
0.366419	0.001447953	0.019119482	Calm3		7	16915379	16924114	-
0.367976	0.003281492	0.036864637	Cyp4f16		17	32536558	32551798	+
0.368165	0.002891817	0.033170832	Sdhb		4	1.41E+08	1.41E+08	+
0.370281	0.004657767	0.049189709	Rpl36a	X		1.35E+08	1.35E+08	+
0.371905	0.000831505	0.012157837	Clec4d		6	1.23E+08	1.23E+08	+
0.372078	0.004020044	0.043842564	Park7		4	1.51E+08	1.51E+08	-
0.372318	0.004205766	0.045349672	Idnk		13	58157649	58164693	+
0.372653	0.002059779	0.02521142	Ssr2		3	88575876	88588419	+
0.373123	1.40E-05	0.000325035	Rasa4		5	1.36E+08	1.36E+08	+
0.373483	0.000152359	0.002770372	Ddx24		12	1.03E+08	1.03E+08	-
0.37357	4.53E-05	0.000939433	Kmt5a		5	1.24E+08	1.24E+08	+
0.374405	0.001163984	0.015928671	Glrx		13	75839868	75850154	+
0.37453	0.001611348	0.020738594	Tank		2	61578585	61654171	+

0.375242	0.000196165	0.003456006	Rac2	15	78559167	78572783	-
0.376988	0.001640174	0.020979172	Osgep	14	50906478	50924893	-
0.378037	0.002291646	0.027560603	Bax	7	45461697	45466898	-
0.378846	0.000104257	0.001975015	Calr	8	84841850	84846934	-
0.38049	4.37E-05	0.000909642	Tnip1	11	54910785	54962917	-
0.3812	0.004240402	0.045655208	Hmga1b	11	1.21E+08	1.21E+08	+
0.381294	0.000461103	0.007287142	Ggh	4	20042052	20066750	+
0.381318	2.15E-06	5.79E-05	Il10ra	9	45253837	45269149	-
0.382865	9.46E-07	2.71E-05	Mapk14	17	28691329	28748406	+
0.382879	0.001806104	0.022681155	Tor1a	2	30960627	30967933	-
0.383189	7.37E-05	0.001462937	Il4ra	7	1.26E+08	1.26E+08	+
0.383335	0.000382736	0.006255671	Rbpj	5	53466152	53657362	+
0.384525	0.003085637	0.034929339	AI837181	19	5425157	5427313	+
0.385696	0.00237322	0.028213868	Hspe1	1	55088132	55091307	+
0.385741	0.000968592	0.013761603	Pdia6	12	17266545	17284770	+
0.386254	0.000347541	0.005736407	Myd88	9	1.19E+08	1.19E+08	-
0.388555	0.003702102	0.04110785	Pkig	2	1.64E+08	1.64E+08	+
0.389015	0.000575065	0.008818886	Mtmr14	6	1.13E+08	1.13E+08	+
0.389109	0.001918074	0.023880348	Cers6	2	68861441	69114282	+
0.389625	0.000343634	0.00567839	Serpinb8	1	1.08E+08	1.08E+08	+
0.39026	4.16E-05	0.000873161	Emilin1	5	30913402	30921277	+
0.391345	0.000677185	0.010106866	Nod1	6	54923949	54972612	-
0.392516	0.002856465	0.032879976	Edf1	2	25557847	25562082	+
0.392788	0.00055722	0.00857243	Chmp4b	2	1.55E+08	1.55E+08	+
0.393933	0.004518031	0.048143608	Atp5l	9	44912557	44920742	-
0.394138	0.002319282	0.027756363	Slc16a3	11	1.21E+08	1.21E+08	+
0.396029	1.13E-06	3.20E-05	Fos	12	85473890	85477273	+
0.396053	0.001339031	0.017885011	Isoc1	18	58659482	58679570	+
0.518016	1.60E-05	0.00036823	1600014C1	7	38183217	38197568	+
0.396606	0.002042378	0.025042208	Tent5a	9	85320439	85327348	-
0.397204	0.002299935	0.027568781	Ptgs1	2	36230426	36252272	+
0.398336	1.52E-06	4.21E-05	Snx10	6	51523901	51590679	+
0.398614	0.001068962	0.014895581	Cd300lf	11	1.15E+08	1.15E+08	-
0.3989	0.003949703	0.043221891	Chrac1	15	73090392	73097554	+
0.400206	0.002936642	0.033431123	Rpl35	2	39001580	39005624	-
0.401956	0.002279429	0.027436455	Cox8a	19	7215158	7217616	-
0.402019	0.000225103	0.003888187	Stxbp1	2	32787602	32847245	-
0.403723	0.000384154	0.006269323	St3gal5	6	72097592	72154571	+
0.404391	0.001126307	0.015514328	Iscu	5	1.14E+08	1.14E+08	+
0.405337	0.000777253	0.011458742	Atp5g3	2	73908447	73911326	-
0.405738	0.003887771	0.042683011	Acbd6	1	1.56E+08	1.56E+08	+
0.405916	7.09E-05	0.001410505	Pim1	17	29490812	29496111	+
0.406015	0.001744262	0.022057461	Mpv17l2	8	70758649	70760946	-
0.406629	0.000702298	0.010460129	Nuak1	10	84370905	84440597	-
0.408833	6.51E-06	0.000161305	Rab32	10	10545002	10558265	-
0.409004	0.001272056	0.01718046	Uqcr11	10	80402997	80406830	-
0.409417	0.003015253	0.034218512	Procr	2	1.56E+08	1.56E+08	+
0.410592	0.002297443	0.027561707	Uqcr10	11	4701973	4704342	-
0.411262	0.000848222	0.012327095	Ndufa3	7	3617373	3620327	+
0.411502	0.002328922	0.027824192	Manf	9	1.07E+08	1.07E+08	-

0.412633	0.000656601	0.009819877	Pfn1		11	70651850	70654644	-
0.413227	0.000337308	0.005586586	Usp35		7	97309380	97332020	-
0.416881	0.000780179	0.011490199	C1qb		4	1.37E+08	1.37E+08	-
0.417776	0.001092048	0.015158963	St3gal1		15	67102875	67176830	-
0.417941	0.000516698	0.00806027	Prdx2		8	84969587	84974834	+
0.418518	9.68E-05	0.00186001	Vars		17	35000987	35016322	+
0.419573	1.73E-05	0.000396355	Tlr1		5	64924679	64933563	-
0.421869	0.00449168	0.048003999	Ethe1		7	24587543	24608925	+
0.422243	7.28E-09	2.80E-07	Tnfaip2		12	1.11E+08	1.11E+08	+
0.423093	0.002113978	0.025722729	Cdk2ap2		19	4097182	4099019	+
0.423797	0.000164433	0.002967574	Trim21		7	1.03E+08	1.03E+08	-
0.425633	3.12E-07	9.67E-06	Nampt		12	32819545	32853349	+
0.42611	4.75E-06	0.000120769	Fgd2		17	29360914	29379660	+
0.426542	0.000232969	0.004014488	Ly86		13	37345208	37419036	+
0.427032	0.001302746	0.017448603	Cops9		1	92637145	92641985	-
0.429824	0.001095515	0.015192545	Dok3		13	55523231	55529296	-
0.432037	2.30E-05	0.00051223	Pnrc1		4	33245423	33290163	-
0.435094	0.002217322	0.026755558	Lsm4		8	70673248	70678752	+
0.438013	0.001825281	0.022882332	Cox7b	X		1.06E+08	1.06E+08	+
0.438799	0.004286204	0.046079878	Il13ra1	X		36112110	36171259	+
0.438849	0.001580787	0.020417792	Gm6472	X		1.53E+08	1.53E+08	-
0.439914	0.001739279	0.022013646	Lamtor2		3	88549819	88553074	-
0.439933	0.000371089	0.0060904	Psma7		2	1.8E+08	1.8E+08	-
0.441455	7.67E-05	0.001513206	Scarf1		11	75513540	75526582	+
0.442513	0.000456884	0.007244154	Dbnl		11	5788488	5800962	+
0.442749	0.000410388	0.006615508	Nectin4		1	1.71E+08	1.71E+08	+
0.443228	0.002866701	0.032925442	Ermard		17	15041208	15090044	+
0.444373	0.003723443	0.041253925	Rpl35a		16	33056453	33060189	+
0.445383	0.000414022	0.006659276	Nfkbib		7	28758251	28767512	-
0.445533	0.000122167	0.002266901	Mvp		7	1.27E+08	1.27E+08	-
0.447904	0.001532992	0.019960579	Ddhd1		14	45588467	45658143	-
0.449604	0.00473732	0.049748724	Anapc13		9	1.03E+08	1.03E+08	+
0.449699	8.18E-05	0.00160242	Tent5c		3	1E+08	1E+08	-
0.449829	7.42E-05	0.001471764	Ctsz		2	1.74E+08	1.74E+08	-
0.450179	0.00027082	0.00459032	Flot1		17	35823230	35832791	+
0.452472	6.51E-05	0.001310681	Ifitm2		7	1.41E+08	1.41E+08	-
0.45453	0.00195581	0.02428758	Uqcrb		13	66900617	66905378	-
0.454612	0.000846122	0.012323623	Hagh		17	24840143	24864450	+
0.45482	8.80E-06	0.000212261	Pak1		7	97788541	97912381	+
1.753492	4.06E-05	0.000853929	-		13	1.2E+08	1.2E+08	+
0.457207	0.001392957	0.018536945	Sem1		6	6557294	6578663	-
0.457616	9.58E-05	0.001847979	Ndufa1	X		37187588	37191163	-
0.457961	0.004509572	0.048088831	Rps19-ps6		12	1.11E+08	1.11E+08	+
0.460395	0.000585803	0.008939869	Nfkbiz		16	55811375	55838899	-
0.460503	0.002721414	0.031576245	Tmem110		14	30825590	30877210	+
0.46085	9.94E-07	2.83E-05	Agtrap		4	1.48E+08	1.48E+08	-
0.461421	4.12E-08	1.44E-06	Pid1		1	84036296	84364180	-
0.461925	9.32E-05	0.001804815	Txn1		4	57943373	57956411	-
0.462268	0.001545806	0.020091316	Fkbp2		19	6977741	6980461	-
0.467129	0.000458217	0.007257361	Rpl7a		2	26910764	26913318	+

0.467669	3.13E-05	0.000675951	Stk40	4	1.26E+08	1.26E+08	+
0.46851	0.001189532	0.016232297	Uqcrq	11	53427922	53430831	-
0.46973	0.001435936	0.019004191	Tmem160	7	16452779	16455494	+
0.469948	1.99E-07	6.44E-06	Adam17	12	21323509	21373632	-
0.470132	1.81E-05	0.000409483	Kctd12	14	1.03E+08	1.03E+08	-
0.470847	0.000189602	0.003350868	Slc25a37	14	69241848	69285112	-
0.475377	0.000266346	0.004530382	Rpl38	11	1.15E+08	1.15E+08	+
0.475444	4.74E-07	1.44E-05	Mcoln2	3	1.46E+08	1.46E+08	+
0.475618	0.001056633	0.01478062	Ebi3	17	55952640	55957022	+
0.476133	0.000429277	0.006828848	Rpl14-ps1	7	45324965	45325617	+
0.476624	0.001129189	0.015525817	Bcat2	7	45570153	45589711	+
0.478516	2.40E-05	0.000529287	Ninj1	13	49187485	49196244	+
0.479478	0.001110582	0.015357399	Gsta3	1	21240589	21265661	+
0.480788	0.004292609	0.046114517	Klf2	8	72319033	72321656	+
0.482581	1.43E-06	3.98E-05	Tnip3	6	65525313	65634040	+
0.483492	2.73E-06	7.28E-05	Fbrsl1	5	1.1E+08	1.1E+08	-
0.48361	1.70E-07	5.54E-06	Lcp2	11	34046920	34092295	+
0.484687	0.001456332	0.019166654	Ndufs6	13	73319838	73328542	-
5.394891	6.42E-05	0.001296353	-	5	64840411	64865449	-
0.487308	0.002572289	0.030135501	Gm4366	7	1.17E+08	1.17E+08	-
0.487909	0.001297243	0.017390978	Rpl9-ps6	19	32465885	32466575	-
0.488623	0.000332539	0.005520222	Gpd2	2	57237635	57370719	+
0.489051	0.000246333	0.004219687	Sec61b	4	47474658	47483242	+
0.489388	2.59E-09	1.06E-07	Csf2rb	15	78325752	78353847	+
0.489694	0.000194559	0.003434283	Sqor	2	1.23E+08	1.23E+08	+
0.491209	4.31E-06	0.000111016	Adar	3	89715022	89753446	+
0.491997	7.33E-06	0.000180304	Mthfr	4	1.48E+08	1.48E+08	+
0.492833	4.85E-05	0.00099717	Cd274	19	29367455	29388095	+
0.494303	0.001246555	0.016914863	Gm6563	19	23675844	23676712	+
0.494758	0.000237712	0.004081656	Rnase4	14	51091077	51106151	+
0.49615	0.000503558	0.007902751	Ptgir	7	16906490	16910905	+
0.497676	0.002206607	0.026670687	B9d2	7	25680780	25686558	+
0.497734	4.08E-06	0.000105675	Hexim1	11	1.03E+08	1.03E+08	+
0.497932	4.18E-07	1.29E-05	Emb	13	1.17E+08	1.17E+08	+
0.498792	7.26E-05	0.001443328	Tspo	15	83563592	83574203	+
0.498986	7.00E-06	0.000172746	Ets2	16	95702075	95721051	+
0.499218	4.33E-05	0.00090327	Parp3	9	1.06E+08	1.06E+08	-
0.501441	0.001074101	0.014952806	Hpgd	8	56294585	56321043	+
0.503059	6.81E-05	0.001362139	Snx20	8	88626563	88636128	-
0.504041	0.000599114	0.00909148	Atp5mpl	12	1.12E+08	1.12E+08	-
0.504878	8.33E-05	0.00162764	Sephs2	7	1.27E+08	1.27E+08	-
0.505273	4.64E-06	0.000118489	Tifab	13	56173704	56178885	-
0.505854	0.000141598	0.002590959	Nfxl1	5	72513301	72559684	-
0.507282	0.000248	0.00423822	Traf3ip3	1	1.93E+08	1.93E+08	-
0.508582	0.001449925	0.019119482	Commd1	11	22896136	22982382	-
0.50878	0.001373099	0.0183063	Mcub	3	1.3E+08	1.3E+08	-
0.510652	0.001568956	0.020337489	Sec61g	11	16500530	16508484	-
0.511873	3.98E-06	0.000103868	Hlx	1	1.85E+08	1.85E+08	-
0.51278	0.00198019	0.024485426	Rpl7a-ps5	17	57838430	57839221	-
0.51288	6.95E-06	0.000171773	Pgap2	7	1.02E+08	1.02E+08	+

0.514183	1.19E-09	5.07E-08	Zmiz2	11	6389074	6406158	+
0.515003	0.000308669	0.0051535	Atp5e	2	1.74E+08	1.74E+08	-
0.516282	3.77E-05	0.000798109	Btg2	1	1.34E+08	1.34E+08	-
0.51632	6.80E-05	0.001360606	Cyba	8	1.22E+08	1.22E+08	-
0.51848	1.50E-05	0.00034775	Mov10	3	1.05E+08	1.05E+08	-
0.519009	0.000892447	0.012920416	Gm26917	17	39843013	39846341	+
0.519888	0.000120884	0.002247618	Bmf	2	1.19E+08	1.19E+08	-
0.521158	0.000101961	0.001936599	Vamp8	6	72385223	72390703	-
0.524211	0.000404646	0.006544782	Krtcap2	3	89245966	89249906	+
0.526519	0.000427897	0.00681438	Rps28	17	33819027	33824562	-
0.526752	1.83E-06	4.99E-05	Arhgdib	6	1.37E+08	1.37E+08	-
0.874611	0.000101361	0.001930253	-	19	4061385	4066463	+
0.52709	4.07E-06	0.000105675	Tap2	17	34203527	34216321	+
0.52736	0.001962804	0.024291163	Creb5	6	53287270	53700376	+
0.529261	0.000453353	0.007196049	Tpst1	5	1.3E+08	1.3E+08	+
0.531247	2.49E-05	0.000545699	Sp140	1	85600378	85645037	+
0.535711	0.000112234	0.002106868	Sfxn5	6	85213049	85333422	-
0.539282	1.65E-05	0.000377986	Msrb1	17	24736642	24742778	+
0.542864	0.000219259	0.003801662	Sh3bp1	15	78899768	78912051	+
0.544023	1.59E-05	0.00036823	Sap30	8	57482707	57487860	-
0.544265	0.000177139	0.003161449	Abracl	10	18011260	18023288	-
0.54428	5.05E-11	2.48E-09	Sgk1	10	21882184	21999903	+
0.544319	0.002170499	0.026300063	Mitd1	1	37874801	37890411	-
0.545422	1.14E-05	0.000270593	H2-DMb1	17	34153072	34160230	+
0.547245	0.001784182	0.022464257	Ptp4a3	15	73723145	73758766	+
0.547796	1.75E-06	4.79E-05	Cfp	X	20925454	20931555	-
0.55011	8.56E-09	3.27E-07	Rnf114	2	1.67E+08	1.68E+08	+
0.552424	2.15E-07	6.92E-06	Cox5b	1	36691449	36693681	+
0.552606	4.34E-08	1.51E-06	Ccdc88b	19	6844623	6858211	-
0.55772	2.08E-06	5.61E-05	Ncf4	15	78244801	78262580	+
0.558515	1.03E-09	4.42E-08	Pld4	12	1.13E+08	1.13E+08	+
0.559597	1.07E-05	0.000255235	Tmem173	18	35733678	35740554	-
0.560992	1.71E-05	0.000392017	Cox5a	9	57521274	57532426	+
0.561306	1.04E-07	3.46E-06	Junb	8	84974484	84978718	-
0.564271	1.92E-11	1.00E-09	Ncf1	5	1.34E+08	1.34E+08	-
0.566892	4.03E-06	0.00010492	Atox1	11	55446641	55461239	-
0.567394	4.55E-05	0.00094204	Rps21	2	1.8E+08	1.8E+08	+
0.570049	4.07E-06	0.000105675	Gch1	14	47153895	47189413	-
0.570155	5.62E-06	0.00014132	Pfkl	10	77986947	78010083	-
0.570278	1.94E-08	7.15E-07	Hif1a	12	73901375	73947530	+
0.571175	1.45E-08	5.42E-07	Ccl9	11	83572919	83578636	-
0.573305	2.08E-09	8.67E-08	Mocs1	17	49428362	49455435	+
0.578771	4.77E-09	1.89E-07	Daxx	17	33909414	33915589	+
0.580009	1.93E-05	0.000436276	Ndufa13	8	69894180	69902558	-
0.581116	8.31E-07	2.41E-05	Dpysl2	14	66802864	66868688	-
0.584463	1.24E-05	0.000290583	Ak2	4	1.29E+08	1.29E+08	+
0.585646	2.43E-05	0.000533962	Nt5c3	6	56882400	56923932	-
0.586097	5.97E-06	0.000149716	Gngt2	11	95837216	95845734	+
0.586101	5.64E-10	2.50E-08	Ms4a6d	19	11586604	11604849	-
0.588463	2.88E-08	1.04E-06	Mllt6	11	97663414	97685463	+

0.590887	0.000162472	0.002943178	Ppp1r12b	1	1.35E+08	1.35E+08	-
0.592073	1.05E-05	0.00025	Mvd	8	1.22E+08	1.22E+08	-
0.592124	3.82E-06	1.00E-04	Acaa1a	9	1.19E+08	1.19E+08	+
0.593432	0.001013425	0.014244967	Cry1	10	85131700	85185064	-
0.594136	2.97E-05	0.000644624	Lipe	7	25379527	25398710	-
0.59423	1.33E-11	7.05E-10	Rnf19b	4	1.29E+08	1.29E+08	+
0.594739	3.39E-08	1.21E-06	H2-M3	17	37270220	37274484	+
0.597532	7.54E-09	2.88E-07	Gm20559	6	3333194	3346128	-
0.597678	2.01E-12	1.13E-10	Slc31a1	4	62360727	62391769	+
0.599373	3.96E-05	0.000834846	Tsku	7	98350668	98361328	-
0.60145	4.96E-08	1.72E-06	Pml	9	58218076	58249786	-
0.603326	7.80E-06	0.000190341	Acot7	4	1.52E+08	1.52E+08	+
0.605944	3.40E-06	8.97E-05	Rassf4	6	1.17E+08	1.17E+08	-
0.609036	3.65E-07	1.12E-05	Hcls1	16	36934983	36963212	+
0.609984	1.23E-05	0.000288444	Olfm1	2	28192992	28230736	+
0.610656	2.74E-11	1.39E-09	B2m	2	1.22E+08	1.22E+08	+
0.614652	2.49E-10	1.14E-08	Pla2g7	17	43568098	43612201	+
0.616493	0.000528042	0.008201908	Fstl1	16	37776873	37836514	+
0.618112	3.39E-05	0.000724877	Samsn1	16	75858793	76022281	-
0.618991	0.001081024	0.015034736	Map4k1	7	28982050	29003279	+
0.62288	4.35E-12	2.39E-10	Trafd1	5	1.21E+08	1.21E+08	-
0.626143	1.80E-05	0.000408825	Lox	18	52516069	52529867	-
0.627898	2.78E-06	7.40E-05	Trim12a	7	1.04E+08	1.04E+08	-
0.628702	1.94E-08	7.14E-07	Gsdmd	15	75862327	75867408	+
0.63082	5.40E-08	1.86E-06	Parp10	15	76231174	76243441	-
0.631379	1.08E-10	5.18E-09	Clec4a3	6	1.23E+08	1.23E+08	+
0.632522	0.002867231	0.032925442	Snhg8	3	1.24E+08	1.24E+08	-
0.634298	0.000140587	0.00257571	Uck2	1	1.67E+08	1.67E+08	-
0.635302	0.00145747	0.019166654	CAAA0114	JH584295.1	66	1479	-
0.635424	3.38E-11	1.70E-09	Gpr65	12	98268635	98276644	+
0.639574	1.24E-07	4.07E-06	Rras	7	45017961	45021647	+
0.64089	6.59E-14	4.12E-12	CT010467.1	17	39846353	39848827	-
0.641071	4.47E-07	1.37E-05	Rarg	15	1.02E+08	1.02E+08	-
0.642641	9.44E-08	3.17E-06	Tent4a	13	69497959	69534617	-
0.643017	0.000163425	0.002956751	Stx11	10	12938209	12964298	-
0.643019	9.45E-08	3.17E-06	Coro1a	7	1.27E+08	1.27E+08	-
0.643799	9.87E-06	0.000236367	Samd9l	6	3372257	3399572	-
0.648105	0.000155268	0.002819726	Svbp	4	1.19E+08	1.19E+08	+
0.649728	7.05E-14	4.38E-12	Mapkapk2	1	1.31E+08	1.31E+08	-
0.652406	1.13E-05	0.000266786	Rps29	12	69157722	69159186	-
-0.389	0.000228805	0.00394743	2010111I0	13	62964893	63326096	+
0.654957	1.77E-18	1.46E-16	Nadk	4	1.56E+08	1.56E+08	+
0.65592	0.002075597	0.025340821	Dst	1	33908225	34308661	+
0.661389	0.002932696	0.033431123	Treml2	17	48299498	48312533	+
0.662748	2.98E-07	9.36E-06	P2ry14	3	59113855	59153618	-
0.663739	9.11E-06	0.000218649	Zfas1	2	1.67E+08	1.67E+08	+
0.66434	6.99E-19	5.96E-17	Samhd1	2	1.57E+08	1.57E+08	-
0.669615	2.44E-10	1.12E-08	mt-Rnr1	MT	70	1024	+
0.669819	2.53E-09	1.04E-07	Fbxw17	13	50417864	50433780	+
0.670208	5.13E-11	2.51E-09	Dram1	10	88322804	88379080	-

0.670519	1.09E-14	7.29E-13	Cd82		2	93419111	93463140	-
0.674025	9.96E-05	0.001908786	Atp5k		5	1.08E+08	1.08E+08	-
0.676415	0.003729011	0.041284057	Lrrc8a		2	30237715	30263790	+
0.676889	3.40E-10	1.54E-08	Lyz2		10	1.17E+08	1.17E+08	-
0.67736	1.66E-06	4.59E-05	Mrpl52		14	54426909	54429756	+
0.677701	5.12E-06	0.000129659	Napsa		7	44572380	44586862	+
0.678111	3.02E-09	1.23E-07	Gsap		5	21186255	21315132	+
0.678544	4.03E-09	1.62E-07	N4bp1		8	86808160	86885258	-
0.678584	6.06E-09	2.37E-07	Limd2		11	1.06E+08	1.06E+08	-
0.679277	7.91E-05	0.001551242	Tagap		17	7926000	7934897	+
0.679569	8.97E-12	4.80E-10	Uba7		9	1.08E+08	1.08E+08	+
0.68176	1.15E-05	0.000272321	Fam213b		4	1.55E+08	1.55E+08	-
0.683287	6.47E-06	0.000160616	Tfec		6	16833373	16898441	-
0.685376	7.14E-08	2.43E-06	Ogfr		2	1.81E+08	1.81E+08	+
0.687672	3.08E-08	1.10E-06	Parp8		13	1.17E+08	1.17E+08	-
0.688275	0.000130244	0.00239226	Siglec1		2	1.31E+08	1.31E+08	-
1.072949	0.000301798	0.005050406	4933412E1		10	1.17E+08	1.17E+08	+
0.691759	5.64E-06	0.000141541	Acp5		9	22126727	22135711	-
0.691937	1.25E-18	1.05E-16	Ikbke		1	1.31E+08	1.31E+08	-
0.693002	2.39E-06	6.41E-05	F10		8	13037308	13056676	+
0.694793	1.07E-15	7.56E-14	Tor3a		1	1.57E+08	1.57E+08	-
0.695274	2.33E-11	1.20E-09	Parvg		15	84324026	84342978	+
0.695485	3.32E-09	1.35E-07	Herpud1		8	94386438	94395377	+
0.69797	1.32E-09	5.60E-08	Src		2	1.57E+08	1.57E+08	+
0.698251	4.63E-09	1.84E-07	Parp14		16	35832874	35871544	-
0.698894	3.59E-05	0.000763826	Ms4a4a		19	11375523	11392790	+
0.701875	3.44E-11	1.72E-09	Gdpd1		11	87033867	87074062	-
0.702184	2.35E-06	6.31E-05	Il18bp		7	1.02E+08	1.02E+08	-
0.707567	4.45E-10	2.00E-08	Clec4a2		6	1.23E+08	1.23E+08	+
0.709007	0.002665483	0.031101599	Gm20658		12	1.16E+08	1.16E+08	-
0.709213	3.40E-08	1.21E-06	Ptms		6	1.25E+08	1.25E+08	-
0.71177	2.74E-07	8.66E-06	Gab1		8	80764438	80880519	-
0.714832	2.29E-09	9.49E-08	Pik3r6		11	68503019	68552698	+
0.717031	2.40E-08	8.73E-07	Agrn		4	1.56E+08	1.56E+08	-
0.719981	7.35E-09	2.82E-07	Psmd10	X		1.41E+08	1.41E+08	-
0.722992	2.73E-14	1.76E-12	Hck		2	1.53E+08	1.53E+08	+
0.725889	0.001136992	0.015603494	Pcx		19	4510472	4621752	+
0.726809	2.01E-11	1.05E-09	Znfx1		2	1.67E+08	1.67E+08	-
0.728721	0.000100302	0.001919529	Arid5a		1	36307733	36324029	+
0.730215	0.002741172	0.031754644	AC121151.		17	16557246	16558043	+
0.735819	3.60E-08	1.27E-06	Pex16		2	92374676	92381217	+
0.736121	0.003201833	0.036081626	Gm10154		4	83505185	83505538	-
0.736251	4.22E-05	0.000884338	Snta1		2	1.54E+08	1.54E+08	-
0.738288	0.002923578	0.033391899	Spef1		2	1.31E+08	1.31E+08	-
0.743325	3.09E-10	1.41E-08	Ccdc86		19	10941481	10949266	-
0.747787	3.43E-07	1.06E-05	Ndufb1-ps		12	1.02E+08	1.02E+08	-
0.749863	0.001562152	0.020267416	Relt		7	1.01E+08	1.01E+08	-
0.754869	1.89E-12	1.07E-10	Fcrls		3	87250758	87263738	-
0.756942	0.000178369	0.00317949	Cacnb1		11	98001508	98023034	-
0.759702	2.15E-05	0.000481629	Eif2ak2		17	78852564	78882573	-

0.760901	0.004502117	0.048080086	Gm10093		17	78491582	78493025	+
0.761313	4.23E-13	2.50E-11	Nxpe5		5	1.38E+08	1.38E+08	+
0.761671	4.92E-07	1.49E-05	Gmfg		7	28437447	28448233	+
0.769163	5.01E-10	2.23E-08	H2-T22		17	36037128	36042747	-
0.769449	1.72E-16	1.27E-14	Irf9		14	55603571	55610030	+
0.77287	2.06E-11	1.07E-09	Psme2b		11	48945354	48946190	-
0.776378	1.36E-19	1.21E-17	Slc31a2		4	62262562	62298411	+
0.778408	2.36E-11	1.21E-09	Cep85		4	1.34E+08	1.34E+08	-
0.779033	2.59E-11	1.33E-09	Cybb	X		9435252	9487771	-
0.780842	4.20E-20	3.93E-18	Igsf6		7	1.21E+08	1.21E+08	-
0.786176	9.19E-07	2.64E-05	Mlkl		8	1.11E+08	1.11E+08	-
0.786792	2.70E-15	1.88E-13	Parp12		6	39086410	39118349	-
0.78738	8.65E-06	0.000209029	Gda		19	21391307	21473445	-
0.789308	8.81E-13	5.08E-11	Irak3		10	1.2E+08	1.2E+08	-
0.791324	3.88E-16	2.78E-14	Btg1		10	96617006	96622809	+
0.794685	3.73E-06	9.77E-05	Acss2		2	1.56E+08	1.56E+08	+
0.79674	2.28E-10	1.05E-08	Tnf		17	35199381	35202007	-
0.799727	4.13E-08	1.44E-06	Selenow		7	15917208	15922402	-
0.800705	4.31E-06	0.000111054	Nectin2		7	19716644	19750483	-
0.806426	4.59E-09	1.83E-07	Lst1		17	35185095	35188439	-
0.807309	1.22E-06	3.44E-05	Cysltr1	X		1.07E+08	1.07E+08	-
0.808902	1.63E-08	6.06E-07	Cebpb		2	1.68E+08	1.68E+08	+
0.812905	5.57E-06	0.00014034	Sirpb1c		3	15795145	15848528	-
0.813011	8.30E-11	3.98E-09	Psme1		14	55578123	55581529	+
0.815901	1.97E-08	7.19E-07	Trim34a		7	1.04E+08	1.04E+08	+
0.815996	2.00E-14	1.32E-12	Gas7		11	67455437	67688990	+
0.816114	1.82E-11	9.51E-10	Zmynd15		11	70459433	70466202	+
0.817936	5.20E-15	3.55E-13	H2-K1		17	33996017	34000333	-
0.818423	5.29E-19	4.54E-17	Gpr162		6	1.25E+08	1.25E+08	-
0.823479	5.33E-07	1.59E-05	Pstpip2		18	77794545	77882007	+
0.825701	1.98E-06	5.40E-05	Helz2		2	1.81E+08	1.81E+08	-
0.827996	2.35E-06	6.31E-05	Slc7a11		3	49892526	50443614	-
0.614554	0.000526825	0.008191794	1700025G(		1	1.52E+08	1.52E+08	-
0.830782	1.23E-16	9.11E-15	Tapbp		17	33915899	33929288	+
0.832273	1.13E-10	5.39E-09	Mthfd2		6	83305691	83325908	-
0.8388	4.78E-12	2.60E-10	Casp4		9	5308828	5336783	+
0.839559	0.004506892	0.048088831	Pkd1l2		8	1.17E+08	1.17E+08	-
0.839956	5.55E-05	0.001133193	Eva1b		4	1.26E+08	1.26E+08	+
0.841295	8.43E-06	0.000204263	Fabp3		4	1.3E+08	1.3E+08	+
0.845146	6.33E-06	0.000157843	Scn1b		7	31116524	31127003	-
0.850071	8.21E-12	4.42E-10	Aldoc		11	78322968	78327781	+
0.850924	1.06E-16	7.95E-15	Fcgr1		3	96282909	96293969	-
0.854449	6.86E-09	2.66E-07	Arrdc4		7	68736995	68749241	-
0.855704	1.08E-07	3.60E-06	Filip1l		16	57353093	57573126	+
0.856067	0.000119085	0.002218235	Nfil3		13	52967209	52981073	-
0.858128	1.18E-10	5.60E-09	Spata13		14	60634001	60764556	+
0.858961	0.001245133	0.016911401	Cd101		3	1.01E+08	1.01E+08	-
0.859516	6.76E-07	1.98E-05	Nlrc5		8	94434356	94527272	+
0.863303	2.42E-12	1.35E-10	Dtx3l		16	35926511	35939151	-
0.863733	4.56E-11	2.26E-09	Ifih1		2	62595798	62646255	-

0.863924	4.23E-06	0.000109199	Ripk3	14	55784995	55788865	-
0.867049	6.45E-23	6.92E-21	Ralgds	2	28513125	28553081	+
0.869331	1.56E-06	4.31E-05	Nmi	2	51948487	51973494	-
0.8697	1.95E-08	7.16E-07	Rrad	8	1.05E+08	1.05E+08	-
0.874461	1.96E-07	6.35E-06	Trpm2	10	77907722	77970563	-
0.882682	0.000103753	0.001968043	Maff	15	79346621	79359076	+
0.887782	5.99E-10	2.63E-08	Ift57	16	49699233	49765126	+
0.889483	2.44E-05	0.000534895	P2ry13	3	59207892	59210882	-
0.891132	4.77E-14	3.03E-12	Sp110	1	85576899	85598817	-
0.891294	2.64E-06	7.05E-05	Cd86	16	36603869	36666081	-
0.892414	0.000447571	0.007112054	Col4a2	8	11312805	11449287	+
0.894341	2.36E-18	1.92E-16	Cxcl16	11	70453983	70459984	-
0.894373	0.000120973	0.002247618	Spic	10	88674772	88685015	-
0.897036	5.88E-13	3.47E-11	Tapbpl	6	1.25E+08	1.25E+08	-
0.898124	9.76E-21	9.43E-19	Fcgr2b	1	1.71E+08	1.71E+08	-
0.901055	9.85E-05	0.001890929	Chchd10	10	75933130	75937747	+
0.903572	1.76E-05	0.000400126	Rps27	3	90212522	90213651	-
0.906319	0.000122532	0.002267856	Il15ra	2	11705290	11734317	+
0.907196	0.000113591	0.002127834	St14	9	31089402	31131853	-
0.908423	1.79E-18	1.47E-16	Clec4a1	6	1.23E+08	1.23E+08	+
0.911324	0.000267472	0.004544196	Dtnb	12	3572381	3781796	+
0.91277	0.001776652	0.022427913	Ndufa12	10	94198720	94221443	+
0.916412	0.001061496	0.014826293	Gm2999	15	71794570	71795107	+
0.917412	3.93E-18	3.13E-16	Ptger4	15	5206661	5244187	-
0.918715	1.49E-18	1.24E-16	Il2rg	X	1.01E+08	1.01E+08	-
0.925118	1.26E-09	5.36E-08	Herc6	6	57581000	57664632	+
0.926087	1.66E-14	1.10E-12	Psmb8	17	34197721	34201454	+
0.931727	6.94E-05	0.001385757	Ceacam19	7	19875742	19887965	-
0.936342	5.39E-15	3.67E-13	Slfn8	11	83002158	83020810	-
-0.29484	0.000761322	0.011269738	1700017B0	9	57253117	57262612	-
0.940702	3.28E-23	3.57E-21	Trim30a	7	1.04E+08	1.04E+08	-
0.943252	3.55E-20	3.34E-18	Pbxip1	3	89436706	89450952	+
0.943328	1.46E-16	1.08E-14	Relb	7	19606217	19629438	-
0.943895	7.11E-11	3.42E-09	Rab20	8	11453518	11478710	-
0.944952	2.27E-10	1.05E-08	Psmb9	17	34181988	34187764	-
0.946171	6.82E-10	2.98E-08	Mfsd7a	5	1.08E+08	1.08E+08	-
0.947169	7.73E-20	7.04E-18	H2-T23	17	36029773	36032855	-
0.950192	0.000224431	0.003881204	Gm31544	13	1.2E+08	1.2E+08	+
0.954807	0.001112853	0.015374138	Ifi203-ps	1	1.74E+08	1.74E+08	-
0.954963	3.39E-14	2.17E-12	Slfn2	11	83065112	83070678	+
0.961081	2.76E-25	3.23E-23	Ms4a6c	19	11469366	11482192	+
0.961244	7.85E-08	2.66E-06	Dnaaf3	7	4522933	4532453	-
0.964097	3.15E-27	4.00E-25	Nfkb2	19	46304737	46312090	+
0.966528	0.00110644	0.015314741	Igfbp7	5	77349240	77408041	-
0.967823	3.00E-16	2.19E-14	Cd52	4	1.34E+08	1.34E+08	-
0.968211	1.88E-10	8.80E-09	Gm5431	11	48887422	48902214	-
0.97456	1.03E-06	2.93E-05	Batf	12	85686669	85709087	+
0.97853	0.000151192	0.002756065	Adgb	10	10335703	10472326	-
0.979578	8.76E-18	6.79E-16	Cd302	2	60251993	60284488	-
0.98052	5.90E-10	2.61E-08	BE692007	19	11470166	11484578	-

0.981169	2.42E-10	1.11E-08	Psmb10	8	1.06E+08	1.06E+08	-
0.987653	8.25E-06	0.000200331	Nod2	8	88647315	88688474	+
0.988319	6.33E-05	0.001280626	Gvin1	7	1.06E+08	1.06E+08	-
0.990411	0.00077053	0.011382799	Inpp4b	8	81342556	82127914	+
0.993106	0.000208152	0.00364317	Nedd4	9	72662346	72749852	+
0.994852	1.33E-18	1.11E-16	Itgal	7	1.27E+08	1.27E+08	+
0.996895	1.24E-30	1.87E-28	Ifi204	1	1.74E+08	1.74E+08	-
1.000414	0.001668772	0.021288594	Plbd1	6	1.37E+08	1.37E+08	-
1.000545	1.74E-08	6.43E-07	Tnfrsf14	4	1.55E+08	1.55E+08	-
1.00395	1.39E-11	7.32E-10	Itgb7	15	1.02E+08	1.02E+08	-
1.006558	2.01E-22	2.13E-20	Tap1	17	34187553	34197225	+
1.015539	0.003610425	0.040186077	Rnf213	11	1.19E+08	1.19E+08	+
1.016549	6.12E-29	8.45E-27	Sp100	1	85649988	85709998	+
1.018507	1.67E-32	2.72E-30	Lgals9	11	78962974	78984946	-
1.020262	2.10E-07	6.76E-06	Gpr68	12	1.01E+08	1.01E+08	-
1.025998	6.77E-11	3.27E-09	Gm6548	17	78850792	78851956	-
1.026064	2.91E-24	3.32E-22	Nfkbia	12	55489410	55492647	-
1.027559	1.59E-08	5.91E-07	Jdp2	12	85599027	85639878	+
1.030354	0.002605282	0.030472759	Chpf	1	75474569	75479307	-
1.033695	5.30E-07	1.59E-05	Oas1b	5	1.21E+08	1.21E+08	+
1.035617	4.90E-11	2.42E-09	Gpr141	13	19749682	19824257	-
1.044051	0.001551926	0.020152794	Col6a3	1	90765923	90843971	-
1.046491	5.82E-21	5.66E-19	Dck	5	88764996	88783281	+
1.047276	0.003713672	0.041177146	Gm6180	8	42246911	42247408	+
1.049755	3.62E-18	2.92E-16	Nlrp3	11	59541568	59566956	+
1.052922	2.35E-12	1.32E-10	Psd	19	46312087	46327156	-
1.057857	4.53E-11	2.25E-09	Cmklr1	5	1.14E+08	1.14E+08	-
1.061249	3.17E-22	3.30E-20	Orai2	5	1.36E+08	1.36E+08	-
1.06176	0.004161169	0.045002732	Gm10146	10	78393320	78393628	+
-1.0009	0.001009753	0.014207999	-	8	34683767	34689942	-
1.062606	3.95E-28	5.25E-26	Acsl1	8	46471037	46536051	+
1.068468	3.65E-43	9.12E-41	Ctsc	7	88278085	88310888	+
1.069354	7.57E-05	0.001496834	Mmp2	8	92827291	92853420	+
1.07456	0.001062195	0.014826293	Socs1	16	10782240	10785536	-
1.075351	5.78E-20	5.34E-18	Epsti1	14	77904239	78002657	+
1.075685	1.04E-08	3.92E-07	Gbp8	5	1.05E+08	1.05E+08	-
1.08538	4.99E-20	4.64E-18	Ube2l6	2	84798828	84810335	+
1.085784	3.78E-28	5.07E-26	Mndal	1	1.74E+08	1.74E+08	-
1.088429	2.47E-17	1.89E-15	Slfn5	11	82951349	82964840	+
1.090035	3.28E-19	2.84E-17	Gpr132	12	1.13E+08	1.13E+08	-
1.092987	1.43E-06	3.98E-05	Gm4070	7	1.06E+08	1.06E+08	-
1.110235	2.21E-08	8.04E-07	H2-Q5	17	35394126	35397800	+
1.119947	0.002392128	0.028368837	Cd300e	11	1.15E+08	1.15E+08	-
1.121579	1.98E-17	1.52E-15	C1qa	4	1.37E+08	1.37E+08	-
1.123176	4.68E-07	1.42E-05	Fn1	1	71585520	71653200	-
1.123649	9.63E-14	5.91E-12	Ifi203	1	1.74E+08	1.74E+08	-
1.124105	4.83E-40	1.05E-37	Lgals3bp	11	1.18E+08	1.18E+08	-
1.132564	1.28E-20	1.22E-18	Pot1b	17	55651951	55712628	-
1.133477	8.35E-07	2.41E-05	Gm10709	9	7751725	7752204	+
1.135612	0.001718638	0.021847804	Epop	11	97627389	97629702	-

1.136644	1.40E-25	1.66E-23	Clec5a	6	40574894	40585821	-
1.143399	0.000551846	0.008507815	Gm16418	1	1.66E+08	1.66E+08	-
1.146114	5.23E-18	4.10E-16	H2-DMa	17	34135182	34139101	+
1.149638	4.88E-28	6.43E-26	Parp9	16	35938470	35972605	+
1.152602	4.76E-09	1.89E-07	Htra1	7	1.31E+08	1.31E+08	+
1.155997	1.36E-09	5.74E-08	Gm11808	4	3973092	3973595	-
1.161315	1.40E-32	2.30E-30	Birc3	9	7848699	7873186	-
1.161403	0.000395313	0.006422499	Gm47567	12	98277517	98281040	+
1.164209	3.17E-32	5.00E-30	Stat2	10	1.28E+08	1.28E+08	+
1.169816	0.000196266	0.003456006	Chst15	7	1.32E+08	1.32E+08	-
1.170538	3.78E-30	5.53E-28	Apobec3	15	79891659	79915906	+
1.172252	4.21E-19	3.63E-17	Cd74	18	60803848	60812646	+
1.173462	0.000280703	0.004735678	Cald1	6	34598500	34775473	+
1.174066	8.02E-25	9.30E-23	Mefv	16	3707218	3718097	-
1.174698	8.00E-12	4.33E-10	Coq8a	1	1.8E+08	1.8E+08	-
1.180185	1.45E-20	1.38E-18	Slamf8	1	1.73E+08	1.73E+08	-
1.181868	1.16E-24	1.34E-22	Nfkbie	17	45555703	45563169	+
1.188673	8.74E-13	5.06E-11	Phf11d	14	59347407	59365470	-
1.198953	0.000215546	0.003754441	Clec2i	6	1.29E+08	1.29E+08	+
1.203288	1.36E-23	1.52E-21	Trim30d	7	1.04E+08	1.05E+08	-
1.207183	2.60E-14	1.69E-12	Foxp4	17	47867133	47924645	-
1.218053	5.48E-31	8.36E-29	H2-Q4	17	35379617	35385290	+
1.220542	4.20E-06	0.000108711	Bst1	5	43818885	43843986	+
1.225171	9.22E-23	9.83E-21	Irgm2	11	58199618	58222782	+
1.226344	9.10E-17	6.84E-15	Igtp	11	58199556	58207591	+
1.228049	5.90E-14	3.72E-12	Ifi35	11	1.01E+08	1.01E+08	+
1.232983	2.61E-29	3.67E-27	Ehd1	19	6276725	6300096	+
1.241227	1.22E-35	2.13E-33	Gm21188	13	1.2E+08	1.2E+08	-
1.243248	8.44E-05	0.001643846	Ccl2	11	82035571	82037453	+
0.797482	0.001288725	0.017308801	1110002J0	10	66912489	66920258	-
1.244397	3.04E-28	4.12E-26	Fgr	4	1.33E+08	1.33E+08	+
1.252556	0.000328752	0.005463609	Il1a	2	1.29E+08	1.29E+08	-
1.259813	5.64E-22	5.76E-20	Prdx5	19	6906697	6910106	-
1.259881	9.08E-14	5.60E-12	Psme2	14	55587441	55591113	-
1.261408	0.000900367	0.013008885	Adora2b	11	62248984	62266453	+
1.262622	0.00278741	0.032161738	Hcar2	5	1.24E+08	1.24E+08	-
1.263002	8.41E-19	7.09E-17	Aif1	17	35170991	35176068	-
1.26328	3.11E-32	4.95E-30	Sdc1	12	8771323	8793715	+
1.266332	1.00E-09	4.32E-08	Ly75	2	60292103	60383303	-
1.267482	6.00E-27	7.50E-25	Cd14	18	36725074	36726736	-
1.267578	3.45E-18	2.79E-16	Ednrb	14	1.04E+08	1.04E+08	-
1.267704	1.83E-07	5.97E-06	Hpn	7	31098725	31115290	-
1.268173	2.51E-07	8.01E-06	Serpine1	5	1.37E+08	1.37E+08	-
1.27226	3.56E-10	1.61E-08	Fads2	19	10064164	10101503	-
1.273526	6.18E-27	7.66E-25	H2-Aa	17	34282744	34287827	-
1.282254	1.88E-10	8.80E-09	Slco3a1	7	74275419	74554780	-
1.290425	4.61E-18	3.63E-16	Pnp	14	50931082	50965237	+
1.291383	6.10E-05	0.001239491	C1rl	6	1.24E+08	1.25E+08	+
1.293141	6.56E-36	1.16E-33	Irf1	11	53770014	53778374	+
1.295875	7.95E-17	6.00E-15	Ddx60	8	61928087	62038244	+

1.297391	0.001544164	0.020087991	Col12a1	9	79598991	79718831	-
1.307138	0.000981813	0.013935778	Rtn2	7	19282624	19296160	+
1.31635	5.80E-11	2.83E-09	Csf1	3	1.08E+08	1.08E+08	-
1.324691	5.68E-14	3.60E-12	Slco4a1	2	1.8E+08	1.8E+08	+
1.330139	5.43E-07	1.61E-05	Spaca6	17	17827158	17843009	+
1.333356	0.000503874	0.007902751	Gm17705	17	35165119	35167186	+
1.335184	7.06E-20	6.47E-18	Rnd3	2	51130438	51149111	-
1.335517	6.79E-08	2.33E-06	Fzd1	5	4753839	4758035	-
1.338592	7.60E-24	8.54E-22	Tma16	8	66473118	66486530	-
1.343319	7.46E-19	6.33E-17	Pald1	10	61319656	61383530	-
1.344375	0.000271592	0.004598024	Gm12174	11	46727818	46728630	+
1.347896	6.21E-39	1.27E-36	Irgm1	11	48861968	48871683	-
1.348714	1.10E-09	4.68E-08	Batf2	19	6140983	6172476	+
1.35012	9.24E-53	2.85E-50	Vasp	7	19256929	19271817	-
1.354272	1.36E-05	0.000316548	Rtn1	12	72211752	72409054	-
1.357495	2.39E-14	1.56E-12	Ciita	16	10480059	10528418	+
1.360255	1.64E-12	9.38E-11	C1ra	6	1.25E+08	1.25E+08	+
1.36214	2.09E-10	9.79E-09	Kcnj10	1	1.72E+08	1.72E+08	+
1.365144	5.30E-28	6.92E-26	Gm36161	13	1.2E+08	1.2E+08	+
1.366767	2.41E-23	2.67E-21	Bst2	8	71534255	71537456	-
1.367184	1.61E-33	2.74E-31	Clec4n	6	1.23E+08	1.23E+08	+
1.373179	2.37E-07	7.58E-06	Gbgt1	2	28496891	28505415	+
1.383306	0.001249318	0.016933424	Klra2	6	1.31E+08	1.31E+08	-
1.386536	6.14E-11	2.99E-09	Cav1	6	17306335	17341452	+
1.386815	1.48E-26	1.80E-24	H2-T24	17	36005695	36020560	-
1.389319	2.59E-05	0.000566996	Gm1966	7	1.07E+08	1.07E+08	-
1.395061	1.05E-36	1.91E-34	Bcl3	7	19808462	19822770	-
1.395232	2.48E-44	6.65E-42	Lmo4	3	1.44E+08	1.44E+08	-
1.395386	0.000201843	0.003541299	Ctgf	10	24595442	24598683	+
1.402338	2.91E-10	1.33E-08	Rbpms	8	33782643	33929863	-
1.404207	1.18E-42	2.90E-40	Lpcat2	8	92855339	92919279	+
1.407459	3.77E-11	1.88E-09	Shb	4	45423278	45532470	-
1.41715	2.70E-21	2.65E-19	Fgl2	5	21372642	21378374	+
1.426598	1.30E-14	8.63E-13	Mturn	6	54681624	54703851	+
1.437871	2.54E-27	3.29E-25	Ifi211	1	1.74E+08	1.74E+08	-
1.438854	1.74E-41	4.07E-39	Sema4a	3	88435959	88461182	-
1.439369	3.98E-50	1.15E-47	Ms4a6b	19	11516512	11531256	+
1.466825	1.15E-37	2.22E-35	Arl5c	11	97989578	97996181	-
1.467165	1.06E-45	2.89E-43	Oas2	5	1.21E+08	1.21E+08	-
1.468787	7.17E-14	4.44E-12	Ahrr	13	74211118	74292331	-
1.473303	0.004205493	0.045349672	Slamf6	1	1.72E+08	1.72E+08	+
1.473444	1.13E-05	0.000266786	Tnc	4	63959785	64047015	-
1.474918	4.49E-05	0.000932023	Gm21370	13	1.2E+08	1.2E+08	-
1.486393	1.17E-51	3.52E-49	Ddx58	4	40203773	40239828	-
1.489613	0.004722232	0.049662257	Ccl7	11	82045712	82047525	+
1.496835	0.000477993	0.007537619	Ccnd2	6	1.27E+08	1.27E+08	-
1.497078	0.004649604	0.049189709	Fam187b	7	30973790	30989726	+
1.509524	1.22E-21	1.24E-19	Phf11b	14	59320964	59341351	-
1.510104	3.76E-37	6.99E-35	Oas1a	5	1.21E+08	1.21E+08	-
1.510533	6.81E-07	1.99E-05	Cd55	1	1.3E+08	1.3E+08	-

1.510785	4.37E-29	6.09E-27	Cebpd	16	15887286	15891031	+
1.521704	8.27E-38	1.64E-35	Stat1	1	52119440	52161865	+
1.533278	1.51E-39	3.23E-37	Dhx58	11	1.01E+08	1.01E+08	-
1.53631	6.88E-09	2.67E-07	Rnase6	14	51123908	51132187	+
1.546869	3.57E-08	1.26E-06	Aebp1	11	5861947	5872088	+
1.547895	0.001948583	0.024218577	Plpp1	13	1.13E+08	1.13E+08	+
1.557175	6.62E-26	7.93E-24	Pde4b	4	1.02E+08	1.03E+08	+
1.560808	4.96E-35	8.56E-33	H2-Eb1	17	34305877	34316199	+
1.565639	1.04E-06	2.94E-05	Acpp	9	1.04E+08	1.04E+08	-
1.566126	0.000426252	0.006798704	Lamb1	12	31265234	31329644	+
1.566714	0.001169615	0.015990628	Gm10134	2	28506095	28506475	+
-0.38797	0.001865536	0.023286256	-	2	1.44E+08	1.44E+08	+
1.568889	0.000595994	0.009053614	Slfn4	11	83175186	83190216	+
1.569804	0.000415167	0.006670284	Trim30b	7	1.04E+08	1.04E+08	-
1.570039	2.65E-21	2.62E-19	Cdc42ep2	19	5917556	5924816	-
1.570309	1.56E-21	1.56E-19	Spn	7	1.27E+08	1.27E+08	-
1.571999	9.80E-20	8.83E-18	Ier3	17	35821684	35822923	+
1.575239	9.57E-15	6.42E-13	Sparc	11	55394500	55423183	-
1.581442	0.002345963	0.027958635	Cyr61	3	1.46E+08	1.46E+08	-
1.58541	0.002039759	0.025042208	Glipr2	4	43957401	43979118	+
1.587041	2.62E-14	1.70E-12	Pilrb2	5	1.38E+08	1.38E+08	-
1.58722	0.001408864	0.018731427	Clmp	9	40685962	40785319	+
1.596626	2.17E-09	9.05E-08	Isg20	7	78913424	78920396	+
1.5993	0.003024781	0.034272964	Rab38	7	88430273	88491572	+
1.605783	3.23E-11	1.63E-09	Bgn	X	73483602	73495933	+
1.631503	3.74E-71	1.94E-68	Tlr2	3	83836272	83841767	-
1.631818	7.13E-47	1.99E-44	Oas3	5	1.21E+08	1.21E+08	-
1.637858	2.16E-37	4.12E-35	Xaf1	11	72301629	72313733	+
1.638392	3.94E-14	2.52E-12	Nupr1	7	1.27E+08	1.27E+08	-
1.640068	1.71E-15	1.20E-13	Cp	3	19957054	20009145	+
1.658435	1.52E-12	8.68E-11	Cd40	2	1.65E+08	1.65E+08	+
1.659256	6.18E-11	2.99E-09	Enpp4	17	44096308	44105809	-
1.661287	4.14E-22	4.26E-20	Icosl	10	78069302	78083913	+
1.66489	2.99E-07	9.37E-06	Col6a1	10	76708792	76726168	-
1.668335	1.05E-85	7.26E-83	Mmp14	14	54431612	54445364	+
1.670387	5.16E-30	7.48E-28	Traf1	2	34941750	34961772	-
1.672774	0.000122765	0.002269266	Postn	3	54361109	54391037	+
1.683727	0.000636	0.00954132	Nid1	13	13437551	13512269	+
1.700422	9.93E-77	6.00E-74	Sod2	17	13006846	13040063	+
1.702405	2.82E-41	6.29E-39	H2-Ab1	17	34263209	34269418	+
1.702786	9.08E-10	3.94E-08	Acta2	19	34241090	34255336	-
1.704966	0.002205226	0.026670687	Sned1	1	93235841	93301065	+
1.709884	0.004660328	0.049189709	Ltb	17	35194439	35196320	+
1.71616	1.64E-05	0.000376289	Arhgef37	18	61493794	61536536	-
1.718516	0.004158644	0.045002732	Timp1	X	20870166	20874735	+
1.722674	2.48E-12	1.38E-10	Atrnl1	19	57611034	58133338	+
1.736061	3.14E-90	2.40E-87	Icam1	9	21015985	21028817	+
1.738188	9.38E-15	6.32E-13	Ptges	2	30889471	30929863	-
1.751887	3.71E-53	1.19E-50	Tmem176b	6	48833818	48841496	-
1.755762	2.87E-86	2.08E-83	Aoah	13	20794113	21036617	+

1.76161	5.36E-09	2.11E-07	Serpinh1	7	99345376	99353239	-
1.800306	0.002133921	0.025900148	Prss34	17	25298394	25300161	+
1.801016	0.000101139	0.001930253	Asb2	12	1.03E+08	1.03E+08	-
1.806858	1.11E-37	2.18E-35	Oas1g	5	1.21E+08	1.21E+08	-
1.809247	0.004572372	0.048579781	Kmo	1	1.76E+08	1.76E+08	+
1.832831	7.15E-06	0.000176286	Gm11772	11	1.2E+08	1.2E+08	-
1.833305	7.78E-41	1.71E-38	Il21r	7	1.26E+08	1.26E+08	+
1.833407	5.19E-33	8.65E-31	Slc39a4	15	76612383	76617384	-
1.844137	0.000297167	0.004990206	Fat1	8	44935447	45052257	+
1.84651	1.13E-10	5.37E-09	Col5a2	1	45374321	45503282	-
1.860052	6.70E-56	2.31E-53	Ifitm3	7	1.41E+08	1.41E+08	-
1.861965	0.003664058	0.040751754	Gm9574	17	36137188	36139105	-
1.866617	1.14E-07	3.79E-06	Ifitm6	7	1.41E+08	1.41E+08	-
1.870048	5.32E-38	1.07E-35	Thbs1	2	1.18E+08	1.18E+08	+
1.900608	5.67E-23	6.13E-21	Calhm6	10	34126067	34127984	-
1.90172	0.000171229	0.003067309	Mrc2	11	1.05E+08	1.05E+08	+
1.907009	8.59E-62	3.77E-59	Ifi209	1	1.74E+08	1.74E+08	+
1.912807	2.53E-12	1.41E-10	Fxyd2	9	45399669	45410278	+
1.91585	8.27E-32	1.29E-29	H2-Q6	17	35424850	35430055	+
1.91937	3.50E-05	0.000743948	AC113595.	15	79889532	79893120	+
1.924271	6.18E-57	2.30E-54	Tmem176a	6	48840919	48847071	+
1.932361	6.52E-16	4.63E-14	Fas	19	34290659	34327770	+
1.933939	9.43E-09	3.59E-07	Fbn1	2	1.25E+08	1.26E+08	-
1.939541	3.60E-22	3.73E-20	Smpdl3b	4	1.33E+08	1.33E+08	-
1.947882	5.42E-59	2.18E-56	Cmpk2	12	26469204	26479837	+
1.952276	0.000506554	0.007927631	Pnp2	14	50955992	50964749	+
1.958252	8.02E-10	3.50E-08	Mmp9	2	1.65E+08	1.65E+08	+
1.963134	3.44E-05	0.000732671	Loxl1	9	58287738	58313186	-
1.972982	0.000924171	0.013231901	Fkbp10	11	1E+08	1E+08	+
1.985812	2.18E-05	0.000486592	Antxr1	6	87133853	87335821	-
1.987171	8.95E-06	0.000215075	Gpr18	14	1.22E+08	1.22E+08	-
2.035682	7.60E-06	0.000186674	Vcan	13	89655312	89742509	-
2.041431	0.004074706	0.044232688	Efna2	10	80179482	80190010	+
2.062979	0.003349279	0.037509858	Apol7c	15	77524852	77533316	-
2.063147	5.12E-07	1.54E-05	Adora2a	10	75316877	75334784	+
2.075069	7.80E-13	4.55E-11	Fbln2	6	91212455	91272540	+
2.080423	2.75E-06	7.32E-05	Col6a2	10	76595762	76623630	-
2.086896	9.69E-57	3.51E-54	Rsad2	12	26442746	26456452	-
2.104388	1.37E-118	1.99E-115	Oasl2	5	1.15E+08	1.15E+08	+
2.10443	6.34E-117	8.35E-114	Tgfbi	13	56609523	56639562	+
2.110356	0.001716685	0.021842139	Ccl22	8	94745590	94751699	+
2.111169	0.001288555	0.017308801	Gja1	10	56377330	56402513	+
2.115459	6.40E-06	0.000159114	Cyp1b1	17	79706953	79715041	-
2.117016	1.86E-20	1.76E-18	Ccr2	9	1.24E+08	1.24E+08	+
2.123475	2.97E-23	3.26E-21	Gm5424	10	62071123	62072362	+
2.135686	4.43E-07	1.36E-05	Trim30c	7	1.04E+08	1.04E+08	-
2.147938	1.01E-11	5.39E-10	Cst7	2	1.51E+08	1.51E+08	+
2.150265	7.71E-07	2.24E-05	Gm34643	14	1.03E+08	1.03E+08	+
2.155704	0.001143059	0.015671918	Slc6a13	6	1.21E+08	1.21E+08	+
2.158916	0.000573578	0.008805391	Ms4a4b	19	11443553	11463549	+

2.169632	2.06E-16	1.51E-14	Mx2	16	97535308	97560900	+
2.17991	0.002725855	0.031602471	Chst1	2	92599707	92615250	+
2.181714	0.000207452	0.003635302	F830016B0	18	60293380	60303016	+
2.184944	2.03E-19	1.78E-17	F13a1	13	36867178	37050244	-
2.189398	3.43E-08	1.22E-06	Slc13a3	2	1.65E+08	1.65E+08	-
2.192181	0.001088425	0.01512316	Cacnb3	15	98630840	98644529	+
2.196716	1.44E-07	4.71E-06	Dcn	10	97479609	97518143	+
2.199838	2.52E-21	2.50E-19	Gbp7	3	1.43E+08	1.43E+08	+
2.204599	0.001127283	0.015514328	Bcl2a1a	9	88956900	88962419	+
2.228773	0.003451204	0.038532245	Tbc1d10c	19	4184358	4191047	-
2.23625	1.99E-32	3.20E-30	H2-Q7	17	35439155	35443773	+
2.242526	0.000608793	0.009199826	Grb10	11	11930508	12038683	-
2.245648	8.74E-56	2.95E-53	Ifi47	11	49076587	49096974	+
2.291528	1.26E-42	3.03E-40	Gm12250	11	58187739	58189012	+
2.303788	1.03E-41	2.45E-39	Gbp3	3	1.43E+08	1.43E+08	+
2.305965	0.000210884	0.003682093	AC154707.	17	22067264	22069888	-
2.317231	3.77E-31	5.81E-29	H2-M2	17	37480851	37483552	-
2.348733	8.64E-06	0.000209029	Tnfsf10	3	27317028	27342427	+
2.372588	7.24E-09	2.79E-07	Phf11a	14	59276913	59297522	-
2.373104	5.02E-07	1.51E-05	Pdgfrb	18	61045150	61085061	+
2.391896	2.49E-13	1.50E-11	Col5a1	2	27886425	28039514	+
2.425081	1.52E-05	0.000352384	Cd38	5	43868553	43912375	+
2.426448	1.31E-17	1.01E-15	Uchl1	5	66676091	66687234	+
2.437746	0.000914422	0.013133605	Vnn3	10	23851462	23869843	+
2.445665	5.08E-26	6.14E-24	Siglece	7	43651070	43660161	-
2.547087	3.92E-36	7.02E-34	Cxcl2	5	90903871	90905938	+
2.558038	6.64E-71	3.21E-68	Ifi27l2a	12	1.03E+08	1.03E+08	-
2.583138	0.000247556	0.004235638	Il12rb1	8	70808449	70821424	+
2.602999	2.12E-14	1.39E-12	Phgdh	3	98313170	98339990	-
2.62142	0.001591292	0.020535175	Capns2	8	92901407	92902411	+
2.631479	4.28E-16	3.05E-14	Ifi205	1	1.74E+08	1.74E+08	-
2.642331	1.54E-98	1.49E-95	Rtp4	16	23520291	23614222	+
2.643558	3.71E-24	4.20E-22	Pilrb1	5	1.38E+08	1.38E+08	-
2.646696	1.70E-06	4.68E-05	Thbs2	17	14665500	14694235	-
2.65753	6.13E-06	0.000153383	Col5a3	9	20770050	20815067	-
2.671432	0.003951767	0.043221891	Art2a-ps	7	1.02E+08	1.02E+08	-
2.675495	2.80E-13	1.69E-11	Fscn1	5	1.43E+08	1.43E+08	+
2.677998	3.32E-62	1.50E-59	Usp18	6	1.21E+08	1.21E+08	+
2.68485	1.96E-43	5.16E-41	Col1a2	6	4504814	4541544	+
2.688794	2.49E-41	5.64E-39	Isg15	4	1.56E+08	1.56E+08	-
2.69802	0.003010203	0.03418798	Shisa3	5	67607882	67623552	+
2.702593	1.81E-148	6.54E-145	C3	17	57203970	57228136	-
2.711576	3.54E-114	4.28E-111	Ly6e	15	74955051	74959905	+
2.728049	4.27E-11	2.13E-09	Ptgs2	1	1.5E+08	1.5E+08	+
2.73772	0.000298513	0.005006998	Olfr56	11	48978889	49135387	+
2.746181	2.85E-27	3.65E-25	Slc6a12	6	1.21E+08	1.21E+08	+
2.751721	0.000580352	0.008881161	Inhbb	1	1.19E+08	1.19E+08	-
2.764556	9.73E-07	2.78E-05	Mcemp1	8	3665754	3669259	+
2.789229	5.08E-93	4.09E-90	Slc2a6	2	27021363	27027998	-
2.808559	6.32E-08	2.17E-06	Cxcl1	5	90891241	90893115	+

2.812844	3.77E-18	3.02E-16	Gbp6	5	1.05E+08	1.05E+08	-
2.833119	0.000277692	0.004695815	Cish	9	1.07E+08	1.07E+08	+
2.84032	2.30E-33	3.87E-31	AW112010	19	11047617	11050566	-
2.840565	4.50E-84	2.84E-81	Zbp1	2	1.73E+08	1.73E+08	-
2.858854	5.79E-55	1.91E-52	Ass1	2	31470207	31520672	+
2.882459	1.86E-12	1.06E-10	Arg2	12	79130777	79156301	+
2.890054	1.25E-30	1.87E-28	Slpi	2	1.64E+08	1.64E+08	-
2.909178	3.06E-58	1.17E-55	Col1a1	11	94936224	94953042	+
2.916581	1.63E-64	7.63E-62	Mx1	16	97447035	97462907	-
2.921701	3.15E-15	2.18E-13	Slfn1	11	83116849	83122670	+
2.955539	1.01E-50	2.98E-48	Oasl1	5	1.15E+08	1.15E+08	+
2.963178	5.37E-137	1.56E-133	Irf7	7	1.41E+08	1.41E+08	-
3.021518	4.31E-71	2.16E-68	Pilra	5	1.38E+08	1.38E+08	-
3.053347	3.60E-13	2.15E-11	Cd69	6	1.29E+08	1.29E+08	-
3.079932	2.35E-41	5.41E-39	Gpr84	15	1.03E+08	1.03E+08	-
3.081526	0.001116013	0.015403101	Adamts2	11	50602084	50807573	+
3.084514	1.31E-107	1.36E-104	Ifit2	19	34550694	34576419	+
3.089937	0.000162089	0.002939922	Spns3	11	72494919	72550506	-
3.091026	5.76E-85	3.79E-82	Cx3cr1	9	1.2E+08	1.2E+08	-
3.102268	1.35E-08	5.05E-07	Serpinf1	11	75409769	75422701	-
3.130246	2.82E-28	3.86E-26	Gm4951	18	60212080	60247820	+
3.13618	3.88E-08	1.36E-06	BC147527	13	1.2E+08	1.2E+08	+
3.197724	2.11E-10	9.84E-09	Cxcl3	5	90786103	90789600	+
3.198214	3.26E-08	1.17E-06	Tarm1	7	3486500	3502624	-
3.213907	2.86E-58	1.12E-55	Ifi44	3	1.52E+08	1.52E+08	-
3.215272	0.000421836	0.006754962	Tlr5	1	1.83E+08	1.83E+08	+
3.225742	7.91E-113	8.82E-110	Marcksl1	4	1.3E+08	1.3E+08	+
3.226529	2.29E-30	3.38E-28	Ifit3b	19	34607970	34613401	+
3.23959	0.000354586	0.005839382	Cemip	7	83932857	84086502	-
3.25556	1.41E-96	1.20E-93	Ifit1	19	34640871	34650009	+
3.281606	2.19E-22	2.30E-20	Col3a1	1	45311538	45349706	+
3.310685	2.20E-39	4.62E-37	Socs3	11	1.18E+08	1.18E+08	-
3.317834	3.54E-120	5.70E-117	Ifit3	19	34583531	34588731	+
3.321525	3.07E-05	0.000662964	Cd248	19	5068078	5070637	+
3.3331	2.43E-120	4.41E-117	Gbp2	3	1.43E+08	1.43E+08	+
3.361833	2.30E-39	4.77E-37	Tgtp2	11	49057194	49064206	-
3.457308	6.24E-53	1.97E-50	Ifi206	1	1.73E+08	1.73E+08	-
3.537631	6.10E-37	1.12E-34	Ms4a4c	19	11404770	11427246	+
3.539629	1.16E-08	4.34E-07	Ifit1bl1	19	34592888	34601968	-
3.566259	3.74E-08	1.31E-06	Cxcl9	5	92321347	92328079	-
3.594211	1.71E-255	8.27E-252	Clec4e	6	1.23E+08	1.23E+08	-
3.672202	0.00023605	0.004062753	Il12b	11	44400063	44414033	+
3.688985	4.71E-56	1.66E-53	Gbp5	3	1.42E+08	1.43E+08	+
3.731323	0.000164884	0.002972021	Gm19026	1	6429655	6441296	-
3.777842	9.67E-27	1.19E-24	Ifi208	1	1.74E+08	1.74E+08	+
3.794806	2.65E-11	1.35E-09	Spint2	7	29256323	29281912	-
3.806797	3.33E-13	1.99E-11	Rasgrp1	2	1.17E+08	1.17E+08	-
3.825234	3.31E-75	1.84E-72	Ifi213	1	1.74E+08	1.74E+08	-
3.8317	0.002935386	0.033431123	Gas1	13	60174405	60177365	-
3.841411	2.77E-07	8.74E-06	Mycl	4	1.23E+08	1.23E+08	+

3.858756	0.000315775	0.005263297	Flrt3	2	1.41E+08	1.41E+08	-
3.931273	1.72E-61	7.14E-59	Hp	8	1.1E+08	1.1E+08	-
4.002221	1.09E-12	6.27E-11	Lcn2	2	32384633	32388252	-
4.017888	0.001630476	0.020892	Col8a1	16	57624258	57754737	-
4.133636	2.83E-07	8.90E-06	Susd2	10	75636706	75644008	-
4.156227	0.001024248	0.014383148	Apol9b	15	77729039	77736382	+
4.157671	5.65E-18	4.40E-16	Ppm1n	7	19276805	19280064	-
4.249141	1.19E-06	3.35E-05	Serpina3f	12	1.04E+08	1.04E+08	+
4.262623	1.70E-29	2.41E-27	Ly6c2	15	75108158	75111970	-
4.318699	8.98E-76	5.20E-73	Fpr2	17	17887824	17893952	+
4.320725	3.28E-125	6.79E-122	Marco	1	1.2E+08	1.21E+08	-
4.398911	7.82E-13	4.55E-11	Gbp4	5	1.05E+08	1.05E+08	-
4.470371	9.51E-132	2.30E-128	Ccl5	11	83525778	83530518	-
4.511181	3.54E-43	8.99E-41	Cxcl10	5	92346638	92348889	-
4.518261	1.64E-61	7.01E-59	Fpr1	17	17876471	17883940	-
4.533771	1.21E-07	4.00E-06	Lpar1	4	58435255	58553898	-
4.571708	1.65E-73	8.85E-71	Ly6a	15	74994877	74998031	-
4.654104	0.001832305	0.022930715	Gm13822	5	1.15E+08	1.15E+08	-
4.689558	4.83E-49	1.37E-46	Il1b	2	1.29E+08	1.29E+08	-
4.767374	1.32E-97	1.20E-94	Adgre4	17	55749984	55853662	+
4.818906	1.60E-296	2.31E-292	Acod1	14	1.03E+08	1.03E+08	+
4.863347	0.000542619	0.008392354	Ptx3	3	66219910	66225805	+
4.865779	0.000602305	0.009130336	Cdkn2a	4	89274471	89294653	-
4.93216	2.35E-13	1.43E-11	Serpina3g	12	1.04E+08	1.04E+08	+
5.021585	2.03E-43	5.25E-41	Ly6i	15	74979534	74983430	-
5.269289	4.59E-06	0.000117516	Cacng8	7	3390683	3415648	+
5.288407	3.13E-37	5.88E-35	Iigp1	18	60376029	60392627	+
5.715206	1.94E-05	0.000436914	Gm5970	18	60220843	60222058	+
6.049626	3.19E-06	8.45E-05	Lad1	1	1.36E+08	1.36E+08	+
6.339511	5.86E-07	1.73E-05	Cfb	17	34856374	34862518	-
6.725187	1.43E-276	1.04E-272	Saa3	7	46711998	46715700	-

gene_lengt	gene_biotype	gene_desci	tf_family
8074	protein_coding	ectonucleo	-
2769	protein_coding	heat shock	-
10553	protein_coding	doublecort	-
2990	protein_coding	solute carri	-
3871	TEC	predicted g	-
432	TR_V_gene	T cell recep	-
5783	TEC	predicted g	-
362	antisense	predicted g	-
3927	protein_coding	nanos C2H(	-
1667	lincRNA	predicted g	-
7317	protein_coding	dachshund	-
2784	protein_coding	serine/argi	-
4531	protein_coding	solute carri	-
2243	antisense	predicted g	-
5217	protein_coding	neuronal p	-
5232	protein_coding	sphingomy	-
1452	lincRNA	predicted g	-
3611	protein_coding	phosphoglu	-
6772	protein_coding	high mobili	HMGI/HMGY
6325	protein_coding	family with	-
1424	protein_coding	carboxyme	-
385	TR_V_gene	T cell recep	-
6497	protein_coding	carbohydra	-
5958	protein_coding	solute carri	-
7322	protein_coding	kelch-like 3	-
4564	protein_coding	solute carri	-
4230	protein_coding	prokineticii	-
10382	protein_coding	EFR3 homc	-
1385	antisense	DNA methy	-
4795	protein_coding	SRY (sex de	HMG
1250	protein_coding	fatty acid b	-
4295	processed_transcript	predicted g	-
16067	protein_coding	enhancer tı	-
6286	protein_coding	angiopoieti	-
1953	protein_coding	predicted g	-
2317	lincRNA	predicted g	-
3401	protein_coding	TOX high m	HMG
9651	protein_coding	xylosyltran	-
5230	protein_coding	leucine rich	-
4921	protein_coding	RAS, guany	-
6637	protein_coding	family with	-
5242	protein_coding	shisa family	-
3277	protein_coding	neuronal P.	-
799	processed_pseudogene	predicted g	-
5497	protein_coding	solute carri	-
6686	protein_coding	gamma-am	-
9875	protein_coding	fibroblast g	-
10660	protein_coding	cytidine mo	-
12451	protein_coding	ArfGAP wit	-

4532	protein_coding	frizzled clas	-
10007	protein_coding	TOPBP1-int	-
3338	protein_coding	MAM dom	-
3202	protein_coding	cell divisior	-
8911	protein_coding	KIT proto-c	-
5935	protein_coding	epithelial n	-
6484	protein_coding	ras homolc	-
4085	protein_coding	arachidona	-
5401	protein_coding	solute carri	-
6730	protein_coding	junctional (	-
5447	lincRNA	RIKEN cDN.	-
12128	protein_coding	Rho GTPas	-
3077	antisense	predicted g	-
2197	protein_coding	glycoprotei	-
4791	protein_coding	neuronal P.	bHLH
4709	protein_coding	cyclin D1 [S	-
5786	lincRNA	RIKEN cDN.	-
4630	protein_coding	G protein-c	-
4442	protein_coding	family with	-
456	processed_pseudogene	predicted g	-
1143	lincRNA	predicted g	-
4210	protein_coding	seizure rela	-
4291	protein_coding	purinergic r	-
3005	protein_coding	patched dc	-
17717	protein_coding	low density	-
4522	protein_coding	synuclein, a	-
14665	protein_coding	histone dea	-
3398	protein_coding	early growt	zf-C2H2
10966	protein_coding	amyloid be	-
5197	protein_coding	adenylate (	-
2262	protein_coding	serine (or c	-
6858	protein_coding	DENN/MAI	-
40491	protein_coding	spectrin re	-
5548	protein_coding	spectrin re	-
2474	protein_coding	tetraspanir	-
8048	protein_coding	ATP-bindin	-
4491	protein_coding	polo like ki	-
8658	protein_coding	sterile alph	-
9935	protein_coding	piezo-type	-
9383	protein_coding	ATP-bindin	-
6596	protein_coding	N-myc dow	-
8296	protein_coding	RALBP1 ass	-
6859	lincRNA	predicted g	-
1712	protein_coding	transmemb	-
6390	protein_coding	ankyrin rep	-
2210	protein_coding	matrix met	-
13709	protein_coding	denticleles	-
6392	protein_coding	FERM dom	-
5911	protein_coding	kit ligand [S	-
3465	protein_coding	purinergic r	-

7237	protein_coding	matrix met	-
4281	protein_coding	dehydroge	-
2616	protein_coding	ankyrin rep	-
12961	protein_coding	huntingtin	-
3899	protein_coding	G protein-c	-
7844	protein_coding	cingulin-lik	-
5161	protein_coding	anaphase p	-
7816	protein_coding	cytoplasmi	-
2721	protein_coding	hepatitis A	-
5901	protein_coding	integrin be	-
8705	protein_coding	DOT1-like,	-
3839	protein_coding	ankyrin rep	-
3812	protein_coding	LON peptid	-
4663	protein_coding	interleukin	-
1850	protein_coding	predicted g	-
3921	-	PF00429:EI	-
9401	protein_coding	oxysterol b	-
11418	protein_coding	calcium cha	-
2716	protein_coding	ATPase, H+	-
6837	protein_coding	microfibrill	-
11345	protein_coding	metastasis	-
3590	protein_coding	CD36 mole	-
12351	protein_coding	pseudopod	-
10478	protein_coding	lipoprotein	-
8193	protein_coding	multiple C2	-
5527	protein_coding	carbohydra	-
6122	protein_coding	CD28 antig	-
1083	protein_coding	pro-platele	-
9978	protein_coding	ribosomal p	-
7880	protein_coding	plexin A3 [S	-
17822	protein_coding	jumonji do	-
5704	protein_coding	myosin IE [	-
8476	protein_coding	E26 avian l	ETS
3561	protein_coding	compleme	-
3550	protein_coding	ATP binding	-
5871	protein_coding	sulfatase 2	-
4638	protein_coding	a disintegri	-
3286	protein_coding	angiopoieti	-
10514	protein_coding	low density	-
16102	protein_coding	aspartate-b	-
2367	protein_coding	plasminoge	-
2717	protein_coding	myelocyto	bHLH
7901	protein_coding	kinesin fam	-
4434	protein_coding	Rho GTPas	-
3946	protein_coding	tropomodu	-
3419	protein_coding	WEE 1 hom	-
4906	protein_coding	potassium	-
1569	sense_intronic	predicted g	-
9079	protein_coding	zinc finger,	-
3777	protein_coding	hyaluronog	-

12851	protein_coding	excision re|	-
3761	protein_coding	MAS-relate	-
2740	protein_coding	dual specifi	-
9157	protein_coding	B cell leuke	-
8728	protein_coding	adrenergic	-
5309	protein_coding	endothelial	-
8990	protein_coding	MKL/myoc	-
9598	protein_coding	family with	-
5425	protein_coding	TBC1 doma	-
5756	protein_coding	zinc finger,	-
6323	protein_coding	insulin rece	-
2154	protein_coding	activating t	TF_bZIP
1257	protein_coding	transmemb	-
4098	protein_coding	interferon	-
597	processed_pseudogene	predicted g	-
6947	protein_coding	regulator o	-
7121	protein_coding	activated le	-
16010	protein_coding	tensin 1 [Sc	-
7457	protein_coding	eukaryotic	-
6369	protein_coding	cytoplasmi	-
3046	protein_coding	sphingosine	-
9181	protein_coding	OTU domai	-
5013	protein_coding	peptidyl ar	-
5812	protein_coding	chimerin 2	-
1426	protein_coding	Na+/K+ tra	-
5210	protein_coding	solute carri	-
9567	protein_coding	diacylglyce	-
4705	protein_coding	basic helix-	bHLH
2953	protein_coding	peroxisome	THR-like
5846	protein_coding	cyclin-depe	-
17303	protein_coding	neuron nav	-
5358	protein_coding	ankyrin rep	-
20676	protein_coding	HEAT repea	-
1703	protein_coding	ras homolo	-
3216	protein_coding	leucine rich	-
6095	protein_coding	solute carri	-
15938	protein_coding	bassoon [S	-
3723	protein_coding	thrombom	-
4103	protein_coding	ets variant	ETS
3837	protein_coding	Cnksr famil	-
3183	protein_coding	non imprin	-
1859	protein_coding	coagulation	-
3610	protein_coding	progressive	-
3876	protein_coding	Rho GTPase	-
2400	protein_coding	proliferatin	-
17517	protein_coding	FYVE, RhoG	-
10744	protein_coding	integrin alp	-
5444	protein_coding	potassium	-
4161	protein_coding	platelet de	-
6612	protein_coding	phosphatid	-

2288	protein_coding	polypeptid	-
10475	protein_coding	Casitas B-li	-
5747	protein_coding	diacylglyce	-
6037	protein_coding	arylsulfatas	-
12580	protein_coding	engulfmen	-
1577	protein_coding	Purkinje ce	-
8990	protein_coding	inositol 1,4	-
5454	protein_coding	integrin alp	-
5580	protein_coding	ectonucleo	-
5715	protein_coding	STEAP fami	-
9235	protein_coding	UHRF1 (ICB	-
8045	protein_coding	O-linked N-	-
6047	protein_coding	microtubul	-
5227	protein_coding	leukocyte i	-
3590	protein_coding	phospholip	-
2929	antisense	predicted g	-
2917	protein_coding	CD300 mol	-
3891	protein_coding	growth fact	-
10317	protein_coding	mannosida	-
4672	protein_coding	family with	-
4426	protein_coding	retrotransp	-
6246	protein_coding	adenylate c	-
4247	protein_coding	dipeptidase	-
7569	protein_coding	sortilin 1 [S	-
14840	protein_coding	sperm anti	-
9785	protein_coding	neuropilin	-
6556	protein_coding	ATPase, H+	-
5119	protein_coding	zinc finger	zf-C2H2
10175	protein_coding	mitogen-ac	-
3798	protein_coding	solute carri	-
10907	protein_coding	thioredoxir	-
20135	-	-	-
4682	protein_coding	dual specifi	-
5439	protein_coding	leukocyte i	-
8341	protein_coding	microtubul	-
6068	protein_coding	phosphoryl	-
18877	protein_coding	cyclin-depe	-
9000	protein_coding	solute carri	-
11182	protein_coding	tensin 3 [Sc	-
6739	protein_coding	glyceropho	-
7010	protein_coding	low density	-
16642	protein_coding	DNA methy	-
11099	protein_coding	interleukin	-
7402	protein_coding	insulin-like	-
13989	protein_coding	cyclin-depe	-
7414	protein_coding	cytoplasmi	-
9030	protein_coding	kinase inse	-
10848	protein_coding	protein tyr	-
4738	protein_coding	transmemb	-
4304	protein_coding	toll-like rec	-

6643	protein_coding	solute carri	-
4803	protein_coding	integrin alp	-
519	processed_pseudogene	predicted g	-
6453	protein_coding	WW domai	-
6056	protein_coding	acyl-CoA th	-
5679	protein_coding	solute carri	-
5946	protein_coding	ATP-bindin	-
11223	protein_coding	Yae1 doma	-
6956	protein_coding	minichrom	-
7638	protein_coding	5, 10-meth	-
2458	protein_coding	solute carri	-
10658	protein_coding	nocturnin [	-
9489	protein_coding	regulator o	-
7951	protein_coding	solute carri	-
3085	protein_coding	regulator o	-
12184	protein_coding	kinesin fam	-
5026	protein_coding	G protein-c	-
6697	protein_coding	archaelysin	-
5815	protein_coding	deiodinase,	-
6655	protein_coding	anthrax tox	-
8231	protein_coding	major facili	-
14803	protein_coding	myosin VA	-
9127	protein_coding	hect domai	-
7857	protein_coding	phospholip	-
12388	protein_coding	CAP-GLY dc	-
8364	protein_coding	folliculin in	-
8264	protein_coding	dynamin bi	-
16538	protein_coding	IQ motif co	-
4021	protein_coding	P450 (cytoc	-
6215	protein_coding	signal trans	-
3467	protein_coding	mucolipin 3	-
2617	protein_coding	plasminoge	-
3787	protein_coding	wolframin	-
12742	protein_coding	gasdermin	-
2354	protein_coding	peripheral	-
4764	protein_coding	cyclin-depe	-
5256	protein_coding	RIKEN cDN/	-
10257	protein_coding	ectonucleo	-
7808	protein_coding	EYA transcı	-
4936	protein_coding	ring finger	-
2044	protein_coding	CD9 antige	-
4363	protein_coding	Fyn proto-c	-
2884	protein_coding	molybdenu	-
5542	protein_coding	heparan su	-
10291	protein_coding	mannoside	-
5397	protein_coding	pyridoxal (p	-
10105	protein_coding	membrane	-
13017	protein_coding	kinesin fam	-
1780	lincRNA	predicted g	-
3826	protein_coding	Ras associa	-

7193	protein_coding	phosphatid	-
3258	protein_coding	cell divisior	-
6345	protein_coding	calpain 2 [S	-
4624	protein_coding	RAB7B, me	-
4871	protein_coding	inverted fo	-
6100	protein_coding	PQ loop rep	-
6256	protein_coding	antizyme ir	-
4003	protein_coding	cannabinoi	-
4231	protein_coding	mitogen-ac	-
9801	protein_coding	talin 1 [Sou	-
6936	protein_coding	mitochond	-
8415	protein_coding	lipoma HM	-
3438	protein_coding	t-complex :	-
6044	protein_coding	ATPase, H+	-
8342	protein_coding	synaptojan	-
12772	protein_coding	tectonin be	-
20771	lincRNA	nuclear par	-
4368	protein_coding	tryptophan	-
3610	protein_coding	solute carri	-
3637	protein_coding	leucine rich	-
2031	protein_coding	regulator o	-
5875	protein_coding	amyloid be	-
11833	protein_coding	tuberous sc	-
3757	protein_coding	adhesion G	-
5860	protein_coding	centromere	-
4455	protein_coding	non-catalyt	-
11271	protein_coding	Ras and Ra	-
7173	protein_coding	integrator (	-
3140	protein_coding	chromatin	-
4705	protein_coding	enolase 2, g	-
9175	protein_coding	xanthine de	-
2636	protein_coding	CCAAT/enh	C/EBP
4863	protein_coding	CLOCK inte	-
9070	processed_transcript	transcriptic	zf-C2H2
6572	protein_coding	chloride int	-
6357	protein_coding	dystroglyca	-
7326	protein_coding	ubiquitin as	-
6733	protein_coding	adenylate c	-
6031	protein_coding	DENN/MAD	-
10725	protein_coding	NPC intrace	-
9567	protein_coding	STRA6-like	-
12025	transcribed_unprocessed	protein pho	-
11601	protein_coding	IQ motif an	-
3766	protein_coding	glutamate-	-
4899	protein_coding	von Willebr	-
3627	protein_coding	regulator o	-
31486	protein_coding	R3H domai	-
9665	protein_coding	lipin 1 [Sou	-
8457	protein_coding	solute carri	-
4716	transcribed_unprocessed	G protein-c	-

5571	protein_coding	glucosamin	-
5846	protein_coding	2-oxoglutaı	-
6602	protein_coding	Rho GTPas	-
9890	protein_coding	dopey fami	-
7866	protein_coding	sorting nex	-
8566	protein_coding	myosin VII/	-
5770	protein_coding	expressed :	-
6168	protein_coding	neuroplasti	-
10463	protein_coding	inositol 1,4	-
3982	protein_coding	cannabinoi	-
3189	protein_coding	jun proto-c	TF_bZIP
5177	protein_coding	feline leuke	-
6378	protein_coding	protein kin	-
3356	protein_coding	dual specifi	-
7103	protein_coding	cytoplasmi	-
18255	protein_coding	S-adenosyl	-
3335	protein_coding	caveolin 2 |	-
12639	protein_coding	von Willebı	-
10440	protein_coding	cell adhesic	-
5968	protein_coding	pleckstrin h	-
2595	protein_coding	methyltran	-
5253	protein_coding	neutral chc	-
6458	protein_coding	MINDY lysi	-
6001	protein_coding	importin 5	-
1560	protein_coding	histone clu	-
5349	protein_coding	solute carri	-
4457	protein_coding	filamin binc	-
4073	protein_coding	solute carri	-
4709	protein_coding	glycoprotei	-
8059	protein_coding	USP6 N-ter	-
6453	protein_coding	ER degrada	-
10800	protein_coding	RAB3 GTPa	-
3407	protein_coding	zinc finger,	-
4002	protein_coding	SPRY doma	-
2246	protein_coding	transmemb	-
2638	polymorphic_pseudogen	C-type lecti	-
1648	protein_coding	secreted pl	-
2877	protein_coding	MSL compl	-
8462	protein_coding	two pore cl	-
8486	protein_coding	karyopheri	-
8225	protein_coding	suppressor	-
1880	protein_coding	sorting nex	-
9904	protein_coding	insulin-like	-
3739	protein_coding	tweety fam	-
5224	protein_coding	ArfGAP wit	-
7862	protein_coding	solute carri	-
2090	protein_coding	adenylosuc	-
4305	protein_coding	complemeı	-
6850	protein_coding	diacylglyce	-
8446	protein_coding	SLAIN moti	-

8395	protein_coding	transmemb	-
1610	protein_coding	tubulin, be	-
4593	-	-	-
2591	protein_coding	delta(4)-de	-
2842	protein_coding	selenoprot	-
4588	protein_coding	low density	-
4092	protein_coding	zinc finger	-
4191	protein_coding	RAS related	-
7990	protein_coding	ATP-bindin	-
6514	protein_coding	glycoprotei	-
6742	protein_coding	KAT8 regul	-
1751	protein_coding	heme oxyg	-
3971	protein_coding	major facili	-
2927	protein_coding	dehydroge	-
7320	protein_coding	tetratricop	-
10201	protein_coding	protein tyr	-
4808	protein_coding	synaptogyr	-
5722	protein_coding	mannosida	-
7779	protein_coding	pyruvate d	-
1144	protein_coding	mitochond	-
9046	protein_coding	NHL repeat	-
2969	protein_coding	lysosomal a	-
3871	protein_coding	polypeptid	-
5490	protein_coding	ELL associa	-
5021	protein_coding	formin-like	-
11159	protein_coding	TAO kinase	-
9775	protein_coding	acyl-Coenz	-
15942	protein_coding	erythrocyte	-
3542	protein_coding	S-adenosyl	-
8994	protein_coding	TSC22 dom	TSC22
3548	protein_coding	TatD DNase	-
7414	protein_coding	amyloid be	-
7923	protein_coding	RUN and SH	-
22493	protein_coding	calcium/ca	-
1108	TEC	RIKEN cDNA	-
8247	processed_transcript	functional i	-
5928	protein_coding	transmemb	-
15751	protein_coding	Rho guanin	-
6272	protein_coding	CD84 antig	-
11530	protein_coding	erythrocyte	-
2621	protein_coding	linker for a	-
12741	protein_coding	SPEG comp	-
4787	protein_coding	pitrilysin m	-
4475	protein_coding	5'-nucleoti	-
6700	protein_coding	B cell leuke	ZBTB
4618	protein_coding	tripartite m	-
6985	protein_coding	phosphofu	-
2570	protein_coding	compleme	-
10714	protein_coding	NIMA (nev	-
13471	protein_coding	regulatory	-

5256	protein_coding	two pore s‹	-	
7159	protein_coding	apoptosis-a	-	
4125	protein_coding	family with	-	
1378	protein_coding	mitochond	-	
6626	protein_coding	RIKEN cDNA	-	
6285	protein_coding	transportin	-	
2036	protein_coding	CD5 antige	-	
3627	protein_coding	solute carri	-	
7530	protein_coding	protein kin	-	
5014	protein_coding	V-set immu	-	
6901	protein_coding	SUMO/sen	-	
7487	protein_coding	tumor prot	-	
5935	protein_coding	NCK associa	-	
4509	protein_coding	CD180 anti	-	
6306	protein_coding	transmemb	-	
13401	protein_coding	phosphofru	-	
5205	protein_coding	aph1 homo	-	
6399	protein_coding	inositol pol	-	
5533	protein_coding	actinin, alp	-	
5140	protein_coding	Rho GTPase	-	
2548	protein_coding	growth arre	-	
7971	protein_coding	sphingosine	-	
3512	protein_coding	perilipin 2 |	-	
2820	protein_coding	sulfiredoxin	-	
3587	protein_coding	glyceronep	-	
3744	protein_coding	tec protein	-	
4666	protein_coding	minichrom	-	
5233	protein_coding	SH2 domain	-	
7077	protein_coding	calcium bin	-	
2613	protein_coding	catalase [S	-	
6127	protein_coding	actinin alph	-	
3933	protein_coding	VPS33B int	-	
6595	protein_coding	hydroxyacy	-	
4812	protein_coding	ceramide k	-	
8500	protein_coding	scavenger r	-	
3709	protein_coding	tyrosine 3-r	-	
5698	protein_coding	ATP-bindin	-	
5305	protein_coding	ATPase, H+	-	
1906	protein_coding	hexosamin	-	
6991	protein_coding	reticulon 4	-	
9791	protein_coding	sterol O-ac	-	
2702	protein_coding	apolipopro	-	
4914	protein_coding	myosin IF [	-	
6270	protein_coding	diacylglyce	-	
5849	protein_coding	cDNA sequ	-	
3896	protein_coding	tissue inhib	-	
3796	protein_coding	nuclear fac	TF_bZIP	
4170	protein_coding	mannose-6	-	
3323	protein_coding	glutamate-	-	
8212	protein_coding	nucleobind	-	

5890	protein_coding	capping prc	-
2321	protein_coding	actin relate	-
6062	protein_coding	DnaJ heat s	-
3840	protein_coding	serine inco	-
4032	protein_coding	heat shock	-
6590	protein_coding	family with	-
3807	protein_coding	transmemb	-
5200	protein_coding	OTU domai	-
7025	protein_coding	purinergic r	-
6723	protein_coding	protein tyr	-
12996	protein_coding	amyloid be	-
4048	protein_coding	myristoylat	-
2802	protein_coding	protein tyr	-
9757	protein_coding	staphyloco	-
3598	protein_coding	Max protei	bHLH
1462	protein_coding	prothymos	-
3079	protein_coding	thymocyte	-
1358	protein_coding	cathepsin S	-
3456	protein_coding	fatty acid d	-
3281	protein_coding	surfeit gen	-
1830	protein_coding	poly(rC) bir	-
3470	protein_coding	zinc finger	-
3217	protein_coding	neuralized	-
4285	protein_coding	vascular en	-
3236	protein_coding	DnaJ heat s	-
1359	protein_coding	translation	-
10921	protein_coding	calcium/ca	-
3981	protein_coding	family with	-
2841	protein_coding	unc-93 hon	-
5989	protein_coding	syndecan 3	-
4885	protein_coding	colony stin	-
1604	protein_coding	predicted g	-
4255	protein_coding	protein pho	-
4301	protein_coding	heat shock	-
5024	protein_coding	integrin alp	-
2838	protein_coding	ring finger	-
5115	protein_coding	actin relate	-
3862	protein_coding	vacuole me	-
6977	protein_coding	arginyl ami	-
4611	protein_coding	stress-asso	-
4569	protein_coding	tripartite m	-
8105	protein_coding	Rho GTPas	-
1646	protein_coding	solute carri	-
3275	protein_coding	solute carri	-
603	protein_coding	cystatin B [	-
2579	protein_coding	histocompa	-
3721	protein_coding	N-acyletha	-
3385	protein_coding	RNA exonu	-
3297	protein_coding	shisa family	-
6626	protein_coding	phospholip	-

3238	protein_coding	adhesion G	-
2845	protein_coding	arrestin, be	-
5020	protein_coding	solute carri	-
2515	protein_coding	zinc finger	-
12302	protein_coding	TRIO and F	-
4521	protein_coding	zinc finger	zf-C2H2
2547	protein_coding	syndecan 4	-
3609	protein_coding	colony stim	-
5734	protein_coding	ring finger	-
12061	protein_coding	nuclear fac	RHD
4601	processed_transcript	transmemb	-
9282	protein_coding	alanyl-tRNA	-
874	protein_coding	Fc receptor	-
2695	protein_coding	spleen focu	ETS
3740	protein_coding	tripartite m	-
4229	protein_coding	heat shock	-
1131	protein_coding	cytochrom	-
5420	protein_coding	voltage-de	-
4408	protein_coding	differential	-
1447	protein_coding	cDNA sequ	-
8842	protein_coding	ubiquitin-c	-
2420	protein_coding	ubiquitin as	-
4255	protein_coding	zinc finger	zf-C2H2
9527	protein_coding	signal trans	STAT
1234	protein_coding	proteasom	-
5033	protein_coding	AXL recept	-
6583	protein_coding	cysteinyl-tR	-
1931	protein_coding	complemen	-
3199	protein_coding	TRAF-intera	-
5186	protein_coding	acid phospl	-
1420	protein_coding	ras homolo	-
899	protein_coding	proteasom	-
3116	protein_coding	choline/eth	-
6752	protein_coding	ATP-bindin	-
4719	protein_coding	TBC1 doma	-
7026	protein_coding	poly (ADP-r	-
5992	protein_coding	solute carri	-
2423	protein_coding	calmodulin	-
3017	protein_coding	cytochrom	-
2239	protein_coding	succinate d	-
1655	protein_coding	ribosomal p	-
1599	protein_coding	C-type lecti	-
2479	protein_coding	Parkinson c	-
3377	protein_coding	idnK glucor	-
1864	protein_coding	signal sequ	-
7485	protein_coding	RAS p21 pr	-
8242	protein_coding	DEAD (Asp-	-
5307	protein_coding	lysine meth	-
5494	protein_coding	glutaredoxi	-
6096	protein_coding	TRAF family	-

3103	protein_coding	Rac family :	-
4933	protein_coding	O-sialoglyc	-
1576	protein_coding	BCL2-assoc	-
2880	protein_coding	calreticulin	-
3539	protein_coding	TNFAIP3 in	-
1626	protein_coding	high mobili	HMGI/HMGY
1965	protein_coding	gamma-glu	-
3498	protein_coding	interleukin	-
4376	protein_coding	mitogen-ac	-
1995	protein_coding	torsin fami	-
6002	protein_coding	interleukin	-
12750	protein_coding	recombinat	CSL
1529	protein_coding	expressed :	-
1474	protein_coding	heat shock	-
3745	protein_coding	protein dis	-
2576	protein_coding	myeloid dif	-
3990	protein_coding	protein kin	-
3006	protein_coding	myotubula	-
8875	protein_coding	ceramide s	Homeobox
4795	protein_coding	serine (or c	-
4937	protein_coding	elastin mic	-
5079	protein_coding	nucleotide-	-
1317	protein_coding	endothelial	-
2027	protein_coding	charged mu	-
6443	protein_coding	ATP syntha	-
5318	protein_coding	solute carri	-
2352	protein_coding	FBJ osteosa	TF_bZIP
2563	protein_coding	isochorism	-
3949	protein_coding	RIKEN cDN	-
5703	protein_coding	terminal nu	-
3884	protein_coding	prostagland	-
9300	protein_coding	sorting nex	-
5149	protein_coding	CD300 mol	-
2353	protein_coding	chromatin	-
1241	protein_coding	ribosomal p	-
545	protein_coding	cytochrom	-
4997	protein_coding	syntaxin bi	-
2618	protein_coding	ST3 beta-ga	-
2176	protein_coding	iron-sulfur	-
2181	protein_coding	ATP syntha	-
6453	protein_coding	acyl-Coenz	-
2638	protein_coding	proviral int	-
1322	protein_coding	MPV17 mit	-
5625	protein_coding	NUAK fami	-
2339	protein_coding	RAB32, me	-
641	protein_coding	ubiquinol-c	-
2031	protein_coding	protein C re	-
584	protein_coding	ubiquinol-c	-
1022	protein_coding	NADH:ubiq	-
3524	protein_coding	mesenceph	-

2405	protein_coding	profilin 1 [S	-
5288	protein_coding	ubiquitin sp	-
1088	protein_coding	complemen	-
6851	protein_coding	ST3 beta-ga	-
3050	protein_coding	peroxiredo	-
5608	protein_coding	valyl-tRNA	-
3346	protein_coding	toll-like rec	-
1484	protein_coding	ethylmalon	-
4984	protein_coding	tumor necr	-
1636	protein_coding	CDK2-asso	-
3714	protein_coding	tripartite m	-
5054	protein_coding	nicotinami	-
4134	protein_coding	FYVE, RhoG	-
3632	protein_coding	lymphocyte	-
1499	protein_coding	COP9 signa	-
2459	protein_coding	docking pro	-
2095	protein_coding	proline-rich	-
1038	protein_coding	LSM4 hom	-
1214	protein_coding	cytochrom	-
3712	protein_coding	interleukin	-
651	transcribed_processed_p	predicted p	-
1218	protein_coding	late endoso	-
3071	protein_coding	proteasom	-
2887	protein_coding	scavenger r	-
2346	protein_coding	drebrin-like	-
3746	protein_coding	nectin cell a	-
7247	protein_coding	ER membra	-
3526	protein_coding	ribosomal p	-
2169	protein_coding	nuclear fac	-
7627	protein_coding	major vault	-
14076	protein_coding	DDHD dom	-
2377	protein_coding	anaphase p	-
6097	protein_coding	terminal nu	-
1441	protein_coding	cathepsin Z	-
2893	protein_coding	flotillin 1 [S	-
655	protein_coding	interferon i	-
876	protein_coding	ubiquinol-c	-
1908	protein_coding	hydroxyacy	-
3943	protein_coding	p21 (RAC1)	-
766	-	-	-
3542	protein_coding	SEM1, 26S	-
419	protein_coding	NADH:ubiq	-
438	processed_pseudogene	ribosomal p	-
5018	protein_coding	nuclear fac	-
4362	protein_coding	transmemb	-
4236	protein_coding	angiotensir	-
8172	protein_coding	phosphotyr	-
1051	protein_coding	thioredoxir	-
663	protein_coding	FK506 bind	-
1781	protein_coding	ribosomal p	-

4117	protein_coding	serine/thre	-
1831	protein_coding	ubiquinol-c	-
883	protein_coding	transmemb	-
7154	protein_coding	a disintegri	-
6057	protein_coding	potassium	-
6021	protein_coding	solute carri	-
542	protein_coding	ribosomal p	-
3288	protein_coding	mucolipin 2	-
1154	protein_coding	Epstein-Bar	-
653	processed_pseudogene	ribosomal p	-
3900	protein_coding	branched c	-
1570	protein_coding	ninjurin 1 [	-
4344	protein_coding	glutathione	-
1847	protein_coding	Kruppel-lik	zf-C2H2
10916	protein_coding	TNFAIP3 in	-
8078	protein_coding	fibrosin-like	-
4459	protein_coding	lymphocyte	-
898	protein_coding	NADH:ubiq	-
1991	-	-	-
1342	processed_pseudogene	predicted g	-
691	protein_coding	ribosomal p	-
6549	protein_coding	glycerol ph	-
1271	protein_coding	Sec61 beta	-
7277	protein_coding	colony stim	-
4084	protein_coding	sulfide quir	-
8118	protein_coding	adenosine	-
7315	protein_coding	methylene	-
3622	protein_coding	CD274 anti	-
869	protein_coding	predicted p	-
1637	protein_coding	ribonucleas	-
3334	protein_coding	prostagland	-
1454	protein_coding	B9 protein	-
3495	protein_coding	hexamethy	-
6906	protein_coding	embigin [Sc	-
1051	protein_coding	translocato	-
5780	protein_coding	E26 avian l	ETS
3699	protein_coding	poly (ADP-r	-
1683	protein_coding	hydroxypro	-
6439	protein_coding	sorting nex	-
1217	protein_coding	ATP syntha	-
2177	protein_coding	selenophos	-
3497	protein_coding	TRAF-intera	-
8307	protein_coding	nuclear tra	zf-NF-X1
5756	protein_coding	TRAF3 inte	-
5936	protein_coding	COMM dor	-
1318	protein_coding	mitochond	-
2464	protein_coding	SEC61, gam	-
2278	protein_coding	H2.0-like h	Homeobox
792	processed_pseudogene	ribosomal p	-
4529	protein_coding	post-GPI at	-

4655	protein_coding	zinc finger,	zf-MIZ
1756	protein_coding	ATP syntha	-
2743	protein_coding	B cell trans	-
1039	protein_coding	cytochrom	-
5607	protein_coding	Moloney le	-
3329	lincRNA	predicted g	-
5253	protein_coding	BCL2 modif	-
2311	protein_coding	vesicle-asso	-
1522	protein_coding	keratinocyt	-
1464	protein_coding	ribosomal p	-
1707	protein_coding	Rho, GDP d	-
5079	-	-	-
3781	protein_coding	transporter	-
25021	protein_coding	cAMP resp	TF_bZIP
2789	protein_coding	protein-tyr	-
3328	protein_coding	Sp140 nucl	SAND
9635	protein_coding	sideroflexir	-
1258	protein_coding	methionine	-
2858	protein_coding	SH3-domai	-
1178	protein_coding	sin3 associa	-
1056	protein_coding	ABRA C-ter	-
4903	protein_coding	serum/gluc	-
2770	protein_coding	MIT, micro	-
1485	protein_coding	histocompa	-
9351	protein_coding	protein tyr	-
1736	protein_coding	complemen	-
3356	protein_coding	ring finger	-
1587	protein_coding	cytochrom	-
4959	protein_coding	coiled-coil	-
2020	protein_coding	neutrophil	-
3462	protein_coding	phospholip	-
2302	protein_coding	transmemb	-
2101	protein_coding	cytochrom	-
2301	protein_coding	jun B proto	TF_bZIP
3663	protein_coding	neutrophil	-
541	protein_coding	antioxidant	-
810	protein_coding	ribosomal p	-
6251	protein_coding	GTP cycloh	-
6675	protein_coding	phosphofru	-
5896	protein_coding	hypoxia inc	-
3045	protein_coding	chemokine	-
3110	protein_coding	molybdenu	-
3058	protein_coding	Fas death c	-
1251	protein_coding	NADH:ubiq	-
4520	protein_coding	dihydropyr	-
2140	protein_coding	adenylate k	-
4250	protein_coding	5'-nucleoti	-
1877	protein_coding	guanine nu	-
1735	protein_coding	membrane	-
7451	protein_coding	myeloid/ly	-

14983	protein_coding	protein pho	-
1859	protein_coding	mevalonate	-
4414	protein_coding	acetyl-Coer	-
3340	protein_coding	cryptochro	-
5886	protein_coding	lipase, horr	-
2872	protein_coding	ring finger	-
1438	protein_coding	histocompa	-
4690	processed_transcript	predicted g	-
4280	protein_coding	solute carri	-
3050	protein_coding	tsukushi, sr	-
5571	protein_coding	promyelocy	-
2124	protein_coding	acyl-CoA th	-
9209	protein_coding	Ras associa	-
2525	protein_coding	hematopoi	-
3553	protein_coding	olfactomec	-
860	protein_coding	beta-2 micr	-
2905	protein_coding	phospholip	-
4914	protein_coding	follistatin-li	-
4935	protein_coding	SAM doma	-
3077	protein_coding	mitogen-ac	-
4486	protein_coding	TRAF type	-
4810	protein_coding	lysyl oxidas	-
5781	protein_coding	tripartite m	-
2377	protein_coding	gasdermin	-
4275	protein_coding	poly (ADP-r	-
1327	protein_coding	C-type lecti	-
853	processed_transcript	small nucle	-
5464	protein_coding	uridine-cyt	-
243	protein_coding		-
2069	protein_coding	G-protein c	-
1121	protein_coding	related RAS	-
2466	rRNA	18s RNA, re	-
4261	protein_coding	retinoic aci	THR-like
8918	protein_coding	terminal nu	-
4209	protein_coding	syntaxin 11	-
2783	protein_coding	coronin, ac	-
5427	protein_coding	sterile alph	-
1065	protein_coding	small vasoh	-
3041	protein_coding	MAP kinase	-
693	protein_coding	ribosomal p	-
11294	protein_coding	RIKEN cDNA	-
3747	protein_coding	NAD kinase	-
41155	protein_coding	dystonin [S	-
4917	protein_coding	triggering r	-
11059	protein_coding	purinergic r	-
2929	processed_transcript	zinc finger,	-
9150	protein_coding	SAM doma	-
955	Mt_rRNA	mitochond	-
3818	protein_coding	F-box and W	-
3045	protein_coding	DNA-dama	-

3005	protein_coding	CD82 antig	-
616	protein_coding	ATP syntha	-
4308	protein_coding	leucine rich	-
1316	protein_coding	lysozyme 2	-
1109	protein_coding	mitochond	-
2660	protein_coding	napsin A as	-
10425	protein_coding	gamma-sec	-
12189	protein_coding	NEDD4 bin	-
3707	protein_coding	LIM domain	-
3080	protein_coding	T cell activa	-
6692	protein_coding	ubiquitin-li	-
1027	protein_coding	family with	-
5594	protein_coding	transcriptic	bHLH
2580	protein_coding	opioid grov	-
8882	protein_coding	poly (ADP-r	-
7143	protein_coding	sialic acid b	-
2434	bidirectional_promoter_	RIKEN cDNA	-
2030	protein_coding	acid phospl	-
9018	protein_coding	inhibitor of	-
2731	protein_coding	coagulation	-
3892	protein_coding	torsin fami	-
3090	protein_coding	parvin, gan	-
2333	protein_coding	homocyste	-
4371	protein_coding	Rous sarco	-
9306	protein_coding	poly (ADP-r	-
836	protein_coding	membrane	-
2336	protein_coding	glyceropho	-
3026	protein_coding	interleukin	-
2926	protein_coding	C-type lecti	-
2768	antisense	predicted g	-
1619	protein_coding	parathymo	-
5082	protein_coding	growth fact	-
3622	protein_coding	phosphoino	-
9285	protein_coding	agrin [Sour	-
1469	protein_coding	proteasom	-
2092	protein_coding	hemopoiet	-
4637	protein_coding	pyruvate ca	-
8189	protein_coding	zinc finger,	-
12540	protein_coding	AT rich inte	ARID
798	processed_pseudogene	ribosomal p	-
1775	protein_coding	peroxisoma	-
354	processed_pseudogene	predicted g	-
3094	protein_coding	syntrophin,	-
2747	protein_coding	sperm flage	-
5774	protein_coding	coiled-coil	-
1153	bidirectional_promoter_	NADH:ubiq	-
3280	protein_coding	RELT tumor	-
2334	protein_coding	Fc receptor	-
4529	protein_coding	calcium cha	-
5113	protein_coding	eukaryotic	-

1444	processed_pseudogene	predicted p	-
4473	protein_coding	neurexoph	-
2817	protein_coding	glia matura	-
3414	protein_coding	histocompa	-
5386	protein_coding	interferon	IRF
837	protein_coding	protease (p	-
2033	protein_coding	solute carri	-
5269	protein_coding	centrosom	-
5808	protein_coding	cytochrom	-
2175	protein_coding	immunoglo	-
2303	protein_coding	mixed linea	-
3995	protein_coding	poly (ADP-r	-
6135	protein_coding	guanine de	-
3676	protein_coding	interleukin	-
5276	protein_coding	B cell trans	-
10163	protein_coding	acyl-CoA sy	-
1639	protein_coding	tumor necr	-
736	protein_coding	selenoprot	-
3494	protein_coding	nectin cell	-
415	protein_coding	leukocyte s	-
5142	protein_coding	cysteinyl le	-
1504	protein_coding	CCAAT/enh	C/EBP
3682	protein_coding	signal-regu	-
2209	protein_coding	proteasom	-
2592	protein_coding	tripartite m	-
11342	protein_coding	growth arre	-
4409	protein_coding	zinc finger,	-
2608	protein_coding	histocompa	-
3838	protein_coding	G protein-c	-
1395	protein_coding	proline-ser	-
11529	protein_coding	helicase wi	-
13922	protein_coding	solute carri	-
11788	protein_coding	RIKEN cDNA	-
3296	protein_coding	TAP binding	-
3487	protein_coding	methylenet	-
3710	protein_coding	caspase 4,	-
8301	protein_coding	polycystic k	-
1942	protein_coding	eva-1 homo	-
823	protein_coding	fatty acid b	-
1859	protein_coding	sodium cha	-
3263	protein_coding	aldolase C,	-
2589	protein_coding	Fc receptor	-
4039	protein_coding	arrestin do	-
5335	protein_coding	filamin A in	-
2026	protein_coding	nuclear fac	TF_bZIP
9102	protein_coding	spermatog	-
3330	protein_coding	CD101 anti	-
8493	protein_coding	NLR family,	-
5725	protein_coding	deltex 3-lik	-
8165	protein_coding	interferon i	-

2292	protein_coding	receptor-in	-
6159	protein_coding	ral guanine	-
2001	protein_coding	N-myc (anc	-
1577	protein_coding	Ras-relatec	-
12459	protein_coding	transient re	-
2594	protein_coding	v-maf musc	TF_bZIP
2862	protein_coding	intraflagell	-
2528	protein_coding	purinergic r	-
6781	protein_coding	Sp110 nucl	SAND
3661	protein_coding	CD86 antig	-
6619	protein_coding	collagen, ty	-
3021	protein_coding	chemokine	-
3841	protein_coding	Spi-C transc	ETS
2352	protein_coding	TAP binding	-
5790	protein_coding	Fc receptor	-
1575	protein_coding	coiled-coil-	-
1043	protein_coding	ribosomal p	-
4446	protein_coding	interleukin	-
7637	protein_coding	suppressior	-
1558	protein_coding	C-type lecti	-
9603	protein_coding	dystrobrev	-
1569	protein_coding	NADH:ubiq	-
428	processed_pseudogene	predicted g	-
6648	protein_coding	prostaglanc	-
2387	protein_coding	interleukin	-
9292	protein_coding	hect domai	-
2736	protein_coding	proteasom	-
3361	protein_coding	carcinoemb	-
4091	protein_coding	schlafen 8 |	-
5235	protein_coding	RIKEN cDNA	-
4111	protein_coding	tripartite m	-
4674	protein_coding	pre B cell le	-
3885	protein_coding	avian reticu	RHD
1392	protein_coding	RAB20, me	-
3659	protein_coding	proteasom	-
2508	protein_coding	major facili	-
2889	protein_coding	histocompa	-
441	processed_pseudogene	predicted g	-
2556	unprocessed_pseudogen	interferon a	-
1664	protein_coding	schlafen 2 |	-
2050	protein_coding	membrane	-
2224	protein_coding	dynein, axc	-
3289	protein_coding	nuclear fac	RHD
1209	protein_coding	insulin-like	-
1187	protein_coding	CD52 antig	-
3862	protein_coding	predicted g	-
1406	protein_coding	basic leucir	TF_bZIP
6192	protein_coding	androglobi	-
1354	protein_coding	CD302 anti	-
5236	processed_transcript	expressed s	-

1705	protein_coding	proteasom	-
5014	protein_coding	nucleotide-	-
9005	protein_coding	GTPase, ve	-
13066	protein_coding	inositol pol	-
10973	protein_coding	neural prec	-
6569	protein_coding	integrin alp	-
3561	protein_coding	interferon a	-
2633	protein_coding	phospholip	-
1438	protein_coding	tumor necr	-
2770	protein_coding	integrin be	-
5476	protein_coding	transporter	-
16888	protein_coding	ring finger	-
7556	protein_coding	nuclear ant	SAND
2137	protein_coding	lectin, gala	-
7159	protein_coding	G protein-c	-
1165	processed_pseudogene	predicted g	-
1610	protein_coding	nuclear fac	-
2148	protein_coding	Jun dimeriz	TF_bZIP
3856	protein_coding	chondroitir	-
1992	polymorphic_pseudogen	2'-5' oligoa	-
5974	protein_coding	G protein-c	-
11963	protein_coding	collagen, ty	-
3781	protein_coding	deoxycytid	-
498	processed_pseudogene	predicted p	-
4057	protein_coding	NLR family,	-
7636	protein_coding	pleckstrin a	-
3408	protein_coding	chemokine	-
6077	protein_coding	ORAI calciu	-
309	processed_pseudogene	predicted g	-
5507	-	-	-
10483	protein_coding	acyl-CoA sy	-
5808	protein_coding	cathepsin C	-
3371	protein_coding	matrix met	-
2908	protein_coding	suppressor	-
2321	protein_coding	epithelial s	-
3443	protein_coding	guanylate-l	-
1996	protein_coding	ubiquitin-c	-
3628	protein_coding	myeloid nu	-
6326	protein_coding	schlafen 5		-
2715	protein_coding	G protein-c	-
9275	protein_coding	predicted g	-
1591	polymorphic_pseudogen	histocompa	-
2088	protein_coding	CD300E mo	-
1040	protein_coding	complemer	-
11685	protein_coding	fibronectin	-
6706	protein_coding	interferon a	-
2847	protein_coding	lectin, gala	-
5268	protein_coding	protection	-
480	processed_pseudogene	predicted g	-
2314	protein_coding	elongin BC	-

3840	protein_coding	C-type lecti	-
171	processed_pseudogene	predicted p	-
1641	protein_coding	histocompa	-
4872	protein_coding	poly (ADP-r	-
2879	protein_coding	HtrA serine	-
504	protein_coding	predicted g	-
5009	protein_coding	baculoviral	-
3524	TEC	predicted g	-
5926	protein_coding	signal trans	STAT
6754	protein_coding	carbohydra	-
9454	protein_coding	apolipopro	-
1410	protein_coding	CD74 antig	-
12041	protein_coding	caldesmon	-
3251	protein_coding	Mediterrar	-
5293	protein_coding	coenzyme (	-
3079	protein_coding	SLAM famil	-
3045	protein_coding	nuclear fac	-
4498	protein_coding	PHD finger	-
3110	protein_coding	C-type lecti	-
3758	protein_coding	tripartite m	-
4926	protein_coding	forkhead b	Fork
2596	protein_coding	histocompa	-
3317	protein_coding	bone marro	-
3690	protein_coding	immunity-r	-
2022	protein_coding	interferon g	-
1727	protein_coding	interferon-	-
3705	protein_coding	EH-domain	-
4572	protein_coding	predicted g	-
1138	protein_coding	chemokine	-
2513	lincRNA	RIKEN cDNA	-
3863	protein_coding	FGR proto-	-
1974	protein_coding	interleukin	-
2139	protein_coding	peroxiredo	-
1764	protein_coding	proteasom	-
1845	protein_coding	adenosine	-
1930	protein_coding	hydroxycar	-
1532	protein_coding	allograft in	-
3809	protein_coding	syndecan 1	-
6905	protein_coding	lymphocyte	-
1572	protein_coding	CD14 antig	-
4266	protein_coding	endothelin	-
4101	protein_coding	hepsin [Sou	-
3876	protein_coding	serine (or c	-
1508	protein_coding	fatty acid d	-
1874	protein_coding	histocompa	-
8474	protein_coding	solute carri	-
3979	protein_coding	purine-nuc	-
3010	protein_coding	compleme	-
3976	protein_coding	interferon r	IRF
8684	protein_coding	DEAD (Asp-	-

15228	protein_coding	collagen, ty	-
4585	protein_coding	reticulon 2	-
5281	protein_coding	colony stim	-
4799	protein_coding	solute carri	-
7067	protein_coding	sperm acro	-
1555	lincRNA	predicted g	-
4432	protein_coding	Rho family	-
4197	protein_coding	frizzled clas	-
7641	protein_coding	translation	-
4535	protein_coding	phosphatas	-
813	processed_pseudogene	predicted g	-
5810	protein_coding	immunity-r	-
2522	protein_coding	basic leucin	TF_bZIP
2617	protein_coding	vasodilator	-
4980	protein_coding	reticulon 1	-
9535	protein_coding	class II tran	-
2981	protein_coding	complemen	-
5407	protein_coding	potassium	-
1509	lincRNA	predicted g	-
903	protein_coding	bone marro	-
1420	protein_coding	C-type lecti	-
1337	protein_coding	globoside a	-
1898	protein_coding	killer cell le	-
3444	protein_coding	caveolin 1,	-
2182	protein_coding	histocompa	-
7293	unprocessed_pseudogen	predicted g	-
2475	protein_coding	B cell leuke	-
3530	protein_coding	LIM domain	-
2861	protein_coding	connective	-
6556	protein_coding	RNA bindin	-
3388	protein_coding	lysophosph	-
3199	protein_coding	src homolo	-
3788	protein_coding	fibrinogen-	-
5695	protein_coding	maturin, ne	-
1785	protein_coding	interferon a	-
5406	protein_coding	sema doma	-
4485	protein_coding	membrane	-
2073	protein_coding	ADP-ribosy	-
4451	protein_coding	2'-5' oligoa	-
9441	protein_coding	aryl-hydroc	bHLH
10832	protein_coding	SLAM famil	-
7772	protein_coding	tenascin C	-
709	processed_pseudogene	predicted g	-
7050	protein_coding	DEAD (Asp-	-
801	protein_coding	chemokine	-
9409	protein_coding	cyclin D2 [S	-
4767	protein_coding	family with	-
1184	protein_coding	PHD finger	-
1885	protein_coding	2'-5' oligoa	-
4853	protein_coding	CD55 mole	-

3746	protein_coding	CCAAT/enh	-
7105	protein_coding	signal trans	STAT
3272	protein_coding	DEXH (Asp-	-
2667	protein_coding	ribonucleas	-
4058	protein_coding	AE binding	-
1750	protein_coding	phospholip	-
10560	protein_coding	phosphodie	-
1184	protein_coding	histocompa	-
7330	protein_coding	acid phospl	-
9917	protein_coding	laminin B1	-
381	protein_coding	predicted g	-
1994	-	PF08333:Pr	-
3964	protein_coding	schlafen 4 |	-
7527	protein_coding	tripartite m	-
1491	protein_coding	CDC42 effe	-
4681	protein_coding	sialophorin	-
1131	protein_coding	immediate	-
3788	protein_coding	secreted ac	-
2019	protein_coding	cysteine ric	-
2320	protein_coding	GLI pathog	-
1957	protein_coding	paired imm	-
4591	protein_coding	CXADR-like	-
1860	protein_coding	interferon-	-
1577	protein_coding	RAB38, me	-
6099	protein_coding	biglycan [Sc	-
3014	protein_coding	toll-like rec	-
7069	protein_coding	2'-5' oligoa	-
4689	protein_coding	XIAP associ	-
2514	protein_coding	nuclear prc	-
9566	protein_coding	ceruloplasr	-
3406	protein_coding	CD40 antig	-
4560	protein_coding	ectonucleo	-
4620	protein_coding	icos ligand	-
4380	protein_coding	collagen, ty	-
5960	protein_coding	matrix met	-
8819	protein_coding	TNF recept	-
8702	protein_coding	periostin, c	-
6943	protein_coding	nidogen 1 [	-
10008	protein_coding	superoxide	-
1637	protein_coding	histocompa	-
1781	protein_coding	actin, alpha	-
9491	protein_coding	sushi, nidog	-
1516	protein_coding	lymphotoxi	-
3234	protein_coding	Rho guanin	-
888	protein_coding	tissue inhib	-
6581	protein_coding	attractin lik	-
4360	protein_coding	intercellula	-
3931	protein_coding	prostaglanc	-
2371	protein_coding	transmemb	-
5925	protein_coding	acyloxyacyl	-

2443	protein_coding	serine (or c	-
1216	protein_coding	protease, s	-
3542	protein_coding	ankyrin rep	-
2340	protein_coding	2'-5' oligoa	-
4724	protein_coding	kynurenine	-
532	antisense	predicted g	-
3324	protein_coding	interleukin	-
3106	protein_coding	solute carri	-
15655	protein_coding	FAT atypica	-
9591	protein_coding	collagen, ty	-
678	protein_coding	interferon i	-
845	unprocessed_pseudogen	predicted g	-
905	protein_coding	interferon i	-
6073	protein_coding	thrombosp	-
1094	protein_coding	calcium ho	-
6674	protein_coding	mannose re	-
3547	protein_coding	interferon a	-
4005	protein_coding	FXYD doma	-
2085	protein_coding	histocompa	-
3589	antisense	novel trans	-
3810	protein_coding	transmemb	-
1801	protein_coding	Fas (TNF re	-
12128	protein_coding	fibrillin 1 [S	-
2006	protein_coding	sphingomy	-
4163	protein_coding	cytidine mo	-
1587	protein_coding	purine-nuc	-
3539	protein_coding	matrix met	-
3297	protein_coding	lysyl oxidas	-
2935	protein_coding	FK506 bind	-
9537	protein_coding	anthrax tox	-
1498	protein_coding	G protein-c	-
14462	protein_coding	versican [So	-
2131	protein_coding	ephrin A2 [	-
2060	protein_coding	apolipopro	-
3062	protein_coding	adenosine	-
6370	protein_coding	fibulin 2 [So	-
8326	protein_coding	collagen, ty	-
4335	protein_coding	radical S-ac	-
6374	protein_coding	2'-5' oligoa	-
3771	protein_coding	transformi	-
2325	protein_coding	chemokine	-
10257	protein_coding	gap junctio	-
5128	protein_coding	cytochrom	-
6760	protein_coding	chemokine	-
1240	processed_pseudogene	predicted g	-
1716	protein_coding	tripartite m	-
986	protein_coding	cystatin F (	-
2289	lincRNA	predicted g	-
2904	protein_coding	solute carri	-
5274	protein_coding	membrane	-

3614	polymorphic_pseudogen	MX dynam	-
2680	protein_coding	carbohydra	-
3297	protein_coding	RIKEN cDN/	-
6739	protein_coding	coagulatior	-
5215	protein_coding	solute carri	-
4010	protein_coding	calcium cha	-
5838	protein_coding	decorin [Sc	-
5614	protein_coding	guanylate k	-
819	protein_coding	B cell leuke	-
1758	protein_coding	TBC1 doma	-
4265	protein_coding	histocompa	-
9089	protein_coding	growth fact	-
2473	protein_coding	interferon g	-
1274	processed_pseudogene	predicted g	-
3265	protein_coding	guanylate k	-
1756	processed_pseudogene	novel C2H2	-
1551	protein_coding	histocompa	-
5266	protein_coding	tumor necr	-
1354	protein_coding	PHD finger	-
5478	protein_coding	platelet de	-
8490	protein_coding	collagen, ty	-
3890	protein_coding	CD38 antig	-
1632	protein_coding	ubiquitin ca	-
1811	protein_coding	vanin 3 [So	-
1849	protein_coding	sialic acid b	-
2068	protein_coding	chemokine	-
463	protein_coding	interferon,	-
4105	protein_coding	interleukin	-
2486	protein_coding	3-phospho	-
1005	protein_coding	calpain, sm	-
4177	protein_coding	interferon a	-
2131	protein_coding	receptor tr	-
1957	protein_coding	paired imm	-
7003	protein_coding	thrombosp	-
6809	protein_coding	collagen, ty	-
1257	polymorphic_pseudogen	ADP-ribosy	-
3012	protein_coding	fascin actin	-
2790	protein_coding	ubiquitin sp	-
12426	protein_coding	collagen, ty	-
756	protein_coding	ISG15 ubiq	-
4754	protein_coding	shisa family	-
5143	protein_coding	complemer	-
3784	protein_coding	lymphocyte	-
4580	protein_coding	prostagland	-
4537	protein_coding	olfactory re	-
3052	protein_coding	solute carri	-
4255	protein_coding	inhibin beta	-
1494	protein_coding	mast cell e:	-
3211	protein_coding	solute carri	-
1875	protein_coding	chemokine	-

5954	protein_coding	guanylate b	-
6419	protein_coding	cytokine in	-
770	lincRNA	expressed s	-
3529	protein_coding	Z-DNA binc	-
2414	protein_coding	argininosuc	-
1428	protein_coding	arginase ty	-
1180	protein_coding	secretory le	-
6563	protein_coding	collagen, ty	-
3785	protein_coding	MX dynami	-
1864	protein_coding	schlafen 1		-
2608	protein_coding	2'-5' oligoa	-
3776	protein_coding	interferon r	IRF
3090	protein_coding	paired imm	-
6509	protein_coding	CD69 antig	-
1725	protein_coding	G protein-c	-
9336	protein_coding	a disintegri	-
3927	protein_coding	interferon-	-
5508	protein_coding	spinster ho	-
4505	protein_coding	chemokine	-
2359	protein_coding	serine (or c	-
2645	protein_coding	predicted g	-
1595	protein_coding	cDNA sequ	-
3498	protein_coding	chemokine	-
2066	protein_coding	T cell-intera	-
6129	protein_coding	interferon-	-
4790	protein_coding	toll-like rec	-
1605	protein_coding	MARCKS-lik	-
2000	protein_coding	interferon-	-
12232	protein_coding	cell migrati	-
2656	protein_coding	interferon-	-
9816	protein_coding	collagen, ty	-
2891	protein_coding	suppressor	-
1745	protein_coding	interferon-	-
2560	protein_coding	CD248 anti	-
2516	protein_coding	guanylate b	-
2938	protein_coding	T cell speci	-
3054	protein_coding	interferon a	-
1751	protein_coding	membrane	-
2275	protein_coding	interferon i	-
3016	protein_coding	chemokine	-
4965	protein_coding	C-type lecti	-
1967	protein_coding	interleukin	-
6637	protein_coding	guanylate b	-
4530	processed_pseudogene	predicted g	-
5258	protein_coding	interferon a	-
4556	protein_coding	serine prot	-
5600	protein_coding	RAS guanyl	-
6840	protein_coding	interferon a	-
2961	protein_coding	growth arre	-
4038	protein_coding	v-myc aviar	bHLH

4865	protein_coding	fibronectin	-
1606	protein_coding	haptoglobi	-
2623	protein_coding	lipocalin 2 |	-
7833	protein_coding	collagen, ty	-
3435	protein_coding	sushi doma	-
1821	protein_coding	apolipopro	-
2234	protein_coding	protein pho	-
2290	protein_coding	serine (or c	-
2740	protein_coding	lymphocyte	-
1296	protein_coding	formyl pep	-
1925	protein_coding	macrophag	-
7494	protein_coding	guanylate b	-
960	protein_coding	chemokine	-
2252	protein_coding	chemokine	-
1324	protein_coding	formyl pep	-
4289	protein_coding	lysophosph	-
1205	protein_coding	lymphocyte	-
346	antisense	predicted g	-
1907	protein_coding	interleukin	-
3491	protein_coding	adhesion G	-
2623	protein_coding	aconitate d	-
2682	protein_coding	pentraxin r	-
1172	protein_coding	cyclin depe	-
2105	protein_coding	serine (or c	-
1156	protein_coding	lymphocyte	-
2345	protein_coding	calcium cha	-
3121	protein_coding	interferon i	-
1216	processed_pseudogene	predicted g	-
3400	protein_coding	ladinin [Sou	-
2825	protein_coding	complemen	-
2606	protein_coding	serum amy	-
