# Supplementary material for: Prolactin-Releasing Peptide Differentially Regulates Gene Transcriptomic Profiles in Mouse Bone Marrow-Derived Macrophages
Source: Int J Mol Sci. 2021 Apr 24;22(9):4456. doi: 10.3390/ijms22094456 (PMC8123224; doi:10.3390/ijms22094456)
Supplement: Supplementary file 1 [file ijms-22-04456-s001.zip › Supplementary File 2_Figure S1 Information of the FACS and RNA-seq detection_R3.pdf]

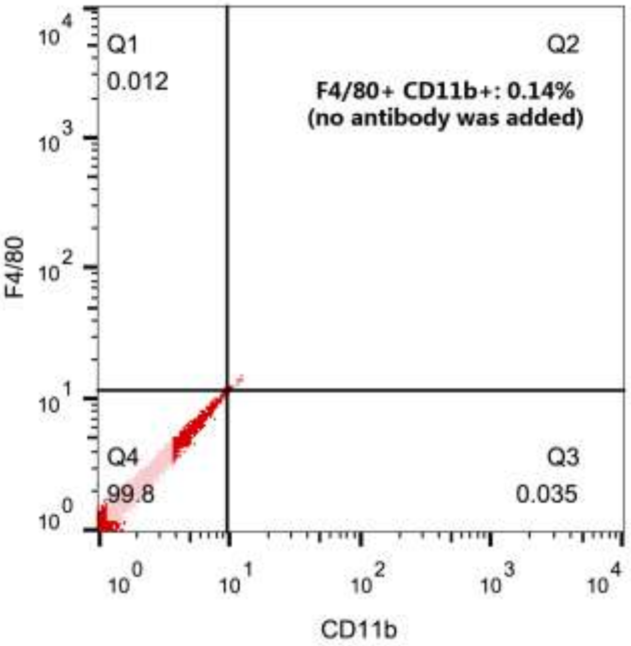
Q1
0.012
Q2
F4/80+ CD11b+: 0.14%
(no antibody was added)
Q4
99.8
Q3
0.035
F4/80
CD11b

(A)

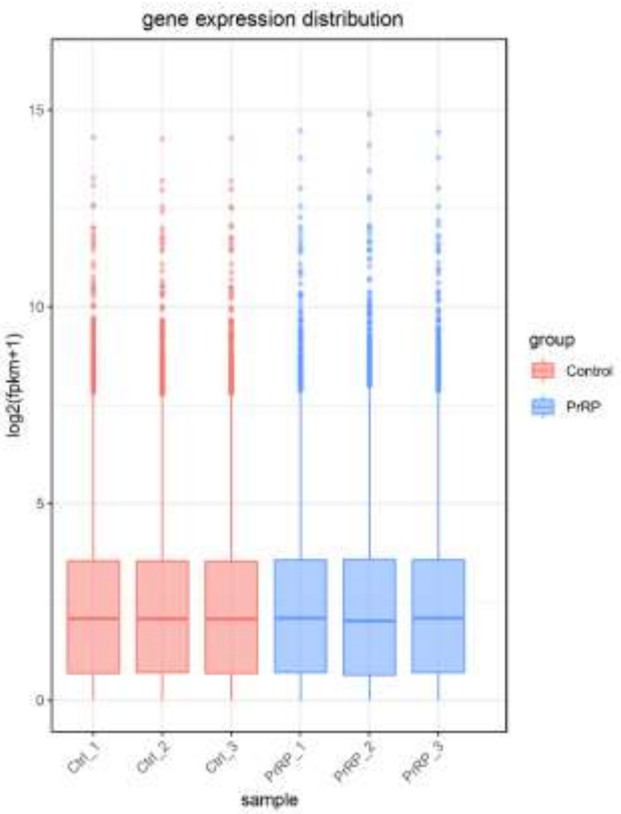
gene expression distribution
log2(fpkm+1)
group
Control
PrRP
Ctrl_1
Ctrl_2
Ctrl_3
PrRP_1
PrRP_2
PrRP_3
sample

(B)

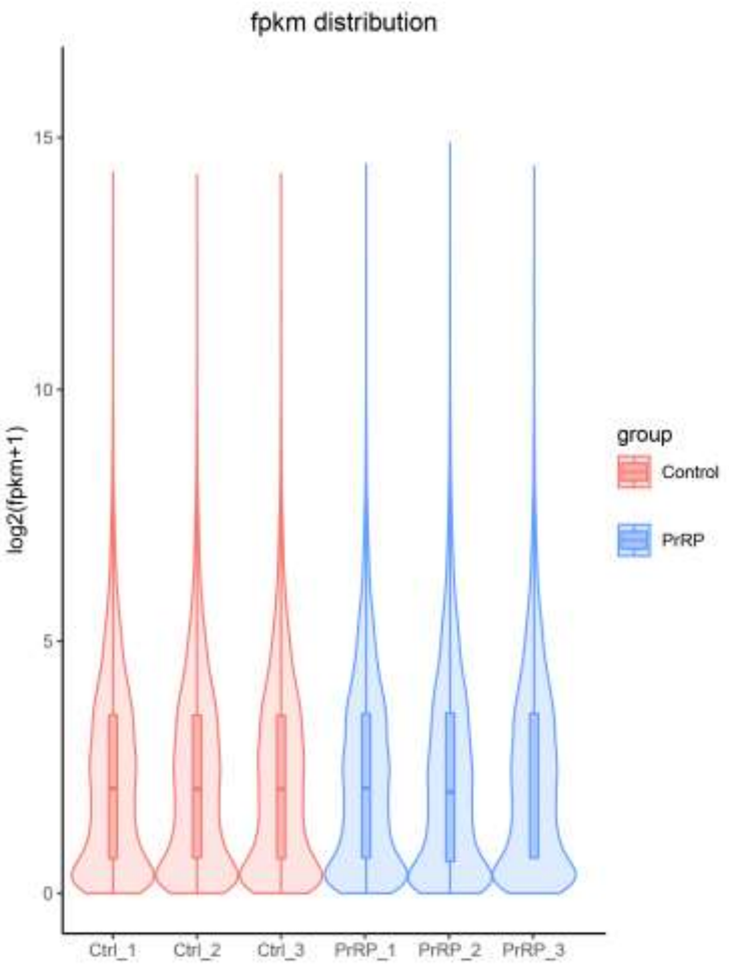
fpkm distribution
log2(fpkm+1)
15
10
5
0
Ctrl_1
Ctrl_2
Ctrl_3
PrRP_1
PrRP_2
PrRP_3
group
Control
PrRP

(C)

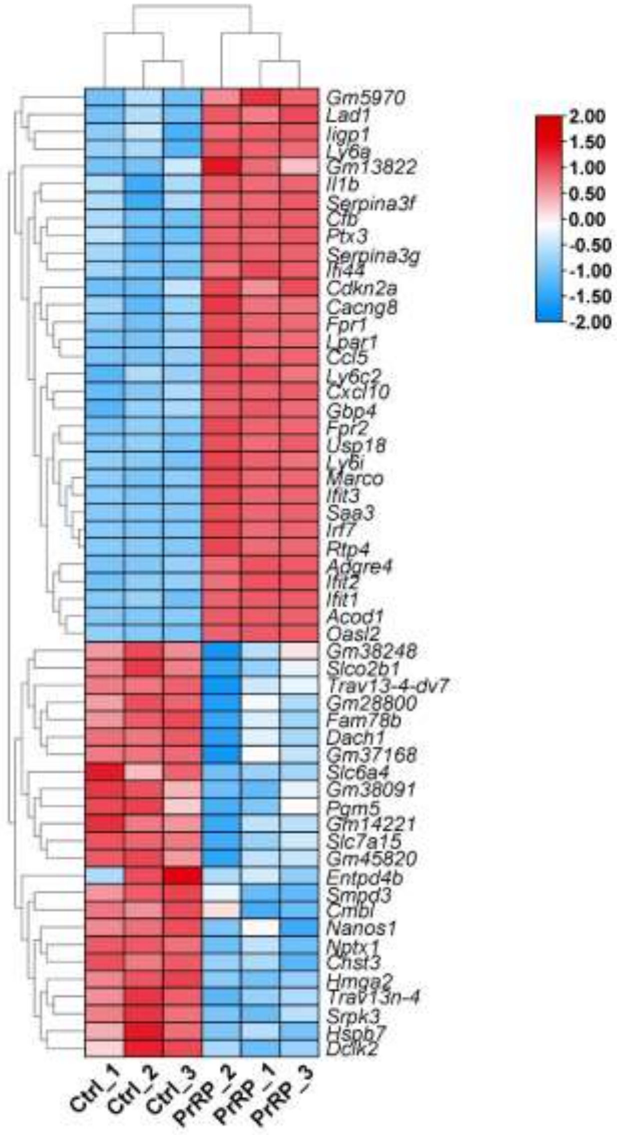

(D)

Figure S1. Information of the FACS and RNA-seq detection. (A) BMDMs were analyzed by the flow cytometer with FITC-conjugated anti-CD11b and PE-conjugated anti-F4/80. For the control group, no antibody was added to the cell culture, and the double-double positive rate of cells was 0.14%. (B) The boxplot of gene expression distribution. (C) Violin plot. (D) Heat map of the 24 up-regulated DEGs, 24 down-regulated DEGs, and 8 hub genes, as the ranking was based on the absolute value of the fold of change in descending order. The red indicated the up-regulated genes and blue represented down-regulated genes.
