# Supplementary material for: Prolactin-Releasing Peptide Differentially Regulates Gene Transcriptomic Profiles in Mouse Bone Marrow-Derived Macrophages
Source: Int J Mol Sci. 2021 Apr 24;22(9):4456. doi: 10.3390/ijms22094456 (PMC8123224; doi:10.3390/ijms22094456)
Supplement: Supplementary file 1 [file ijms-22-04456-s001.zip › Supplementary File 3_Table S2 KEGG pathway analysis kegg.pdf]

Table S2. KEGG pathway analysis of DEGs

	Pathway ID	Name	Gene count	PValue	%	Genes
1	mmu05168	Herpes simplex virus 1 infection-Mus musculus (mouse)	68	0.000695808	3.03	*C3, Irf7, Ccl5, Tlr2, Pilra, Ddx58, Il1b, Oas3, Oas2, H2-Ab1, Socs3, Stat1, Oas1g, Oas1a, H2-Eb1, Birc3, H2-Q7, Stat2, H2-Q6, H2-M2, H2-Q4, Sp100, H2-Aa, H2-T24, Nfkbia, Pilrb1, Bst2, Tap1, H2-T23, Cd74, Ikbke, H2-DMa, Tapbp, Irf9, Fas, H2-K1, Pilrb2, B2m, Ifih1, Tnf, H2-T22, Src, Daxx, Tnfrsf14, H2-Q5, H2-M3, Pml, Itgb3, Oas1b, Cfp, Tap2, Tmem173, H2-DMb1, Eif2ak2, Eif2ak4, Ccl2, Calr, Pik3cb, Nfkb1, AC154707.1, Il12b, Myd88, H2-D1, Zfp715, Bax, Bcl2, Tsc1, Gm9574*
2	mmu05169	Epstein-Barr virus infection-Mus musculus (mouse)	65	2.55E-13	2.90	*Irf7, Icam1, Tlr2, Ddx58, Oas3, Oas2, Cxcl10, Isg15, H2-Ab1, Stat1, Oas1g, Oas1a, H2-Eb1, H2-Q7, Stat2, H2-Q6, H2-M2, H2-Q4, Nfkb2, H2-Aa, H2-T24, Nfkbie, Nfkbia, Tap1, H2-T23, Ikbke, Itgal, H2-DMa, Tapbp, Relb, Irf9, Fas, Ccnd1, H2-K1, Cd40, B2m, Tnf, H2-T22, Entpd1, H2-Q5, H2-M3, Oas1b, Mapk14, Tap2, Jun, H2-DMb1, Sap30, Eif2ak2, Myc, Calr, Pik3cb, Stat3, Nfkb1, Nedd4, Cdk6, Myd88, Rbpj, Nfkbib, Ccnd2, Sem1, H2-D1, Bax, Bcl2, Gm9574, Gm10093*
3	mmu05165	Human papillomavirus infection-Mus musculus (mouse)	61	6.37E-06	2.72	*Oasl2, Mx1, Col1a1, Oasl1, Col1a2, Isg15, Thbs1, Stat1, Irf1, H2-Q7, Stat2, H2-Q6, H2-M2, H2-Q4, H2-T24, H2-T23, Atp6v0d2, Ikbke, Ptger4, Fzd7, Irf9, Mx2, Fas, Ccnd1, H2-K1, Atp6v0a1, Itgb7, Ptgs2, Tnf, H2-T22, H2-Q5, H2-M3, Fzd1, Itgb3, Col6a1, Fn1, Pdgfrb, Thbs2, Col6a2, Tnc, Eif2ak2, Spp1, Pik3cb, Nfkb1, Cdk6, Rbpj, Atp6v1a, Lamb1, Col4a2, Ccnd2, Itga2b, Itgav, H2-D1, Col6a3, Atp6v1b2, Creb5, Bax, Tsc1, Gm9574, Vegfa, Gm10093*
4	mmu05167	Kaposi sarcoma-associated herpesvirus infection-Mus musculus (mouse)	52	2.62E-09	2.32	*C3, Irf7, Icam1, Stat1, Cxcl2, H2-Q7, Stat2, H2-Q6, H2-M2, H2-Q4, H2-T24, Nfkbia, H2-T23, Ikbke, Irf9, Fas, Ccnd1, H2-K1, Hck, Mapkapk2, Ptgs2, Cxcl3, Pdgfb, H2-T22, Src, Gm11808, Pik3r6, Hif1a, H2-Q5, H2-M3, Cxcl1, Mapk14, Fos, Cd86, Gngt2, Jun, Eif2ak2, Rcan1, Myc, Itpr3, Pik3cb, Stat3, Nfkb1, Cdk6,*

						Plcg1, Calm3, H2-D1, Zfp36, Il6st, Bax, Gm9574, Vegfa
5	mmu04621	NOD-like receptor signaling pathway-Mus musculus (mouse)	51	6.77E-11	2.27	*Irf7, Ccl5, Gbp2, Gbp5, Ifi206, Il1b, Oas3, Oas2, Gbp3, Stat1, Oas1g, Oas1a, Cxcl2, Birc3, Stat2, Ifi204, Mefv, Nfkbia, Gbp7, Ikbke, Nlrp3, Irf9, Casp4, Cybb, Cxcl3, Tnf, Gsdmd, Cxcl1, Antxr2, Trpm2, Nampt, Oas1b, Mapk14, Ripk3, Nod2, Jun, Tmem173, Antxr1, Cyba, Ccl2, Itpr3, Txn1, Nfkb1, Myd88, Nfkbib, Rnf31, Nod1, Vdac3, Tank, Bcl2, Mcu*
6	mmu05166	Human T-cell leukemia virus 1 infection-Mus musculus (mouse)	51	5.07E-07	2.27	*Icam1, H2-Ab1, H2-Eb1, H2-Q7, H2-Q6, H2-M2, H2-Q4, Nfkb2, H2-Aa, H2-T24, Nfkbia, H2-T23, Itgal, Il2rg, H2-DMa, Relb, Ccnd1, H2-K1, Cd40, Egr2, B2m, Tnf, H2-T22, H2-Q5, H2-M3, Fos, Ets2, Jun, H2-DMb1, Myc, Tspo, Calr, Il15ra, Pik3cb, Nfkb1, Tln1, Ccnd2, Cdkn2a, Ets1, Adcy9, Slc25a5, Adcy3, H2-D1, Vdac3, Zfp36, Creb5, Adcy7, Bax, Nrp1, Spi1, Gm9574*
7	mmu04145	Phagosome-Mus musculus (mouse)	50	9.62E-10	2.23	*C3, Marco, Tlr2, H2-Ab1, Thbs1, H2-Eb1, H2-Q7, H2-Q6, H2-M2, H2-Q4, Cd14, H2-Aa, H2-T24, Tap1, Fcgr2b, H2-T23, Cd36, Atp6v0d2, H2-DMa, Fcgr1, H2-K1, C1ra, Atp6v0a1, Ncf1, Cybb, H2-T22, Rab7b, H2-Q5, H2-M3, Itgb3, Coro1a, Clec7a, Thbs2, Ncf4, Tap2, H2-DMb1, Cyba, Calr, Mrc2, Tubb2a, Sec61b, Atp6v1a, Itgav, Itgam, Scarb1, H2-D1, Atp6v1b2, M6pr, Gm9574, Ctss*
8	mmu05163	Human cytomegalovirus infection-Mus musculus (mouse)	49	2.23E-06	2.18	*Ccl5, Il1b, H2-Q7, H2-Q6, H2-M2, H2-Q4, H2-T24, Nfkbia, Tap1, H2-T23, Ptger4, Tapbp, Fas, Ccnd1, H2-K1, B2m, Ptgs2, Tnf, H2-T22, Src, H2-Q5, H2-M3, Itgb3, Mapk14, Il10ra, Tap2, Gngt2, Tmem173, Myc, Ccl2, Itpr3, Calr, Pik3cb, Stat3, Nfkb1, Rac2, Cdk6, Itgav, Cdkn2a, Adcy9, Adcy3, Calm3, H2-D1, Creb5, Adcy7, Bax, Tsc1, Gm9574, Vegfa*
9	mmu04151	PI3K-Akt signaling pathway-Mus musculus (mouse)	48	0.005424486	2.14	*Tlr2, Col1a1, Col1a2, Thbs1, Il2rg, Ccnd1, Il7r, Itgb7, Sgk1, Csf1, Pdgfb, Pik3r6, Itgb3, Lpar1, Col6a1, Fn1, Pdgfrb, Thbs2, Col6a2, Fgfr1, Gngt2, Tnc, Csf1r, Myc, Il4ra, Igf1, Spp1, Kit, Csf3r, Pik3cb, Kitl, Nfkb1, Cdk6, Lamb1, Col4a2, Ccnd2, Itga2b, Itgav, Col6a3, Ywhah, Hsp90b1, Creb5, Kdr, Bcl2, Rptor, Tsc1, Vegfa, Efna2*
10	mmu05020	Prion disease-Mus	48	0.049300566	2.14	*Ccl5, Il1b, C1qa, Ncf1, Cybb, Cav1, Tnf, Mapk14, Ncf4, Cox5a, Ndufa13, Hspa5,*

		musculus (mouse)				*Cyba, Itpr3, Ndufa1, Pik3cb, Rac2, Tubb2a, Atp5e, Il1a, Psma7, Fyn, Gm16418, Atp5g3, C1qb, Ndufa3, mt-Cytb, Uqcrq, Slc25a5, Uqcr11, Cav2, Hspa8, Sem1, Ndufs6, Vdac3, Psma6, Ndufa12, Cox7b, Uqcrb, Creb5, C1qc, mt-Nd4, Cox8a, Bax, Uqcr10, Sdhb, Psmb5, Mcu*
11	mmu04010	MAPK signaling pathway-Mus musculus (mouse)	46	0.000830673	2.05	*Il1b, Nfkb2, Cd14, Relb, Fas, Rasgrp3, Mapkapk2, Rasgrp1, Csf1, Pdgfb, Tnf, Daxx, Dusp3, Rras, Pdgfrb, Rps6ka2, Mapk14, Fos, Cacna1a, Cacng8, Fgfr1, Dusp4, Pak1, Jun, Dusp7, Csf1r, Myc, Igf1, Kit, Kitl, Nfkb1, Cacnb1, Rac2, Il1a, Myd88, Map3k20, Map4k3, Map4k1, Cacnb3, Hspa8, Arrb2, Kdr, Vegfa, Max, Taok3, Efna2*
12	mmu05164	Influenza A-Mus musculus (mouse)	45	3.80E-09	2.01	*Irf7, Ccl5, Icam1, Mx1, Rsad2, Ddx58, Il1b, Oas3, Oas2, Cxcl10, H2-Ab1, Socs3, Stat1, Oas1g, Oas1a, H2-Eb1, Stat2, H2-Aa, Nfkbia, Ikbke, Nlrp3, H2-DMa, Irf9, Mx2, Fas, Ciita, Ifih1, Tnf, Pml, Oas1b, Adar, Tnfsf10, H2-DMb1, Eif2ak2, Ccl2, Pik3cb, Nfkb1, Il12b, Cdk6, Il1a, Myd88, Nfkbib, Slc25a5, Bax, Kpna1*
13	mmu05203	Viral carcinogenesis-Mus musculus (mouse)	44	2.11E-05	1.96	*C3, Irf7, H2-Q7, H2-Q6, H2-M2, H2-Q4, Traf1, Sp100, Nfkb2, H2-T24, Nfkbia, H2-T23, Atp6v0d2, Irf9, Ccnd1, H2-K1, Mapkapk2, Egr2, H2-T22, Src, H2-Q5, H2-M3, Hpn, Jun, Eif2ak2, Pik3cb, Stat3, Nfkb1, Cdk6, Rbpj, Ccnd2, Hdac9, Cdkn2a, Snd1, H2-D1, Vdac3, Ywhah, Creb5, Il6st, Bax, Gm9574, Actn4, Actn1, Gm10093*
14	mmu05170	Human immunodeficiency virus 1 infection-Mus musculus (mouse)	43	4.82E-05	1.92	*Tlr2, H2-Q7, H2-Q6, H2-M2, H2-Q4, Apobec3, H2-T24, Nfkbia, Trim30d, Bst2, Tap1, H2-T23, Samhd1, Tapbp, Fas, H2-K1, B2m, Tnf, H2-T22, H2-Q5, H2-M3, Mapk14, Fos, Tap2, Gngt2, Pak1, Jun, Tmem173, Itpr3, Calr, Pik3cb, Nfkb1, Rac2, Myd88, Wee1, Plcg1, Calm3, H2-D1, Trim12c, Bax, Bcl2, Gm9574, Gm6180*
15	mmu05205	Proteoglycans in cancer-Mus musculus (mouse)	42	1.32E-05	1.87	*Tlr2, Col1a1, Col1a2, Thbs1, Sdc1, Fzd7, Fas, Ccnd1, Plau, Cav1, Tnf, Mmp9, Src, Hif1a, Fzd1, Itgb3, Rras, Dcn, Gab1, Hcls1, Fn1, Mapk14, Fgfr1, Pak1, Myc, Plaur, Igf1, Mmp2, Itpr3, Pik3cb, Stat3, Ppp1r12b, Il12b, Sdc4, Iqgap1, Itgav, Plcg1, Cav2, Ptpn6, Kdr, Camk2d, Vegfa*
16	mmu05162	Measles-Mus musculus	40	6.73E-09	1.78	*Irf7, Tlr2, Mx1, Ddx58, Il1b, Oas3, Oas2, Stat1, Oas1g, Oas1a, Stat2, Nfkbia,*

		(mouse)				*Fcgr2b, Ikbke, Il2rg, Irf9, Mx2, Fas, Ccnd1, Ifih1, Oas1b, Cd28, Fos, Adar, Jun, Eif2ak2, Cblb, Eif2ak4, Pik3cb, Stat3, Nfkb1, Il12b, Cdk6, Il1a, Myd88, Nfkbib, Ccnd2, Hspa8, Bax, Bcl2*
17	mmu04510	Focal adhesion-Mus musculus (mouse)	40	0.000119968	1.78	*Col1a1, Vasp, Col1a2, Thbs1, Birc3, Ccnd1, Itgb7, Parvg, Cav1, Pdgfb, Src, Itgb3, Col6a1, Fn1, Pdgfrb, Capn2, Thbs2, Col6a2, Pak1, Jun, Tnc, Igf1, Spp1, Pik3cb, Ppp1r12b, Rac2, Tln1, Fyn, Lamb1, Col4a2, Ccnd2, Itga2b, Itgav, Cav2, Col6a3, Kdr, Bcl2, Vegfa, Actn4, Actn1*
18	mmu05152	Tuberculosis-Mus musculus (mouse)	39	5.60E-06	1.74	*Clec4e, C3, Tlr2, Il1b, H2-Ab1, Stat1, H2-Eb1, Cd14, H2-Aa, Fcgr2b, Atp6v0d2, Cd74, H2-DMa, Fcgr1, Ciita, Atp6v0a1, Tnf, Src, Cebpb, Coro1a, Itgax, Mapk14, Clec7a, Il10ra, Nod2, H2-DMb1, Tlr1, Nfkb1, Mrc2, Il12b, Il1a, Myd88, Itgam, Calm3, Bax, Bcl2, Camk2d, Ctss, Fcer1g*
19	mmu04015	Rap1 signaling pathway-Mus musculus (mouse)	39	0.000374116	1.74	*Fpr1, Vasp, Thbs1, Ralgds, Itgal, Rasgrp3, Csf1, Pdgfb, Src, P2ry1, Itgb3, Lpar1, Rras, Lcp2, Pdgfrb, Adora2a, Mapk14, Fgfr1, Csf1r, Igf1, Kit, Pik3cb, Arap3, Kitl, Apbb1ip, Rac2, Tln1, Itga2b, Pfn1, Itgam, Plcg1, Adora2b, Adcy9, Adcy3, Calm3, Adcy7, Kdr, Vegfa, Efna2*
20	mmu05132	Salmonella infection-Mus musculus (mouse)	39	0.01395924	1.74	*Tlr2, Il1b, Birc3, Cd14, Nfkbia, Nlrp3, Casp4, Tnf, Rhoj, Elmo1, Gsdmd, Rab7b, Rras, Mlkl, Mapk14, Fos, Ripk3, Tnfsf10, Pak1, Jun, Plekhm1, Myc, Txn1, Pik3cb, Nfkb1, Myd88, Tlr5, Pfn1, Nod1, Arpc1b, Rhog, Hsp90b1, Cyfip2, Nckap1l, Bax, Bcl2, M6pr, Kpna1, Arpc4*
21	mmu04514	Cell adhesion molecules-Mus musculus (mouse)	38	1.38E-09	1.69	*Icam1, H2-Ab1, H2-Eb1, H2-Q7, Sdc1, H2-Q6, H2-M2, H2-Q4, H2-Aa, H2-T24, Icosl, Spn, H2-T23, Itgal, H2-DMa, H2-K1, Cd40, Itgb7, H2-T22, H2-Q5, H2-M3, Alcam, Cd28, Cd86, Nectin2, Vcan, H2-DMb1, Cd274, Vsir, Cadm1, Siglec1, Sdc4, Itgav, Itgam, Sdc3, H2-D1, CAAA01147332.1, Gm9574*
22	mmu04060	Cytokine-cytokine receptor interaction-Mus musculus (mouse)	38	0.000140566	1.69	*Ccl5, Cx3cr1, Il1b, Cxcl10, Il21r, Cxcl2, Ccr2, Il2rg, Cxcl16, Fas, Il7r, Cd40, Csf1, Cxcl3, Tnf, Csf2rb, Ccl9, Tnfrsf14, Cxcl9, Cxcl1, Il10ra, Tnfsf10, Ppbp, Csf1r, Il4ra, Ccl2, Csf3r, Il15ra, Il12b, Il12rb1, Il1a, Inhbb, Relt, Ccl22, Il6st, Il13ra1, Ltb, Ccl7*
23	mmu05418	Fluid shear stress and atherosclerosis-Mus	37	1.03E-05	1.65	*Icam1, Ass1, Il1b, Sdc1, Gm5424, Ncf1, Cav1, Pdgfb, Tnf, Mmp9, Src, Itgb3, Mapk14, Fos, Jun, Thbd, Cyba, Mmp2, Ccl2, Txn1, Pik3cb, Nfkb1, Rac2, Sdc4,*

		musculus (mouse)				*Il1a, Itga2b, Itgav, Hmox1, Gsta3, Cav2, Calm3, Hsp90b1, Kdr, Bcl2, Nfe2l2, Vegfa, Klf2*
24	mmu05206	MicroRNAs in cancer-Mus musculus (mouse)	35	7.77E-05	1.56	*Thbs1, Ccnd1, Plau, Fscn1, Ptgs2, Pdgfb, Mmp9, Itgb3, Pdgfrb, Dnmt3a, Cyp1b1, Tnc, Kif23, Myc, Pim1, St14, Bmf, Pik3cb, Stat3, Nfkb1, Cdk6, Ccnd2, Irs2, Marcks, Cdkn2a, Plcg1, Hmox1, Socs1, Sox4, Bcl2, Rptor, Abcb1b, Vegfa, Efna2, Gm10093*
25	mmu04062	Chemokine signaling pathway-Mus musculus (mouse)	35	0.000146578	1.56	*Ccl5, Cx3cr1, Cxcl10, Stat1, Cxcl2, Stat2, Fgr, Nfkbia, Ccr2, Cxcl16, Hck, Ncf1, Cxcl3, Src, Pik3r6, Elmo1, Ccl9, Cxcl9, Cxcl1, Gngt2, Pak1, Ppbp, Ccl2, Pik3cb, Stat3, Nfkb1, Rac2, Nfkbib, Plcg1, Adcy9, Adcy3, Ccl22, Adcy7, Arrb2, Ccl7*
26	mmu05202	Transcriptional misregulation in cancer-Mus musculus (mouse)	33	0.003563379	1.47	*Birc3, Traf1, Cd14, Fcgr1, Nupr1, Plau, Cd40, Dot1l, Itgb7, Mmp9, Pparg, Cebpb, Pml, Etv5, Cd86, Cebpa, Csf1r, Myc, Igf1, Nfkb1, Jmjd1c, Eya1, Ccnd2, Cdk14, Nfkbiz, Itgam, Hpgd, Bcl2a1a, Bax, Bcl6, Spi1, Max, Gm10093*
27	mmu04625	C-type lectin receptor signaling pathway-Mus musculus (mouse)	32	2.56E-07	1.43	*Clec4e, Il1b, Stat1, Bcl3, Irf1, Clec4n, Stat2, Nfkb2, Nfkbia, Ikbke, Nlrp3, Relb, Irf9, Mapkapk2, Egr2, Ptgs2, Tnf, Src, Rras, Mapk14, Clec7a, Pak1, Jun, Cblb, Itpr3, Pik3cb, Nfkb1, Il12b, Clec4d, Calm3, Ccl22, Fcer1g*
28	mmu04668	TNF signaling pathway-Mus musculus (mouse)	32	4.81E-07	1.43	*Ccl5, Icam1, Mmp14, Ifi47, Il1b, Cxcl10, Socs3, Bcl3, Cxcl2, Irf1, Birc3, Traf1, Nfkbia, Fas, Ptgs2, Csf1, Gm5431, Cxcl3, Tnf, Mmp9, Cebpb, Cxcl1, Mlkl, Mapk14, Fos, Ripk3, Nod2, Jun, Ccl2, Pik3cb, Nfkb1, Creb5*
29	mmu04380	Osteoclast differentiation-Mus musculus (mouse)	32	1.12E-05	1.43	*Il1b, Socs3, Stat1, Stat2, Nfkb2, Nfkbia, Fcgr2b, Fcgr1, Relb, Irf9, Ncf1, Csf1, Tnf, Pparg, Itgb3, Lcp2, Mapk14, Fos, Ncf4, Sirpb1c, Acp5, Jun, Lilrb4a, Csf1r, Cyba, Pik3cb, Nfkb1, Il1a, Fyn, Socs1, Tec, Spi1*
30	mmu05160	Hepatitis C-Mus musculus (mouse)	32	0.000108231	1.43	*Irf7, Ifit1, Mx1, Rsad2, Ddx58, Oas3, Oas2, Cxcl10, Socs3, Stat1, Oas1g, Oas1a, Stat2, Nfkbia, Ikbke, Irf9, Mx2, Fas, Ccnd1, Tnf, Ifit1bl1, Oas1b, Eif2ak2, Eif2ak4, Myc, Pik3cb, Stat3, Nfkb1, Cdk6, Scarb1, Ywhah, Bax*
31	mmu04218	Cellular senescence-Mus musculus (mouse)	32	0.009287756	1.43	*H2-Q7, H2-Q6, H2-M2, H2-Q4, H2-T24, H2-T23, Ccnd1, H2-K1, Mapkapk2, H2-T22, H2-Q5, H2-M3, Rras, Serpine1, Capn2, Mapk14, Myc, Itpr3, Pik3cb, Nfkb1, Cdk6, Il1a, Ccnd2, Cdkn2a, Ets1, Slc25a5, Calm3, H2-D1, Vdac3, Mcu,*

						Tsc1, Gm9574
32	mmu04612	Antigen processing and presentation-Mus musculus (mouse)	31	2.62E-09	1.38	*H2-Ab1, H2-Eb1, H2-Q7, H2-Q6, H2-M2, H2-Q4, H2-Aa, H2-T24, Tap1, H2-T23, Cd74, H2-DMa, Tapbp, H2-K1, Ciita, Psme2, Psme2b, B2m, Psme1, Tnf, H2-T22, H2-Q5, H2-M3, Tap2, H2-DMb1, Hspa5, Calr, Hspa8, H2-D1, Gm9574, Ctss*
33	mmu05140	Leishmaniasis-Mus musculus (mouse)	29	4.31E-09	1.29	*C3, Marcksl1, Tlr2, Il1b, H2-Ab1, Stat1, H2-Eb1, H2-Aa, Nfkbia, H2-DMa, Fcgr1, Ncf1, Cybb, Ptgs2, Gm6548, Tnf, Mapk14, Fos, Ncf4, Jun, H2-DMb1, Cyba, Nfkb1, Il12b, Il1a, Myd88, Nfkbib, Itgam, Ptpn6*
34	mmu05416	Viral myocarditis-Mus musculus (mouse)	28	5.51E-09	1.25	*Icam1, H2-Ab1, H2-Eb1, H2-Q7, H2-Q6, H2-M2, H2-Q4, H2-Aa, H2-T24, H2-T23, Itgal, H2-DMa, Ccnd1, H2-K1, Cd40, Cav1, H2-T22, H2-Q5, H2-M3, Cd55, Cd28, Cd86, H2-DMb1, Rac2, Fyn, H2-D1, Dag1, Gm9574*
35	mmu05323	Rheumatoid arthritis-Mus musculus (mouse)	28	2.34E-08	1.25	*Ccl5, Icam1, Tlr2, Il1b, H2-Ab1, Cxcl2, H2-Eb1, H2-Aa, Atp6v0d2, Itgal, H2-DMa, Atp6v0a1, Csf1, Cxcl3, Tnf, Cxcl1, Cd28, Fos, Cd86, Acp5, Jun, H2-DMb1, Ccl2, Il1a, Atp6v1a, Atp6v1b2, Vegfa, Ltb*
36	mmu04630	JAK-STAT signaling pathway-Mus musculus (mouse)	28	0.000206919	1.25	*Il21r, Socs3, Stat1, Stat2, Il2rg, Irf9, Ccnd1, Il7r, Pdgfb, Csf2rb, Pdgfrb, Il10ra, Myc, Pim1, Il4ra, Csf3r, Il15ra, Pik3cb, Stat3, Il12b, Il12rb1, Cish, Ccnd2, Socs1, Il6st, Ptpn6, Bcl2, Il13ra1*
37	mmu04020	Calcium signaling pathway-Mus musculus (mouse)	28	0.010512235	1.25	*Orai2, Ednrb, Pdgfb, Mcoln2, Pdgfrb, Adora2a, Mcoln3, Cysltr1, Cacna1a, Phka2, Asph, Cd38, Adra1a, Itpr3, Tpcn2, Tpcn1, Plcg1, Adora2b, Itpkb, Adcy9, Slc25a5, Adcy3, P2rx4, Calm3, Vdac3, Adcy7, Camk2d, Mcu*
38	mmu04933	AGE-RAGE signaling pathway in diabetic complications-Mus musculus (mouse)	26	2.85E-05	1.16	*Icam1, Col1a1, Il1b, Col1a2, Stat1, Col3a1, Ccnd1, Cybb, Tnf, Serpine1, Fn1, Mapk14, Jun, Thbd, Pim1, Mmp2, Ccl2, Pik3cb, Stat3, Nfkb1, Il1a, Col4a2, Plcg1, Bax, Bcl2, Vegfa*
39	mmu05145	Toxoplasmosis-Mus musculus (mouse)	26	0.00025262	1.16	*Tlr2, H2-Ab1, Stat1, H2-Eb1, Birc3, H2-Aa, Nfkbia, Irgm2, H2-DMa, Igtp, Ciita, Cd40, Tnf, Pik3r6, Mapk14, Il10ra, H2-DMb1, Stat3, Nfkb1, Il12b, Myd88, Nfkbib, Lamb1, Socs1, Hspa8, Bcl2*
40	mmu05161	Hepatitis B-Mus	26	0.043610621	1.16	*Irf7, Tlr2, Ddx58, Stat1, Stat2, Nfkbia, Ikbke, Fas, Egr2, Ifih1, Tnf, Mmp9, Src,*

		musculus (mouse)				*Pcna, Mapk14, Fos, Jun, Myc, Pik3cb, Stat3, Nfkb1, Myd88, Vdac3, Creb5, Bax, Bcl2*
41	mmu04640	Hematopoietic cell lineage-Mus musculus (mouse)	25	3.64E-06	1.11	*Il1b, H2-Ab1, H2-Eb1, Cd14, H2-Aa, Cd36, H2-DMa, Fcgr1, Il7r, Csf1, Tnf, Itgb3, Cd55, Cd9, H2-DMb1, Cd38, Csf1r, Il4ra, Kit, Csf3r, Kitl, Il1a, Itga2b, Itgam, Gp5*
42	mmu04611	Platelet activation-Mus musculus (mouse)	25	0.003659443	1.11	*Col1a1, Vasp, Col1a2, Col3a1, Rasgrp1, Src, Pik3r6, P2ry1, Itgb3, Lcp2, Mapk14, Itpr3, Vamp8, Pik3cb, Apbb1ip, Tln1, Fyn, Ptgir, Itga2b, Adcy9, Adcy3, Adcy7, Ptgs1, Gp5, Fcer1g*
43	mmu04940	Type I diabetes mellitus-Mus musculus (mouse)	24	2.62E-09	1.07	*Il1b, H2-Ab1, H2-Eb1, H2-Q7, H2-Q6, H2-M2, H2-Q4, H2-Aa, H2-T24, H2-T23, H2-DMa, Fas, H2-K1, Tnf, H2-T22, H2-Q5, H2-M3, Cd28, Cd86, H2-DMb1, Il12b, Il1a, H2-D1, Gm9574*
44	mmu05330	Allograft rejection-Mus musculus (mouse)	23	5.15E-10	1.03	*H2-Ab1, H2-Eb1, H2-Q7, H2-Q6, H2-M2, H2-Q4, H2-Aa, H2-T24, H2-T23, H2-DMa, Fas, H2-K1, Cd40, Tnf, H2-T22, H2-Q5, H2-M3, Cd28, Cd86, H2-DMb1, Il12b, H2-D1, Gm9574*
45	mmu05332	Graft-versus-host disease-Mus musculus (mouse)	23	6.76E-10	1.03	*Il1b, H2-Ab1, H2-Eb1, H2-Q7, H2-Q6, H2-M2, H2-Q4, H2-Aa, H2-T24, H2-T23, H2-DMa, Fas, H2-K1, Tnf, H2-T22, H2-Q5, H2-M3, Cd28, Cd86, H2-DMb1, Il1a, H2-D1, Gm9574*
46	mmu04620	Toll-like receptor signaling pathway-Mus musculus (mouse)	23	0.000108231	1.03	*Irf7, Ccl5, Tlr2, Il1b, Cxcl10, Stat1, Cd14, Nfkbia, Ikbke, Cd40, Tnf, Cxcl9, Mapk14, Fos, Cd86, Jun, Tlr1, Spp1, Pik3cb, Nfkb1, Il12b, Myd88, Tlr5*
47	mmu05146	Amoebiasis-Mus musculus (mouse)	23	0.000330325	1.03	*Tlr2, Col1a1, Il1b, Col1a2, Cxcl2, Cd14, Col3a1, Arg2, Cxcl3, Tnf, Serpinb6b, Rab7b, Cxcl1, Fn1, Pik3cb, Nfkb1, Il12b, Lamb1, Col4a2, Gm21399, Itgam, Actn4, Actn1*
48	mmu04080	Neuroactive ligand-receptor interaction-Mus musculus (mouse)	23	0.036471853	1.03	*C3, Fpr2, Fpr1, Ednrb, Ptger4, S1pr1, P2ry1, Lpar1, P2ry10b, P2ry14, Adora2a, Cysltr1, C3ar1, Adra1a, P2ry13, Tspo, C5ar1, Cnr2, Ptgir, S1pr2, Adora2b, P2rx4, Gabbr2*
49	mmu04512	ECM-receptor interaction-Mus	22	5.35E-06	0.98	*Col1a1, Col1a2, Thbs1, Sdc1, Cd36, Itgb7, Agrn, Itgb3, Col6a1, Fn1, Thbs2, Col6a2, Tnc, Spp1, Sdc4, Lamb1, Col4a2, Itga2b, Itgav, Col6a3, Dag1, Gp5*

		musculus (mouse)				
50	mmu04064	NF-kappa B signaling pathway-Mus musculus (mouse)	22	0.001733079	0.98	*Icam1, Ddx58, Il1b, Cxcl2, Birc3, Traf1, Nfkb2, Cd14, Nfkbia, Relb, Plau, Cd40, Ptgs2, Cxcl3, Tnf, Cxcl1, Nfkb1, Myd88, Plcg1, Bcl2a1a, Bcl2, Ltb*
51	mmu04659	Th17 cell differentiation-Mus musculus (mouse)	22	0.003197046	0.98	*Il1b, H2-Ab1, Il21r, Stat1, H2-Eb1, H2-Aa, Nfkbie, Nfkbia, Il2rg, H2-DMa, Hif1a, Mapk14, Fos, Jun, H2-DMb1, Il4ra, Stat3, Nfkb1, Il12rb1, Nfkbib, Plcg1, Il6st*
52	mmu04926	Relaxin signaling pathway-Mus musculus (mouse)	22	0.01395924	0.98	*Col1a1, Col1a2, Nfkbia, Col3a1, Ednrb, Mmp9, Acta2, Src, Mapk14, Fos, Gngt2, Jun, Mmp2, Pik3cb, Nfkb1, Col4a2, Adcy9, Adcy3, Creb5, Adcy7, Arrb2, Vegfa*
53	mmu05320	Autoimmune thyroid disease-Mus musculus (mouse)	21	2.62E-09	0.94	*H2-Ab1, H2-Eb1, H2-Q7, H2-Q6, H2-M2, H2-Q4, H2-Aa, H2-T24, H2-T23, H2-DMa, Fas, H2-K1, Cd40, H2-T22, H2-Q5, H2-M3, Cd28, Cd86, H2-DMb1, H2-D1, Gm9574*
54	mmu04061	Viral protein interaction with cytokine and cytokine receptor-Mus musculus (mouse)	21	1.12E-05	0.94	*Ccl5, Cx3cr1, Cxcl10, Cxcl2, Ccr2, Il2rg, Csf1, Cxcl3, Tnf, Ccl9, Tnfrsf14, Cxcl9, Cxcl1, Il10ra, Tnfsf10, Ppbp, Csf1r, Ccl2, Ccl22, Il6st, Ccl7*
55	mmu05133	Pertussis-Mus musculus (mouse)	21	9.77E-05	0.94	*C3, Il1b, Irf1, Cd14, Nlrp3, C1qa, C1ra, Tnf, Mapk14, Fos, Jun, Nfkb1, Il12b, Il1a, Myd88, Nod1, Itgam, C1qb, Calm3, C1qc, Gm6180*
56	mmu05150	Staphylococcus aureus infection-Mus musculus (mouse)	20	2.25E-08	0.89	*C3, Icam1, Fpr2, Fpr1, H2-Ab1, H2-Eb1, H2-Aa, Fcgr2b, Itgal, H2-DMa, C1qa, Fcgr1, C1ra, Cfb, H2-DMb1, C3ar1, C5ar1, Itgam, C1qb, C1qc*
57	mmu05321	Inflammatory bowel disease-Mus musculus (mouse)	20	2.79E-06	0.89	*Tlr2, Il1b, H2-Ab1, Il21r, Stat1, H2-Eb1, H2-Aa, Il2rg, H2-DMa, Tnf, Nod2, Jun, H2-DMb1, Il4ra, Stat3, Nfkb1, Il12b, Il12rb1, Il1a, Tlr5*
58	mmu05235	PD-L1 expression and PD-1 checkpoint pathway in cancer-Mus	20	0.003197046	0.89	*Tlr2, Stat1, Nfkbie, Nfkbia, Rasgrp1, Batf2, Hif1a, Cd28, Mapk14, Batf, Fos, Jun, Cd274, Pik3cb, Stat3, Nfkb1, Myd88, Nfkbib, Plcg1, Ptpn6*

		musculus (mouse)				
59	mmu05142	Chagas disease-Mus musculus (mouse)	20	0.006560707	0.89	*C3, Ccl5, Tlr2, Il1b, Nfkbia, C1qa, Fas, Tnf, Serpine1, Mapk14, Fos, Jun, Ccl2, Calr, Pik3cb, Nfkb1, Il12b, Myd88, C1qb, C1qc*
60	mmu04610	Complement and coagulation cascades-Mus musculus (mouse)	19	1.32E-05	0.85	*C3, F13a1, C1qa, Plau, C1ra, Serpine1, Itgax, Cfb, Cd55, F10, C3ar1, Thbd, Plaur, C5ar1, Itgam, C1qb, F7, C1qc, Procr*
61	mmu04657	IL-17 signaling pathway-Mus musculus (mouse)	19	0.003197046	0.85	*Il1b, Cxcl10, Cxcl2, Nfkbia, Ikbke, Lcn2, Ptgs2, Cxcl3, Tnf, Mmp9, Cebpb, Cxcl1, Mapk14, Fos, Jun, Ccl2, Nfkb1, Hsp90b1, Ccl7*
62	mmu04658	Th1 and Th2 cell differentiation-Mus musculus (mouse)	19	0.003197046	0.85	*H2-Ab1, Stat1, H2-Eb1, H2-Aa, Nfkbie, Nfkbia, Il2rg, H2-DMa, Mapk14, Fos, Jun, H2-DMb1, Il4ra, Nfkb1, Il12b, Il12rb1, Rbpj, Nfkbib, Plcg1*
63	mmu04660	T cell receptor signaling pathway-Mus musculus (mouse)	19	0.02621427	0.85	*Nfkbie, Nfkbia, Rasgrp1, Tnf, Lcp2, Cd28, Mapk14, Fos, Pak1, Jun, Cblb, Pik3cb, Nfkb1, Nck2, Nfkbib, Fyn, Plcg1, Ptpn6, Tec*
64	mmu05134	Legionellosis-Mus musculus (mouse)	18	0.021228787	0.80	*C3, Tlr2, Il1b, Cxcl2, Nfkb2, Cd14, Nfkbia, Gm6548, Cxcl3, Tnf, Cxcl1, Nfkb1, Il12b, Myd88, Tlr5, Itgam, Hspa8, Gm4366*
65	mmu05322	Systemic lupus erythematosus-Mus musculus (mouse)	18	0.043610621	0.80	*C3, H2-Ab1, H2-Eb1, H2-Aa, H2-DMa, C1qa, Fcgr1, Cd40, C1ra, Tnf, Cd28, Cd86, H2-DMb1, Trim21, C1qb, C1qc, Actn4, Actn1*
66	mmu04622	RIG-I-like receptor signaling pathway-Mus musculus (mouse)	16	0.000406696	0.71	*Irf7, Ddx58, Cxcl10, Isg15, Dhx58, Nfkbia, Ikbke, Ifih1, Tnf, Mapk14, Tmem173, Nfkb1, Il12b, Nfkbib, Otud5, Tank*
67	mmu05221	Acute myeloid leukemia-Mus musculus (mouse)	15	0.026118345	0.67	*Cd14, Fcgr1, Ccnd1, Pml, Cebpa, Csf1r, Myc, Pim1, Kit, Pik3cb, Stat3, Nfkb1, Itgam, Bcl2a1a, Spi1*
68	mmu04974	Protein digestion and absorption-Mus	14	0.041605439	0.62	*Col1a1, Col1a2, Col3a1, Col5a1, Fxyd2, Col5a2, Col6a1, Col6a2, Col5a3, Col4a2, Col12a1, Col6a3, Col8a1, Slc7a15*

		musculus (mouse)				
69	mmu05144	Malaria-Mus musculus (mouse)	13	0.001322142	0.58	*Icam1, Tlr2, Il1b, Thbs1, Sdc1, Cd36, Itgal, Cd40, Tnf, Thbs2, Lrp1, Ccl2, Myd88*
70	mmu04979	Cholesterol metabolism-Mus musculus (mouse)	13	0.001649619	0.58	*Cd36, Lpl, Ldlrap1, Lrp1, Lipa, Sort1, Nceh1, Tspo, Angptl4, Scarb1, Vdac3, Npc1, Soat1*
71	mmu04623	Cytosolic DNA-sensing pathway-Mus musculus (mouse)	13	0.007145902	0.58	*Irf7, Ccl5, Zbp1, Ddx58, Il1b, Cxcl10, Nfkbia, Ikbke, Ripk3, Adar, Tmem173, Nfkb1, Nfkbib*
72	mmu04917	Prolactin signaling pathway-Mus musculus (mouse)	13	0.02621427	0.58	*Socs3, Stat1, Irf1, Ccnd1, Src, Mapk14, Fos, Pik3cb, Stat3, Nfkb1, Cish, Ccnd2, Socs1*
73	mmu04672	Intestinal immune network for IgA production-Mus musculus (mouse)	11	0.00534305	0.49	*H2-Ab1, H2-Eb1, H2-Aa, Icosl, H2-DMa, Cd40, Itgb7, Cd28, Cd86, H2-DMb1, Il15ra*
74	mmu00760	Nicotinate and nicotinamide metabolism-Mus musculus (mouse)	9	0.021399855	0.40	*Nadk, Pnp, Nampt, Bst1, Cd38, Enpp1, Pnp2, Nt5c2, Art2a-ps*
75	mmu05310	Asthma-Mus musculus (mouse)	8	0.000508203	0.36	*H2-Ab1, H2-Eb1, H2-Aa, H2-DMa, Cd40, Tnf, H2-DMb1, Fcer1g*
