# Supplementary material for: Prolactin-Releasing Peptide Differentially Regulates Gene Transcriptomic Profiles in Mouse Bone Marrow-Derived Macrophages
Source: Int J Mol Sci. 2021 Apr 24;22(9):4456. doi: 10.3390/ijms22094456 (PMC8123224; doi:10.3390/ijms22094456)
Supplement: Supplementary file 1 [file ijms-22-04456-s001.zip › Supplementary File 4_Table S3 RMSD-RMSF-Rg.pdf]

Table S3. Average value of RMSD, RMSF, and Rg

Protein	RMSD	RMSF	Rg
Ifit1	0.7154	0.2412	2.738
Oasl2	0.7572	0.3507	2.449
Irf7	0.9027	0.3929	2.235
Ifit3	1.082	0.4669	2.643
Ifit2	0.5803	0.2438	2.705
Usp18	0.3669	0.2118	2.119
Ifi44	0.9566	0.6008	2.836
Rtp4	0.8615	0.3364	1.943
